# Gold(I)-Catalyzed Synthesis of 3-Sulfenyl Pyrroles and Indoles by a Regioselective Annulation of Alkynyl Thioethers

**DOI:** 10.1021/acscatal.1c01457

**Published:** 2021-05-13

**Authors:** Peter E. Simm, Prakash Sekar, Jeffery Richardson, Paul W. Davies

**Affiliations:** † School of Chemistry, University of Birmingham, Edgbaston, Birmingham B15 2TT, U.K.; ‡ Lilly U.K., Erl Wood Manor, Windlesham GU20 6PH, U.K.

**Keywords:** gold, pyrroles, nitrenoid, annulation, thioether, indole

## Abstract

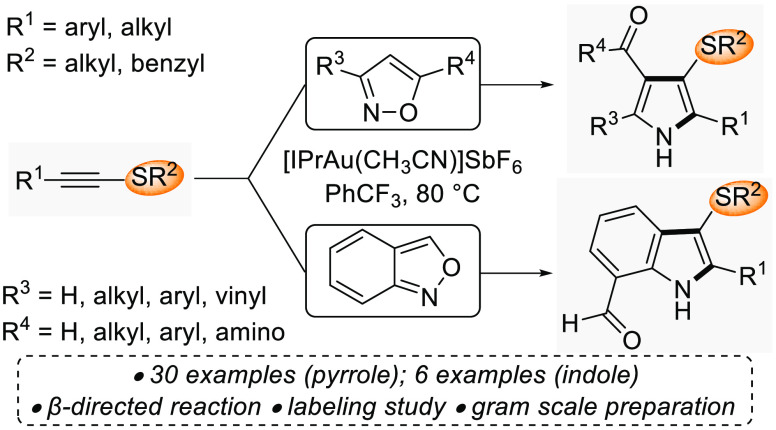

The combination of nucleophilic nitrenoids and π-acid catalysis has emerged as a powerful tool in heterocycle synthesis. Accessing more varied heterocycle-substitution patterns by maintaining the same reaction pathways across different alkynes remains a challenge. Here we show that Au(I) catalysis of isoxazole-based nitrenoids with alkynyl thioethers provides controlled access to (3 + 2) annulation by a regioselective addition β to the sulfenyl group. The reaction with isoxazole-containing nitrenoids delivers sulfenylated pyrroles and indoles as single regioisomers bearing useful functional groups and structural variety.

N-nucleophilic nitrenoids have proved to be versatile surrogates for nitrene-containing 1,3-dipoles, providing modular and expedient access to a variety of densely functionalized N-heterocycles by a formal (3 + 2) cycloaddition to gold-activated alkynes.^1^ Most of these annulations were first realized with ynamides,^2^ and to date there are only a few specific instances where those same transformations have been achieved with other types of alkynes.^2j,3^ As the alkyne substitution pattern is directly translated into the product, retaining the same transformation across different alkyne types would greatly expand the potential of these convergent annulation methods for complex molecule synthesis. A challenge lies in finding alkynes that are sufficiently reactive and do not change how the reaction pathway evolves while providing useful substitution patterns.

The use of isoxazole-based N-nucleophilic nitrenoids is illustrative: The groups of Ye^2f^ and Hashmi^2j^ demonstrated the potential of using isoxazoles and [2,1]benzisoxazoles, respectively, to assemble pyrroles and indoles through a formal (3 + 2) cycloaddition across C-aryl ynamides (Figure 1b). Several groups have subsequently established that a tremendous variety of heterocyclic structures can be formed when these nitrenoids are combined with differently substituted alkynes.^4^ Because the alkyne structure affects the nature of the intermediate α-imino gold carbene **B** and the pathways through which it can evolve (Figure 1a), even relatively small changes can divert the pathway away from formal (3 + 2) cycloaddition (Figure 1c).

**Figure 1:**
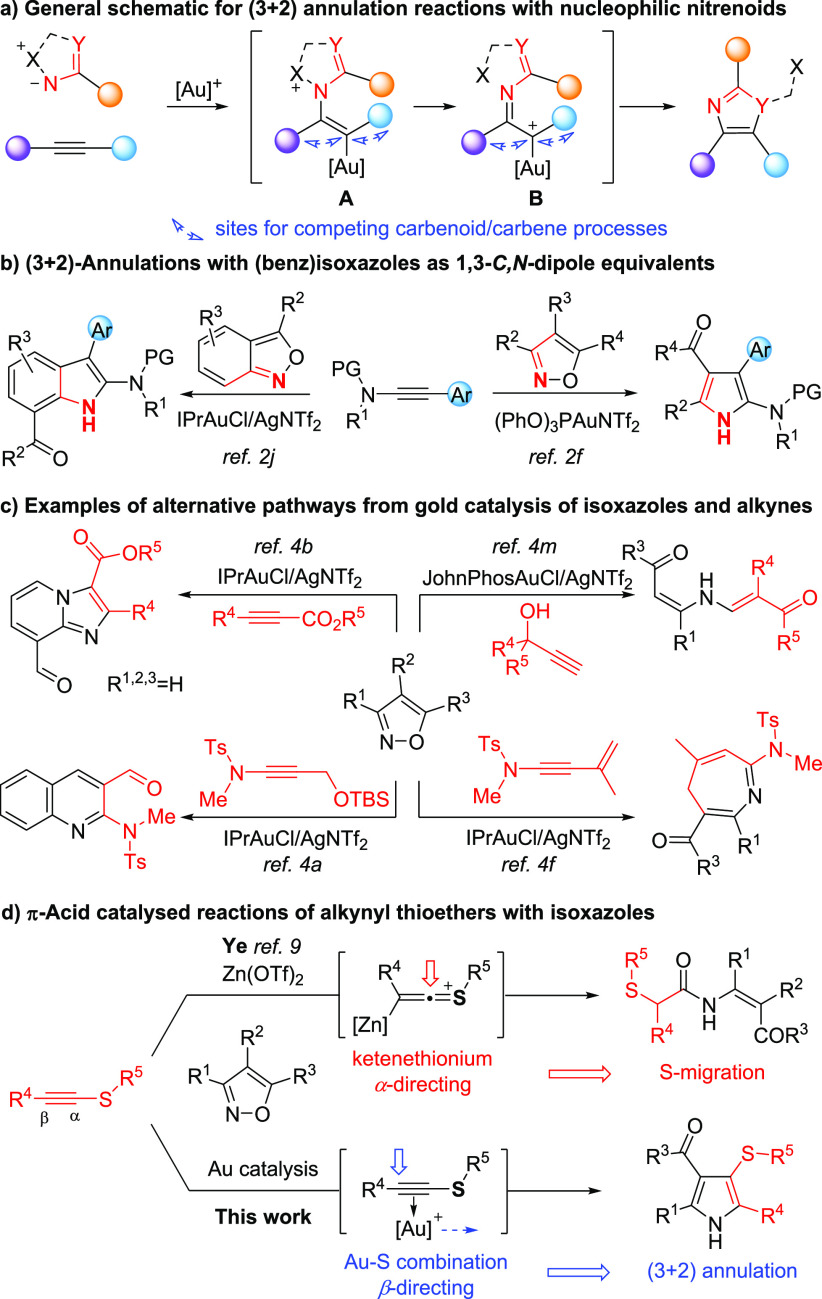
Divergent outcomes for the reaction of alkynes and isoxazoles under π-acid catalysis.

Few reports have addressed gold-catalyzed intermolecular reactions with alkynyl thioethers, and the majority have reported nucleophilic addition α to the sulfur,^5^ invoking a dominant gold ketenethionium character. However, a recent study from our group using N-acylpyridinium aminides showed that, in the presence of a Au(III) precatalyst, the outcomes matched selective attack at the β-position of alkynyl thioethers.^3b^ We reasoned that if this latter route was generally accessible from other types of nitrenoids, especially those accessed using Au(I) catalysis, then a more generalized approach for (3 + 2)-type annulations with nitrenoids could be realized. Replicating this reactivity would ensure that the aurated carbon is substituted by the sulfenyl group throughout the reaction manifold, potentially facilitating a more consistent reactivity profile that tolerates modifications elsewhere. Sulfur-substituted heterocycles are desirable, not least in medicinal chemistry.^6,7^ The increasing number of C–S functionalization methods renders them potentially useful substrates for further elaboration.^8^ The prospective utility of sulfenylated pyrrole and indole products combined with the diversity of reaction pathways that can be accessed from (benz)isoxazoles identified them as ideal systems for probing the wider utility of alkynyl thioethers in nitrenoid-based annulations.

During our studies Ye and co-workers reported the Zn(II)-catalyzed reaction of alkynyl thioethers with isoxazoles, where selective α-addition led to sulfur-substituted β-keto enamides with 1,2-S migration (Figure 1d).^9^ Here we show that the Au(I)-catalyzed reaction of alkynyl thioethers with isoxazoles and [2,1]benzisoxazoles proceeds regioselectively by β-addition to access the formal (3 + 2) cycloaddition pathway.

We found that using alkynyl thioether **1a** and 3,5-dimethylisoxazole **2a** gave rise to regioselective formation of the 3-sulfenylated pyrrole **3a** under gold catalysis. The finalized conditions employ a 1:2 stoichiometry of **1a** to **2a** in 1,1,1-trifluorotoluene at 80 °C in the presence of [IPrAu(CH_3_CN)]SbF_6_ in a capped vial and no measures taken to exclude air and moisture (Scheme 1; see the Supporting Information for an optimization survey). No reaction was seen in the absence of catalyst or on replacing it with a strong Brønsted acid, while gold(I) and gold(III) catalysts with other ligand combinations were less effective. The noncoordinating counterions tetrakis[3,5-bis(trifluoromethyl)phenyl]borate and hexafluoroantimonate were optimal in comparison to weakly coordinating alternatives. Only a small reduction in yield is seen at lower temperatures or with a lower stoichiometry of **2a**.

**Scheme 1:**
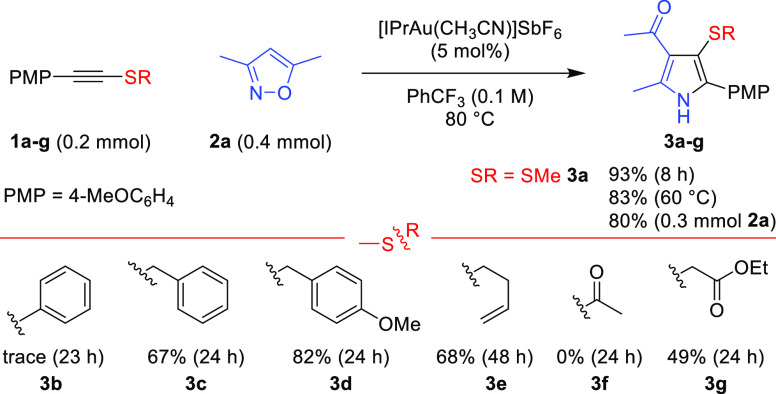
Pyrrole Formation from Alkynyl Thioethers: S-Substituent

The effects of different S-substituents on the annulation were tested (Scheme 1). While an *S*-phenyl substituent stalled the process, presumably due to its rigid steric bulk, *S-*benzylated systems were reactive (**3c**,**d**). 1,5-Enynyl thioether **1e** gave solely the pyrrole (**3e**), despite the potential for intramolecular cyclopropanation at an intermediate α-imino gold carbene.^4k^ The *S*-alkynyl thioester **1f** degraded under the reaction conditions, but the 2-(ethynylthio)acetate derivative **1g** afforded pyrrole **3g**.

The wider scope of the annulation was then explored (Scheme 2). The reaction was largely invariant to steric bulk at the C-terminus of the alkynyl thioether, allowing ortho and diortho substitution (**3h**–**j**). A single-crystal X-ray diffraction analysis confirmed the structure of **3j**. Electron-donating substituents on the aryl group are beneficial but not required (**3h**–**q**). An aryl fluoride and an aniline derivative worked well, as did heteroaromatic groups such as indole and thiophene (**3m**–**p**). Alkyl-substituted alkynyl thioethers reacted sluggishly but did give the desired 3-sulfenylated pyrroles **3r**,**s** in serviceable yields.

**Scheme 2:**
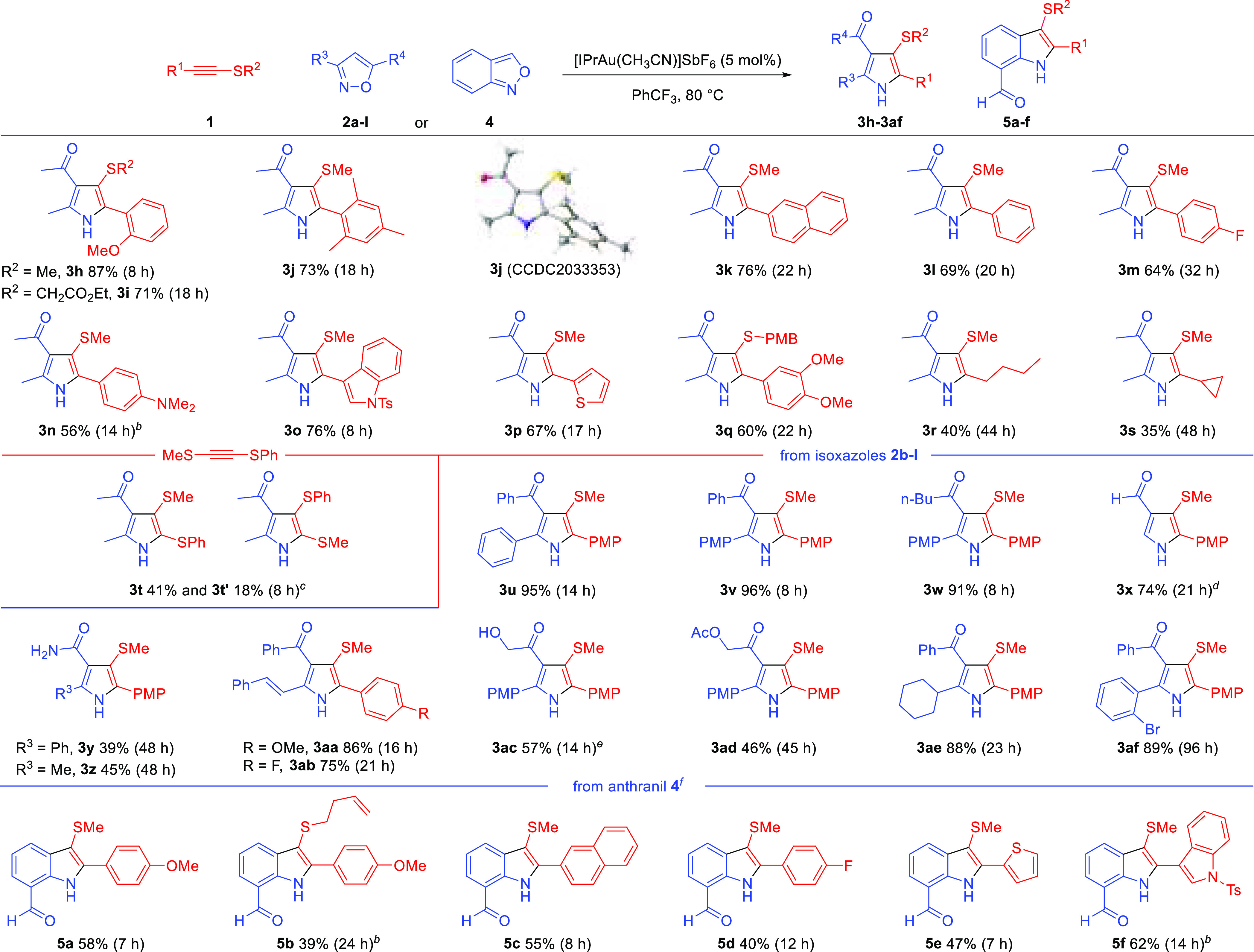
Au(I)-Catalyzed Synthesis of Pyrroles and Indoles from Alkynyl Thioethers Reactions unless stated otherwise were run in capped vials with no precautions against air or moisture with alkynyl thioethers (1.0 equiv), **2a**–**l** (2.0 equiv), [IPrAu(CH_3_CN)]SbF_6_ (5 mol %), and PhCF_3_ (0.1 M) at 80 °C. The crystal structure of **3j** is shown with ellipsoids drawn at the 50% probability level. Reaction at 50 °C; 53% of **3n** at 80 °C. ^1^H NMR spectroscopic analysis of the reaction mixture elucidated a 43% yield of **3t** and 19% of the putative **3t′**. The regioisomerism of **3t** was confirmed by ^1^H NOESY. 4 equiv of isoxazole. 0.4 mmol of **1a** with 0.2 mmol of **2i**. **4** was used as the limiting reagent to **1**.

An unsymmetrical acetylene disulfide reacted to give the 2,3-bis-sulfenyl pyrroles as a mixture of regioisomers **3t**,**t′**. The regioselective preference correlates to the relative reactivity seen on changing the sulfenyl group in alkynyl thioethers (cf. Scheme 1, **1a**,**b**), the major product arising from C–N bond formation β to the smaller sulfenyl group, although the regioisomer **3t′** was formed despite the recalcitrance of **1b**. Neither triisopropylsilyl nor terminal alkynyl thioethers gave productive reactions (**1u**,**v**, see Supporting Information).

A variety of differently substituted isoxazoles proved to be compatible with the reaction. 3-Sulfenylated pyrroles can be prepared with alkyl, aryl, and vinyl groups at the 5-position (**3u–3af**), and with aldehydes (**3x**), amides (**3y**,**z**), and aryl- or alkyl-substituted carbonyl groups at the 4-position (**3u**–**w**,**ac**–**af**). No reaction was seen with the electronically deactivated 3-(perfluorophenyl)-5-phenylisoxazole and 3-phenyl-5-(trifluoromethyl)isoxazole (not shown). The use of isoxazol-5-amines had not previously been reported under gold catalysis,^10^ while Liu reported non-pyrrole-forming pathways when unsubstituted isoxazoles were reacted with propiolate derivatives.^4b,4c^ The formation of pyrroles (**3x**–**z**) from both of these systems illustrates the consistent reaction outcomes obtained in gold-catalyzed reactions of alkynyl thioethers with nitrenoids. The reaction tolerates a range of functionalities, including 1° and 3° aromatic amines (**3n**,**y**,**z**), a free hydroxyl group (**3ac**), an ester (**3ad**), and an aryl halide (**3af**).

The wider generality of alkynyl thioethers was established using anthranil **4** as the nitrenoid to deliver 3-sulfenyl-7-acyl indole motifs **5a**–**f** by the formal (3 + 2) cycloaddition pathway. 3-Sulfenyl indoles have shown some medicinal potential with activity against HIV^11^ and inhibition of tubulin polymerization.^12^ As anthranil and the products coeluted, the alkynyl thioether was used in excess (see the Supporting Information). A competition experiment showed that anthranil **4** is less reactive than isoxazole **2a** (see the Supporting Information).

Reactions with 3,4,5-trisubstituted isoxazoles **6a**,**b** were investigated (Scheme 3). Reactions with isoxazoles **6a**,**b** saw formation of the N-acylated pyrrole **7** and deacylative annulation product **9**, respectively, as has been precedented in ynamide reactions.^13^ Reactivity unique to alkynyl thioethers was also elucidated with the formation of desulfenylated 3-acylated pyrroles **8a**,**b**.

**Scheme 3:**
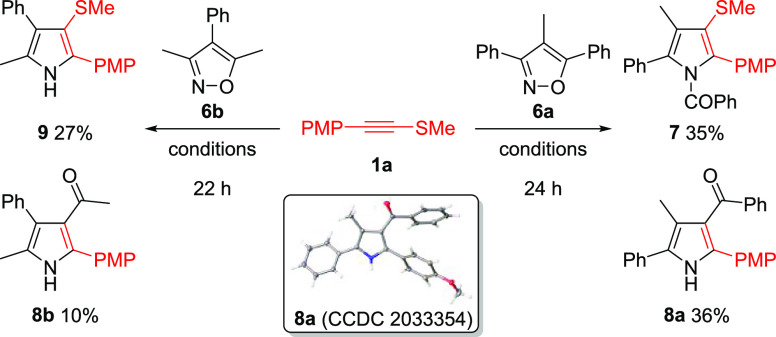
Au(I)-Catalyzed Reactions of Alkynyl Thioethers with 3,4,5-Trisubstituted Isoxazoles All reactions were run in capped vials with no precautions against air or moisture, using **1a** (1.0 equiv), **6a** or **6b** (2.0 equiv), [IPrAu(CH_3_CN)]SbF_6_ (5 mol %), and PhCF_3_ (0.1 M), at 80 °C. The crystal structure of **8a** is shown with ellipsoids drawn at the 50% probability level.

The practicality of the annulation protocol is demonstrated by its ready upscaling, which proceeded smoothly under noninert conditions to give **3a** on a gram scale (Scheme 4). Selective oxidations of the resulting pyrrole **3a** into its sulfonyl and sulfinyl forms (**10** and **11**) were effective.^14^ Treating **11** with triflic anhydride in the presence of base led to protodesulfinylation and the formation of trisubstituted pyrrole **12**. The direct use of triflic acid^15^ gave a dirtier reaction and only traces of **12**. However, Brønsted acid mediated desulfenylation of the sulfide **3d** also gave **12**. Such acid-mediated desulfuration reactions permit chemoselective reduction of the sulfenyl group in the presence of the ketone and enable the nitrenoid chemistry to be used to access non-heteroatom-substituted pyrroles. Initial attempts to achieve metal-catalyzed C–S activation for cross-coupling have so far been unsucessful with these elaborated pyrroles.

**Scheme 4:**
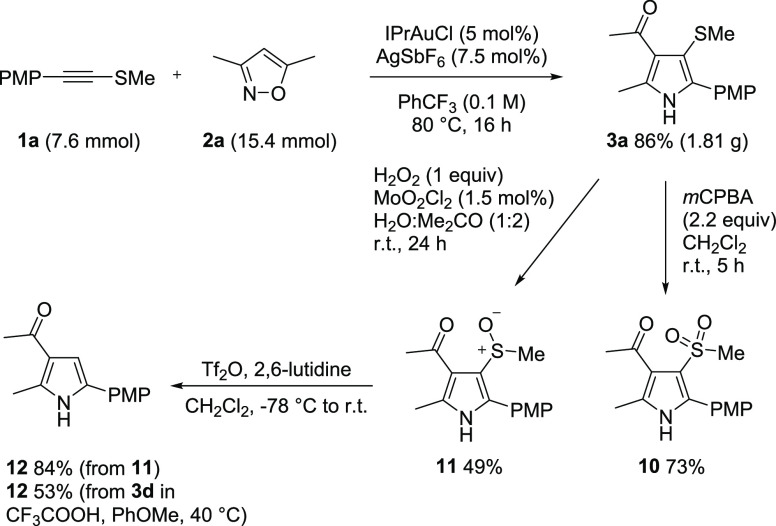
Scale-up Annulation and Selective Oxidation of a 3-Sulfenyl Pyrrole

The isotopically labeled substrate ^**13**^**C-1l** was prepared in order to determine whether there was any skeletal rearrangement of the alkynyl thioether backbone during or after annulation. 1,2-Sulfenyl migrations have been reported in π-acid mediated reactions that invoke carbenoid character,^5a−5d^ while methylthio-substituted pyrroles have been shown to undergo Brønsted acid mediated isomerization.^16^ The single pyrrole isotopomer ^**13**^**C-3l** was formed under the standard conditions. A combination of HMBC and NOESY experiments was used to confirm the regiochemical outcome on **3l**/^**13**^**C-3l** and that the connectivity of the alkynyl thioether was maintained (Scheme 5). A small amount of the enriched β-keto enamide ^**13**^**C-13** was also isolated. This outcome is in concordance with the pathway reported by Ye under Zn(II) catalysis^9^ and provides labeling evidence for the 1,2-S*-*migration step.

**Scheme 5:**
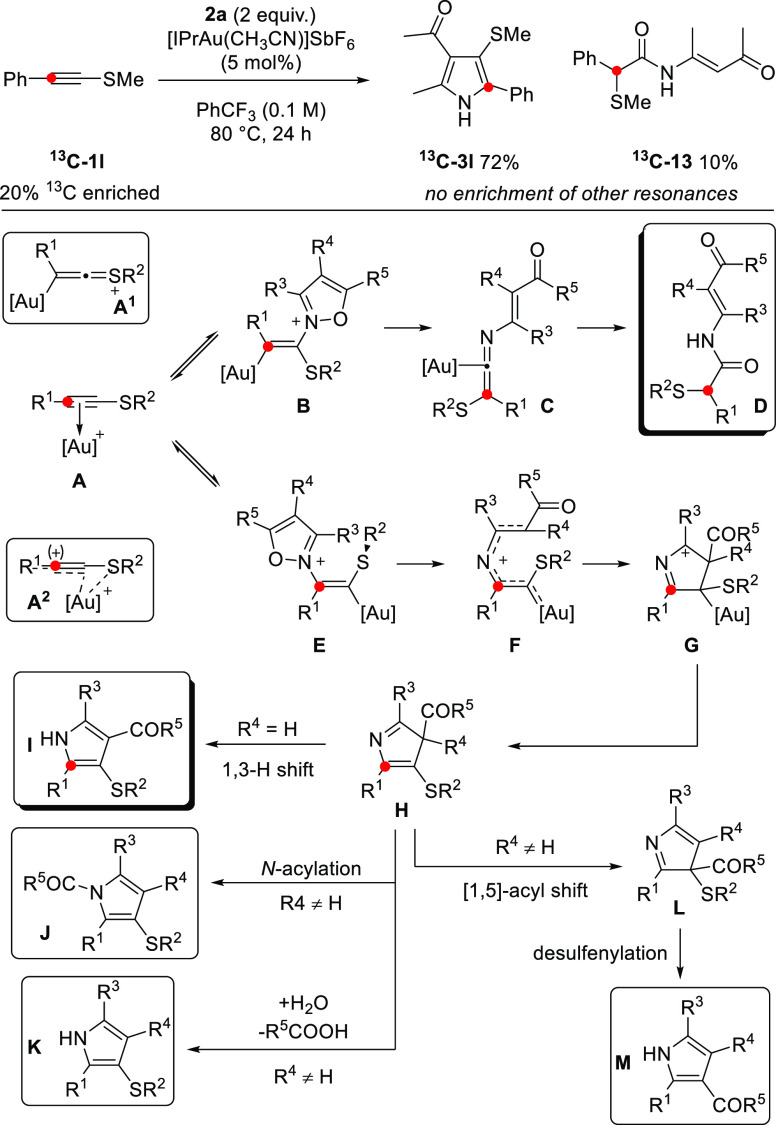
^13^C-Labeling Experiment and Outline Mechanism

A general mechanism is proposed on the basis of these observations and literature precedent (Scheme 5). The ^13^C-labeling experiment is consistent with slippage to either end of the triple bond being energetically accessible: α-addition, matching a gold ketenethionium type activation **A**^**1**^ invoked in the majority of Au(I)-catalyzed reactions of alkynyl thioethers, is the minor pathway and is not viable for the formation of pyrrole. This pathway leads to β-ketoenamide **D**. The β-addition pathway is more productive (**A → E**), and we postulate that a stabilizing S–Au interaction^17^ promotes irreversible gold carbene formation. This pathway is aided by, but not dependent upon, the presence of more electron rich alkyne substituents (Scheme 2), which would further distort the π-complex toward a nascent Au–S interaction **A**^**2**^.^18^ The resulting α-imino α′-sulfenyl gold carbene **F** undergoes cyclization and aromatization to pyrrole **I**. When C-4-substituted isoxazoles are used, in addition to established deacylative mechanisms (**H → J**/**K**),^2h,3c^ an unprecedented desulfenylation can follow 1,5-acyl migration,^4b^ leading to all-carbon substituted pyrroles (**H → M**).

In conclusion, alkynyl thioethers react with isoxazoles and anthranils under gold catalysis and provide selective access to the formal (3 + 2) cycloaddition pathway. This pathway is maintained across reactants with broad structural and functional group changes. As a result, these readily accessed substrates can be used to deliver convergent and modular access to sulfenylated pyrroles and indoles. The practical and straightforward protocol has been demonstrated on a multi-millimole scale. Initial investigations into the annulation with trisubstituted isoxazoles also reveal reactivity unique to alkynyl thioethers. One-step access to all-carbon-substituted pyrroles establishes the intriguing potential of using a sulfenyl moiety as a traceless directing and alkyne-activating group for heterocycle synthesis. The observed outcomes and an isotopic labeling study match a β-selective addition to the alkynyl thioether. In contrast to α-selective approaches, such as from ubiquitously employed ynamides, the gold carbene character is developed adjacent to the heteroatom group and not the other, variable, alkyne substituent. If gold-catalyzed reactions of alkynyl thioethers and nitrenoids consistently proceed through the putative α-imino α′-sulfenyl gold carbene, then this can provide the basis of a unifying (3 + 2) cycloaddition strategy for heterocycle synthesis.

## Supporting Information Available

Experimental details, compound characterization data, crystallographic data, and NMR spectra (PDF)
Crystallographic data for **3j** (CIF)
Crystallographic data for **8a** (CIF)

## Supplementary Material

cs1c01457_si_001.pdf

cs1c01457_si_002.cif

cs1c01457_si_003.cif
