# Supplementary material for: Gold(I)-Catalyzed Synthesis of 3-Sulfenyl Pyrroles and Indoles by a Regioselective Annulation of Alkynyl Thioethers
Source: ACS Catal. 2021 May 13;11(11):6357–62. doi: 10.1021/acscatal.1c01457 (PMC8291588; doi:10.1021/acscatal.1c01457)

SUPPORTING INFORMATION

Gold(I)-Catalyzed Synthesis of 3-Sulfenyl Pyrroles and Indoles by a Regioselective Annulation of Alkynyl Thioethers

Peter E. Simm†, Prakash Sekar†, Jeffery Richardson‡, and Paul W. Davies†*

† School of Chemistry, University of Birmingham, Edgbaston, Birmingham, B15 2TT, UK

‡ Lilly UK, Erl Wood Manor, Windlesham, GU20 6PH, UK

E-mail: p.w.davies@bham.ac.uk

General Experimental 2

Starting Materials 3

Catalysis Reactions 17

Crystallographic data 37

References 39

^{1}H NMR and ^{13}C NMR Spectra 40

General Experimental

All reagents were purchased from commercial sources and used without further purification unless stated otherwise. Unless stated otherwise, reactions were performed under an atmosphere of argon using standard Schlenk techniques. Anhydrous THF, toluene, dichloromethane and acetonitrile were dried and degassed using a Pure-Solv MD solvent purification system; all other anhydrous solvents were dried over 4 Å molecular sieves. TLC analysis was undertaken using Merck silica gel 60 F254 (aluminium support) TLC plates; plates were visualised under UV light (254 nm), or through a vanillin or potassium permanganate stain. Manual flash column chromatography was undertaken using Sigma Aldrich 60 Å silica gel; automated flash column chromatography was undertaken using a Teledyne Isco Combiflash NextGen 100 instrument, using either Teledyne Isco Redisep RediSep® Normal-phase, RediSep Rf Gold® Normal-Phase, or InterChim Puriflash IR Silica flash columns. Mass spectra were obtained using Waters GCT Premier (EI), Waters LCT (ES), Waters Synapt (ES) or Bruker MicroTOF spectrometers, each fitted with TOF detector. For measurements on Waters instruments the calculated and measured values are reported as neutrals within Waters MassLynx (V4.1 used). High resolution spectra used a lock-mass to adjust the calibrated mass scale. IR spectra were recorded on Perkin Elmer spectrum 100 FT-IR spectrometer. NMR spectra were obtained using a Bruker AVIII300, AVIII400, or Avance NEO 400 spectrometer; chemical shifts are stated in ppm, and are measured relative to residual non-deuterated solvent. 1D ^{13}C NMR spectra were recorded using the UDEFT or PENDANT pulse sequences from the Bruker standard pulse program library. HSQC and HMBC spectra were recorded using the Bruker standard pulse program library. For 1H NMR, multiplicities are denoted by the following notation: s (singlet), d (doublet), t (triplet), q (quadruplet), quin (quintuplet), br (broad), m (multiplet), app. (apparent). NMR spectra were processed using either MestReNova 10.0.

Starting Materials

Synthesis of Alkynyl Thioethers

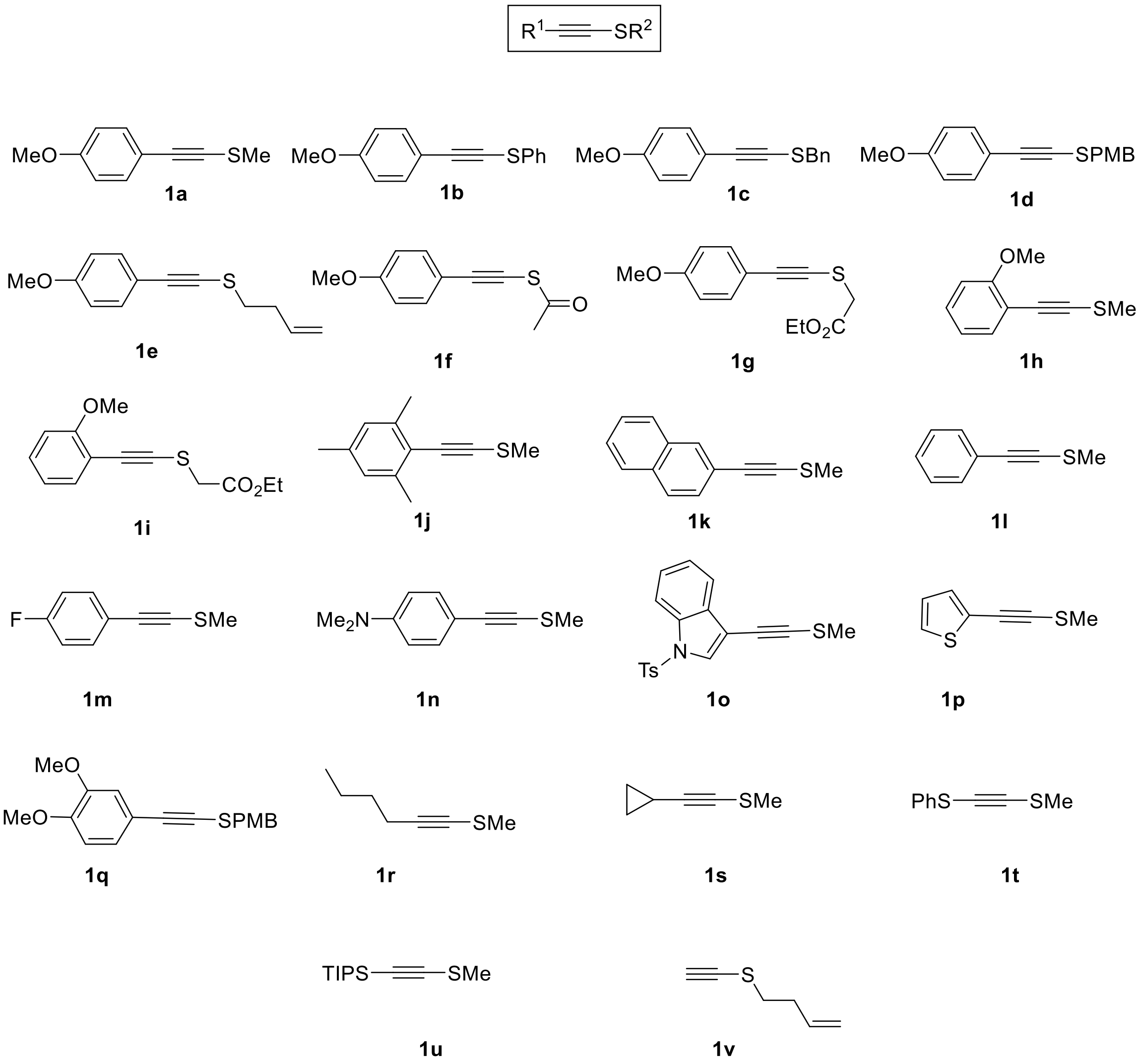

The syntheses and spectroscopic analysis of the following alkynyl thioethers was reported in prior work within the group[1, 2]; **1a, 1b, 1e, 1j, 1l, 1o,** and **1r**. Preparation of the other alkynyl thioethers is described herein.

Synthesis of Precursors

Terminal alkynes **TA1 – 7** (below) were synthesised in accordance to literature precedent. **TA1**[3] (94% over 2 steps), **TA2**[4] (71% yield over two steps), **TA3**[5] (67% yield over 2 steps), **TA4**[6] (80% yield over 2 steps), **TA5**[7] (35% yield over 2 steps), **TA6**[8] (75% yield over 2 steps), **TA7**[9] (67% yield over 2 steps).

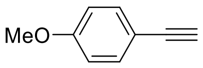

TA1

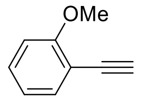

TA2

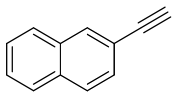

TA3

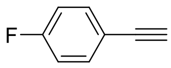

TA4

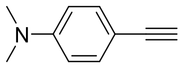

TA5

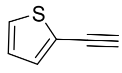

TA6

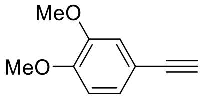

TA7

Sulfonothiates **TS1–4** (below) were synthesised in accordance to literature precedent. **TS1**[10] (95%), **TS2**[10] (93%), **TS3**[11] (51% over 2 steps), **TS4**[11] (52% yield over 2 steps) and **TS5**[12] (63% over 2 steps).

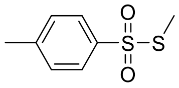

TS1

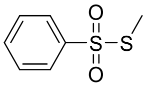

TS2

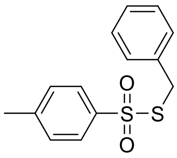

TS3

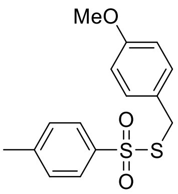

TS4

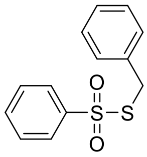

TS5

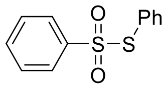

TS6

General procedure for the synthesis of alkynyl thioethers from corresponding lithium acetylides and *S*-aryl/alkyl thiosulfonates (GP1); preparation of 1c, 1d, 1h, 1k, 1m, 1n, 1p, 1q and 1s.

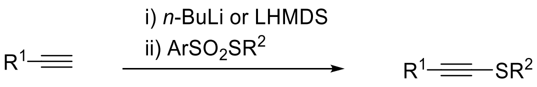

To a dry and degassed 3-necked round bottom flask was charged the terminal alkyne and anhydrous THF (0.2 M). The solution was cooled to –78 °C, and *n*-butyllithium (1.6 M or 2.5 M in hexanes, 1.1 equiv) or LHMDS (1 M in THF, 1.1 equiv) was added dropwise. The reaction was stirred for 1 h at –78 °C, at which point the corresponding *S*-aryl/alkyl thiosulfonate was added dropwise (if a liquid) or in a single portion (if a solid). The reaction was allowed to warm to room temperature and stirred overnight. The reaction was quenched by addition of $NH_4Cl_{(sat.\ aq)}$ and extracted with Et_2O. Ethereal extracts were dried over Na_2SO_4, and the solvent removed *in vacuo*; the resulting residue was chromatographed (SiO_2) to yield alkynyl thioethers **1**.

Benzyl((4-methoxyphenyl)ethynyl)sulfane (1c)

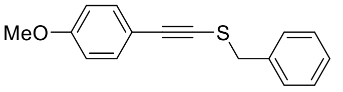

Prepared according to GP1, using 4-ethynyl anisole (**TA1**) (0.201 g, 1.52 mmol), anhydrous THF (10 mL), followed by *n*-BuLi (1.6 M in hexanes, 1.2 mL, 1.818 mmol, 1.2 equiv) and $PhSO_2SBn$ (**TS5**) (0.480 g, 1.818 mmol, 1.2 equiv). Column chromatography (SiO_2, 0 to 5% EtOAc in hexane) afforded **1c** as a yellow oil (0.16 g, 42%). ^{1}H NMR (400 MHz, $CDCl_3$): δ = 7.39–7.29 (m, 7H), 6.82 (d, *J* = 8.8 Hz, 2H), 4.00 (s, 2H), 3.81 (s, 3H); ^{13}C NMR (101 MHz, $CDCl_3$) δ = 159.7, 136.8, 133.4, 129.2, 128.6, 127.8, 115.5, 114.0, 94.5, 77.3, 55.4, 40.6; HRMS (ES+) calculated for $C_{17}H_{19}O_2S$: 255.0844, found 255.0838 [M+H]. This compound is known and the spectroscopic data matches that reported.[13]

((4-Methoxybenzyl)((4-methoxyphenyl)ethynyl)sulfane (1d)

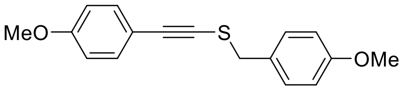

Prepared according to GP1, using 4-ethnylanisole (**TA1**, 0.844 g, 6.83 mmol), anhydrous THF (50 mL), followed by *n*-BuLi (2.5 M in hexanes, 3 mL, 7.5 mmol, 1.1 equiv) and TsSPMB (**TS4**) (2.53 g, 8.21 mmol, 1.2 equiv). Following column chromatography (SiO_2, 1:1 CH_2Cl_2:Hexane), impure **1d** was obtained as an orange oil. This oil was suspended in hot heptane (*ca.* 2 mL) and CH_2Cl_2 was added dropwise until a solution was obtained; the solution was cooled in the freezer, and the resulting precipitate collected to afford **1d** as a pale-red solid (1.38 g, 71%).^{1}H NMR (400 MHz, $CDCl_3$): δ = 7.33–7.29 (m, 4H), 6.88 (d, *J* = 8.7 Hz, 2H), 6.82 (d, *J* = 8.9 Hz, 2H), 3.98 (s, 2H), 3.80(9) (s, 3H), 3.80(5) (s, 3H); ^{13}C NMR (101 MHz, $CDCl_3$) δ 159.7, 159.3, 133.4(1), 130.4(3), 128.9, 115.7, 114.1, 114.0, 94.4, 77.63, 55.4 (2×OCH_3), 40.3; HRMS (ES+); calculated for $C_{17}H_{17}O_2S$: 285.0949, found 285.0942 [M+H]; IR (ATR): ν/ cm^{-1} = 1600, 1505, 1467, 1437, 1292, 1248, 1228, 1172, 1029, 830, 747. This compound is known and the obtained spectroscopic data matches that reported. [14]

(2-Methoxyphenyl)ethynyl)(methyl)sulfane (1h)

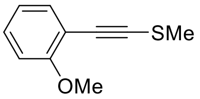

Prepared according to GP1, using 2-ethynylanisole (**TA2**, 1.013 g, 7.66 mmol), anhydrous THF (40 mL), followed by *n*-BuLi (2.5 M in hexanes, 3.4 mL, 8.50 mmol, 1.1 equiv) and $PhSO_2SMe$ (**TS2**) (1.65 g, 8.70 mmol, 1.1 equiv). Column chromatography (SiO_2, 2.5% EtOAc in hexane) afforded **1h** as an orange oil (0.595 g, 44%). ^{1}H NMR ($CDCl_3$, 400 MHz): δ = 7.39 (dd, *J* = 7.7 Hz , 1.7 Hz, 1H), 7.26 (ddd, *J* = 8.9, 7.7 Hz, 1.7 Hz, 1H), 6.91–6.84 (m, 2H), 3.87 (s, 3H), 2.49 (s, 3H); ^{13}C NMR (101 MHz, $CDCl_3$): δ = 160.1, 133.6, 129.6, 120.4, 112.6, 110.6, 87.9, 84.7, 55.8, 19.6. This compound is known and the obtained spectroscopic data matches that reported.[15]

Methyl(naphthalen-2-ylethynyl)sulfane (1k)

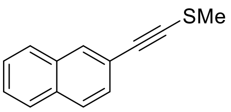

Prepared according GP1, using 2-ethynylnaphthalene (**TA3**, 0.301 g, 1.98 mmol), anhydrous THF (10 mL), followed by *n*-BuLi (2.5 M in hexanes, 0.9 mL, 2.25 mmol, 1.2 equiv) and $PhSO_2SMe$ (**TS2**) (0.476 g, 2.53 mmol, 1.2 equiv). Column chromatography (SiO_2, hexane) afforded **1k** as a yellow solid (0.362 g, 91%). ^{1}H NMR (400

MHz, $CDCl_3$): δ = 7.94 (s, 1H), 7.83–7.74 (m, 3H), 7.51 -7.45 (m, 3H), 2.53 (s, 3H); ^{13}C NMR (101 MHz, $CDCl_3$): δ = 133.0, 132.7, 131.2, 128.3, 127.9, 127.8, 127.7, 126.6, 126.5, 120.7, 92.3, 81.4, 19.5. This compound is known and the obtained spectroscopic data matches that reported.[16]

((4-Fluorophenyl)ethynyl)(methyl)sulfane (1m)

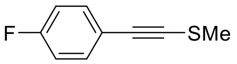

Prepared according to GP1, using 1-ethynyl-4-fluorobenzene (**TA4**, 0.822 g, 6.84 mmol), anhydrous THF (35 mL), followed by LHMDS (1 M in THF, 7.5 mL, 7.50 mmol, 1.1 equiv) and $PhSO_2SMe$ (**TS2**) (1.518 g, 8.33 mmol, 1.2 equiv). Column chromatography (SiO_2, hexane) afforded **1m** as a pale-yellow oil (0.851 g, 75%). ^{1}H NMR (400 MHz, $CDCl_3$): δ = 7.39 (dd, J = 9.0 Hz, $J_{H\text{-}F}$ = 5.2 Hz, 2H), 6.99 (app t, J = 9.0 Hz, 2H), 2.47 (s, 3H); ^{13}C NMR (101 MHz, $CDCl_3$): δ = 162.4 (d, $J_{C\text{-}F}$ = 249.0 Hz), 133.5 (d, $J_{C\text{-}F}$ = 8.5 Hz), 119.5 (d, $J_{C\text{-}F}$ = 3.8 Hz), 115.6 (d, $J_{C\text{-}F}$ = 22.8 Hz), 90.7, 80.6, 19.4. This compound is known and the obtained spectroscopic data matches that reported.[17]

***N,N*-Dimethyl-4-((methylthio)ethynyl)aniline (1n)**

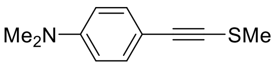

Prepared according to GP1, using 4-ethynyl-*N,N*-dimethylaniline (**TA5**, 0.470 g, 3.24 mmol), anhydrous THF (18 mL), followed by *n*-BuLi (2.5 M in hexanes, 1.4 mL, 3.50 mmol, 1.1 equiv) and $PhSO_2SMe$ (**TS2**) (0.730 g, 4.00 mmol, 1.2 equiv). Column chromatography (5% EtOAc in hexane) afforded **1n** as a yellow solid that turned brown over time (0.506 g, 82%). ^{1}H NMR (400 MHz, $CDCl_3$): δ = 7.33 (d, J = 9.0 Hz, 2H), 6.60 (d, J = 8.9 Hz, 2H), 2.97 (s, 6H), 2.44 (s, 3H); ^{13}C NMR (101 MHz, $CDCl_3$): δ = 150.2, 133.4, 111.7, 110.0, 92.8, 77.5, 40.2, 19.8; HRMS (ES+): calculated for $C_{11}H_{14}NS$: 192.0847, found 192.0845 [M+H]; IR (ATR): ν/ cm^{-1} = 2924, 2150, 1603, 1517, 1441, 1358, 1228, 1183, 1063, 945, 812.

2-((Methylthio)ethynyl)thiophene (1p)

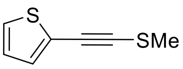

Prepared according to GP1, using 2-ethynylthiophene (**TA6**, 0.841 g, 7.78 mmol), in anhydrous THF (40 mL), followed by *n*-BuLi (2.5 M in hexanes, 3.4 mL, 8.5 mmol, 1.1 equiv) and $PhSO_2SMe$ (**TS2**) (1.690 g, 9.28 mmol, 1.15 equiv). Column chromatography (SiO_2, hexane) afforded **1p** as a pale-yellow oil (0.907 g, 76%). ^{1}H NMR (300 MHz, $CDCl_3$): δ = 7.26 (dd, J = 5.2 Hz, 1.1 Hz, 1H), 7.22 (dd, J = 3.7 Hz, 1.2 Hz, 1H), 6.96 (dd, J = 5.2, 3.6 Hz, 1H), 2.46 (s, 3H); ^{13}C NMR (101 MHz, $CDCl_3$): δ = 133.0, 127.8, 127.0, 123.6, 85.4, 84.6, 19.5; IR (ATR): ν/ cm^{-1} = 2925, 2154, 1419, 1310, 1233, 1172, 1042, 975, 854, 830, 769, 696. This compound is known and the obtained spectroscopic data matches that reported.[18]

((3,4-Dimethoxyphenyl)ethynyl)(4-methoxybenzyl)sulfane (1q)

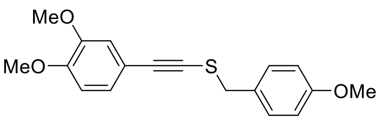

Prepared according to GP1, using 4-ethynyl-1,2-dimethoxybenzene (**TA7**, 0.645 g, 3.98 mmol), anhydrous THF (20 mL), followed by *n*-BuLi (2.5 M in hexanes, 1.8 mL, 4.5 mmol, 1.1 equiv) and TsSMPB (**TS4**) (1.418 g, 4.60 mmol, 1.1 equiv). Column chromatography (SiO_2, 0 to 20% EtOAc in

hexane) afforded **1q** as an off-white solid (0.869 g, 69%). M.P: 83 – 85 °C; ^{1}H NMR (400 MHz, $CDCl_3$): δ = 7.30 (d, *J* = 8.8 Hz, 2H), 6.99 (dd, *J* = 8.3 Hz, ^{4}J = 1.9 Hz, 1H), 6.90–6.85 (m, 3H), 6.78 (d, *J* = 8.3 Hz, 1H), 3.99 (s, 2H), 3.88 (s, 3H), 3.85 (s, 3H), 3.80 (s, 3H); ^{13}C NMR (101 MHz, $CDCl_3$): δ = 159.2, 149.5, 148.5, 130.4, 128.7, 125.1, 115.6, 114.5, 114.0, 113.9, 110.9, 94.4, 55.9, 55.8, 55.3, 40.2; ^{13}C NMR (101 MHz, $CDCl_3$) δ 159.3, 149.6, 148.6, 130.5, 128.8, 125.2, 115.7, 114.6, 114.1, 111.0, 94.6, 77.7, 56.0, 55.9, 55.4, 40.3. HRMS (ES+); calculated for $C_{18}H_{19}O_3S$: 315.1055, found 315.1064 [M+H]; IR (ATR): ν/ cm^{-1} = 1610, 1596, 1575, 1509, 1463, 1439, 1319, 1265, 1247, 1236, 1135, 1020, 848, 840, 813.

(Cyclopropylethynyl)(methyl)sulfane (1s)

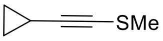

Prepared according to GP1, using ethynylcyclopropane (0.37 mL, 0.333 g, 5 mmol), in anhydrous THF (25 mL), followed by LHMDS (1 M in THF, 5.5 mL, 5.50 mmol, 1.1 equiv) and $PhSO_2SMe$ (**TS2**) (1.07 g, 5.68 mmol, 1.1 equiv). Column chromatography (SiO_2, hexane) afforded **1s** as a pale-yellow oil (0.339 g, 60%). ^{1}H NMR (400 MHz, $CDCl_3$): δ = 2.33 (s, 3H), 1.37–1.29 (m, 1H), 0.81–0.74 (m, 2H), 0.74–0.68 (m, 2H); ^{13}C NMR (101 MHz, $CDCl_3$): δ = 96.3, 65.1, 18.8, 8.2, 0.0. This compound is known and the obtained spectroscopic data matches that reported.[19]

Triisopropyl((methylthio)ethynyl)silane (1u)

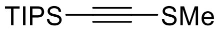

Prepared according to GP1, using (Triisopropylsilyl)acetylene (0.926 g, 5.08 mmol), anhydrous THF (25 mL), followed by *n*-BuLi (1.6 M in hexanes, 3.5 mL, 5.6 mmol, 1.1 equiv) and TsSMe (**TS1**) (1.100 g, 5.60 mmol, 1.1 equiv). Column chromatography (SiO_2, hexane) afforded **1u** as a colourless oil (0.877 g, 76%). ^{1}H NMR (400 MHz, $CDCl_3$): δ = 2.41 (s, 3H), 1.06 (m, 21H); ^{13}C NMR (101 MHz, $CDCl_3$) δ 97.6, 96.0, 19.9, 18.7, 11.4; HRMS (CI+); calculated for $C_{12}H_{24}SSi$: 228.1368, found 228.1374 [M]. IR (ATR): v /cm^{-1} = 2985, 2865, 2091, 1463, 882, 858

General procedure for the synthesis of alkynyl thioethers from corresponding lithium acetylides, elemental sulfur and haloalkanes (GP2); preparation of 1f, 1g and 1i

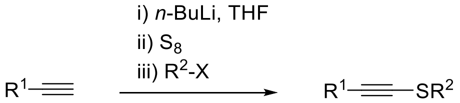

Following the method of Zheng *et al*;[20] to a dry and degassed three-necked round bottom flask was charged terminal alkyne and anhydrous THF (0.2 M). The resulting solution was cooled to –78 °C, and *n*-BuLi (2.5 M in hexanes, 1.1 equiv) was added dropwise. After stirring for 1 h at –78 °C, S_8 (1 equiv atom S) was added, and the reaction mixture was allowed to warm to 0 °C, whereupon it was stirred for 2 hours. The appropriate electrophile (1 equiv) was added dropwise, and the reaction mixture stirred for overnight at room temperature. The reaction was quenched with NH_4Cl $_{(sat.\ aq)}$, and extracted with Et_2O. Ethereal extracts were washed with brine and dried over Na_2SO_4. The solvent was removed *in vacuo*, and the resulting residue chromatographed to yield alkynyl thioethers **1**.

S-((4-methoxyphenyl)ethynyl) ethanethioate (1f)

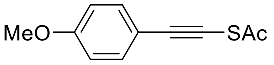

Prepared according to GP2, using 4-ethynylanisole (**TA1**, 1.23 g, 9.32 mmol), anhydrous THF (50 mL), followed by *n*-BuLi (1.6 M in hexanes, 6.4 mL, 10.3 mmol, 1.1 equiv), S_8 (0.327 g, 10.2 mmol S, 1.07 equiv. atom S) and acetyl chloride (0.71 ml, 9.95 mmol, 1.07 equiv). Column chromatography (SiO_2, 10% EtOAc in hexane) afforded **1f** as a yellow solid (808 mg, 43%). M.P: 48 – 50 °C; ^{1}H NMR (400 MHz, $CDCl_3$) δ 7.45 (d, *J* = 8.9 Hz, 2H), 6.86 (d, *J* = 8.9 Hz, 2H), 3.82 (s, 3H), 2.50 (s, 3H). ^{13}C NMR (101 MHz, $CDCl_3$) δ 193.4, 160.5, 133.9, 114.4, 114.2, 103.3, 69.6, 55.5, 29.7. HRMS (ES+) calculated for $C_{11}H_{10}O_2S$: 207.0480, found 207.0476 [M+H]. IR (ATR): ν/ cm^{-1} =2978, 2172, 1720, 1600, 1506, 1460, 1428, 1255, 1171, 1107, 1023, 971, 834, 815.

Ethyl 2-(((4-methoxyphenyl)ethynyl)thio)acetate (1g)

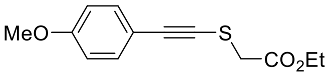

Prepared according to GP2, using 4-ethynylanisole (**TA1**, 0.30 g, 2.27 mmol), anhydrous THF (5 mL), followed by *n*-BuLi (1.6 M in hexanes, 1.6 mL, 2.50 mmol, 1.1 equiv), S_8 (0.0729 g, 2.27 mmol, 1 equiv. atom S) and ethyl bromoacetate (0.28 ml, 2.50 mmol, 1.1 equiv). Column chromatography (SiO_2, 0 to 5% EtOAc in hexane) afforded **1g** as a yellow oil (254 mg, 38%). ^{1}H NMR (400 MHz, $CDCl_3$): δ = 7.37 (d, *J* = 8.9 Hz, 2H), 6.82 (d, *J* = 8.9 Hz, 2H), 4.25 (q, *J* = 7.1 Hz, 2H), 3.81 (s, 3H), 3.54 (s, 2H), 1.30 (t, *J* = 7.1 Hz, 3H); ^{13}C NMR (101 MHz, $CDCl_3$): δ = 168.5, 150.0, 133.7, 115.1, 114.0, 94.4, 75.6, 61.9, 55.4, 37.9, 14.3; HRMS (ES+) calculated for $C_{13}H_{14}O_3SNa$: 273.0561, found 273.0564 [M+Na]. IR (ATR): ν/ cm^{-1} = 2980, 2165, 1732, 1506, 1246, 1170, 1025, 831, 776.

Ethyl 2-(((2-methoxyphenyl)ethynyl)thio)acetate (1i)

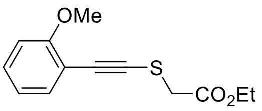

Prepared according to GP2, using 2-ethynylanisole (**TA2**, 0.521 g, 3.94 mmol), anhydrous THF (20 mL), followed by *n*-BuLi (2.5 M in hexanes, 1.75 mL, 4.4 mmol, 1.1 equiv), S_8 (0.129 g, 4.02 mmol S, 1 equiv atom S) and ethyl bromoacetate (0.45 mL, 0.675 g, 4.0 mmol). Column chromatography (SiO_2, 5 to 15% EtOAc in hexane) afforded **1i** as a yellow oil (616 mg, 62%).^{1}H NMR (400 MHz, $CDCl_3$): δ = 7.38 (dd, *J* = 7.6 Hz, 1.8 Hz, 1H), 7.28 (ddd, *J* = 8.2 Hz, 7.4 Hz, 1.7 Hz, 1H), 6.90–6.84 (m, 2H), 4.25 (q, *J* = 7.2 Hz, 2H), 3.86 (s, 3H), 3.60 (s, 2H), 1.30 (t, *J* = 7.2 Hz, 3H); ^{13}C NMR (101 MHz, $CDCl_3$): δ = 168.4, 160.2, 133.8, 130.0 120.4, 112.2, 110.6, 90.7, 81.0, 61.9, 55.8, 38.0, 14.2. IR (ATR): ν/ cm^{-1} = 2981, 2171, 1732, 1594, 1572, 1490, 1434, 1278, 1255, 1114, 1021, 750.

Synthesis of alkynyl disulfide (1t)

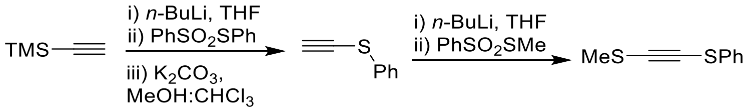

Ethynyl(phenyl)sulfane

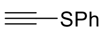

To a dry and degassed 100 mL round bottom flask was charged trimethylsilyl acetylene (1.1 mL, 0.781 g, 7.95 mmol) and anhydrous THF (35 mL). The resulting solution was cooled to – 78 °C, and *n*-BuLi (2.5 M in hexanes, 3.5 mL, 8.75 mmol, 1.1 equiv) was added dropwise. After stirring for an hour at –78 °C $PhSO_2SPh$ (2.33 g, 9.31 mmol, 1.2 equiv) was added dropwise, and the reaction mixture allowed to warm to room temperature and stirred overnight. The reaction was quenched with $NH_4Cl_{(sat.\ aq)}$ (30 mL), and extracted with Et_2O (3 × 30 mL). Ethereal extracts were combined, dried over Na_2SO_4, and concentrated *in vacuo*; the resulting residue was subject to column chromatography (SiO_2, hexane). The resulting TMS protected alkynyl sulfide was dissolved in $CHCl_3$:MeOH (1:1, 30 mL), and K_2CO_3 added (0.567 g, 4.10 mmol, 0.5 equiv). After stirring for 2 h, H_2O (20 mL) was added, and the reaction mixture extracted with $CHCl_3$ (3 × 20 mL); organic extracts were washed with H_2O (30 mL), brine (30 mL), and dried over Na_2SO_4. Removal of the solvent *in vacuo* yielded ethynyl(phenyl)sulfane as a pale-yellow oil (757 mg, 71% over two steps). ^{1}H NMR (300 MHz, $CDCl_3$): δ = 7.46 (d, *J* = 7.5 Hz, 2H), 7.35 (app t, *J* = 7.5 Hz, 2H), 7.29–7.21 (m, 1H), 3.25 (s, 1H).

Methyl((phenylthio)ethynyl)sulfane (1t)

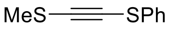

Following GP1 with ethynyl(phenyl) sulfane (0.666 g, 4.96 mmol), anhydrous THF (25 mL), followed by *n*-BuLi (2.5 M in hexanes, 2.2 mL, 5.50 mmol, 1.1 equiv) and TsSMe (1.16 g, 5.73 mmol, 1.2 equiv). Column chromatography (SiO_2, hexane) afforded **1s** as a pale-red oil (0.393 g, 44%). ^{1}H NMR (400 MHz, $CDCl_3$): δ = 7.44–7.41 (m, 2H), 7.35 (app t, *J* = 8.0 Hz, 7.5 Hz, 2H), 7.23 (tt, *J* = 7.4 Hz, 1.2 Hz, 1H), 2.44 (s, 3H); ^{13}C NMR (101 MHz, $CDCl_3$): δ = 133.9, 129.2, 126.7, 125.9, 93.7, 80.8, 20.4; IR (ATR): ν/ cm^{-1} = 1581, 1477, 1440, 1309, 1023, 974, 900, 735, 686. This compound is known and the obtained analytical data matches that reported.[21]

But-3-en-1-yl(ethynyl)sulfane (1v)

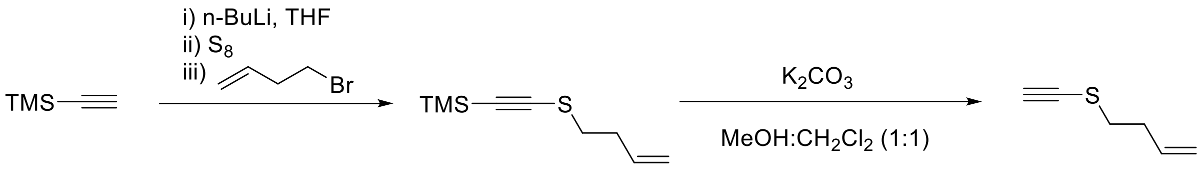

1v

To a dry and degassed 100 mL round bottom flask was charged trimethylsilyl acetylene (0.71 mL, 0.489 g, 5 mmol) and anhydrous THF (25 mL). The resulting solution was cooled to – 78 °C, and *n*-BuLi (1.6 M in hexanes, 3.4 mL, 5.5 mmol, 1.1 equiv) was added dropwise. After stirring for an hour at –78 °C, S_8 (0.160 g, 5 mmol, 1. equiv) was added and the reaction mixture allowed to warm to 0 °C, whereupon it was stirred for two hours. 4-bromobut-1-ene (0.42 mL 560 mg, 1 eq) was added dropwise and the reaction stirred for a further two hours at room temperature., The reaction was quenched with

$NH_4Cl_{(sat.\ aq)}$ (30 mL), and extracted with Et_2O (3 × 30 mL). Ethereal extracts were combined, dried over Na_2SO_4, and concentrated *in vacuo*; the resulting residue was subject to column chromatography (SiO_2, hexane). The resulting TMS protected alkynyl sulfide was dissolved in $CHCl_3$:MeOH (1:1, 20 mL), and K_2CO_3 added (0.230 g, 1.67 mmol, 0.3 equiv). After stirring for 2 h, H_2O (20 mL) was added, and the reaction mixture extracted with $CHCl_3$ (3 × 20 mL); organic extracts were washed with H_2O (30 mL), brine (30 mL), and dried over Na_2SO_4. Removal of the solvent *in vacuo* yielded **1v** as a brown liquid (90.7 mg, 16% over two steps). ^{1}H NMR (400 MHz, $CDCl_3$): δ = 5.77 – 5.88 (m, 1H), 5.07 – 5.17 (m, 2H), 2.76 – 2.82 (m, 3H), 2.49 – 2.53 (m, 2H); ^{13}C NMR (101 MHz, $CDCl_3$) δ 135.6, 117.1, 82.4, 74.4, 34.3, 33.4; HRMS (CI+); calculated for C_6H_7S: 111.0268, found 111.0270 [M-H]; IR (ATR): ν /cm^{-1} = 2925, 1728, 1640, 1436, 1249, 993, 917

Synthesis of isotopically enriched ^{13}C-3l

^{13}C enriched **3l** was synthesised from the correspondingly isotopically enriched dibromoolefin, as below;

Methyl(phenylethynyl-2-^{13}*C*)sulfane

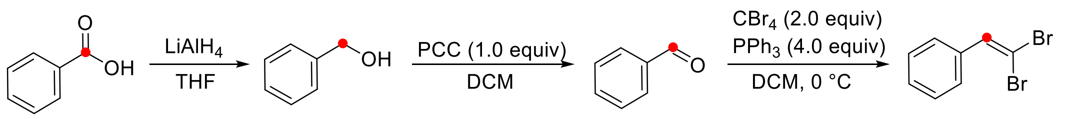

$LiAlH_4$ (3.08 g, 81.2 mmol) was slowly added to a suspension of ^{13}C-enriched benzoic acid (4.96 g, 40.6 mmol, ^{13}C:^{12}C 1:4) in THF (100 mL) at 0 °C and then the reaction mixture was stirred at room temperature for 5 h. After cooling to 0 °C, ethyl acetate was cautiously added followed by methanol to quench the reaction. The reaction mixture was filtered through celite and dried (Na_2SO_4), and concentrated under reduced pressure to give ^{13}C-enriched benzyl alcohol which was further used without purification. Pyridinium chlorochromate (5.98 g, 27.8 mmol) was slowly added to a solution of the crude ^{13}C-enriched benzyl alcohol (3.00 g, 27.8 mmol) in CH_2Cl_2 (100 mL). The reaction mixture was stirred at room temperature for 5 h and the reaction mixture was filtered through celite, dried (Na_2SO_4), and concentrated under reduced pressure to give crude ^{13}C-enriched benzaldehyde (1.80 g). Crude ^{13}C-enriched benzaldehyde (1.80 g, 18.0 mmol, 1.0 equiv) was dissolved in CH_2Cl_2 (100 mL) at 0 °C, followed by addition of CBr_4 (11.26 g, 34.0 mmol, 2.0 equiv) and PPh_3 (17.8 g, 67.9 mmol, 4.0 equiv). The resulting solution was stirred for 2 hours at 0 °C. The solvent was removed under reduced pressure and the residue was purified by column chromatography (SiO_2, 5% EtOAc in hexane) to give the labelled (2,2-dibromo-1-vinyl-1-^{13}C)benzene (2.2 g, 50%) as a yellow oil. ^{1}H NMR (400 MHz, $CDCl_3$): δ = 7.55–7.52 (m, 2H), 7.49 (s, 0.8H), 7.48 (d, *J* = 160.0 Hz, 0.2H), 7.40–7.31 (m, 3H); ^{13}C NMR (101 MHz, $CDCl_3$): δ = 136.8 (labelled carbon), 135.3, 128.5, 128.4(3), 128.4(0), 89.6.

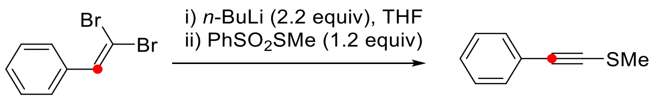

Following GP1 with (2,2-dibromo-1-vinyl-1-^{13}C)benzene (1.00 g, 3.80 mmol), anhydrous Et_2O (15 mL), followed by *n*-BuLi (1.6 M in hexanes, 5.2 mL, 8.36 mmol, 2.2 equiv) and $PhSO_2SMe$ (0.86 g, 4.56 mmol, 1.2 equiv). Column chromatography (SiO_2, hexane) afforded labelled methyl(phenylethynyl)sulfane **^{13}C-1l** as a yellow oil (0.21 g, 37%). ^{1}H NMR (400 MHz, $CDCl_3$): δ = 7.46–7.37 (m, 2H), 7.33–7.27 (m, 3H), 2.48 (s, 3H); ^{13}C NMR (101 MHz, $CDCl_3$) δ = 131.5, 128.4, 128.1, 123.5, 91.9, 81.0, 19.5.

Synthesis of isoxazoles 2a – n

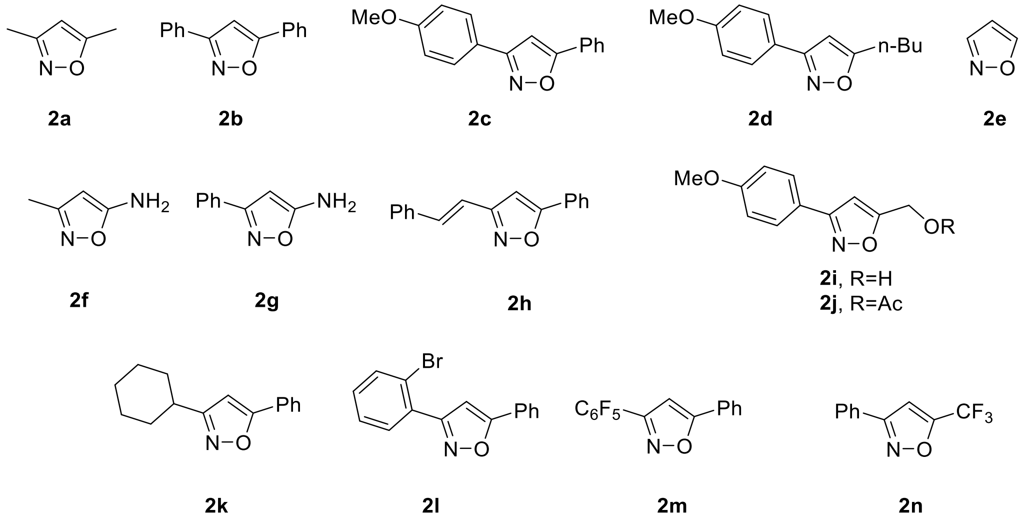

Isoxazoles **2a**, **2e**, **2f**, and **2g** are available commercially.

3,5-Diphenylisoxazole (2b)

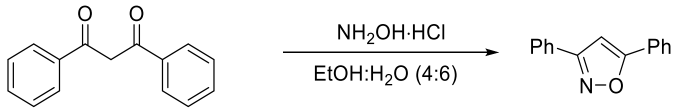

Following literature precedent[22]: A mixture of dibenzoylmethane (1.02 g, 4.53 mmol) and hydroxylamine hydrochloride (0.401 g, 5.77 mmol, 1.25 equiv) in EtOH:H_2O (3:2, 12.5 mL) was heated at reflux overnight. After cooling to room temperature, the resulting precipitate was collected and washed with cold ethanol, affording **2g** as white crystals (0.796 g, 79%). This compound is known and the obtained analytical data corresponds to that reported.

Isoxazoles **2c**, **2d**, **2h**, and **2j** were made through the Cu (I) catalyzed cycloaddition between nitrile oxides and alkynes according to the protocol of Fokin[23]:

3-(4-Methoxyphenyl)-5-phenylisoxazole (2c)

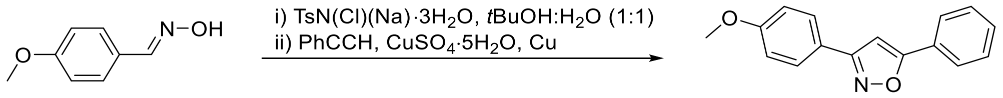

p-Anisaldehyde oxime (0.783 g, 5.17 mmol) was dissolved in *t*BuOH:H_2O (1:1) (40 mL), and chloramine T-trihydrate (1.54 g, 5.46 mmol, 1.05 equiv) was added portionwise over 5 minutes, and the reaction stirred for a further 10 minutes. $CuSO_4·5H_2O$ (39 mg, 0.15 mmol, 3%), Cu (14 mg, 0.22 mmol, 4.3%), and phenylacetylene (0.6 mL, 0.558 g, 5.46 mmol, 1.05 equiv) were then added in that order, and the reaction mixture basified to *ca*. pH 7 by addition of a few drops of NaOH (2.5 M aq. soln). After stirring overnight, the reaction mixture was poured into ice water (50 mL), and the resulting precipitate collected, redissolved in EtOAc, and filtered through a pad of silica (eluting with EtOAc). Removal of the solvent *in vacuo* yielded crude **2c**, with tosyl sulfonamide a significant impurity; recrystallization from hot ethanol afforded **2c** as an off-white solid (0.469 g, 36%). ^{1}H NMR (400 MHz, $CDCl_3$): δ = 7.86–7.79 (m, 4H), 7.52–7.42 (m, 3H), 7.00 (d, *J* = 8.9 Hz, 2H), 6.79 (s, 1H), 3.87 (s, 3H); ^{13}C NMR (101 MHz, $CDCl_3$): δ = 170.2, 162.6, 161.0, 130.2, 129.0, 128.2, 127.6, 125.8, 121.7, 114.3, 97.3, 55.4. This compound is known and the obtained analytical data matches that reported.[24]

5-Butyl-3-(4-methoxyphenyl)isoxazole (2d)

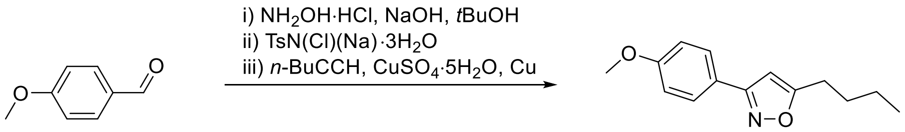

To a mixture of *p*-anisaldehyde (1.38 g, 10.1 mmol) in *t*BuOH:H_2O (1:1), was added $NH_2OH·HCl$ (0.734 g, 10.5 mmol, 1.05 equiv) and NaOH (3 M aq soln.) (3.5 mL, 10.5 mmol, 1.05 equiv). Stirring for 3 hours was followed by addition of chloramine T-trihydrate (3.00 g, 10.6 mmol, 1.06 equiv), $CuSO_4·5H_2O$ (75.8 mg, 0.303 mmol, 3%), Cu dust (31 mg, 0.5 mmol, 5%) and 1-hexyne (1.20 mL, 0.858 g, 10.4 mmol, 1.04 equiv). The reaction mixture was poured into ice water (*ca* 100 mL), and extracted with EtOAc (4 × 50 mL). Combined organic extracts were washed with dilute NH_4OH solution (30 mL), followed by filtration through a pad of silica (eluting with EtOAc). Organic extracts were dried over Na_2SO_4, and the solvent removed *in vacuo*; the resulting residue was subject to column chromatography (5% EtOAc in hexane) to afford **2d** as a pale-yellow oil that slowly crystallised on standing (230 mg, 10%). ^{1}H NMR (400 MHz, $CDCl_3$): δ = 7.72 (d, *J* = 8.9 Hz, 2H), 6.96 (d, *J* = 8.9 Hz, 2H), 6.22 (s, 1H), 3.84 (s, 3H), 2.77 (t, *J* = 7.8 Hz, 2H), 1.72 (app quint, *J* = 8.2 Hz, 2H), 1.42 (app sextet, *J* = 7.6 Hz, 2H), 0.95 (t, *J* = 7.6 Hz, 3H); ^{13}C NMR (101 MHz, $CDCl_3$): δ = 174.0, 161.9, 160.8, 128.1, 128.0, 122.0, 114.2, 98.5, 55.3, 29.6, 26.5, 22.2, 13.7. This compound is known and the obtained analytical data matches that reported.[25]

(*E*)-5-Phenyl-3-styrylisoxazole (2h)

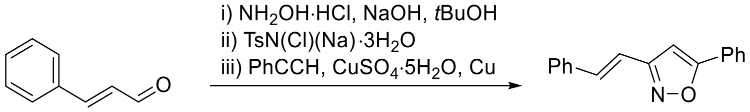

To a mixture of *trans*-cinnamaldehyde (2.50 mL, 2.63 g. 19.9 mmol) in ^{t}BuOH:H_2O (1:1, 80 mL) was added NH_2OH·HCl (1.467 g, 21.0 mmol, 1.05 equiv) and NaOH (1.02 g, 25.5 mmol, 1.2 equiv). The reaction mixture was stirred for 6 hours at room temperature, at which point TLC analysis showed oxime formation to be near complete. Chloramine T-trihydrate (5.98 g, 21.2 mmol, 1.05 equiv) was added portion wise over 5 minutes; after stirring for 10 minutes, $CuSO_4·5H_2O$ (0.17 g, 0.7 mmol, 3.5%), Cu dust (0.050 g, 7%), and phenyl acetylene (2.30 mL, 2.14 g, 21 mmol, 1.05 equiv) were added in that order. The reaction was basified to *ca*. pH 7 by addition of NaOH (2.5 M), and subsequently stirred overnight. The reaction mixture was poured into ice water (*ca* 100 mL), and extracted with EtOAc (4 × 50 mL). Combined organic extracts were washed with dilute NH_4OH solution (30 mL), followed by filtration through a pad of silica (eluting with EtOAc). Organic extracts were dried over Na_2SO_4, and the solvent removed *in vacuo*; the resulting residue was subject to column chromatography (5% EtOAc in hexane). Following recrystallization of mixed fractions, **2h** was obtained as a white solid (1.082 g, 22%). ^{1}H NMR (400 MHz, $CDCl_3$): δ = 7.81 (dd, *J* = 8.0 Hz, 1.8 Hz, 2H), 7.54 (d, *J* = 8.0 Hz, 2H), 7.50–7.43 (m, 3H), 7.39 (app t, *J* = 7.5 Hz, 7.2 Hz, 2H), 7.35–7.31 (m, 1H), 7.24 (d, *J* = 16.6 Hz, 1H), 7.16 (d, *J* = 16.6 Hz, 1H), 6.76 (s, 1H); ^{13}C NMR (101 MHz, $CDCl_3$): δ = 169.8, 162.3, 135.8(9), 135.8(5), 130.2, 129.1, 129.0, 128.9, 128.8, 127.4, 127.0, 125.9, 116.2, 96.5. This compound is known and the obtained analytical data matches that reported.[26]

(3-(4-Methoxyphenyl)isoxazol-5-yl)methanol (2i) and (3-(4-Methoxyphenyl)isoxazol-5-yl)methyl acetate (2j)

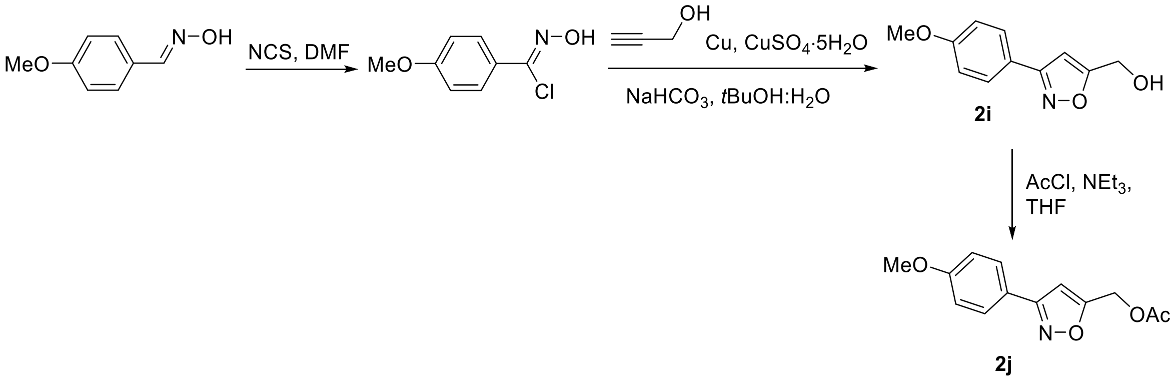

Prepared according to the method of Bernardes et al;[27] to a solution of *p*-anisaldehyde oxime (0.906 g, 6.00 mmol) in DMF (16 mL) was added *N*-chlorosuccinimide (0.887 g, 6.64 mmol, 1.1 equiv) portion wise over 15 minutes. After reaction for 3 hours, Et_2O (25 mL) was added, and the reaction mixture washed with brine (3 × 25 mL). The ethereal extract was dried (Na_2SO_4), followed by the removal of

solvent in vacuo to yield the crude hydroxomyl chloride as a pale-yellow oil, which was used immediately in the next step assuming a 100% yield.

The previously obtained hydroxomyl chloride was dissolved in *t*BuOH:H_2O (1:1, 10 mL), and propargyl alcohol (0.36 mL, 6.24 mmol, 1 equiv), Cu dust (20 mg, 0.046 mmol, 0.8%), $CuSO_4 \cdot 5H_2O$ (48 mg, 0.2 mmol, 3%) and $NaHCO_3$ (1.61 g, 19.2 mmol, 3.2 equiv) were added in that order. After stirring overnight, the reaction was extracted with EtOAc (2 × 20 mL); organic extracts were washed with H_2O (2 × 20 mL) and dried over Na_2SO_4. Removal of the solvent *in vacuo*, followed by chromatography of the resulting residue (20% EtOAc in hexane) afforded **2i** as an off-white solid (0.677 g, 55%) with analytical data matching that reported.

2j was obtained through acylation of **2i**. Under an atmosphere of Ar, **2i** (155.2 mg, 0.75 mmol) was dissolved in anhydrous THF (20 mL). NEt_3 (0.32 mL, 232 mg, 2.29 mmol) was added, followed by Ac_2O (0.22 mL, 239 mg, 2.24 mmol), and the reaction stirred overnight. The reaction was diluted with H_2O and extracted with Et_2O (3 × 20 mL); ethereal extracts were dried over Na_2SO_4, the solvent removed *in vacuo*, and the residue subject to column chromatography (SiO_2, 0 to 30% EtOAc in hexane) to afford **2j** as a foam (66.8 mg, 36%) with analytical data matching that reported.[28]

3-Cyclohexyl-5-phenylisoxazole (2k)

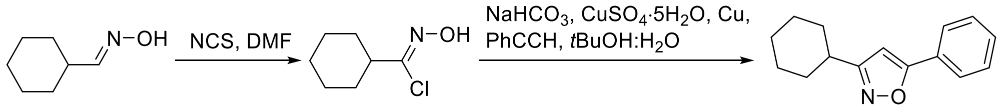

2k

To a solution of cyclohexanecarbaldehyde oxime (1.876 g, 10.5 mmol) in DMF (32 mL) was added *N*-chlorosuccinimide (1.53 g, 11.5 mmol, 1.1 equiv) portion wise over 15 minutes. After reaction for 3 hours, Et_2O (40 mL) was added, and the reaction mixture washed with brine (3 × 40 mL). The ethereal extract was dried (Na_2SO_4), followed by the removal of solvent in vacuo to yield the crude hydroxomyl chloride which was used immediately in the next step assuming a 100% yield.

The previously obtained hydroxomyl chloride was dissolved in *t*BuOH:H_2O (1:1, 32 mL), and phenyl acetylene (1.30 mL, 11.8 mmol, 1.1 equiv), Cu dust (37 mg, 0.59 mmol, 0.6%), $CuSO_4 \cdot 5H_2O$ (90 mg, 0.36 mmol, 3.4%) and $NaHCO_3$ (3.15 g, 37.5 mmol, 3.2 equiv) were added in that order. After stirring overnight, the reaction was extracted with EtOAc (2 × 50 mL); organic extracts were washed with H_2O (2 × 50 mL) and dried over Na_2SO_4. Removal of the solvent *in vacuo*, followed by chromatography of the resulting residue (hexane) afforded **2k** as an off-white solid (1.215 g, 51%).^{1}H NMR ($CDCl_3$, 400 MHz): 7.78–7.73 (m, 2H), 7.46–7.37 (m, 3H), 6.38 (s, 1H), 2.79 (tt, *J*= 10.5 Hz, 3.8 Hz, 1H), 1.58–1.23 (m, 6H), 2.06–1.70 (m, 5H); ^{13}C NMR (101 MHz, $CDCl_3$) δ 169.4, 169.1, 130.0, 129.0, 127.9, 125.8, 97.9, 36.1, 32.2, 26.1, 26.0. This compound is known and the obtained analytical data matches that reported.[23]

3-(2-Bromophenyl)-5-phenylisoxazole (2l)

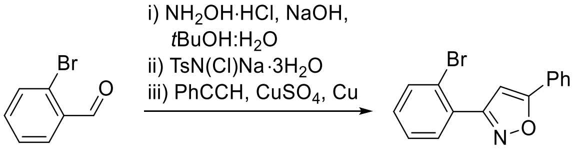

To a mixture of 4-bromobenzaldehyde (2.35 mL, 3.72 g, 20.1 mmol) in *t*BuOH:H_2O (1:1, 80 mL) was added $NH_2OH·HCl$ (1.47 g, 21.0 mmol, 1.05 equiv) and NaOH (0.897 g, 21.0 mmol, 1.1 equiv). The reaction mixture was stirred for 6 hours at room temperature, at which point TLC analysis showed oxime formation to be near complete. Chloramine T-trihydrate (6.10 g, 21.7 mmol, 1.05 equiv) was added portion wise over 5 minutes; after stirring for 10 minutes, $CuSO_4·5H_2O$ (0.157 g, 0.63 mmol, 3%), Cu dust (0.048 g, 0.075 mmol, 0.4%), and phenyl acetylene (2.3 mL, 2.14 g, 21 mmol, 1.05 equiv) were added in that order. The reaction was basified to *ca*. pH 7 by addition of NaOH (2.5 M), and subsequently stirred overnight. The reaction mixture was poured into ice water (*ca* 100 mL), and extracted with EtOAc (4 × 50 mL). Combined organic extracts were washed with dilute NH_4OH solution (30 mL), followed by filtration through a pad of silica (eluting with EtOAc). Organic extracts were dried over Na_2SO_4, and the solvent removed *in vacuo*; the resulting residue was subject to column chromatography (5% EtOAc in hexane). Following recrystallization of mixed fractions, **2l** was obtained as a crystalline white solid (1.92 g, 32%). ^1H NMR ($CDCl_3$, 300 MHz): δ = 7.87–7.82 (m, 2H), 7.72–7.67 (m, 2H), 7.52–7.38 (m, 5H), 7.32 (td, *J* = 7.8 Hz, 1.8 Hz, 1H), 6.95 (s, 1H). ^{13}C NMR (101 MHz, $CDCl_3$) δ 169.7, 163.1, 133.7, 131.5, 131.1, 130.7, 130.4, 129.1, 127.8, 127.5, 126.0, 122.4, 101.0. This compound is known and the obtained analytical data matches that reported.[23]

3-(Perfluorophenyl)-5-phenylisoxazole (2m)

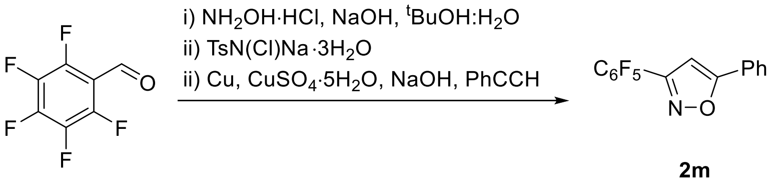

2m

Synthesised in 24% yield following literature precedent.[23]

3-Phenyl-5-(trifluoromethyl)isoxazole (2n)

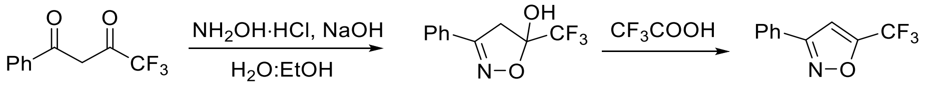

2n

Synthesised in 65% yield over two steps following literature precedent.[29]

4-Methyl-3,5-diphenylisoxazole (6a)

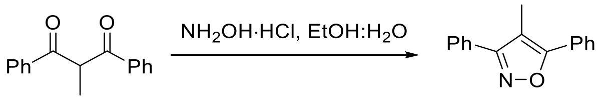

Following literature precedent[22]; a mixture of 2-methyl-1,3-diphenylpropane-1,3-dione (1.01 g, 4.20 mmol) and $NH_2OH{\cdot}HCl$ (378 mg, 5.44 mmol, 1.2 equiv) in EtOH:H_2O (1:1, 12 mL) was heated at reflux overnight. After cooling to room temperature, the precipitate was collected and washed with ice cold ethanol, affording **6a** as a white solid (895.7 mg, 91%) with analytical data matching that reported.

3,5-Dimethyl-4-phenylisoxazole (6b)

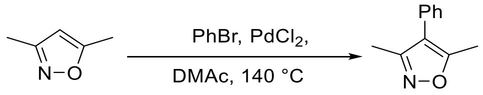

Following literature precedent[30]; To a dry and degassed round bottom flask was charged 3,5-dimethylisoxazole (1.50 mL, 1.49 g, 15.3 mmol), DMAc (40 mL), PhBr (1.10 mL, 1.65 g, 10.5 mmol) and $PdCl_2$ **(**18.8 mg, 0.106 mmol, 1%); the reaction was heated at 140 °C for 48 hours. After cooling to room temperature, the reaction was filtered through celite (eluting with 1:1 Et_2O:hexane); H_2O (100 mL) was added, and the layers separated. The aqueous layer was further extracted with Et_2O:hexane (50 mL); combined organic extracts were washed with water (5 × 50 mL) and brine (50 mL) and dried (Na_2SO_4). Evaporation of the solvent and column chromatography of the residue (0 to 20% EtOAc in hexane) afforded **6b** as a clear oil (659 mg, 36%), with analytical data matching that reported.

Catalysis Reactions

Study of Reaction Conditions

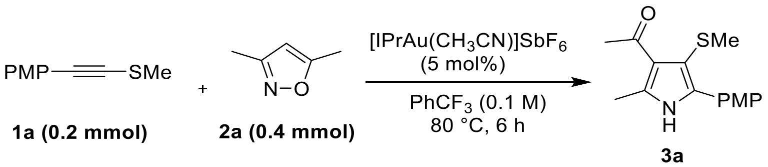

entry	variation from standard conditions	yield (%)[a]
1	No change	92[b]
2[c]	IPrAuCl/$AgNTf_2$ as catalyst	70
3[c]	IPrAuCl/$AgBF_4$ as catalyst	73
4[c]	IPrAuCl/AgOTf as catalyst	68
5[c]	IPrAuCl/NaBARF as catalyst	93
6[c]	IPrAuCl/$AgSbF_6$ as catalyst	91
7[c]	JohnPhosAuCl/$AgSbF_6$ as catalyst	74
8[c]	$Ph_3PAuCl/AgSbF_6$ as catalyst	22
9[c]	DTBPAuCl/$AgSbF_6$ as catalyst	53
10	$PicAuCl_2$ as catalyst	50
11	CH_3NO_2 instead of $PhCF_3$	68
12	$PhCH_3$ instead of $PhCF_3$	72
13	60 °C instead of 80 °C	83
14	1.5 equiv of **2a** instead of 2.0 equiv	80
15	No catalyst	0
16	$HNTf_2$ instead of $[IPrAu(CH_3CN)]SbF_6$	0

[a] Determined by ^1H NMR spectroscopy relative to an internal standard of 3,5-dinitrobenzoate. [b] Isolated yield. BARF = $[B(3,5-(CF_3)_2C_6H_3)_4]$. PMP =*p*-methoxyphenyl. [c] Gold precatalyst and silver cocatalyst used in a 1:1 ratio.

Study of Reaction Conditions for the reaction between 1l and anthranil (4)

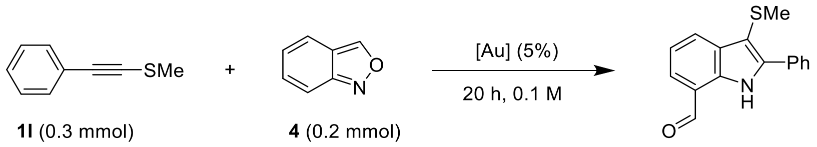

entry	[Au]	Temp/ °C	Solvent	yield (%)[a]
1	IPrAuCl/$AgNTf_2$	r.t	$PhCF_3$	3
2	IPrAuCl/$AgSbF_6$	r.t	$PhCF_3$	4
3	IPrAuCl/$AgNTf_2$	65	$PhCF_3$	31
4	IPrAuCl/$AgSbF_6$	65	$PhCF_3$	31
5	$IPrAuNTf_2$	65	$PhCF_3$	30
6	[IPrAuNCMe]SbF_6	65	$PhCF_3$	28
7	IAdAuCl/$AgNTf_2$	65	$PhCF_3$	Trace
8	JohnPhosAuCl/$AgNTf_2$	65	$PhCF_3$	Trace
9	$IPrAuNTf_2$/$AgNTf_2$ (10 %)	65	$PhCF_3$	33
10	$AuBr_3$	65	$PhCF_3$	0
11	DTBPAuCl/$AgNTf_2$	65	$PhCF_3$	3
12	DMSAuCl	65	$PhCF_3$	Trace
13[b]	$IPrAuNTf_2$	65	$PhCF_3$	17
14	IMesAuCl/$AgNTf_2$	65	$PhCF_3$	Trace
15	SIPrAuCl/$AgNTf_2$	65	$PhCF_3$	10
16	IPrAuCl/$AgNTf_2$	65	1,4-dioxane	20
17	IPrAuCl/$AgNTf_2$	65	$MeNO_2$	24
18	IPrAuCl/$AgNTf_2$	65	PhCl	30
19	IPrAuCl/$AgNTf_2$	65	CPME	Trace
20	IPrAuCl/$AgNTf_2$	65	$PhCF_3$	18

[a] *Determined by ^{1}H NMR spectroscopy relative to an internal standard of 1,2,4,5-tetramethylbenzene.* [b] *4 h reaction.* [c] *0.2 mmol of **1l** used to 0.3 mmol of **4**.*

Comparison between $[B(3,5\text{-}(CF_3)_2C_6H_3)_4]^-$ and SbF_6^- counterions

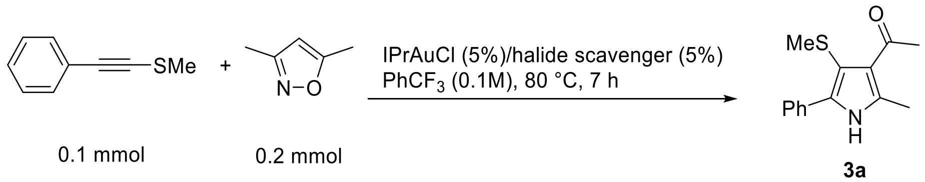

3a

Halide scavenger = NaBARF, 53%
$AgSbF_6$, 54%

Determination of regioisomerism by NMR spectroscopic analysis of 3l and ^{13}C-3l

- Pyrrole N-H (**H1**) is a characteristically distinct broad 1H resonance at 8.59 ppm; exhibits NOESY correlation to 2H doublet at 7.65 ppm.
- Resonance at 7.65 ppm exhibits HMBC correlation to ^{13}C label; can be identified as the *ortho* phenyl protons (**H5**).
- **H5** correlates by NOESY to one of three methyl resonances, that at 2.21 ppm; this can be identified as SMe resonance (**H4**).
- **H4** correlates by NOESY to methyl resonance at 2.75 ppm. Said methyl resonance exhibits HMBC correlation to the distinctive carbonyl resonance at 196.9 ppm. Can be identified as the methyl resonance of the methyl ketone (**H3).**
- **H1** also exhibits NOESY correlation to methyl resonance at 2.55 ppm, which can be assigned as the methyl directly attached to the pyrrole core (**H2**). HMBC analysis supports this assignment of **H2** relative to **H4; H2** resonance exhibits correlations to two carbon resonances of the pyrrole core, in contrast to **H4** which exhibits one, reflecting the more remote connectivity in the latter case. This assignment is further supported by ^{1}H NMR analysis of **3a**, and desulfenylated structural congener **12**; upon desulfenylation the most upfield methyl resonance (2.17 ppm) is no longer observed, whilst resonances at 2.74 (analogous to **H3**) and 2.60 (analogous to **H2**) are shifted upfield slightly.

3l 1H-1H NOESY (400 MHz, $CDCl_3$)

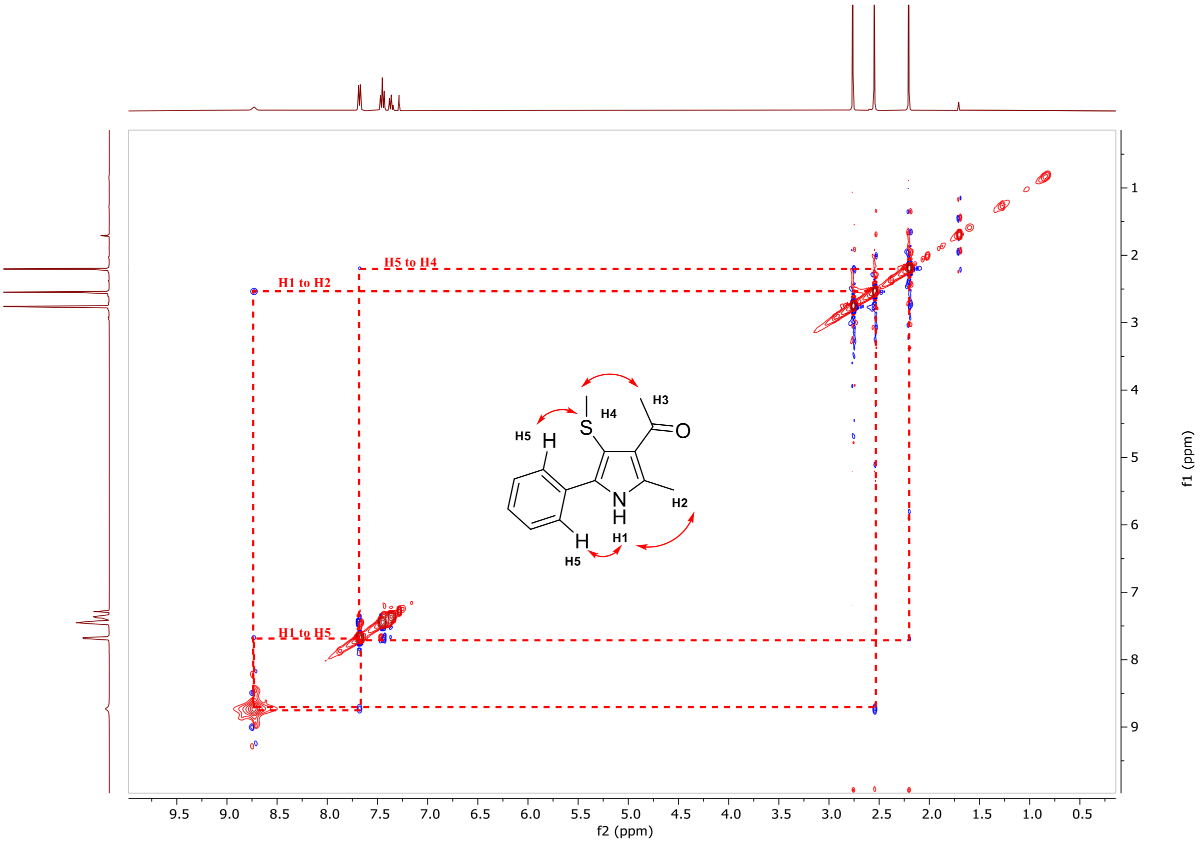

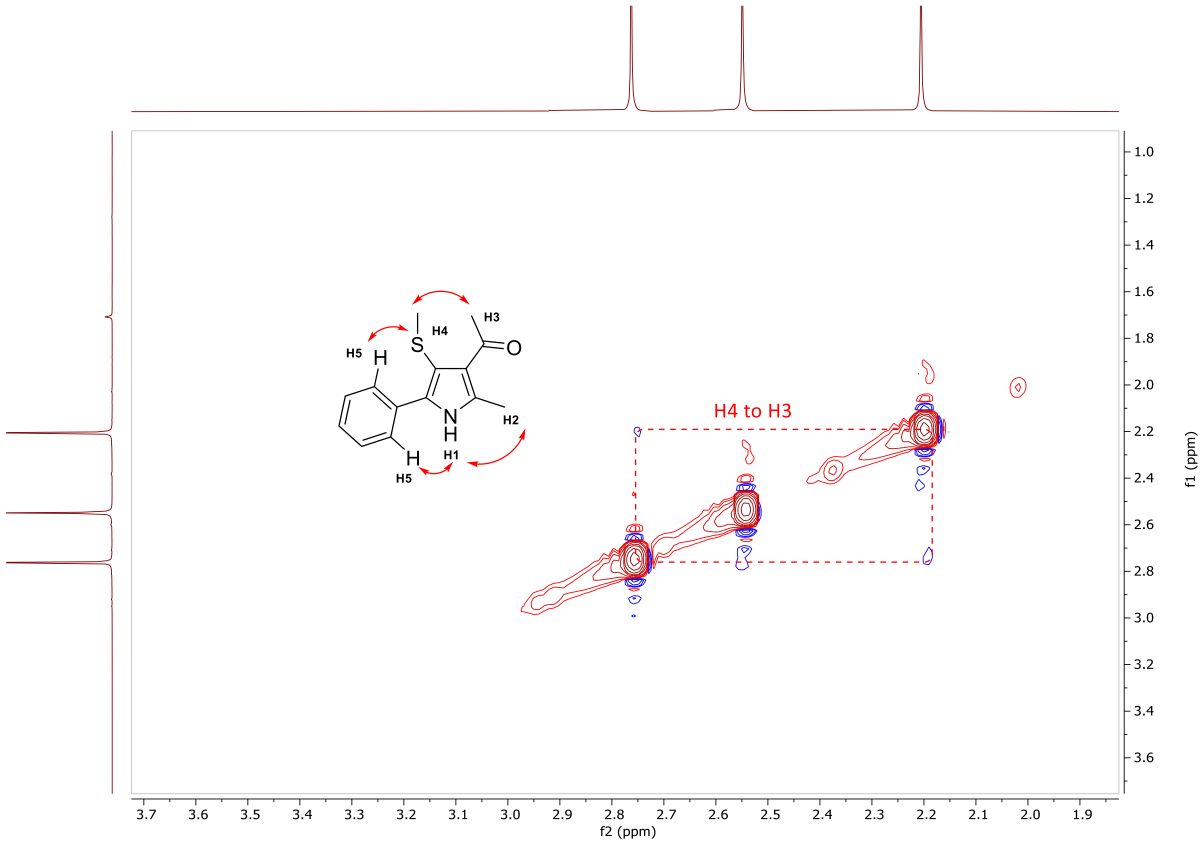

^{13}C-3l HMBC (400MHz/101MHz, $CDCl_3$).

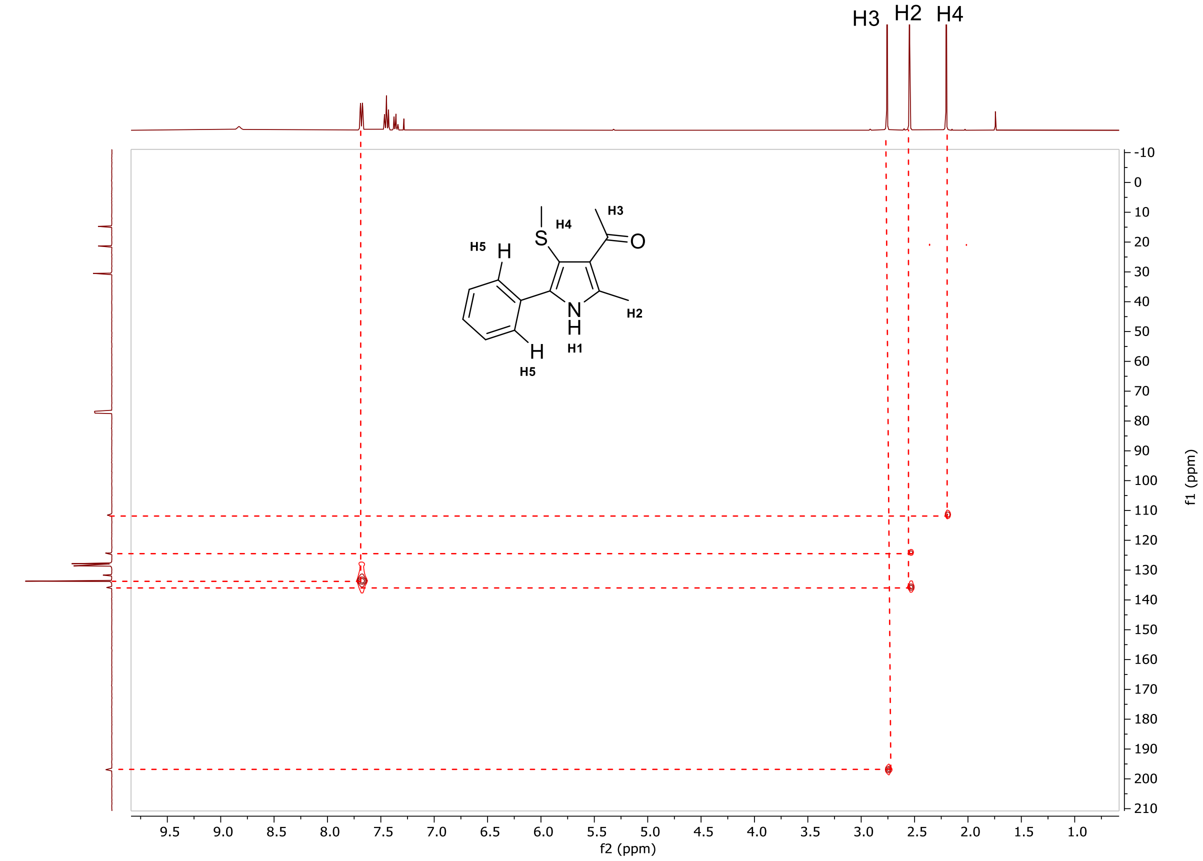

Competition experiment between anthranil (4) and 3,5-dimethylisoxazole (2a)

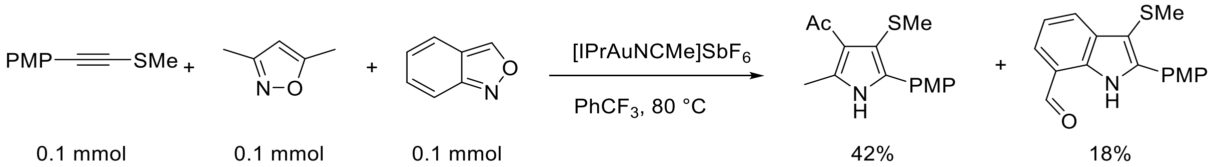

General procedure for the Au (I) catalyzed synthesis of 3-thio-4-acyl-pyrroles (3a – 3ag) (GP3)

To a 1 dram vial was charged alkynyl thioether (0.20 mmol), trifluorotoluene (2 mL) and isoxazole (2 equiv). [IPrAu(CH_3CN)]SbF_6 (8.3 mg, 0.01 mmol, 5 mol%) was added to the resultant solution, and the vial was immediately capped and transferred to an aluminium heating block preheated to 80 °C, whereupon the reaction was stirred until the alkynyl thioether was consumed, or reaction progress was deemed stagnant (determined by TLC). Upon reaction completion, the reaction mixture was allowed to cool to room temperature, and eluted through celite with dichloromethane (*ca*. 15 mL). The solvent was removed under reduced pressure, and the resultant residue was subjected to column chromatography to afford the desired pyrrole.

1-(5-(4-Methoxyphenyl)-2-methyl-4-(methylthio)-1*H*-pyrrol-3-yl)ethan-1-one (3a)

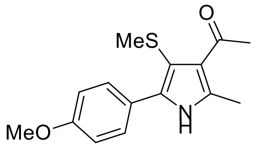

Following GP3 with alkynyl thioether **1a** (35.9 mg, 0.201 mmol), isoxazole **2a** (39.0 mg, 0.402 mmol); after reaction for 8 hours, column chromatography (SiO_2, 30 % EtOAc in hexane) afforded pyrrole **3a** as a white solid (51.4 mg, 93%). M.P: 160–163 °C; ^{1}H NMR (400 MHz, $CDCl_3$): δ = 8.43 (br, s, 1H), 7.56 (d, *J* = 9.1 Hz, 2H), 6.96 (d, *J* = 9.1 Hz, 2H), 3.85 (s, 3H), 2.74 (s, 3H), 2.52 (s, 3H), 2.17 (s, 3H); ^{13}C NMR (101 Hz, $CDCl_3$): δ = 196.8, 159.2, 135.3, 133.8, 129.2, 124.3, 124.1, 114.0, 110.7, 55.3, 30.4, 21.3, 14.7; HRMS (EI+): calculated for $C_{15}H_{17}NO_2S$: 275.0980, found 275.0978 [M]; IR (ATR): ν / cm^{-1} = 3204, 2918, 2833, 1615, 1578, 1554, 1482, 1432, 1358, 1243, 1183, 1177, 1036, 956, 829.

1-(4-(Benzylthio)-5-(4-methoxyphenyl)-2-methyl-1*H*-pyrrol-3-yl)ethan-1-one (3c)

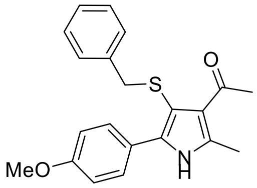

Following GP3 with alkynyl thioether **1c** (51.0 mg, 0.201 mmol), isoxazole **2a** (39.0 mg, 0.402 mmol); after reaction for 24 h, column chromatography (SiO_2, 0 to 35% EtOAc in hexane) afforded pyrrole **3c** as an off-white solid (47.1 mg, 67%). M.P: 165–167 °C; ^{1}H NMR (400 MHz, $CDCl_3$): δ = 8.70 (s, 1H), 7.43–7.30 (m, 2H), 7.18–7.08 (m, 3H), 7.01–6.93 (m, 2H), 6.93–6.82 (m, 2H), 3.83 (s, 3H), 3.68 (s, 2H), 2.62 (s, 3H), 2.50 (s, 3H); ^{13}C NMR (101 MHz, $CDCl_3$): δ = 196.9, 159.2, 137.7, 135.4, 135.1, 129.4, 128.9, 128.2, 126.9, 124.5, 124.2, 113.8, 108.1, 55.4, 42.2, 30.7, 14.8; HRMS (ES+) calculated for $C_{21}H_{22}NO_2S$: 352.1371, found 352.1373 [M+H]; IR (ATR): ν/ cm^{-1} = 3030, 2961, 1615, 1579, 1478, 1426, 1245, 1185, 1033, 832, 697.

1-(4-((4-Methoxybenzyl)thio)-5-(4-methoxyphenyl)-2-methyl-1*H*-pyrrol-3-yl)ethan-1-one (3d)

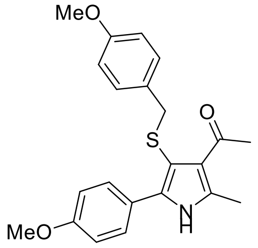

Following GP3 with alkynyl thioether **1d** (57.1 mg, 0.201 mmol), isoxazole **2a** (39.3 mg, 0.405 mmol); after reaction for 24 hours, column chromatography (SiO_2, 30% EtOAc in hexane) afforded pyrrole **3d** as an off-white solid (62.7 mg, 82%). M.P: 136–138 °C; ^{1}H NMR (300 MHz, $CDCl_3$): δ = 8.36 (br s, 1H), 7.36 (d, *J* = 9.0 Hz, 2H), 6.86 (app t, *J* = 8.9 Hz, 4H), 6.64 (d, *J* = 8.9 Hz, 2H), 3.84 (s, 3H), 3.74 (s, 3H), 3.64 (s, 2H), 2.67 (s, 3H), 2.51 (s, 3H); ^{13}C NMR (101 MHz, $CDCl_3$): δ = 196.8, 159.1, 158.5, 135.2, 134.9, 130.0, 129.8, 129.3, 124.5, 124.2, 113.7, 113.5, 108.2, 55.3, 55.2, 41.6, 30.7, 14.8; HRMS (EI+): calculated for $C_{22}H_{23}NO_3S$: 381.1399, found 381.1394 [M]; IR (ATR): v /cm^{-1} = 2934, 1616, 1609, 1579, 1510, 1478, 1426, 1359, 1252, 1185, 1032, 833, 649.

1-(4-(But-3-en-1-ylthio)-5-(4-methoxyphenyl)-2-methyl-1*H*-pyrrol-3-yl)ethan-1-one (3e)

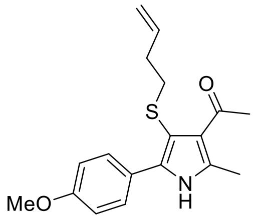

Following GP3 with alkynyl thioether **1e** (43.7 mg, 0.200 mmol), isoxazole **2a** (39.1 mg, 0.403 mmol); after reaction for 48 hours, column chromatography (SiO_2, CH_2Cl_2, to 5% EtOAc in CH_2Cl_2) afforded pyrrole **3e** as a pale-yellow gum (43.1 mg, 68%). ^{1}H NMR (400 MHz, $CDCl_3$): δ = 8.50 (br, s, 1H), 7.57 (d, *J* = 8.8 Hz, 2H), 6.95 (d, *J* = 8.8 Hz, 2H), 5.68–5.58 (m, 1H), 4.90–4.84 (m, 2H), 3.84 (s, 3H), 2.71 (s, 3H), 2.55 (t, *J* = 7.5 Hz, 2H), 2.51 (s, 3H), 2.09 (t, *J* = 7.5 Hz, 2H); ^{13}C NMR (101 MHz, $CDCl_3$): δ = 197.0, 159.2, 136.7, 135.3, 134.4, 129.4, 124.7, 124.3, 115.7, 114.0, 108.6, 55.3, 36.9, 33.3, 30.8, 14.7; HRMS (EI+): calculated for $C_{18}H_{21}NO_2S$: 315.1293, found 315.1296 [M]; IR (ATR): v /cm^{-1} = 2911, 1618, 1580, 1478, 1426, 1359, 1244, 1189, 1034, 990, 957, 917, 831, 649.

Ethyl 2-((4-acetyl-2-(4-methoxyphenyl)-5-methyl-1*H*-pyrrol-3-yl)thio)acetate (3g)

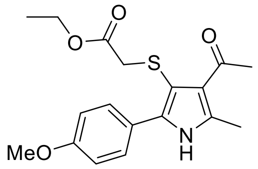

Following GP3 with alkynyl thioether **1g** (50.0 mg, 0.200 mmol), isoxazole **2a** (39.0 mg, 0.402 mmol); after reaction for 24 h, column chromatography (SiO_2, 0 to 35% EtOAc in hexane) afforded pyrrole **3g** as an off-white solid (34.2 mg, 49%). mp: 108–110 °C; ^{1}H NMR (400 MHz, $CDCl_3$): δ = 9.01 (s, 1H), 7.55 (d, *J* = 8.89 Hz, 2H), 6.90 (d, *J* = 8.89 Hz, 2H), 3.90–3.78 (m, 5H), 3.22 (s, 2H), 2.65 (s, 3H), 2.46 (s, 3H), 1.07 (t, *J* = 7.15 Hz, 3H); ^{13}C NMR (101 MHz, $CDCl_3$): δ = 196.3, 170.0, 159.3, 135.7, 135.3, 129.5, 124.3, 123.9, 114.0, 106.8, 61.2, 55.4, 39.1, 31.0, 14.8, 13.9; HRMS (ES+) calculated for $C_{18}H_{22}NO_4S$: 348.1270, found 348.1278 [M+H]; IR (ATR): ν/ cm^{-1} = 3207, 2973, 2918, 1723, 1632, 1562, 1445, 1421, 1407, 1219, 1181, 1127, 1016, 845, 798.

1-(5-(2-Methoxyphenyl)-2-methyl-4-(methylthio)-1*H*-pyrrol-3-yl)ethan-1-one (3h)

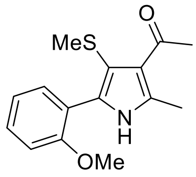

Following GP3 with alkynyl thioether **1h** (35.7 mg, 0.200 mmol), isoxazole **2a** (38.8 mg, 0.400 mmol); after reaction for 8 hours, column chromatography (SiO_2, 20% EtOAc in hexane) afforded pyrrole **3h** as a white solid (47.8 mg, 87 %). M.P: 133–136 °C; ^{1}H NMR (400 MHz, $CDCl_3$): δ = 9.02 (br s, 1H), 7.91 (dd, *J* = 7.7 Hz,

1.7 Hz, 1H), 7.32 (ddd, J = 8.2 Hz, 7.6 Hz, 1.7 Hz, 1H), 7.06 (app td, J = 7.6 Hz, 1.1 Hz, 1H), 6.99 (br d, J = 8.2 Hz, 1H), 3.87 (s, 3H), 2.74 (s, 3H), 2.53 (s, 3H), 2.19 (s, 3H); ^{13}C NMR (101 MHz, $CDCl_3$) δ 196.6, 156.5, 135.0, 131.5, 130.1, 129.2, 123.7, 121.0, 120.0, 112.7, 111.3, 55.6, 30.7, 21.4, 15.1; HRMS (EI +): calculated for $C_{15}H_{17}NO_2S$: 275.0980, found 275.0981 [M]; IR (ATR): ν / cm^{-1} = 3282, 2925, 1623, 1482, 1465, 1432, 1237, 1104, 1023, 950, 745.

Ethyl 2-((4-acetyl-2-(2-methoxyphenyl)-5-methyl-1*H*-pyrrol-3-yl)thio)acetate (3i)

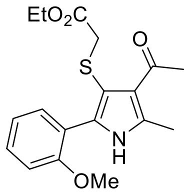

Following GP3 with alkynyl thioether **1i** (50.7 mg, 0.203 mmol), isoxazole **2a** (40 mg, 0.412 mmol); after reaction for 18 hours, column chromatography (SiO_2, 0 to 50% EtOAc in hexane over 15 minutes) afforded pyrrole **3i** as a yellow gum (49.6 mg, 71%). ^{1}H NMR (400 MHz, $CDCl_3$): δ = 9.12 (br, s, 1H), 7.91 (dd, J = 7.4 Hz, 1.7 Hz, 1H), 7.31 (ddd, J = 8.4 Hz, 7.4 Hz, 1.7 Hz, 1H), 7.04 (app td, J = 7.6 Hz, 1.2 Hz, 1H), 6.97 (br d, J = 8.4 Hz, 1H), 3.84–3.91 (m, 5H), 3.26 (s, 2H), 2.68 (s, 3H), 2.52 (s, 3H), 1.07 (t, ^3J = 7.2 Hz, 3H); ^{13}C NMR (101 MHz, $CDCl_3$): δ = 196.0, 169.7, 156.5, 134.9, 131.6, 129.3, 123.8, 120.9, 119.5, 111.2, 108.9, 61.0, 55.8, 39.3, 31.0, 15.1, 13.9; *The resonance of one quaternary carbon cannot be observed*; HRMS (ES-): calculated for $C_{18}H_{22}NO_5S$: 364.1219, found 364.1216 [M+OH]; IR (ATR): ν / cm^{-1} = 3251, 2982, 1728, 1631, 1513, 1464, 1417, 1362, 1264, 1244, 1116, 1025, 951, 732.

1-(5-Mesityl-2-methyl-4-(methylthio)-1*H*-pyrrol-3-yl)ethan-1-one (3j)

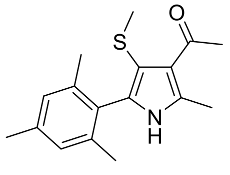

Following GP3 with alkynyl thioether **1j** (39.0 mg, 0.205 mmol), isoxazole **2a** (42.1 mg, 0.433 mmol); after reaction for 18 hours, column chromatography (SiO_2, 20% EtOAc in hexane) afforded pyrrole **3j** as a brown solid (42.8 mg, 73%). M.P: 130–133 °C; ^{1}H NMR (300 MHz, $CDCl_3$): δ = 7.99 (br s, 1H), 6.94 (s, 2H), 2.73 (s, 3H), 2.53 (s, 3H), 2.32 (s, 3H), 2.08 (s, 9H); ^{13}C NMR (101 MHz, $CDCl_3$): δ = 196.5, 138.7, 138.6, 135.4, 132.7, 128.3, 128.2, 122.8, 112.7, 30.2, 21.2, 20.3, 15.0; HRMS (EI+): calculated for $C_{17}H_{21}NOS$: 287.1344, found 287.1342 [M]·; IR (ATR): v /cm^{-1} = 3250, 2956, 2918, 2851, 1626, 1461, 1417, 1357, 1028, 955, 849.

1-(2-Methyl-4-(methylthio)-5-(naphthalen-2-yl)-1*H*-pyrrol-3-yl)ethan-1-one (3k)

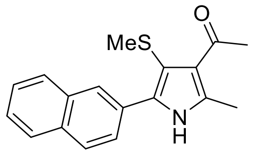

Following GP3 with alkynyl thioether **1k** (39.3 mg, 0.198 mmol), isoxazole **2a** (38.7 mg, 0.398 mmol); after reaction for 22 hours, column chromatography (SiO_2, 15% EtOAc in hexane to 20% EtOAc in hexane) afforded pyrrole **3k** as an off-white solid (44.6 mg, 76%). M.P: 122–125 °C; ^{1}H NMR (300 MHz, $CDCl_3$): δ = 8.59 (br, s, 1H), 8.05 (s, 1H), 7.92–7.84 (m, 4H), 7.54–7.47 (m, 2H), 2.77 (s, 3H), 2.56 (s, 3H), 2.19 (s, 3H); ^{13}C NMR ^{13}C NMR (101 MHz, $CDCl_3$) δ 196.9, 136.1, 133.7, 133.4, 132.8, 129.4, 128.3, 128.2, 127.9, 126.6, 126. 4, 126.0, 124.6, 30.7, 21.4, 14.9; HRMS (ES+): calculated for $C_{18}H_{17}NONaS$: 318.0929, found 318.0930 [M+Na]; IR (ATR): v /cm^{-1} = 3228, 2916, 1622, 1600, 1558, 1497, 1417, 1358, 1219, 1137, 955.

1-(2-Methyl-4-(methylthio)-5-phenyl-1*H*-pyrrol-3-yl)ethan-1-one (3l)

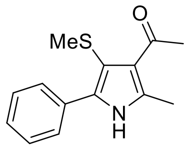

Following GP3 with alkynyl thioether **1l** (30.0 mg, 0.202 mmol), isoxazole **2a** (38.9 mg, 0.401 mmol); after reaction for 20 hours, column chromatography (SiO_2, 20% EtOAc in hexane) afforded pyrrole **3l** as an off-white solid (34.6 mg, 69%). M.P.: 129–133 °C; ^{1}H NMR (400 MHz, $CDCl_3$): δ = 8.59 (br, s, 1H), 7.65 (dd, *J* = 8.5 Hz, 1.4 Hz, 2H), 7.44 (app t, *J* = 7.6 Hz, 2H), 7.35 (tt, *J* = 7.6 Hz, 1.4 Hz, 1H), 2.74 (s, 3H), 2.52 (s, 3H), 2.18 (s, 3H); ^{13}C NMR (101 MHz, $CDCl_3$): δ = 196.8, 135.7, 133.7, 131.7, 128.6, 127.8, 127.7, 124.4, 111.6, 30.5, 21.3, 14.7; HRMS (EI+): calculated for $C_{14}H_{15}NOS$: 245.0874, found 245.0876 [M]; IR (ATR): ν / cm^{-1} = 3218, 1618, 1558, 1476, 1420, 1358, 1218, 1137, 951, 764, 790, 560.

1-(5-(4-Fluorophenyl)-2-methyl-4-(methylthio)-1*H*-pyrrol-3-yl)ethan-1-one (3m)

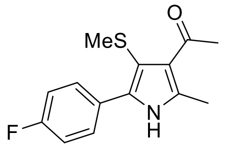

Following GP3 with alkynyl thioether **1m** (33.0 mg, 0.199 mmol), isoxazole **2a** (39.1 mg, 0.403 mmol); after reaction for 32 hours, column chromatography (SiO_2, CH_2Cl_2 to 2.5% EtOAc in CH_2Cl_2) afforded pyrrole **3m** as an off-white solid (33.8 mg, 64%). M.P: 169–171 °C; ^{1}H NMR (300 MHz, $CDCl_3$): δ = 8.34 (br s, 1H), 7.59–7.63 (m, 2H), 7.12 (app t, *J* = 7.6 Hz, 2H), 2.73 (s, 3H), 2.52 (s, 3H), 2.17 (s, 3H); ^{13}C NMR (101 MHz, $CDCl_3$) δ 196.8, 162.5 (d, C, $^1J_{C-F}$ = 246.0 Hz), 135.7, 132.9, 129.8 (d, C-H, $^3J_{C-F}$ =7.9 Hz), 127.9 (d, C, $^4J_{C-F}$ =3.3 Hz), 124.4, 115.8 (d, C-H, $^2J_{C-F}$ = 21.6 Hz), 111.8, 30.6, 21.4, 14.9; HRMS (EI+): calculated for $C_{14}H_{14}NOFS$: 263.0780, found 263.0781 [M]; IR (ATR): ν /cm^{-1} = 3199, 1619, 1560, 1480, 1411, 1358, 1216, 1159, 937, 834.

1-(5-(4-(Dimethylamino)phenyl)-2-methyl-4-(methylthio)-1*H*-pyrrol-3-yl)ethan-1-one (3n)

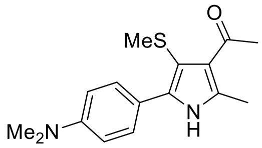

Following GP3 with alkynyl thioether **1n** (39.9 mg, 0.209 mmol), isoxazole **2a** (38.8 mg, 0.400 mmol); after reaction for 14 hours at 50 °C, column chromatography (SiO_2, 30% EtOAc in hexane) afforded pyrrole **3n** as an off-white solid (33.9 mg, 56%). M.P: 139–141 °C; ^{1}H NMR (300 MHz, $CDCl_3$): δ = 8.26 (br s, 1H), 7.51 (d, *J* = 8.9 Hz, 2H), 6.77 (d, *J* = 8.9 Hz, 2H), 3.00 (s, 6H), 2.75 (s, 3H), 2.52 (s, 3H), 2.18 (s, 3H); ^{13}C NMR (101 MHz, $CDCl_3$) δ 196.8, 150.1, 134.9, 134.6, 128.9, 124.2, 119.7, 112.3, 109.9, 40.5, 30.6, 21.5, 14.9; HRMS (EI+): calculated for $C_{16}H_{20}N_2OS$: 288.1296, found 288.1295 [M]; IR (ATR): ν /cm^{-1} = 3256, 2916, 1623, 1613, 1567, 1488, 1418, 1356, 1193, 952, 817.

1-(2-Methyl-4-(methylthio)-5-(1-tosyl-1*H*-indol-3-yl)-1*H*-pyrrol-3-yl)ethan-1-one (3o)

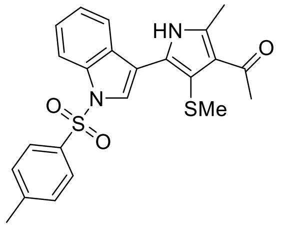

Following GP3 with alkynyl thioether **1o** (68.5 mg, 0.201 mmol), isoxazole **2a** (39.1, 0.403 mmol); after reaction for 8 hours, column chromatography (SiO_2, CH_2Cl_2 to 5% EtOAc in CH_2Cl_2) afforded pyrrole **3o** as an off-white solid (67.3 mg, 76%). M.P: 150–153 °C; ^{1}H NMR (400 MHz, $CDCl_3$): δ = 8.88 (br, s, 1H), 8.00 (d, *J* = 8.5 Hz, 1H), 7.89 (s, 1H), 7.79 (d, *J* = 8.5 Hz, 2H), 7.57 (d, *J* = 8.1 Hz, 1H), 7.33 (app t, *J* = 8.5 Hz, 1H), 7.26 (app t, *J* = 7.4 Hz, 1H), 7.21 (d,

J = 8.2 Hz, 2H), 2.70 (s, 3H), 2.52 (s, 3H), 2.33 (s, 3H), 2.06 (s, 3H); ^{13}C NMR (101 MHz, $CDCl_3$): δ = 196.6, 145.2, 136.2, 134.9, 134.8, 129.9, 129.5, 126.9, 126.2, 125.6, 125.1, 124.0, 123.7, 120.2, 114.0, 113.4, 113.4, 30.4, 21.6, 21.1, 14.8; HRMS (ES+): calculated for $C_{23}H_{23}N_2O_3S_2$: 439.1150, found 439.1154 [M+H]; IR (ATR): ν /cm^{-1} = 3206, 1635, 1432, 1371, 1175, 1130, 1117, 1096, 761, 749, 690, 660.

1-(2-Methyl-4-(methylthio)-5-(thiophen-2-yl)-1*H*-pyrrol-3-yl)ethan-1-one (3p)

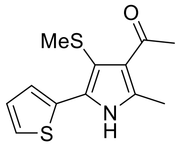

Following GP3 with alkynyl thioether **1p** (31.2 mg, 0.202 mmol), isoxazole **2a** (40.8 mg, 0.420 mmol); after reaction for 17 hours, column chromatography (SiO_2, 30% EtOAc in hexane) afforded pyrrole **3p** as a brown solid (34.1 mg, 67%). M.P: 128–130 °C; ^{1}H NMR (400 MHz, $CDCl_3$): δ = 8.58 (br, s, 1H), 7.31 (dd, *J* = 5.2 Hz, 1.0 Hz, 1H), 7.28 (dd, *J* = 3.7 Hz, 1.2 Hz, 1H), 7.07 (dd, *J* = 5.2 Hz, 3.7 Hz, 1H), 2.75 (s, 3H), 2.53 (s, 3H), 2.27 (s, 3H); ^{13}C NMR (101 MHz, $CDCl_3$): δ = 196.5, 136.0, 133.1, 128.8, 126.9, 125.5, 124.5, 123.6, 111.7, 30.6, 21.0, 14.8; HRMS (ES+): calculated for [$C_{12}H_{13}NOS_2Na$]: 274.0336, found 274.0334 [M+Na]; IR (ATR): ν / cm^{-1} = 3217, 2911, 1623, 1575, 1530, 1482, 1417, 1363, 1324, 1221, 1114, 968, 833.

1-(5-(3,4-Dimethoxyphenyl)-4-((4-methoxybenzyl)thio)-2-methyl-1*H*-pyrrol-3-yl)ethan-1-one (3q)

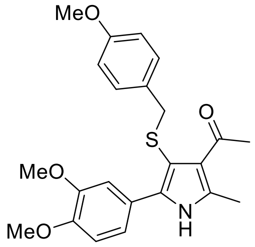

Following GP3 with alkynyl thioether **1q** (63.1 mg, 0.201 mmol), isoxazole **2a** (39.1 mg, 0.403 mmol); after reaction for 22 hours, column chromatography (SiO_2, 0 to 90% EtOAc in hexane) afforded pyrrole **3q** as a brown gum (50.0 mg, 60%). ^{1}H NMR (400 MHz, $CDCl_3$): δ = 8.56 (br s, 1H), 7.17 (d, *J* = 2.1 Hz, 1H), 6.93 (dd, *J* = 8.5 Hz, 2.0 Hz, 1H), 6.82–6.85 (m, 3H), 6.62 (d, *J* = 8.2 Hz, 2H), 3.90 (s, 3H), 3.85 (s, 3H), 3.73 (s, 3H), 3.64 (s, 2H), 2.67 (s, 3H), 2.51 (s, 3H); ^{13}C NMR (101 MHz, $CDCl_3$): δ = 196.8, 158.5, 148.5(7), 148.5(6), 135.3, 134.8, 129.9, 129.7, 124.6, 124.5, 120.7, 113.5, 111.4, 110.9, 108.3, 55.9 (2×OCH_3), 55.2, 41.5, 30.8, 14.7; HRMS (ES+): calculated for $C_{23}H_{26}NO_4S$: 412.1577, found 412.1572 $[M+H]^+$; IR (ATR): ν /cm^{-1} = 3309, 1626, 1609, 1509, 1421, 1426, 1221, 1174, 1024, 807, 647.

1-(5-Butyl-2-methyl-4-(methylthio)-1*H*-pyrrol-3-yl)ethan-1-one (3r)

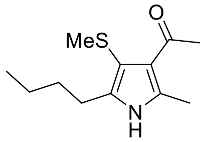

Following GP3 with alkynyl thioether **1r** (25.6 mg, 0.200 mmol), isoxazole **2a** (38.9 mg, 0.401 mmol); after reaction for 44 hours, column chromatography (SiO_2, 0 to 40% EtOAc in hexane) afforded pyrrole **3r** as an off-white solid (18.1 mg, 40%). ^{1}H NMR (400 MHz, $CDCl_3$): δ = 8.21 (br s, 1H), 2.70–2.75 (m, 5H), 2.47 (s, 3H), 2.20 (s, 3H), 1.52–1.59 (m, 2H), 1.34–1.41 (m, 2H), 0.94 (t, *J* = 7.4 Hz, 3H); ^{13}C NMR (101 MHz, $CDCl_3$): δ = 196.5, 136.0, 134.5, 122.5, 110.5, 32.2, 30.1, 25.4, 22.4, 21.7, 14.8, 13.9.; HRMS (ES+): calculated for $C_{12}H_{20}NOS$: 226.1260, found 226.1267 $[M+H]^+$; IR (ATR): ν /cm^{-1} = 3191, 2958, 2914, 1608, 1507, 1433, 1362, 1223, 1044, 954.

1-(5-Cyclopropyl-2-methyl-4-(methylthio)-1*H*-pyrrol-3-yl)ethan-1-one (3s)

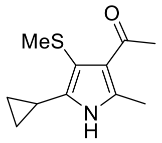

Following GP3 with alkynyl thioether **1s** (23.0 mg, 0.205 mmol), isoxazole **2a** (40.1 mg, 0.413 mmol); after reaction for 48 hours, column chromatography (SiO_2, 0 to 40% EtOAc in hexane) afforded pyrrole **3q** as a brown solid (15.0 mg, 35%). M.P: 76–80 °C; ^{1}H NMR (300 MHz, $CDCl_3$): δ = 7.89 (br s, 1H), 2.70 (s, 3H), 2.43 (s, 3H), 2.26 (s, 3H), 2.12–2.19 (m, 1H), 0.95–1.00 (m, 2H), 0.66–0.70 (m, 2H); ^{13}C NMR (101 MHz, $CDCl_3$): δ = 196.4, 135.9, 133.6, 123.1, 111.5, 30.4, 21.2, 14.7, 7.4, 6.7; HRMS (EI+): calculated for $C_{11}H_{15}NOS$: 209.0874, found 209.0873 [M] ; IR (ATR): v /cm^{-1} = 3206, 2917, 1613, 1506, 1417, 1361, 1223, 954, 761.

1-(2-Methyl-4-(methylthio)-5-(phenylthio)-1*H*-pyrrol-3-yl)ethan-1-one (3t) and 1-(2-methyl-5-(methylthio)-4-(phenylthio)-1*H*-pyrrol-3-yl)ethan-1-one (3t')

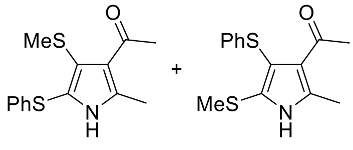

3t **3t'**

Following GP3 with alkynyl thioether **1t** (38.5 mg, 0.214 mmol), isoxazole **2a** (49 mg, 0.504 mmol); after reaction for 8 hours, column chromatography (SiO_2, 0 to 35% EtOAc in hexane over 16 minutes) afforded pyrrole **3t** as a red solid (24.6 mg, 41%), and the opposing regioisomer **3t'** as an impure fraction. Further column chromatography of the latter (SiO_2, 0 to 100% CH_2Cl_2 in hexane) afforded **3t'** as a red gum (10.8 mg, 18%).

1-(2-Methyl-4-(methylthio)-5-(phenylthio)-1*H*-pyrrol-3-yl)ethan-1-one (3t) M.P: 94–99 °C; ^{1}H NMR (400 MHz, $CDCl_3$): δ = 8.52 (br, s, 1H), 7.22–7.27 (m, 2H), 7.14 (tt, *J* = 7.2 Hz, 1.1 Hz, 1H), 7.07 (d, *J* = 8.2 Hz, 2H), 2.74 (s, 3H), 2.49 (s, 3H), 2.26 (s, 3H); ^{13}C NMR (101 MHz, $CDCl_3$): δ = 196.1, 138.5, 137.2, 129.2, 126.6, 126.1, 124.5, 124.0, 121.2, 30.3, 21.1, 14.9; HRMS (EI+): calculated for $C_{14}H_{15}NOS_2$: 277.0595, found 277.0594 [M]·; IR (ATR): ν / cm^{-1} = 3193, 3055, 2967, 2874, 1631, 1583, 1477, 1423, 1407, 1345, 1225, 1023, 950, 781, 732

1-(2-Methyl-5-(methylthio)-4-(phenylthio)-1*H*-pyrrol-3-yl)ethan-1-one (3t') ^{1}H NMR (400 MHz, $CDCl_3$): δ = 8.63 (br, s, 1H), 7.19–7.21 (m, 2H), 7.09 (t, *J* = 7.5 Hz, 1H), 7.03 (dd, *J* = 8.5 Hz, 1.2 Hz, 2H), 2.56 (s, 3H), 2.50 (s, 3H), 2.26 (s, 3H); ^{13}C NMR (101 MHz, $CDCl_3$): δ = 196.0, 139.3, 138.4, 129.1, 128.4, 125.6, 125.1, 124.4, 115.6, 30.3, 20.6, 15.2; HRMS (EI+): calculated for $C_{14}H_{16}NOS_2$: 278.0673, found 278.0676 [M+H]; IR (ATR): ν / cm^{-1} = 3196, 3059, 2921, 1627, 1587, 1539, 1412, 1352, 1225, 1207, 1024, 953, 736, 689.

(5-(4-Methoxyphenyl)-4-(methylthio)-2-phenyl-1*H*-pyrrol-3-yl)(phenyl)methanone (3u)

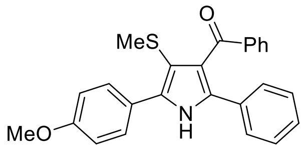

Following GP3 with alkynyl thioether **1a** (36.7 mg, 0.206 mmol), isoxazole **2b** (91.8 mg, 0.415 mmol); after reaction for 14 hours, column chromatography (SiO_2, 10% EtOAc in hexane to 25% EtOAc in hexane) afforded pyrrole **3u** as a yellow solid (78.7 mg, 95%). M.P: 67–71 °C; ^{1}H NMR (400 MHz, $CDCl_3$): δ = 8.50 (br s, 1H), 7.85 (d, *J* = 7.8 Hz, 2H), 7.73 (d, *J* = 8.8 Hz, 2H), 7.42 (t, *J* = 7.3 Hz, 1H), 7.27–7.32 (m, 4H), 7.16–7.23 (m, 3H), 7.01 (d, *J* = 8.8 Hz, 2H), 3.87 (s, 3H), 2.19 (s, 3H); ^{13}C NMR (101 MHz,

$CDCl_3$): δ = 194.3, 159.4, 138.6, 135.1, 133.3, 132.6, 131.3, 130.1, 128.8, 128.7, 128.0, 127.7, 127.2, 125.5, 124.0, 114.2, 113.2, 55.4, 20.9; HRMS (ES+): calculated for $C_{25}H_{22}NO_2S$: 400.1371, found 400.1373 [M+H]; IR (ATR): ν /cm^{-1} = 3282, 2919, 1612, 1596, 1577, 1492, 1474, 1246, 1177, 1024, 908, 831, 768, 732, 692.

(5-(4-Methoxyphenyl)-4-(methylthio)-2-phenyl-1*H*-pyrrol-3-yl)(phenyl)methanone (3v)

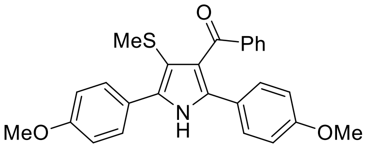

Following GP3 with alkynyl thioether **1a** (35.9 mg, 0.201 mmol), isoxazole **2c** (101.8 mg, 0.405 mmol); after reaction for 8 hours, column chromatography (SiO_2, 10% EtOAc in hexane to 20% EtOAc in hexane) afforded pyrrole **3v** as a yellow solid (83.4 mg, 96 %.) M.P: 69–73 °C; ^{1}H NMR (400 MHz, $CDCl_3$): δ = 8.39 (br s, 1H), 7.85 (d, *J* = 8.2 Hz, 2H), 7.71 (d, *J* = 8.9 Hz, 2H), 7.42 (t, *J* = 7.4 Hz, 1H), 7.21–7.33 (m, 4H), 7.01 (d, *J* = 8.5 Hz, 2H), 6.75 (d, *J* = 8.9 Hz, 2H), 3.87 (s, 3H), 3.74 (s, 3H), 2.18 (s, 3H); ^{13}C NMR (101 MHz, $CDCl_3$): δ = 194.2, 159.32, 159.25, 138.7, 134.6, 133.6, 132.5, 130.1, 128.8, 128.7, 128.0, 124.7, 124.2, 124.0, 114.2, 114.1, 113.0, 55.4, 55.3, 20.9.; HRMS (ES+): calculated for $C_{26}H_{24}NO_3S$: 430.1477, found 430.1480 [M+H]; IR (ATR): ν /cm^{-1} = 3266, 2923, 1612, 1577, 1501, 1475, 1439, 1245, 1176, 1027, 909, 830, 729.

1-(2,5-Bis(4-methoxyphenyl)-4-(methylthio)-1*H*-pyrrol-3-yl)pentan-1-one (3w)

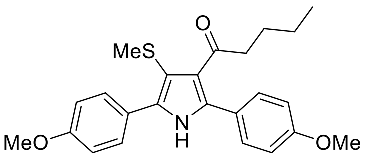

Following GP3 with alkynyl thioether **1a** (35.7 mg, 0.200), isoxazole **2d** (92.1 mg, 0.394 mmol); after reaction for 8 hours, column chromatography (SiO_2, 25% EtOAc in hexane) afforded pyrrole **3w** as a yellow gum (74.4 mg, 91%). ^{1}H NMR (400 MHz, $CDCl_3$): δ = 8.32 (br s, 1H), 7.63 (d, *J* = 8.7 Hz, 2H), 7.37 (d, *J* = 8.7 Hz, 2H), 6.98 (d, *J* = 8.7 Hz, 2H), 6.94 (d, *J* = 8.7 Hz, 2H), 3.85 (s, 3H), 3.84 (s, 3H), 2.67 (t, *J* = 7.9 Hz, 2H), 1.59 (app. quint, *J* = 7.5 Hz, 2H), 1.25 (app. quint, *J* = 7.5 Hz, 2H), 0.82 (t, *J* = 7.5 Hz, 3H); ^{13}C NMR (101 MHz, $CDCl_3$): δ = 201.1, 159.8, 159.3, 134.8, 134.3, 129.7, 129.1, 126.4, 124.7, 124.2, 114.2, 114.1, 111.8, 55.39, 55.36, 42.9, 27.0, 22.4, 21.1, 13.9. HRMS (EI+): calculated for $C_{24}H_{27}NO_3S$: 409.1712, found 409.1711 [M]; IR (ATR): ν/ cm^{-1} = 3217, 2959, 1634, 1613, 1502, 1439, 1246, 1177, 1031, 831, 732.

5-(4-Methoxyphenyl)-4-(methylthio)-1*H*-pyrrole-3-carbaldehyde (3x)

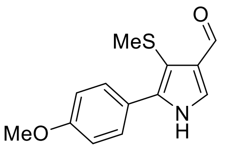

Following GP3 with alkynyl thioether **1a** (35.5 mg, 0.199), isoxazole **2e** (56.6 mg, 0.820 mmol); after reaction for 21 hours, column chromatography (SiO_2, 25 to 40% EtOAc in hexane) afforded pyrrole **3x** as a brown solid (36.9 mg, 74%). M.P: 132–135 °C; ^{1}H NMR (400 MHz, $CDCl_3$): δ = 10.03 (s, 1H), 8.93 (br s, 1H), 7.60 (d, *J* = 8.7 Hz, 2H), 7.49 (d, *J* = 3.2 Hz, 1H), 6.99 (d, *J* = 8.7 Hz, 2H), 3.85 (s, 3H), 2.29 (s, 3H); ^{13}C NMR (101 MHz, $CDCl_3$): δ = 186.7, 159.6, 136.0, 129.1, 127.4, 125.7, 123.4, 114.2, 112.1, 55.4, 20.7.; HRMS (EI+): calculated for $C_{13}H_{13}NO_2S$: 247.0667, found 247.0668 [M]; IR (ATR): ν /cm^{-1} = 3267, 1646, 1636, 1551, 1490, 1397, 1369, 1260, 1245, 1181, 1024, 830, 776, 751.

5-(4-Methoxyphenyl)-4-(methylthio)-2-phenyl-1*H*-pyrrole-3-carboxamide (3y)

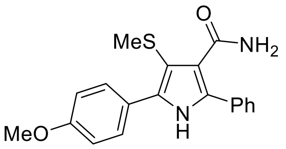

Following GP3 with alkynyl thioether **1a** (36.1 mg, 0.203 mmol), isoxazole **2f** (64.5 mg, 0.403 mmol); after reaction for 48 hours, column chromatography (SiO2, 10% to 40% EtOAc in CH_2Cl_2) afforded pyrrole **3y** as an off-white solid (27 mg, 39%). M.P: 120–124 °C; ^{1}H NMR (300 MHz, $CDCl_3$): δ = 8.37 (br s, 1H), 7.68 (br s, 1H), 7.58–7.61 (m, 3H), 7.34–7.46 (m, 3H), 7.00 (d, *J* = 8.8 Hz, 1H), 5.46 (br, s, 1H), 3.87 (s, 3H), 2.29 (s, 3H); ^{13}C NMR (101 MHz, DMSO-d_6): δ = 168.0, 158.5, 134.4, 132.0, 129.3, 129.2, 128.2, 126.8, 126.7, 124.1, 123.7, 113.7, 109.0, 55.2, 20.5; HRMS (ES+): calculated for $C_{19}H_{19}N_2O_2S$: 339.1167, found 339.1169 [M+H]; IR (ATR): ν/ cm^{-1} = 3172, 1646, 1576, 1494, 1487, 1246, 1179, 1031, 832, 768, 694.

5-(4-Methoxyphenyl)-2-methyl-4-(methylthio)-1*H*-pyrrole-3-carboxamide (3z)

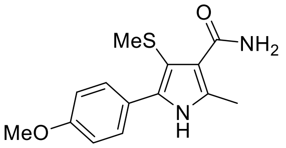

Following GP3 with alkynyl thioether **1a** (35.8 mg, 0.200 mmol), isoxazole **2g** (40.2 mg, 0.400 mmol); after reaction for 48 h, column chromatography (SiO_2, 50-70% EtOAc in hexane) afforded pyrrole **3z** as off-white solid (25.1 mg, 45%). M.P: 195–197 °C; ^{1}H NMR (400 MHz, CD_3OD): δ = 7.60 (d, *J* = 8.9 Hz, 2H), 6.97 (d, *J* = 8.8 Hz, 2H), 3.83 (s, 3H), 2.52 (s, 3H), 2.15 (s, 3H); ^{13}C NMR (101 MHz, CD_3OD): δ = 170.7, 160.6, 137.3, 135.8, 130.4, 125.7, 115.5, 114.7, 108.4, 55.7, 21.3, 13.8; HRMS (ES+) calculated for $C_{14}H_{17}N_2O_2S$: 277.1011, found 277.1008 [M+H]; IR (ATR): ν/ cm^{-1} = 3324, 3145, 1648, 1610, 1595, 1534, 1489, 1415, 1241, 1110, 833.

(*E*)-(5-(4-Methoxyphenyl)-4-(methylthio)-2-styryl-1*H*-pyrrol-3-yl)(phenyl)methanone (3aa)

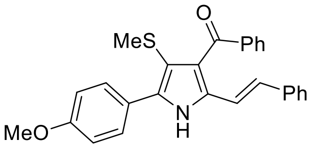

Following GP3 with alkynyl thioether **1a** (37 mg, 0.208 mmol), isoxazole **2h** (102.6 mg, 0.415 mmol); after reaction for 16 hours, column chromatography (SiO_2, 10% EtOAc in hexane to 20% EtOAc in hexane) afforded pyrrole **3aa** as an orange solid (75.7 mg, 86%). M.P: 84–87 °C; ^{1}H NMR (400 MHz, $CDCl_3$): δ = 8.70 (br s, 1H), 7.93 (d, *J* = 8.4 Hz, 2H), 7.72 (d, *J* = 8.7 Hz, 2H), 7.58 (app t, *J* = 7.2 Hz, 1H), 7.47 (app t, *J* = 7.2 Hz, 2H), 7.27–7.29 (m, 4H), 7.19–7.23 (m, 1H), 7.01 (d, *J* = 8.7 Hz, 2H), 6.95 (d, *J* = 16.4 Hz, 1H), 6.76 (d, *J* =16.4 Hz, 1H), 3.87 (s, 3H), 2.07 (s, 3H); ^{13}C NMR (101 MHz, $CDCl_3$): δ = 193.4, 159.6, 139.6, 136.5, 135.3, 132.7, 132.5, 130.1, 129.0, 128.7, 128.2, 127.8, 127.1, 126.7, 126.3, 123.8, 117.0, 114.3, 113.3, 55.4, 20.6; HRMS (ES+): calculated for $C_{27}H_{24}NO_2S$: 426.1528, found 426.1527 [M+H]; IR (ATR): v /cm^{-1} = 3270, 1610, 1595, 1515, 1472, 1430, 1346, 1276, 1176, 1031, 957, 923, 830, 692.

(*E*)-(5-(4-Fluorophenyl)-4-(methylthio)-2-styryl-1*H*-pyrrol-3-yl)(phenyl)methanone (3ab)

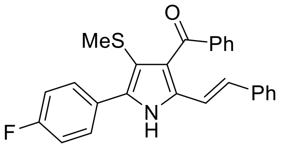

Following GP3 with alkynyl thioether **1m** (33.3 mg, 0.200 mmol), isoxazole **2h** (110.1 mg, 0.401 mmol); after reaction for 21 hours, column chromatography (SiO_2, 60% CH_2Cl_2 in hexane to 80% CH_2Cl_2 in hexane)

afforded pyrrole **3ab** as an orange solid (61.8 mg, 75%). M.P: 136–140 °C; ^{1}H NMR (400 MHz, $CDCl_3$): δ = 8.88 (br s, 1H), 7.92 (dd, J = 8.2 Hz, 1.5 Hz, 2H), 7.77 (dd, J = 8.8 Hz, 5.3 Hz, 2H), 7.59 (tt, J = 7.5 Hz, 1.1 Hz, 1H), 7.47 (t, J = 7.5 Hz, 2H), 7.14–7.41 (m, 7H), 6.94 (d, J = 16.5 Hz, 1H), 6.78 (d, J = 16.5 Hz, 1H), 2.07 (s, 3H); ^{13}C NMR (101 MHz, $CDCl_3$): δ = 193.4, 162.5 (d, $^1J_{C\text{-}F}$ = 248 Hz), 139.4, 136.4, 134.4, 133.0, 132.8, 130.1, 129.6 (d, $^3J_{C\text{-}F}$ = 8.1 Hz), 128.7, 128.3, 128.0, 127.4 (d, $^4J_{C\text{-}F}$ = 4.0 Hz), 127.2, 127.0, 126.3, 116.8, 115.8 (d, $^2J_{C\text{-}F}$ = 21. 9 Hz), 114.1, 20.6.; HRMS (ES+): calculated for $C_{26}H_{21}NOFS$: 414.1328, found 414.1327 [M+H]; IR (ATR): ν /cm^{-1} = 3243, 1613, 1576, 1595, 1515, 1473, 1448, 1434, 1346, 1226, 1159, 964, 923, 934, 754, 733, 723, 691.

1-(2,5-Bis(4-methoxyphenyl)-4-(methylthio)-1*H*-pyrrol-3-yl)-2-hydroxyethan-1-one (3ac)

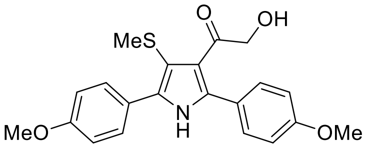

Following GP3 with alkynyl thioether **1a** (72.4 mg, 0.406 mmol), isoxazole **2i** (41 mg, 0.200 mmol); after reaction for 14 hours, column chromatography (SiO_2, 40% EtOAc in hexane) afforded pyrrole **3ac** as a brown solid (43.8 mg, 57%). M.P.: 210–213 °C; ^{1}H NMR (400 MHz, $CDCl_3$): δ = 8.38 (br s, 1H), 7.64 (d, J = 8.6 Hz, 2H), 7.44 (d, J = 8.6 Hz, 2H), 7.00–7.03 (m, 4H), 4.56 (d, J = 4.5 Hz, 2H), 3.88(9) (s, 3H), 3.88(5) (s, 3H), 3.70 (t, J = 4.8 Hz, 1H), 2.32 (s, 3H); ^{13}C NMR (101 MHz, acetone-d_6): δ = 197.3, 160.1, 159.4, 136.2, 135.8, 130.3, 129.5, 124.4, 124.0, 122.1, 113.8, 113.7, 110.8, 67.8, 54.8, 54.7, 20.4.; HRMS (ES+): calculated for $C_{21}H_{22}NO_4S$: 384.1264, found 384.1263 [M+H]$^+$; IR (ATR): ν/ cm^{-1} = 3242, 2158, 1699, 1635, 1610, 1506, 1388, 1249, 1181, 1050, 1025, 980, 932, 865, 832, 760.

2-(2,5-Bis(4-methoxyphenyl)-4-(methylthio)-1*H*-pyrrol-3-yl)-2-oxoethyl acetate (3ad)

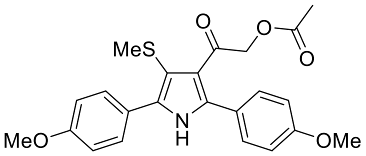

Following GP3 with alkynyl thioether **1a** (73.3 mg, 0.411 mmol), isoxazole **2j** (53.2 mg, 0.220 mmol); after reaction for 45 hours, column chromatography (SiO_2, 0 to 50% EtOAc in hexane over 16 minutes) afforded pyrrole **3ad** as an off-white foam (42.9 mg, 46%). ^{1}H NMR (400 MHz, $CDCl_3$): δ = 8.34 (br s, 1H), 7.62 (d, J = 8.9 Hz, 2H), 7.45 (d, J = 8.7 Hz, 2H), 6.96–7.00 (m, 4H), 5.10 (s, 2H), 3.86 (s, 3H), 3.85 (s, 3H), 2.30 (s, 3H), 2.16 (s, 3H); ^{13}C NMR (101 MHz, $CDCl_3$): δ = 191.3, 170.6, 160.3, 159.7, 136.4, 135.5, 130.2, 129.3, 124.1, 123.8, 122.3, 114.5, 114.3, 112.0, 68.7, 55.5 (x 2 OMe), 21.2, 20.8; HRMS (ES+): calculated for $C_{23}H_{24}NO_5S$ 426.1375, found 426.1376 [M+H]; IR (ATR): ν/ cm^{-1} = 3260, 1730, 1688, 1610, 1567, 1504, 1468, 1429, 1275, 1246, 1183, 1173, 1021, 931.

(2-Cyclohexyl-5-(4-methoxyphenyl)-4-(methylthio)-1*H*-pyrrol-3-yl)(phenyl)methanone (3ae)

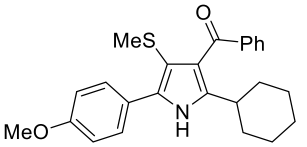

Following GP3 with alkynyl thioether **1a** (35.5 mg, 0.199 mmol), isoxazole **2k** (97.5 mg, 0.429 mmol); after reaction for 23 hours, column chromatography (SiO_2, hexane to 40% EtOAc in hexane over 15 minutes) afforded pyrrole **3ae** as a yellow solid (71.3 mg, 88%). M.P: 68–73 °C; ^{1}H NMR (400 MHz, $CDCl_3$): δ = 8.22 (br s, 1H), 7.88 (d, J = 8.7 Hz, 2H), 7.62 (dm, J = 8.9 Hz, 2H), 7.56 (tt, J = 7.5 Hz, 1H), 7.45 (t, J = 7.5 Hz, 2H), 6.98 (d, J = 8.7 Hz, 2H), 3.85 (s, 3H), 2.81 (tt, J_{TRANS} = 11.9 Hz, J_{CIS} = 3.4 Hz, 1H),

2.00 (s, 3H), 1.94 (br d, J = 12.5 Hz, 2H), 1.74–1.78 (br m, 2H), 1.66–1.69 (br m, 1H), 1.13–1.41 (m, 5H); ^{13}C NMR (101 MHz, $CDCl_3$): δ = 194.5, 159.2, 141.6, 140.2, 132.4(4), 132.4(0), 129.9, 128.8, 128.2, 124.6, 123.4, 114.3, 111.2, 55.5, 36.0, 33.4, 26.5, 26.0, 20.7.; HRMS (ES+): calculated for $C_{25}H_{28}NO_2S$: 406.1841, found 406.1846 [M+H]; IR (ATR): ν /cm^{-1} = 3288, 2922, 2851, 1614, 1483, 1448, 1246, 1176, 1033, 916, 830, 694.

(2-(2-Bromophenyl)-5-(4-methoxyphenyl)-4-(methylthio)-1*H*-pyrrol-3-yl)(phenyl)methanone (3af)

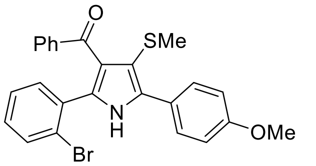

Following GP3 with alkynyl thioether **1a** (36.1 mg, 0.203 mmol), isoxazole **2l** (121.1 mg, 0.403 mmol); after reaction for 96 hours, column chromatography (SiO_2, hexane to 100% CH_2Cl_2 in hexane over 15 minutes) afforded pyrrole **3af** as a yellow gum (86.8 mg, 89%). ^{1}H NMR (400 MHz, $CDCl_3$): δ = 8.78 (br s, 1H), 7.70–7.77 (m, 4H), 7.47 (dd, J = 7.8 Hz, 1.4 Hz, 1H), 7.32 (app t, J = 7.3 Hz, 1H), 7.15–7.24 (m, 3H), 6.97–7.07 (m, 4H), 3.85 (s, 3H), 2.26 (s, 3H); ^{13}C NMR (101 MHz, $CDCl_3$): δ = 192.9, 159.2, 138.6, 134.8, 132.9, 132.8, 132.4, 132.0, 131.9, 129.6, 129.5, 129.4, 128.8, 127.5, 126.9, 126.4, 123.7, 122.2, 114.0, 113.9, 112.4, 55.1, 20.7; HRMS (ES+): calculated for $C_{25}H_{21}NO_2SBr$: 478.0476, found 478.0474 [M+H]; IR (ATR): ν /cm^{-1} = 3272, 1634, 1486, 1461, 1248, 1179, 1027, 910, 832, 740, 697.

Unsuccessful reactions

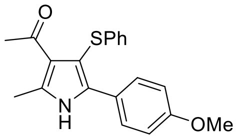

3b^a

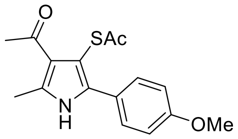

3f^b

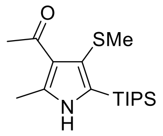

3ag^a

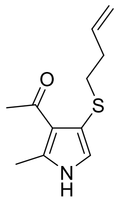

3ah^b

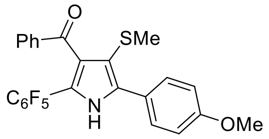

3ai^a

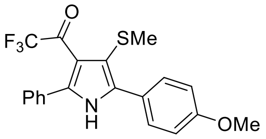

3aj^a

^{a}*Starting material not consumed* ^{b}*Starting material decomposed.*

Procedure for the reaction of 1a and 2a on gram scale:

To a 250 mL round bottom flask was charged alkynyl thioether **1a** (1.353 g, 7.6 mmol), 3,5-dimethylisoxazole **2a** (1.500 g, 15.4 mmol), and trifluorotoluene (75 mL). IPrAuCl (257 mg, 5 mol%), followed by $AgSbF_6$ (212 mg, 7.5%) was added to the resultant solution, and the flask was immediately

stoppered and immersed in an oil bath preheated to 80 °C, whereupon the reaction was stirred for 16 hours. The reaction was allowed to cool to room temperature and filtered through celite (eluting with CH_2Cl_2). The solvent was removed *in vacuo*, and the residue dissolved in CH_2Cl_2, adsorbed onto SiO_2, and chromatographed (SiO_2, CH_2Cl_2 to 20% EtOAc in CH_2Cl_2) to yield **3a** as an off-white solid (1.81 g, 86%). Analytical data is identical to that obtained for reaction on a 0.2 mmol scale.

Au (I) catalyzed synthesis of 3-thio-7-acyl-indoles (5)

Indoles (**5**) were synthesised according to **GP3**, with the modification that 0.2 mmol of anthranil (**4**) was used to a stoichiometric excess of alkynyl thioether (1.5 or 2 equivalents).

2-(4-Methoxyphenyl)-3-(methylthio)-1*H*-indole-7-carbaldehyde (5a)

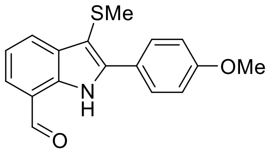

Following GP3 with alkynyl thioether **1a** (53.1 mg, 0.298 mmol), anthranil **4** (25.3 mg, 0.212 mmol); after reaction for 7 hours, column chromatography (SiO_2, 0 to 20% EtOAc in hexane) yielded indole **5a** as a yellow solid (36.4 mg, 58%). M.P: 136–138 °C; ^{1}H NMR (300 MHz, $CDCl_3$): δ = 10.22 (br s, 1H), 10.14 (s, 1H), 8.08 (d, *J* = 7.6 Hz, 1H), 7.88 (d, *J* = 8.8 Hz, 2H), 7.68 (d, *J* = 7.6 Hz, 1H), 7.35 (app t, *J* = 8.0 Hz, 1H), 7.05 (d, *J* = 8.8 Hz, 2H), 3.89 (s, 3H), 2.29 (s, 3H); ^{13}C NMR (101 MHz, $CDCl_3$): δ = 193.7, 160.2, 141.6, 133.7, 132.5, 129.7, 129.1, 126.6, 123.9, 120. 5, 120.3, 114.4, 104.3, 55.5, 20.1; HRMS (ES+): calculated for $C_{17}H_{16}NO_2S$: 298.0902, found 298.0901 [M+H]; IR (ATR): ν/cm^{-1} = 3309, 1677, 1604, 1592, 1490, 1353, 1244, 1178, 1166, 1083, 1033, 982, 832, 791, 747, 736, 694, 667.

3-(But-3-en-1-ylthio)-2-(4-methoxyphenyl)-1*H*-indole-7-carbaldehyde (5b)

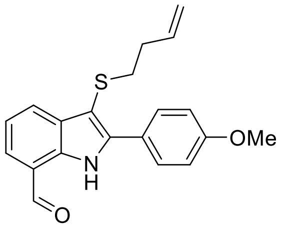

Following GP3 with alkynyl thioether **1e** (88.8 mg, 0.407 mmol), anthranil **4** (24.4 mg, 0.204 mmol); after reaction for 24 hours, column chromatography (SiO_2, 0 to 20% EtOAc in hexane) afforded indole **5b** as an orange solid (26.6 mg, 39%). M.P: 106–109 °C; ^{1}H NMR (400 MHz, $CDCl_3$): δ = 10.26 (br s, 1H), 10.14 (s, 1H), 8.08 (d, *J* = 7.8 Hz, 1H), 7.91 (d, *J* = 9.0 Hz, 2H), 7.68 (dd, *J* = 7.4 Hz, 1.1 Hz, 1H), 7.35 (dd, *J* = 7.8 Hz, 7.6 Hz, 1H), 7.05 (d, *J* = 9.0 Hz, 2H), 5.65–5.76 (m, 1H), 4.86 4.94 (m, 2H), 3.89 (s, 3H), 2.70 (t, *J* = 7.6 Hz, 2H), 2.14–2.21 (m, 2H).; ^{13}C NMR (101 MHz, $CDCl_3$): δ = 193.5, 160.1, 142.4, 136.6, 133.6, 133.0, 129.7, 129.0, 126.7, 123.8, 120.3, 120.2, 115.9, 114.2, 102.3, 55.4, 35.9, 33.9.; HRMS (ES+): calculated for $C_{20}H_{20}NO_2S$: 338.1209, found 338.1208 $[M+H]^+$; IR (ATR): ν/ cm^{-1} = 3267, 1646, 1636, 1551, 1490, 1260, 1245, 1181, 1145, 1096, 1056, 1024, 830, 776, 751.

3-(Methylthio)-2-(naphthalen-2-yl)-1*H*-indole-7-carbaldehyde (5c)

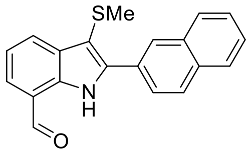

Following GP3 with alkynyl thioether **1l** (61.5 mg, 0.310 mmol), anthranil **4** (26.0 mg, 0.218 mmol); after reaction for 8 hours, column chromatography (SiO_2, 0 to 20% EtOAc in hexane) afforded indole **5c** as an orange solid (38.2 mg, 55%). M.P: 88–92 °C; ^{1}H NMR (400 MHz, $CDCl_3$): δ = 10.42 (br s, 1H), 10.17 (s, 1H), 8.37 (s, 1H), 8.13–8.16 (m, 2H), 7.96–8.00 (m, 2H), 7.89–7.92 (m, 1H), 7.73 (dd, *J* = 7.4 Hz, 1.1 Hz, 1H), 7.54–7.57 (m, 2H), 7.39 (app t, *J* = 7.7 Hz, 1H), 2.32 (s, 3H); ^{13}C NMR (101 MHz, $CDCl_3$): δ = 193.6, 141.4, 133.8, 133.3, 133.2, 132.4, 129.4, 128.7, 128.5, 127.9, 127.8, 127.5, 126.8, 126.7, 125.7, 120.5, 120.4, 105.8, 20.1; IR (ATR): HRMS (ES+): calculated for $C_{20}H_{16}NOS$: 318.0947, found 318.0949 $[M+H]^+$; ν/ cm^{-1} = 3320, 1665, 1591, 1505, 1351, 1332, 1290, 1271, 1217, 1170, 1078, 1058, 962, 897, 808, 814, 756, 687.

2-(4-Fluorophenyl)-3-(methylthio)-1*H*-indole-7-carbaldehyde (5d)

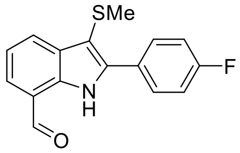

Following GP3 with alkynyl thioether **1m** (50.5 mg, 0.303 mmol), anthranil **4** (23.9 mg, 0.201 mmol); after reaction for 12 hours, column chromatography (SiO_2, 0 to 20% EtOAc in hexane) afforded indole **5d** as an orange solid (23.0 mg, 40%). ^{1}H NMR (400 MHz, $CDCl_3$): δ = 10.25 (br s, 1H), 10.14 (s, 1H), 8.10 (d, *J* = 7.8 Hz, 1H), 7.91 (m, 2H), 7.71 (dd, *J* = 7.4 Hz, 1.1 Hz, 1H), 7.37 (app t, *J* = 7.6 Hz, 1H), 7.19–7.25 (m, 2H), 2.29 (s, 3H); ^{13}C NMR (101 MHz, $CDCl_3$): δ = 193.5, 162.0 (d, C, $^1J_{C\text{-}F}$ = 250 Hz), 140.5, 133.6, 132.1, 130.2 (d, CH, $^3J_{C\text{-}F}$ = 8.5 Hz), 129.4, 127.4 (d, C, $^4J_{C\text{-}F}$ = 3.5 Hz), 126.8, 120.5, 120.4, 115.9 (d, CH, $^2J_{C\text{-}F}$ = 21.7 Hz), 105.3, 20.0.; HRMS (EI+) calculated for $C_{16}H_{12}NOFS$: 285.0624, found 285.0623 [M]; IR (ATR): ν/ cm^{-1} = 3313, 2918, 2916, 1673, 1590, 1488, 1350, 1219, 1159, 1085, 983, 838, 791, 752, 747, 715, 665.

3-(Methylthio)-2-(thiophen-2-yl)-1*H*-indole-7-carbaldehyde (5e)

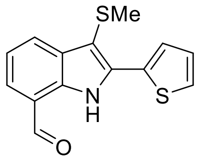

Following GP3 with alkynyl thioether **1p** (49.0 mg, 0.318 mmol), anthranil **4** (24.3 mg, 0.204 mmol); after reaction for 7 hours, column chromatography (SiO_2, 0 to 20% EtOAc in hexane) afforded **5e** as an orange solid (25.8 mg, 47%). M.P: 109–112 °C; ^{1}H NMR (300 MHz, $CDCl_3$): δ = 10.30 (br s, 1H), 10.13 (s, 1H), 8.06 (d, *J* = 7.9 Hz, 1H), 7.69 (dd, *J* = 7.4 Hz, 1.1 Hz, 1H), 7.64 (dd, *J* = 3.7 Hz, 1.1 Hz, 1H), 7.47 (dd, *J* = 5.1 Hz, 1.1 Hz, 1H), 7.34 (app t, *J* = 7.6 Hz, 1H), 7.18 (dd, *J* = 5.1 Hz, 3.7 Hz, 1H), 2.34 (s, 3H); ^{13}C NMR (101 MHz, $CDCl_3$): δ = 193.5, 136.7, 133.8, 132.7, 132.3, 129.4, 127.5, 127.2, 126.5, 125.6, 120.5, 120.2, 105.0, 19.8.; HRMS (EI+): calculated for $C_{14}H_{11}NOS_2$: 273.0282, found 273.0281 [M]: IR (ATR): ν/ cm^{-1} = 3344, 1670, 1592, 1354, 1212, 1169, 1084, 950, 851, 787, 743, 700.

3-(Methylthio)-1'-tosyl-1*H*,1'*H*-[2,3'-biindole]-7-carbaldehyde (5f)

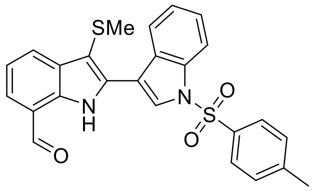

Following GP3 with alkynyl thioether **1o** (50.1 mg, 0.150 mmol), anthranil **4** (12.8 mg, 0.107 mmol); after reaction for 14 hours at 45 °C, column chromatography (SiO_2, 10% EtOAc in hexane to 15% EtOAc in hexane) afforded indole **5f** as an orange solid (30.6 mg, 62%). M.P: 172–175 °C; ^{1}H NMR (300 MHz, $CDCl_3$): δ = 10.43 (br s, 1H), 10.14 (s, 1H), 8.30 (s, 1H), 8.13 (dm, *J* = 7.8 Hz, 2H), 7.87 (t, *J* = 8.4 Hz, 3H), 7.74 (dd, *J* = 7.4 Hz, 1.0 Hz, 1H), 7.37–7.49 (m, 3H), 7.26 (d, *J* = 8.2 Hz, 2H), 2.37 (s, 3H), 2.30 (s, 3H); ^{13}C NMR (101 MHz, $CDCl_3$) δ 193.7, 145.5, 135.2, 135.1, 134.0, 131.8, 130.2, 129.3, 128.9, 127.2, 126.9, 126.7, 125.5, 124.4, 120.6, 120.4, 114.2, 113.0, 106.4, 21.8, 20.0. HRMS (ES+) calculated for $C_{25}H_{21}N_2O_3S_2$: 461.0988, found 461.0986 $[M+H]^+$; IR (ATR): ν/ cm^{-1} = 3646, 1663, 1606, 1593, 1505, 1490, 1445, 1367, 1329, 1299, 1343, 1223, 1173, 1145, 1104, 996, 812

Au (I) catalyzed reaction between alkynyl thioether 1a and trisubstituted isoxazoles 6a/b

2-(4-Methoxyphenyl)-5-methyl-3-(methylthio)-4-phenyl-1*H*-pyrrole (9) and 1-(2-(4-Methoxyphenyl)-5-methyl-4-phenyl-1*H*-pyrrol-3-yl)ethan-1-one (8b)

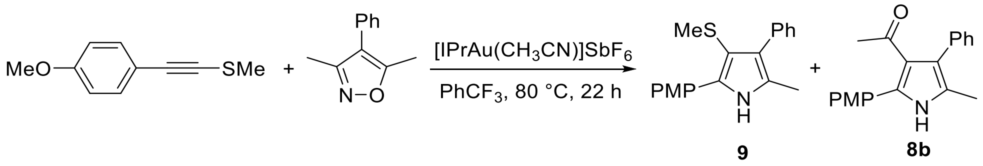

Following GP3 with alkynyl thioether **1a (**36.5 mg, 0.205 mmol), isoxazole **6b** (71.2 mg, 0.411 mmol); after reaction for 22 h, column chromatography (SiO_2, 0 to 20% EtOAc in hexane over 15 minutes) afforded **9** (16.6 mg, 27%) as a white gum, and **8b** (6.2 mg, 10%) as an off-white solid.

2-(4-Methoxyphenyl)-5-methyl-3-(methylthio)-4-phenyl-1*H*-pyrrole (9): ^{1}H NMR (400 MHz, $CDCl_3$): δ = 8.05 (br s, 1H), 7.65 (d, *J* = 8.2 Hz, 2H), 7.49–7.52 (m, 2H), 7.43 (app t, *J* = 7.6 Hz, 2H), 7.31 (tt, *J* = 7.3 Hz, 1.2 Hz, 1H), 6.98 (d, *J* = 8.2 Hz, 2H), 3.86 (s, 3H), 2.31 (br s, 3H), 1.91 (s, 3H); ^{13}C NMR (101 MHz, $CDCl_3$): δ = 158.8, 135.5, 132.4, 130.2, 128.6, 128.1, 126.2, 125.5, 125.4, 125.0, 114.1, 110.08, 55.4, 20.1, 12.3; HRMS (ES+) calculated for $C_{19}H_{19}NOS$: 310.1266, found 310.1267 [M+H]; IR (ATR): ν/ cm^{-1} = 3402, 2915, 2835, 1604, 1531, 1498, 1285, 1244, 1177, 1037, 1015, 829, 775, 737, 699.

1-(2-(4-Methoxyphenyl)-5-methyl-4-phenyl-1*H*-pyrrol-3-yl)ethan-1-one (8b): M.P.: 215–220 °C; ^{1}H NMR (400 MHz, $CDCl_3$): δ = 8.04 (br s, 1H), 7.49 (d, *J* = 8.7 Hz, 2H), 7.36–7.42 (m, 2H), 7.27–7.33 (m, 3H), 6.95 (d, *J* =8.9 Hz, 2H), 3.85 (s, 3H), 2.21 (s, 3H), 1.96 (s, 3H); ^{13}C NMR (101 MHz, $CDCl_3$): δ = 197.5, 159.8, 136.1, 133.8, 130.3, 130.2, 128.3, 126.6, 125.3, 125.1, 123. 0, 122.3, 114.0, 55.5, 31.3, 11.5; HRMS (ES+); calculated for $C_{20}H_{20}NO_2$: 306.1494, found 306.1496 [M+H]; IR (ATR): ν/ cm^{-1} = 3218, 1628, 1502, 1443, 1419, 1404, 1287, 1248, 1035, 953, 835, 780, 701.

(2-(4-Methoxyphenyl)-4-methyl-3-(methylthio)-5-phenyl-1*H*-pyrrol-1-yl)(phenyl)methanone (7) and (2-(4-Methoxyphenyl)-4-methyl-5-phenyl-1*H*-pyrrol-3-yl)(phenyl)methanone (8a)

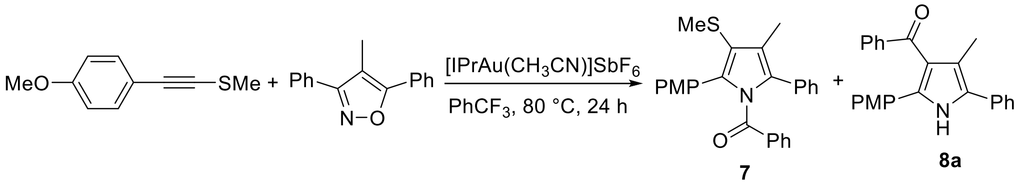

7

8a

Following GP3 with alkynyl thioether **1a** (71.0 mg, 0.398 mmol), isoxazole **6a** (189.4 mg, 0.8 mmol); after reaction for 24 h, column chromatography (SiO_2, Hexane/EtOAc gradient) afforded **7** and **8a** along with minor unidentified impurities. Subsequent crystallisation (layering, CH_2Cl_2:pentane) afforded **7** as a yellow solid (58.2 mg, 35%), and **8a** as a yellow crystalline solid (43 mg, 36%).

(2-(4-Methoxyphenyl)-4-methyl-3-(methylthio)-5-phenyl-1*H*-pyrrol-1-yl)(phenyl)methanone (7): M.P.: 116–118 °C; ^{1}H NMR (400 MHz, $CDCl_3$): δ = 7.48 (dd, *J* = 8.4 Hz, 1.2 Hz, 2H), 7.33 (tt, *J* = 7.5 Hz, 1.3 Hz, 1H), 7.21–7.27 (m, 6H), 7.13–7.20 (m, 3H), 6.77 (d, *J* = 8.5 Hz, 2H), 3.76 (s, 3H), 2.25 (s, 3H), 2.18 (s, 3H); ^{13}C NMR (101 MHz, $CDCl_3$): δ = 170.8, 159.1, 137.4, 134.9, 133.4, 132.4, 131.8, 130.5, 129.8, 128.2, 128.1, 127.2, 124.1, 122.6, 116.9, 113.4, 55.3, 19.8, 10.7; HRMS (ES+): calculated for $C_{26}H_{24}NO_2S$: 414.1528, found 414.1530 [M+H]; IR (ATR): ν/ cm^{-1} = 3003, 2924, 2834, 1700, 1609, 1596, 1529, 1463, 1489, 1450, 1301, 1280, 1247, 1176, 1029, 921, 912, 836, 773, 700.

(2-(4-Methoxyphenyl)-4-methyl-5-phenyl-1*H*-pyrrol-3-yl)(phenyl)methanone (8a): M.P.: 219–226 °C; ^{1}H NMR (400 MHz, $CDCl_3$): δ = 8.30 (s, 1H), 7.74 (dd, *J* = 8.4 Hz, 1.5 Hz, 2H), 7.52 (d, *J* = 8.6 Hz, 2H), 7.46 (app t, *J* = 7.5 Hz, 2H), 7.33–7.37 (m, 2H), 7.16–7.24 (m, 4H), 6.69 (d, *J* = 8.7 Hz, 2H), 3.72 (s, 3H), 2.29 (s, 3H); ^{13}C NMR (101 MHz, $CDCl_3$): δ = 194.5, 159.1, 139.4, 135.7, 132.6, 131.9, 129.9, 129.6, 129.3, 128.9, 127.9, 127.1, 126.9, 124.7, 121.9, 118.7, 113.9, 55.3, 11.6; HRMS (ES+): calculated for $C_{25}H_{22}NO_2$: 368.1651, found 368.1651 [M+H]; IR (ATR): ν/ cm^{-1} = 3319, 1600, 1574, 1494, 1458, 1446, 1406, 1244, 1173, 1039, 906, 832, 749, 700, 662.

Isotopic labelling study: Au (I) catalyzed reaction between ^{13}C-1l and 2a

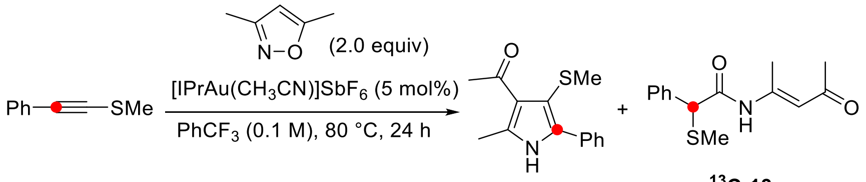

^{13}C-1l

^{13}C-3l

^{13}C-13

1-(2-Methyl-4-(methylthio)-5-phenyl-1*H*-pyrrol-3-yl-5-^{13}*C*)ethan-1-one (^{13}C-3l) and (*Z*)-2-(methylthio)-*N*-(4-oxopent-2-en-2-yl)-2-phenylacetamide-2-^{13}C (^{13}C-13).

Following GP3 with alkynyl thioether **^{13}C-1l** (30.0 mg, 0.201 mmol), isoxazole **2a** (39.0 mg, 0.402 mmol); after reaction for 24 h, column chromatography (SiO_2, 0 to 35% EtOAc in hexane)

afforded pyrrole **^{13}C-3l** as an off-white solid (35.6 mg, 72%), along with isotopically enriched *β*-ketoenamide **^{13}C-13** as a yellow liquid (5.6 mg, 10%).

1-(2-Methyl-4-(methylthio)-5-phenyl-1*H*-pyrrol-3-yl-5-^{13}*C*)ethan-1-one (^{13}C-3l): M.P.: 131–133 °C; ^{1}H NMR (400 MHz, $CDCl_3$): δ = 8.80 (s, 1H), 7.70–7.60 (m, 2H), 7.47–7.39 (m, 2H), 7.39–7.29 (m, 1H), 2.73 (s, 3H), 2.52 (s, 3H), 2.18 (s, 3H). ^{13}C NMR (101 MHz, $CDCl_3$): δ = 197.0, 135.9, 133.8, 131.8, 128.6, 127.9, 127.8, 124.4, 111.6, 30.5, 21.4, 14.8; HRMS (ES+) calculated for $^{12}C_{14}H_{16}NOS$: 246.0952, found 246.0953 [M+H], calculated for $^{12}C_{13}{}^{13}CH_{16}NOS$: 247.0986, found 247.0983; IR (ATR): ν/ cm^{-1} = 3185, 2924, 1629, 1556, 1473, 1444, 1419, 1219, 1140, 793, 767.

(*Z*)-2-(Methylthio)-*N*-(4-oxopent-2-en-2-yl)-2-phenylacetamide-2-^{13}C (^{13}C-13): ^{1}H NMR (400 MHz, $CDCl_3$): δ = 12.86 (s, 1H), 7.50–7.47 (m, 2H), 7.39–7.29 (m, 3H), 5.36 (d, *J* = 7.3 Hz, 1H), 4.51 (s, 0.8H), 4.51 (d, *J* = 142.4 Hz, 0.2 H, ^{13}C Satellites), 2.35 (d, *J* = 0.8 Hz, 3H), 2.14 (s, 3H), 2.12 (s, 3H); ^{13}C NMR (101 MHz, $CDCl_3$): δ = 199.8, 170.1, 154.7, 135.9, 129.0, 128.4(9), 128.4(7), 106.7, 58.2, 30.6, 21.9, 15.6; ; HRMS (ES+) calculated for $^{12}C_{14}H_{18}NO_2S$: 264.1053, found 264.1058, calculated for $^{12}C_{13}{}^{13}CH_{18}NO_2S$: 265.1092, found 265.1086 [M+H]; IR (ATR): ν/ cm^{-1} = 2919, 1702, 1645, 1590, 1474, 1249, 1129, 786, 732, 696.

Transformations of 3a

Synthesis of 1-(5-(4-methoxyphenyl)-2-methyl-4-(sulfonyl)-1*H*-pyrrol-3-yl)ethan-1-one (10)

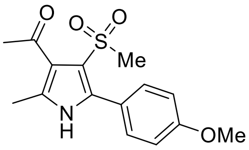

In a 1 dram vial was dissolved **3a** (40.4 mg, 0.147 mmol) in CH_2Cl_2 (1 mL). The resultant solution was cooled to 0 °C, and *m*-CPBA (≤ 77%, remainder H_2O) (74.1 mg, ≤ 0.33 mmol) was added portionwise over 10 minutes. The reaction was stirred at 0 °C for 1 hour, and then at room temperature for 5 hours. The reaction mixture was diluted with CH_2Cl_2 (10 mL), and washed with $NaHCO_3$ $_{(sat.\ aq.)}$ (3 × 10 mL); the organic layer was dried (Na_2SO_4), concentrated *in vacuo*, and the obtained residue purified by column chromatography (0 to 100% EtOAc in hexane) to yield sulfone **10** as a white solid (32.5 mg, 73%). M.P: 216–221 °C; ^{1}H NMR (400 MHz, DMSO-d_6): δ = 11.92 (br s, 1H), 7.42 (d, *J* = 8.8 Hz, 2H), 6.98 (d, *J* = 8.8 Hz, 2H), 3.80 (s, 3H), 3.27 (s, 3H), 2.44 (s, 3H), 2.38 (s, 3H); ^{13}C NMR (101 MHz, DMSO-d_6): δ = 195.0, 159.5, 135.7, 132.9, 131.4, 122.9, 122.3, 118.2, 113.2, 55.2, 45.6, 31.3, 12.9; HRMS (ES+): calculated for $C_{15}H_{18}NO_4S$: 308.0951, found 308.0948 $[M+H]^+$; IR (ATR): ν/ cm^{-1} = 3282, 1654, 1610, 1490, 1446, 1291, 1256, 1161, 1128, 1033, 966, 953, 842, 771, 635.

Synthesis of 1-(5-(4-methoxyphenyl)-2-methyl-4-(methylsulfinyl)-1*H*-pyrrol-3-yl)ethan-1-one (11)

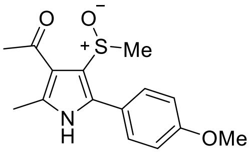

To a 1 dram vial was charged **3a** (136 mg, 0.494 mmol), $(CH_3)_2CO:H_2O$ (2:1, 1.2 mL) and MoO_2Cl_2 (1.5 mg, 7.5 µmol, 1.5 mol %). The mixture was stirred, and H_2O_2 (30% in H_2O)(55 µL, 0.533 mmol) was added dropwise. After reaction for 24 hours, the reaction mixture was diluted with brine (5 mL) and

extracted with EtOAc (3 × 5mL). Combined organic extracts were washed with brine (1 × 5 mL), dried (Na_2SO_4), and the solvent removed *in vacuo* to afford sulfoxide **11** as a white solid (70.8 mg, 49%). ^1H NMR (400 MHz, DMSO-d_6): δ = 11.85 (br s, 1H), 7.45 (d, *J* = 8.5 Hz, 2H), 6.99 (d, *J* = 8.5 Hz, 2H), 3.80 (s, 3H), 2.87 (s, 3H), 2.49 (s, 3H), 2.48 (s, 3H); ^{13}C NMR (101 MHz, DMSO-d_6): δ = 193.5, 159.2, 135.8, 133.1, 131.2, 123.0, 121.1, 120.9, 113.2, 55.2, 40.9, 30.8, 13.8; HRMS (ES+): calculated for $C_{15}H_{18}NO_3S$: 292.1007, found 292.1015 [M+H]; IR (ATR): ν/ cm^{-1} = 2975, 1629, 1530, 1491, 1450, 1398, 1273, 1242, 1182, 1014, 996, 961, 915, 830.

Synthesis of 1-(5-(4-methoxyphenyl)-2-methyl-1*H*-pyrrol-3-yl)ethan-1-one (12) from 11

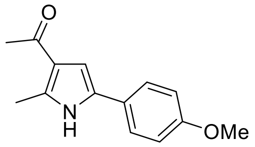

To a dry and degassed finger Schlenk flask was charged sulfoxide **11** (26.1 mg, 0.09 mmol), anhydrous CH_2Cl_2 (1 mL) and 2,6-lutidine (31 µL, 28.6 mg, 0.267 mmol, 3 equiv). The reaction mixture was cooled to –78 °C, and trifluoromethanesulfonic anhydride (36 µL, 60.5 mg, 0.214 mmol, 2.4 equiv) was added dropwise. After 30 minutes the reaction was allowed to warm to room temperature, whereupon it was stirred for 48 h. Upon careful quenching with $NaHCO_3$ $_{(sat.aq)}$ (2 mL), the reaction mixture was extracted with CH_2Cl_2 (2 × 5 mL); combined organic extracts were dried (Na_2SO_4) and the solvent removed *in vacuo*; column chromatography (SiO_2, 10% to 60% EtOAc in hexane) of the resulting residue afforded **12** as a white solid (17.3 mg, 84%). M.P: 170–172 °C; ^1H NMR (400 MHz, $CDCl_3$): δ = 8.50 (br s, 1H), 7.39 (dd, *J* = 8.6 Hz, 2H), 6.92 (d, *J* = 8.6 Hz, 2H), 6.65 (d, *J* = 2.9 Hz, 1H), 3.83 (s, 3H), 2.60 (s, 3H), 2.44 (s, 3H); ^{13}C NMR (101 MHz, $CDCl_3$): δ = 195.3, 158.8, 135.5, 130.0, 125.4, 124.8, 122.3, 114.6, 106.4, 55.5, 28.7, 14.2; HRMS (ES+); calculated for $C_{14}H_{16}NO_2$: 230.1181, found 230.1190 [M+H]; IR (ATR): ν/ cm^{-1} = 3256, 1636, 1596, 1573, 1530, 1430, 1245, 1187, 1030, 951, 933, 830, 795.

Synthesis of 1-(5-(4-methoxyphenyl)-2-methyl-1*H*-pyrrol-3-yl)ethan-1-one (12) from 3d

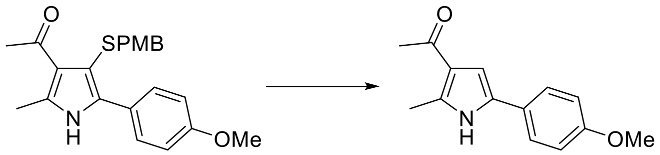

To a 25 mL round bottom flask was charged **3d** (199 mg, 0.521 mmol), trifluoroacetic acid (6 mL) and anisole (1 mL). The reaction mixture was stirred for 16 hours at 40 °C. The reaction mixture was poured into an ice cold saturated solution of Na_2CO_3, and extracted with CH_2Cl_2 (3 × 50 mL). Organic extracts were washed with Na_2CO_3 $_{(sat\ aq.)}$, brine and dried over Na_2SO_4; following removal of the solvent *in vacuo*, the resulting residue was subject to column chromatography (SiO_2, 0 to 100% EtOAc in hexane) to afford **12** as a brown solid (63.9 mg, 53%), with analytical data matching that of **12** obtained previously.

Crystallographic data

The datasets for **3j** and **8a** were measured on an Agilent SuperNova diffractometer using an Atlas detector. The data collections were driven and processed and absorption corrections were applied using CrysAlisPro.[31] The structures were solved using ShelXT[32] and were refined by a full-matrix least-squares procedure on F^2 in ShelXL.[33] Figures and reports were produced using OLEX2.[34] All non-hydrogen atoms were refined with anisotropic displacement parameters. In both structures, the hydrogen atom bonded to N(1) was located in the electron density and freely refined. All remaining hydrogen atoms were fixed as riding models and the isotropic thermal parameters (U_{iso}) were based on the U_{eq} of the parent atoms.

Crystal data for **3j**: $C_{17}H_{21}NOS$ (M =287.41 g/mol): orthorhombic, space group $P2_12_12_1$ (no. 19), a = 6.7593(3) Å, b = 11.1929(4) Å, c = 20.8815(9) Å, V = 1579.81(12) $Å^3$, Z = 4, T = 100.01(10) K, μ(CuKα) = 1.770 mm^{-1}, $Dcalc$ = 1.208 g/cm^3, 14508 reflections measured (8.468° ≤ 2Θ ≤ 149.562°), 3135 unique (R_{int} = 0.0414, R_{sigma} = 0.0370) which were used in all calculations. The final R_1 was 0.0401 (I > 2σ(I)) and wR_2 was 0.0973 (all data). The structure occupies a chiral space group and has been refined as an inversion twin with the refined ratio of enantiomers being 72 (3) : 28 (3).

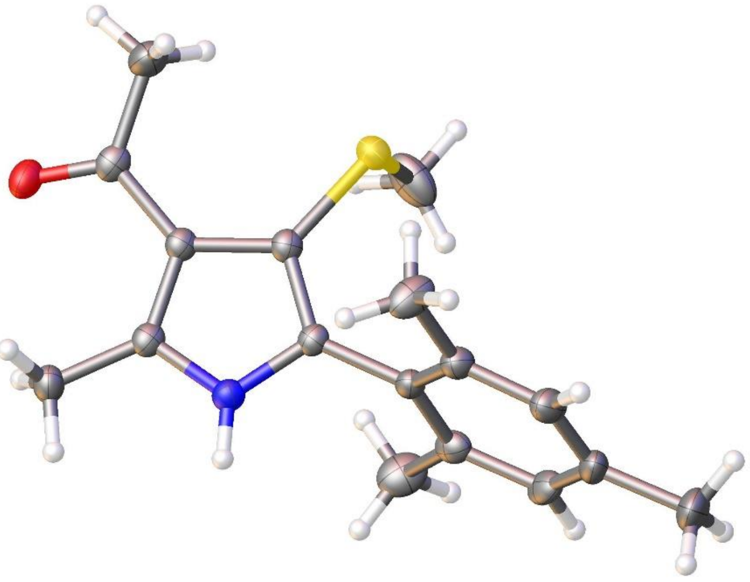

Figure 1 *Crystal structure of **3j** with ellipsoids drawn at the 50 % probability level.*

Crystal data for **8a**: $C_{25}H_{21}NO_2$ (M =367.43 g/mol): monoclinic, space group Cc (no. 9), a = 19.2387(9) Å, b = 12.2869(2) Å, c = 10.5907(5) Å, β = 130.407(7)°, V = 1906.3(2) $Å^3$, Z = 4, T = 100.01(10) K, μ(CuKα) = 0.638 mm^{-1}, $Dcalc$ = 1.280 g/cm^3, 17791 reflections measured (9.394° ≤ 2Θ ≤ 148.672°), 3672 unique (R_{int} = 0.0294, R_{sigma} = 0.0191) which were used in all calculations. The final R_1 was 0.0279 (I > 2σ(I)) and wR_2 was 0.0708 (all data).

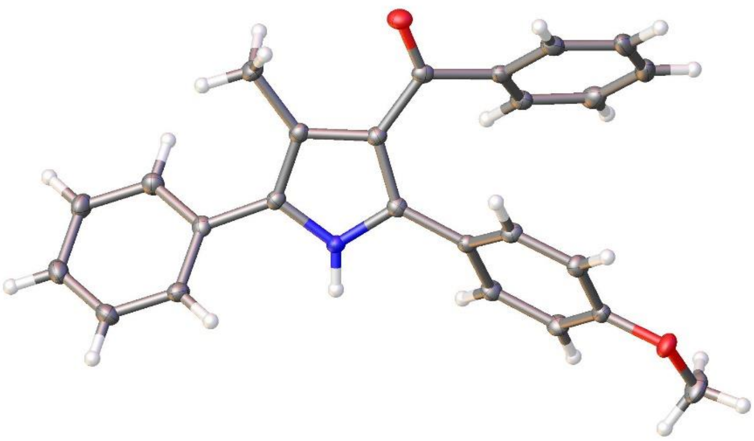

Figure 2 Crystal structure of 8a with ellipsoids drawn at the 50 % probability level.

CCDC 2033353 and CCDC 2033354 contain the supplementary crystallographic data for this paper. These data can be obtained free of charge from The Cambridge Crystallographic Data Centre via www.ccdc.cam.ac.uk/data_request/cif.

References

(1) Reddy, R. J.; Ball-Jones, M. P.; Davies, P. W. Alkynyl Thioethers in Gold-Catalyzed Annulations To Form Oxazoles. *Angew. Chem. Int. Ed*. **2017**, *56*, 13310–13313.

(2) Barrett, M. J.; Khan, G. F.; Davies, P. W.; Grainger, R. S. Alkynyl Sulfoxides as α-Sulfinyl Carbene Equivalents: Gold-Catalysed Oxidative Cyclopropanation. *Chem. Commun.* **2017**, *53*, 5733–5736.

(3) Zhou, N.; Wang, L.; Thompson, D. W.; Zhao, Y. OPE/OPV H-mers: Synthesis, Electronic Properties, and Spectroscopic Responses to Binding with Transition Metal Ions. *Tetrahedron* **2011**, *67*, 125–143.

(4) González-Cantalapiedra, E.; de Frutos, Ó.; Atienza, C.; Mateo, C.; Echavarren, A. M. Synthesis of the Benzo[b]fluorene Core of the Kinamycins by Arylalkyne–Allene and Arylalkyne–Alkyne Cycloadditions. *Eur. J. Org. Chem.* **2006**, *6*, 1430–1443.

(5) Benanti, T. L.; Saejueng, P.; Venkataraman, D. Segregated Assemblies in Bridged Electron-rich and Electron-poor π-Conjugated Moieties. *Chem. Commun.* **2007**, 692–694.

(6) Everett, R. K.; Wolfe, J. P. Synthesis of Substituted 3-Hydroxy-2-Furanone Derivatives via an Unusual Enolate Wittig Rearrangement/Alkylative Cyclization Sequence. *Org. Lett.* **2013**, *15*, 2926–2929.

(7) Urner, L. M.; Sekita, M.; Trapp, N.; Schweizer, W. B.; Wörle, M.; Gisselbrecht, J.-P.; Boudon, C.; Guldi, D. M.; Diederich, F. Systematic Variation of Cyanobuta-1,3-dienes and Expanded Tetracyanoquinodimethane Analogues as Electron Acceptors in Photoactive, Rigid Porphyrin Conjugates. *Eur. J. Org. Chem.* **2015**, 91–108.

(8) Wu, R.; Schumm, J. S.; Pearson, D. L.; Tour, J. M. Convergent Synthetic Routes to Orthogonally Fused Conjugated Oligomers Directed toward Molecular Scale Electronic Device Applications. *J. Org. Chem.* **1996**, *61*, 6906–6921.

(9) Abrams, M. L.; Foarta, F.; Landis, C. R. Asymmetric Hydroformylation of Z-Enamides and Enol Esters with Rhodium-Bisdiazaphos Catalysts. *J. Am. Chem. Soc.* **2014**, *136*, 14583–14588.

(10) Fujiki, K.; Tanifuji, N.; Sasaki, Y.; Yokoyama, T. New and Facile Synthesis of Thiosulfonates from Sulfinate/Disulfide/I_2 System. *Synthesis* **2002**, *3*, 0343–0348.

(11) Kaschula, C. H.; Hunter, R.; Stellenboom, N.; Caira, M. R.; Winks, S.; Ogunleye, T.; Richards, P.; Cotton, J.; Zilbeyaz, K.; Wang, Y.; Siyo, V.; Ngarande, E.; Parker, M. I. Structure–Activity Studies on the Anti-Proliferation Activity of Ajoene Analogues in WHCO1 Oesophageal Cancer Cells. *Eur. J. Med. Chem.* **2012**, *50*, 236–254.

(12) Reddy, R. J.; Waheeda, Md.; Krishna, G. R. *Org. Biomol. Chem.* **2020**, *18*, 3243–3248.

(13) Kim, W. G.; Baek, S.-y.; Jeong, S. Y.; Nam, D.; Jeon, J. H.; Choe, W.; Baik, M.-H.; Hong, S. Y. Chemo- and Regioselective Click Reactions through Nickel-Catalyzed Azide–Alkyne Cycloaddition. *Org. Biomol. Chem.* **2020**, *18*, 3374–3381.

(14) Santandrea, J.; Minozzi, C.; Cruché, C.; Collins, S. K. Photochemical Dual-Catalytic Synthesis of Alkynyl Sulfides. *Angew. Chem. Int. Ed.* **2017**, *56*, 12255–12259.

(15) Aurelio, L.; Volpe, R.; Halim, R.; Scammells, P. J.; Flynn, B. L. Synthesis of Thieno-Fused Heterocycles through Reiterative Iodocyclization. *Adv. Synth. Catal.* **2014**, *356*, 1974–1978.

(16) Su, Q.; Zhao, Z.-J.; Xu, F.; Lou, P.-C.; Zhang, K.; Xie, D.-X.; Shi, L.; Cai, Q.-Y.; Peng, Z.-H.; An, D.-L. One-Pot Preparation of Arylethynyl Sulfides and Bis(arylethynyl) Sulfides. *Eur. J. Org. Chem.* **2013**, *8*, 1551–1557.

(17) Melzig, L.; Stemper, J.; Knochel, P. A Novel Palladium-Catalyzed Cross-Coupling of Thiomethylated Alkynes with Functionalized Organozinc Reagents. *Synthesis* **2010**, *12*, 2085–2091.

(18) Braga, A. L.; Comasseto, J. V.; Petragnani, N. Pyrolysis of α-Acyl,α,-Thio Phosphoranes → Thioacetylenes. *Tetrahedron Lett.* **1984**, *25*, 1111–1114.

(19) Xie, L.-G.; Shaaban, S.; Chen, X.; Maulide, N. Metal-Free Synthesis of Highly Substituted Pyridines by Formal [2 + 2 + 2] Cycloaddition under Mild Conditions. *Angew. Chem. Int. Ed.* **2016**, *55*, 12864–12867.

(20) Zheng, W.; Zheng, F.; Hong, Y.; Hu, L. A one-pot synthesis of alkynyl sulfides from terminal alkynes. *Heteroat. Chem.* **2012**, *23*, 105–110.

(21) Miller, J. A.; Pugh, A. W.; Ullah, G. M.; Welsh, G. M. Synthesis and Anti-fungal Properties of Some Simple Cyclopentenones and Derivatives. *Tetrahedron Lett.* **2001**, *42*, 955–959.

(22) Takikawa, H.; Takada, A.; Hikita, K.; Suzuki, K. Formation of α-Hydroxy-β-diketones through Hydroxylation of Isoxazolium Salts: Stereoselective Approach to Angular cis-Diols in Polycyclic Systems. *Angew. Chem. Int. Ed.* **2008**, *47*, 7446–7449.

(23) Hansen, T. V.; Wu, P.; Fokin, V. V. One-Pot Copper(I)-Catalyzed Synthesis of 3,5-Disubstituted Isoxazoles. *J. Org. Chem.* **2005**, *70*, 7761–7764.

(24) Himo, F.; Lovell, T.; Hilgraf, R.; Rostovtsev, V. V.; Noodleman, L.; Sharpless, K. B.; Fokin, V. V. Copper(I)-Catalyzed Synthesis of Azoles. DFT Study Predicts Unprecedented Reactivity and Intermediates. *J. Am. Chem. Soc.* **2005**, *127*, 210–216.

(25) Lei, X.; Li, L.; He, Y.-P.; Tang, Y. Rhodium(II)-Catalyzed Formal [3 + 2] Cycloaddition of N-Sulfonyl-1,2,3-triazoles with Isoxazoles: Entry to Polysubstituted 3-Aminopyrroles. *Org. Lett.* **2015**, *17*, 5224–5227.

(26) Jawalekar, A. M.; Reubsaet, E.; Rutjes, F. P. J. T.; van Delft, F. L. Synthesis of Isoxazoles by Hypervalent Iodine-Induced Cycloaddition of Nitrile Oxides to Alkynes. *Chem. Commun.* **2011**, *47*, 3198–3200.

(27) Zimmermann, L. A.; de Moraes, M. H.; da Rosa, R.; de Melo, E. B.; Paula, F. R.; Schenkel, E. P.; Steindel, M.; Bernardes, L. S. C. Synthesis and SAR of New Isoxazole-Triazole bis-Heterocyclic Compounds as Analogues of Natural Lignans with Antiparasitic Activity. *Bioorg. Med. Chem.* **2018**, *26*, 4850–4862.

(28) Ueda, M.; Ikeda, Y.; Sato, A.; Ito, Y.; Kakiuchi, M.; Shono, H.; Miyoshi, T.; Naito, T.; Miyata, O. Silver-catalyzed Synthesis of Disubstituted Isoxazoles by Cyclization of Alkynyl Oxime Ethers. *Tetrahedron* **2011**, *67*, 4612–4615.

(29) Felix, C. P.; Khatimi, N.; Laurent, A. J. Reduction of 5-(Trifluoromethyl)isoxazoles with Lithium Aluminum Hydride: Synthesis of (2,2,2-Trifluoroethyl)aziridines. *J. Org. Chem.* **1995**, *60*, 3907–3909.

(30) Hewings, D. S.; Wang, M.; Philpott, M.; Fedorov, O.; Uttarkar, S.; Filippakopoulos, P.; Picaud, S.; Vuppusetty, C.; Marsden, B.; Knapp, S.; Conway, S. J.; Heightman, T. D. 3,5-Dimethylisoxazoles Act As Acetyl-lysine-mimetic Bromodomain Ligands. *J. Med. Chem.* **2011**, *54*, 6761–6770.

(31) CrysAlisPro, Rigaku Oxford Diffraction, 2015.

(32) Sheldrick, G. M. Integrated space-group and crystal-structure determination. *Acta Cryst.* **2015**, *A71*, 3-8.

(33) Sheldrick, G. M. Crystal structure refinement with SHELXL. *Acta Cryst.* **2015**, *C71*, 3-8.

(34) Dolomanov, O. V.; Bourhis, L. J.; Gildea, R. J.; Howard, J. A. K.; Puschmann, H. OLEX2: a complete structure solution, refinement and analysis program. *J. Appl. Crystallogr.* **2009**, *42*, 339-341.

^{1}H NMR and ^{13}C NMR Spectra

Benzyl((4-methoxyphenyl)ethynyl)sulfane (1c) in $CDCl_3$ ^{1}H-NMR and ^{13}C-NMR

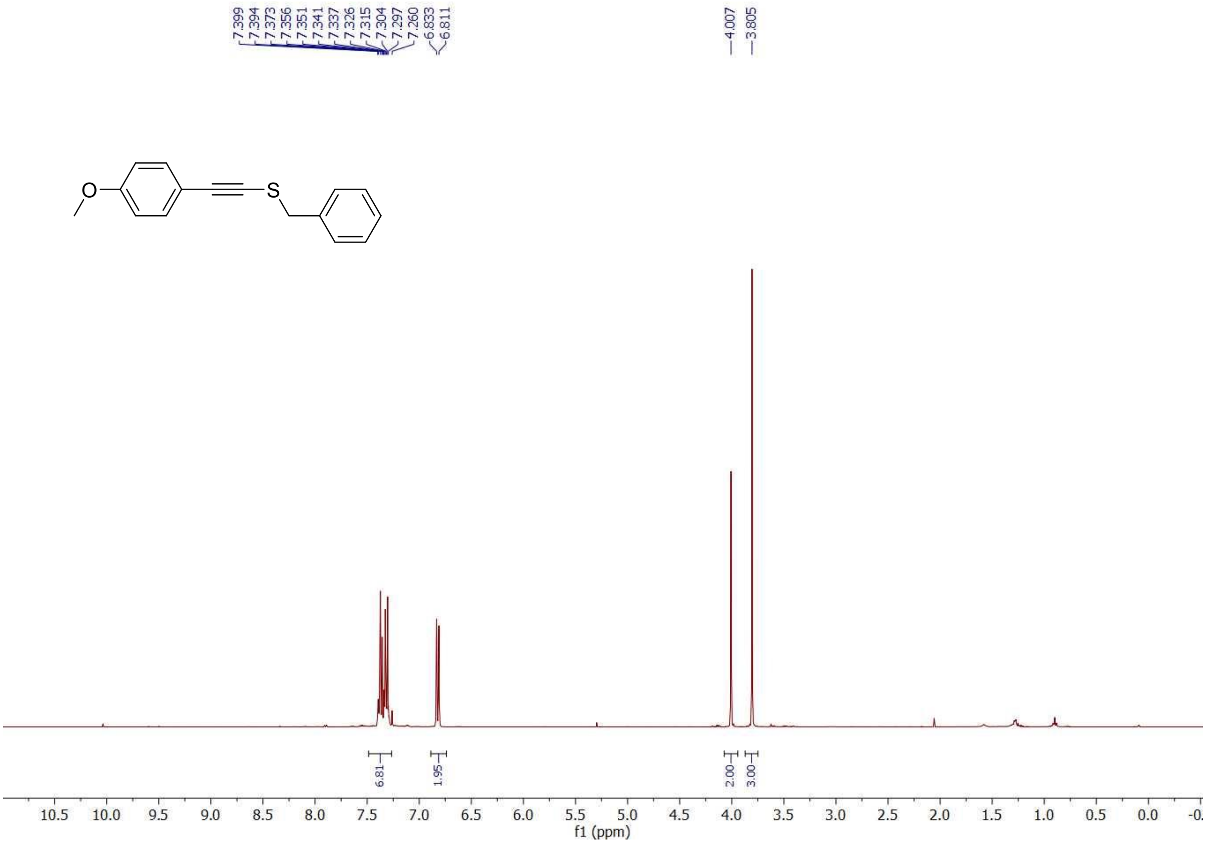

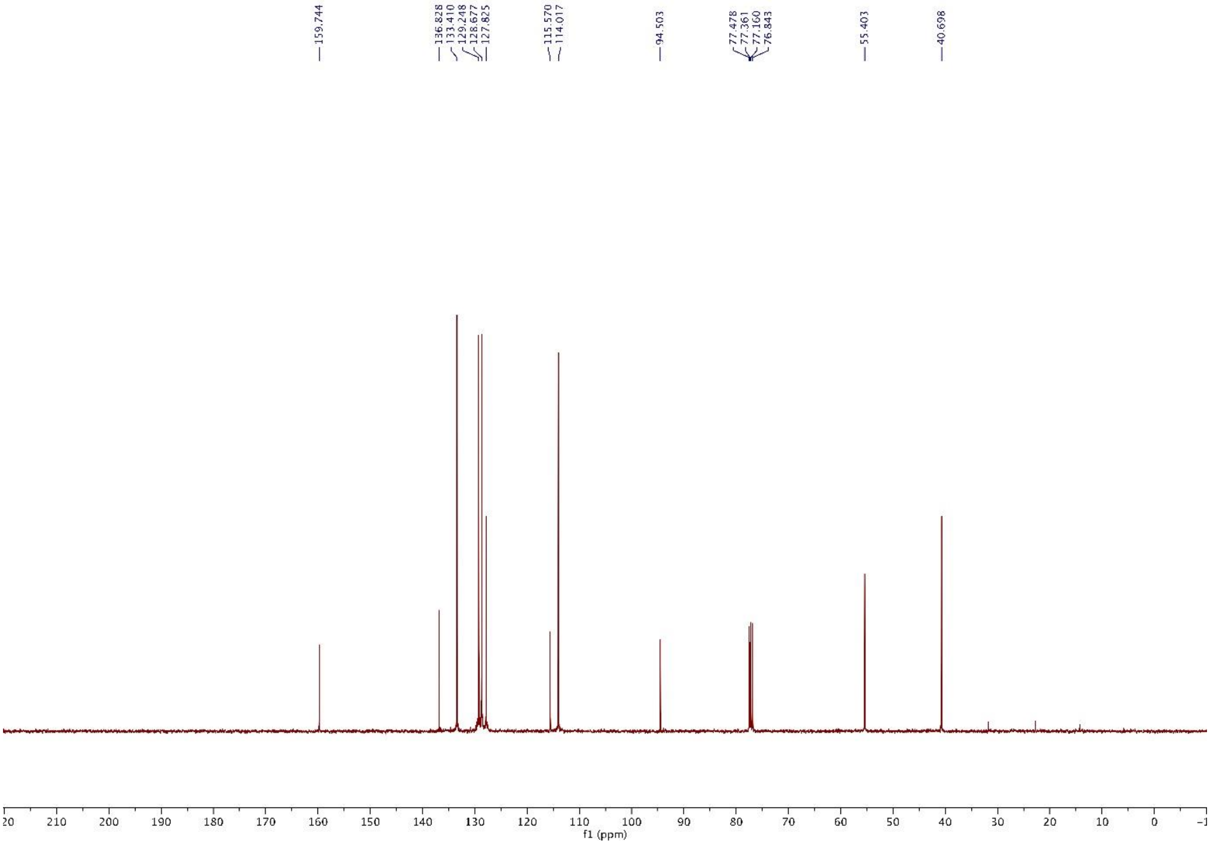

((4-Methoxybenzyl)((4-methoxyphenyl)ethynyl)sulfane (1d) in $CDCl_3$ ^1H-NMR and ^{13}C-NMR

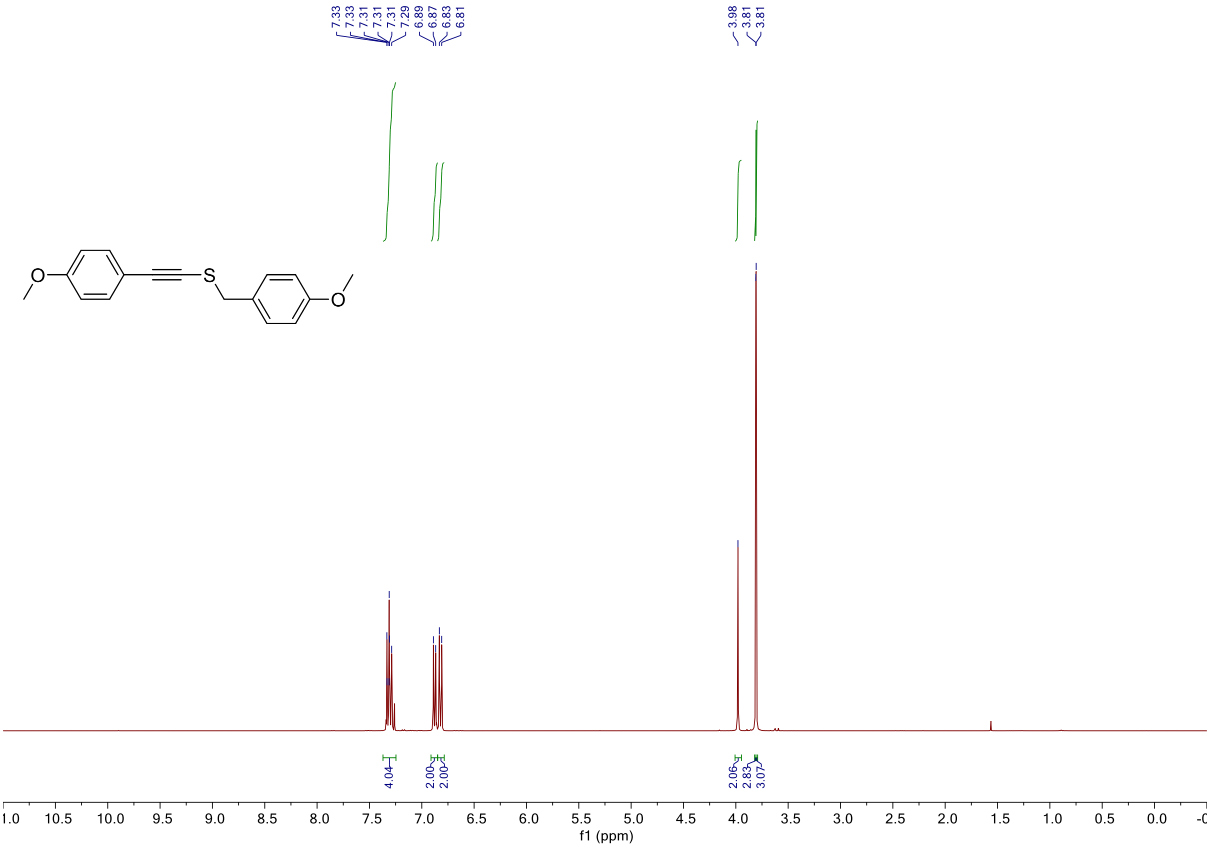

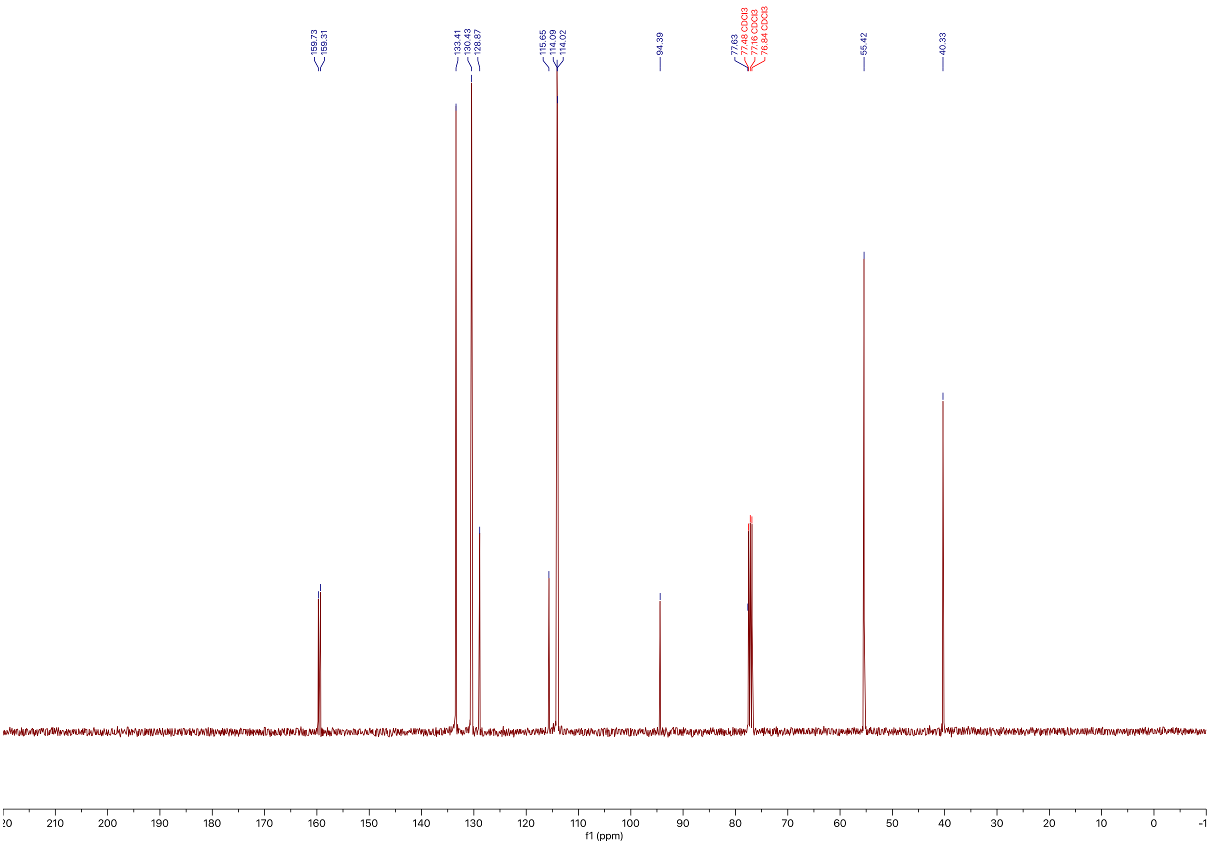

S-((4-Methoxyphenyl)ethynyl) ethanethioate (1f) in $CDCl_3$ ^1H NMR and ^{13}C NMR

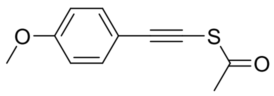

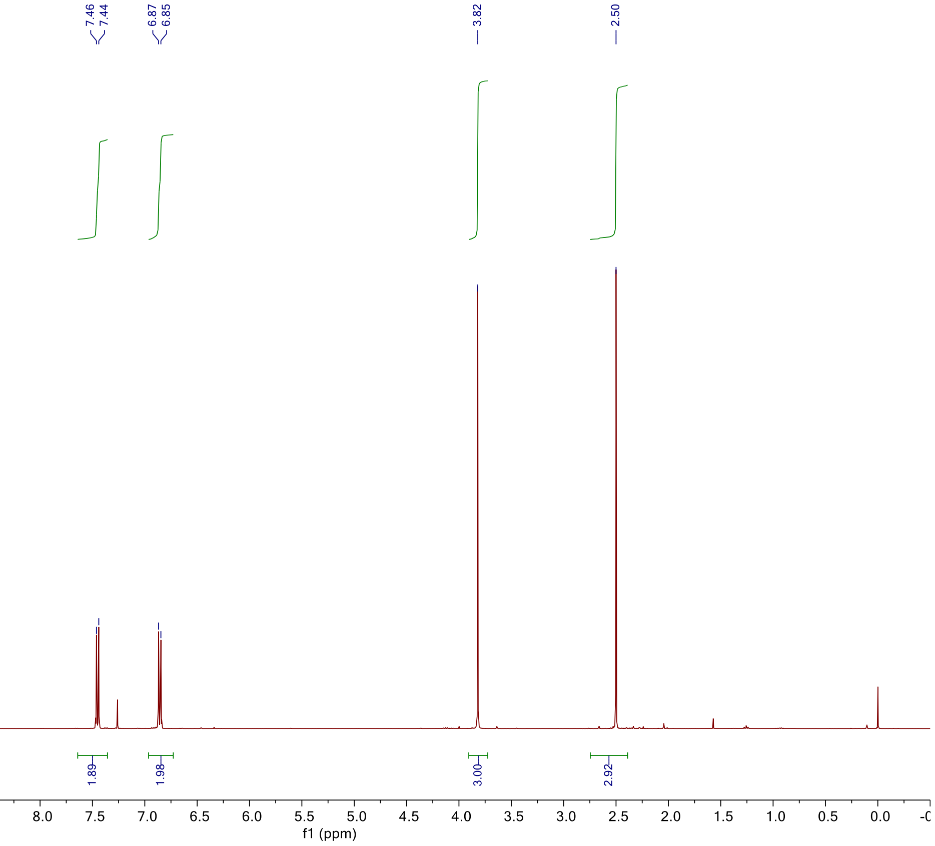

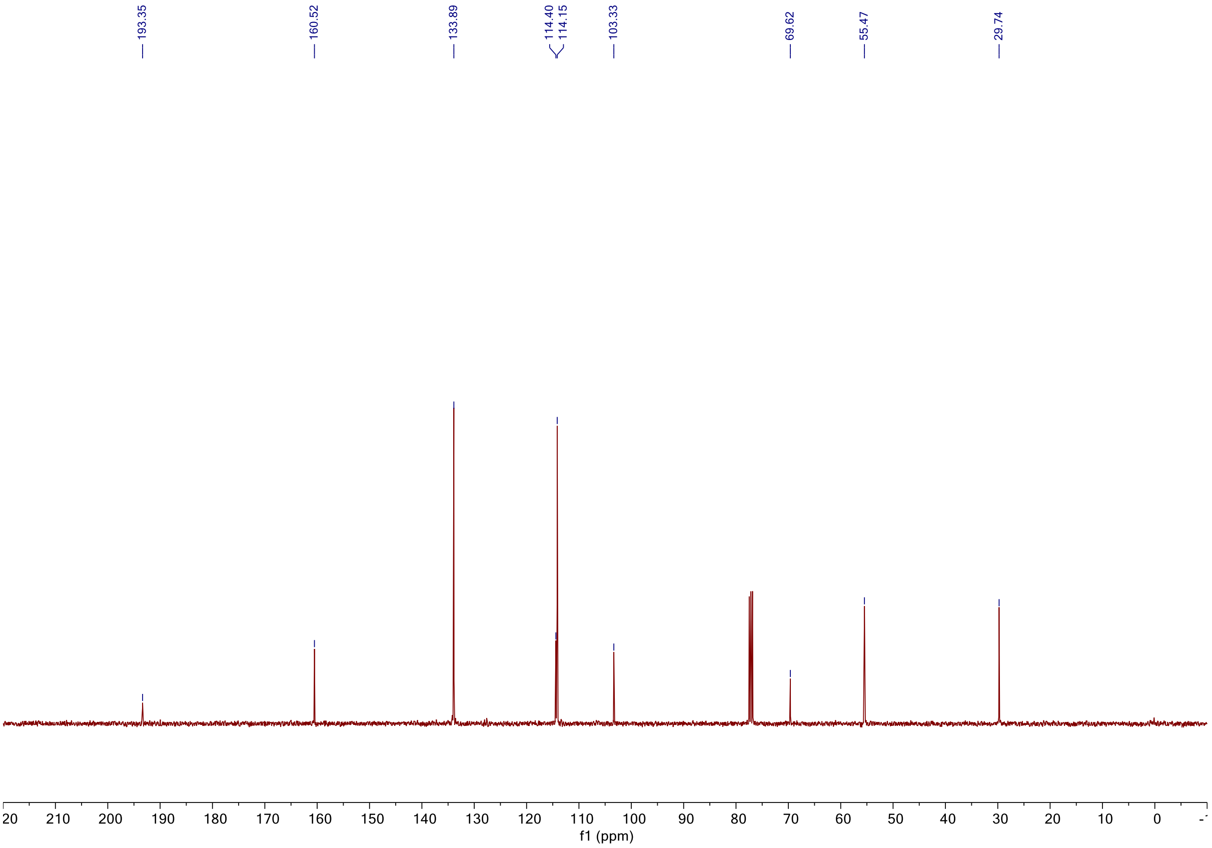

Ethyl 2-(((4-methoxyphenyl)ethynyl)thio)acetate (1g) in $CDCl_3$ ^{1}H-NMR and ^{13}C-NMR

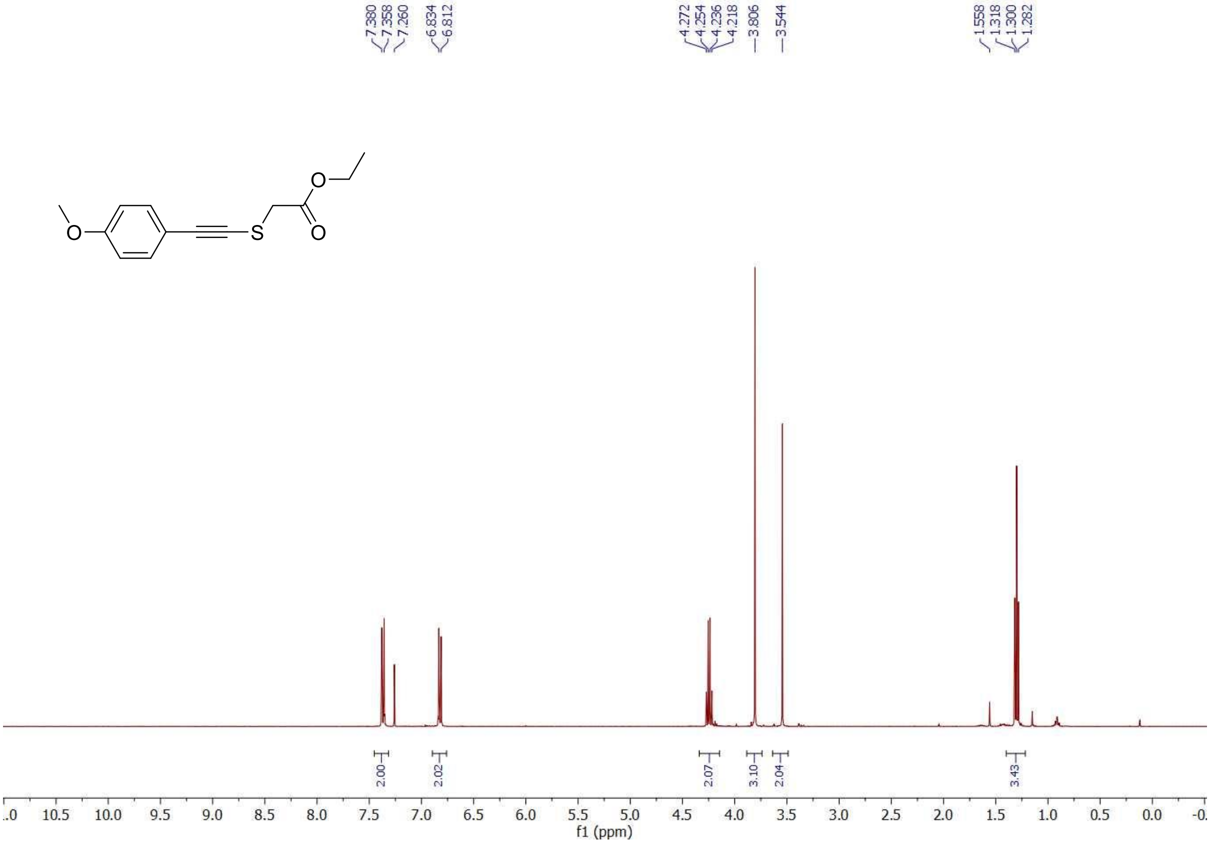

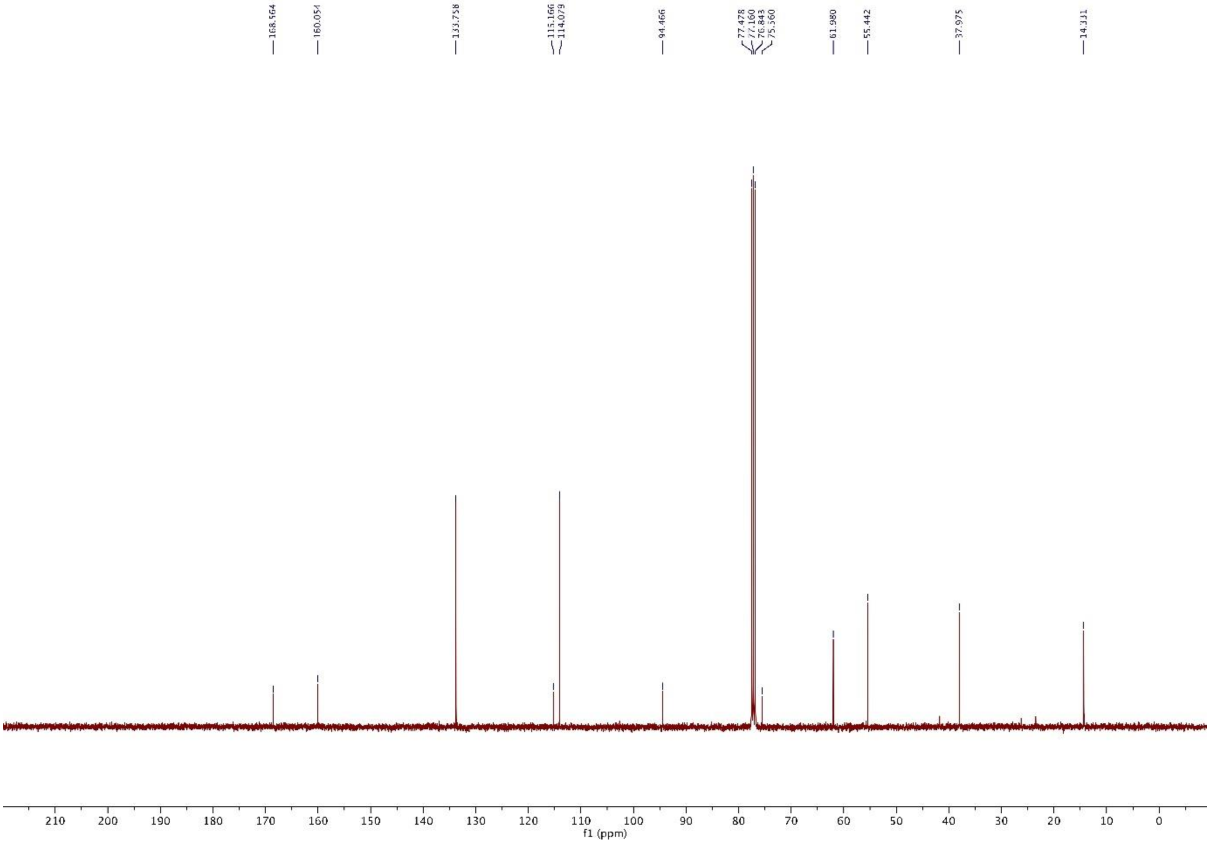

(2-Methoxyphenyl)ethynyl)(methyl)sulfane (1h) in $CDCl_3$ ^{1}H-NMR and ^{13}C-NMR

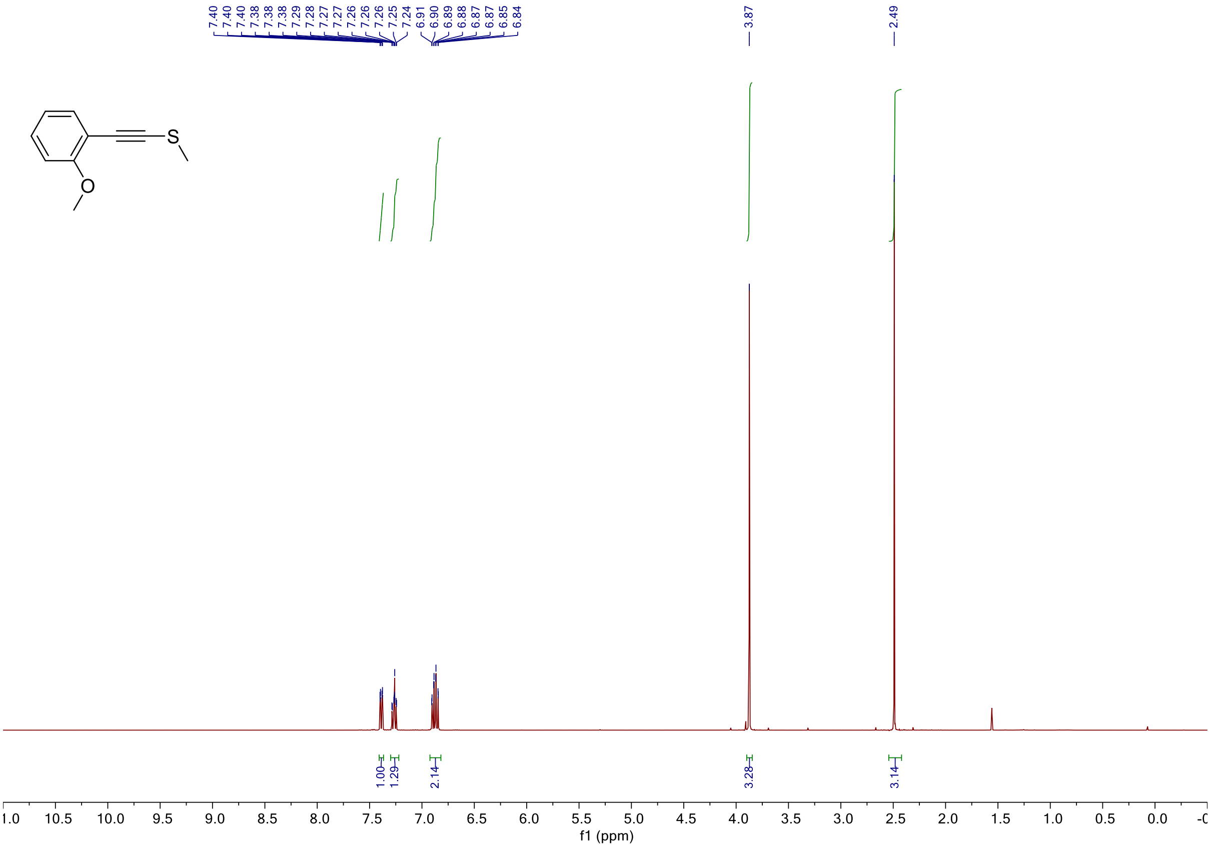

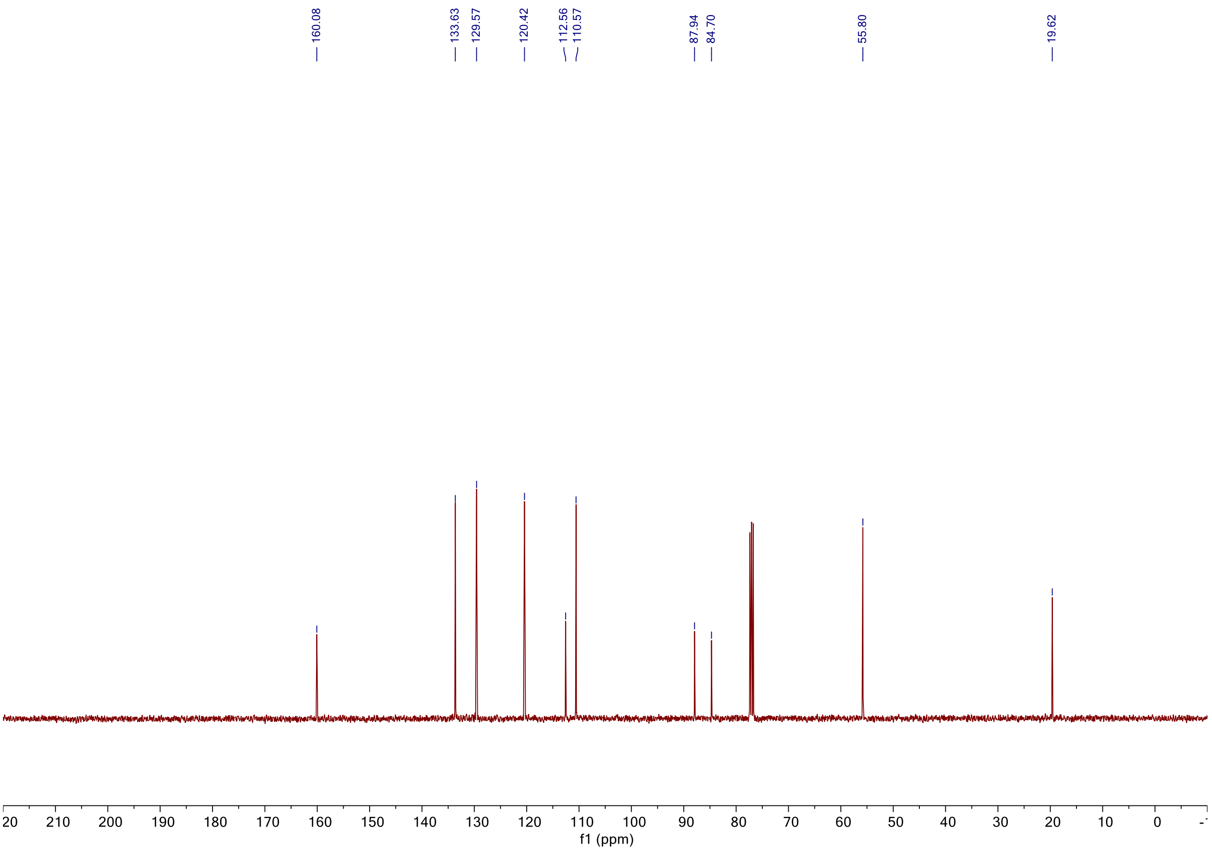

Ethyl 2-(((2-methoxyphenyl)ethynyl)thio)acetate (1i) in $CDCl_3$ ^1H-NMR and ^{13}C-NMR

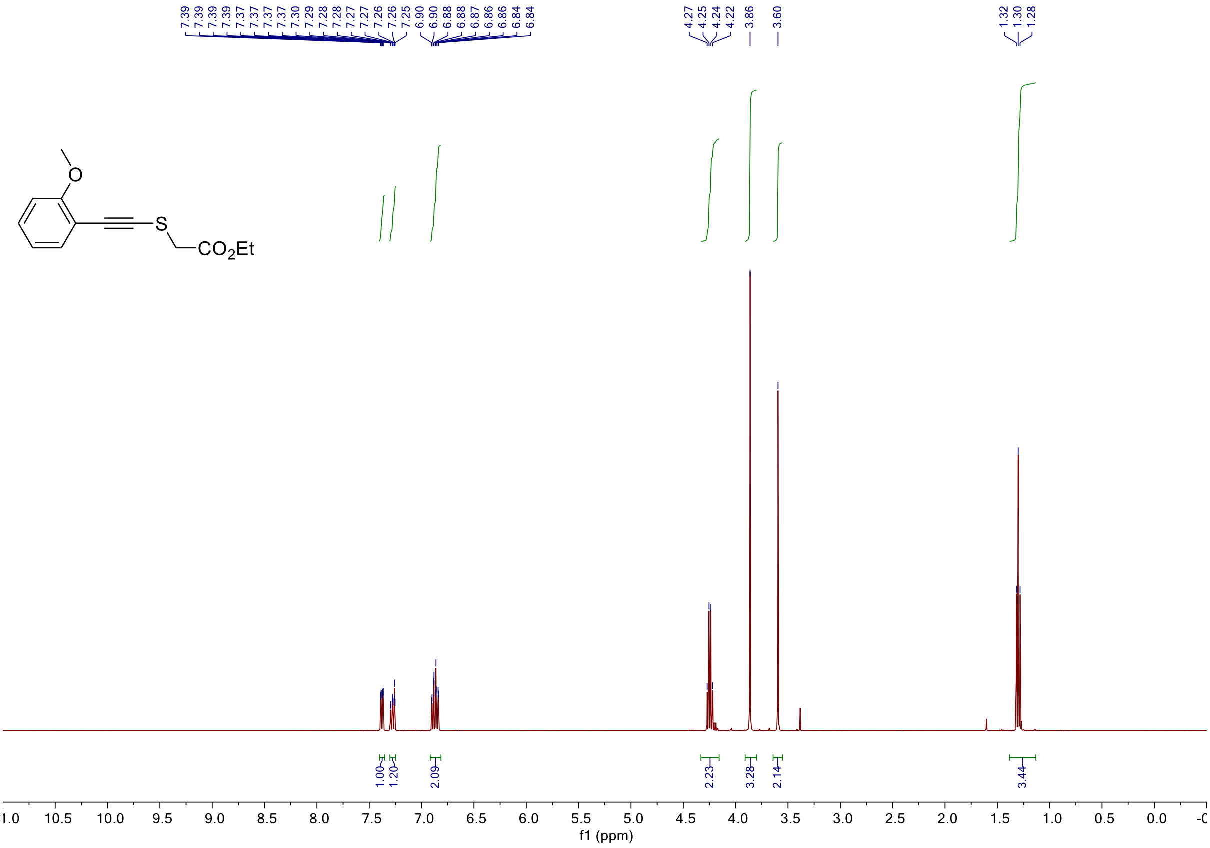

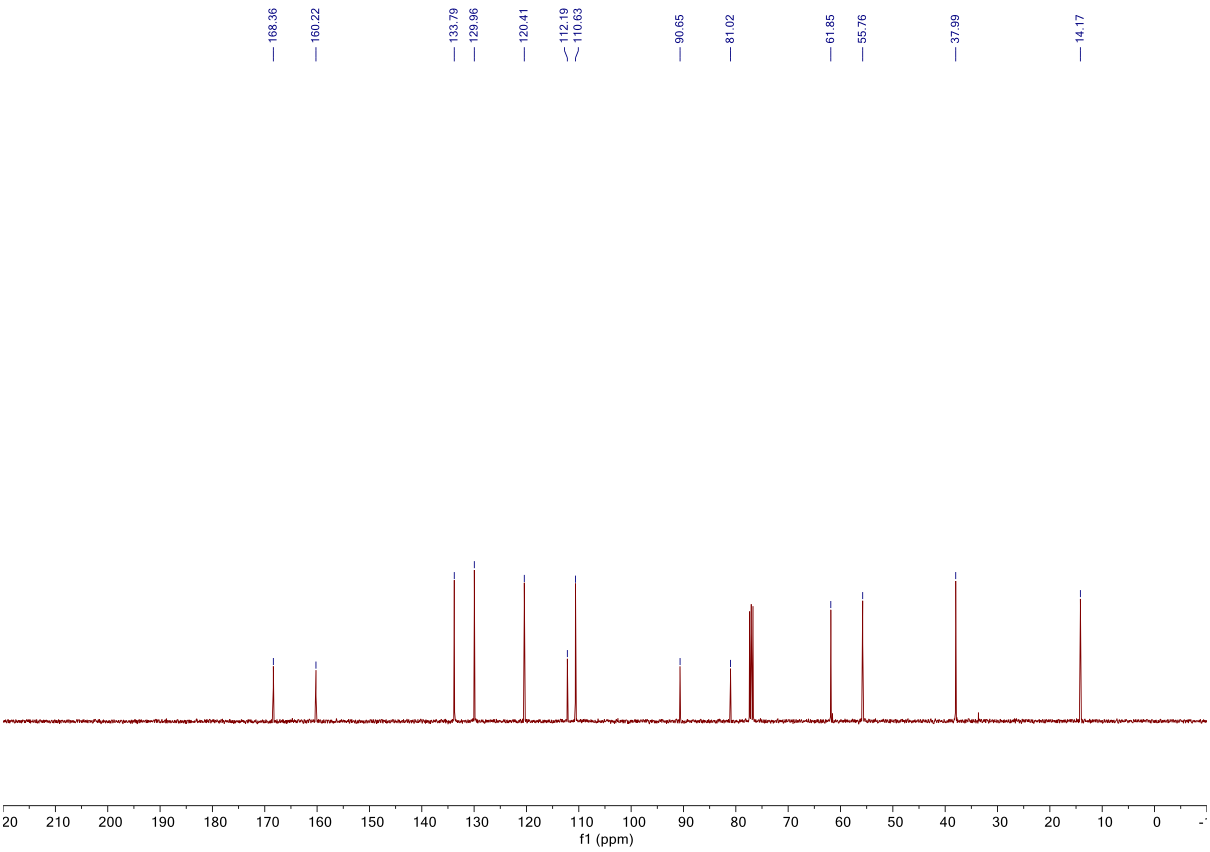

Methyl(naphthalen-2-ylethynyl)sulfane (1k) in $CDCl_3$ ^{1}H-NMR and ^{13}C-NMR

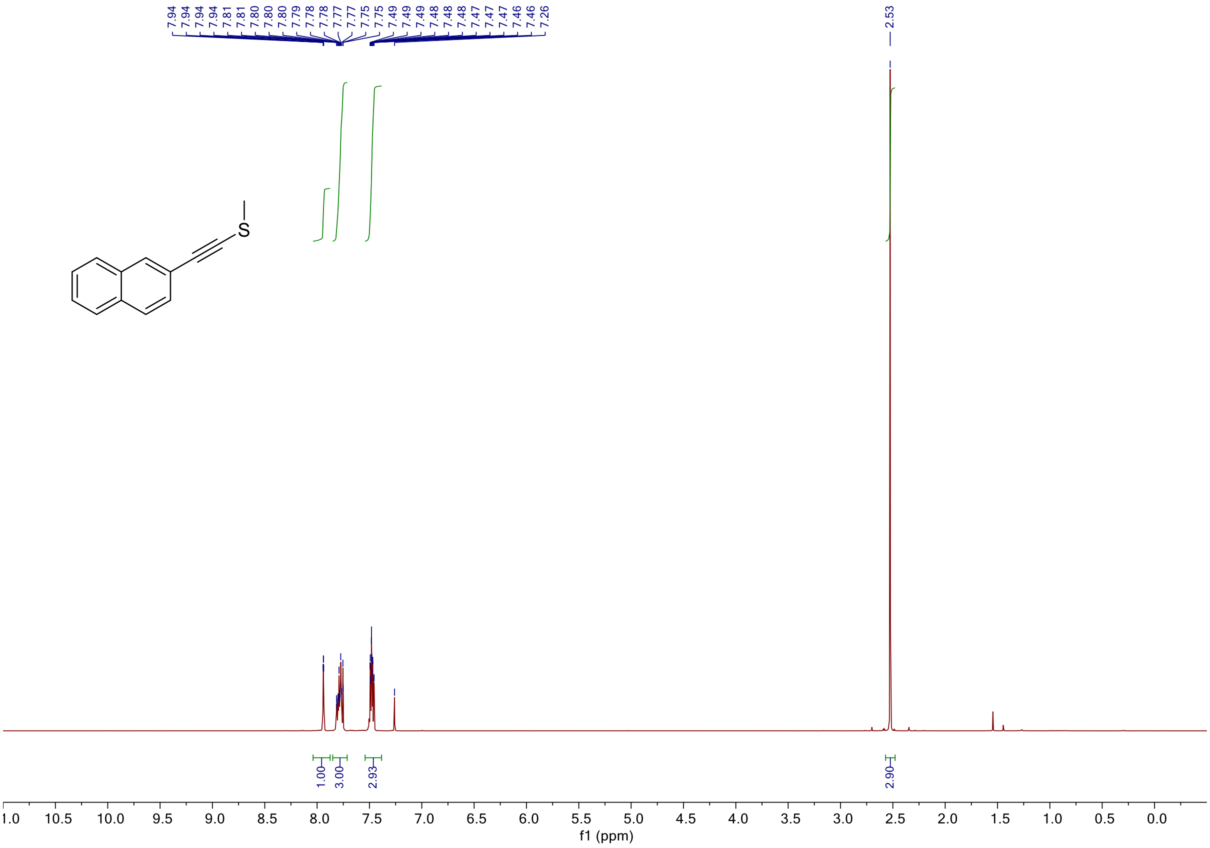

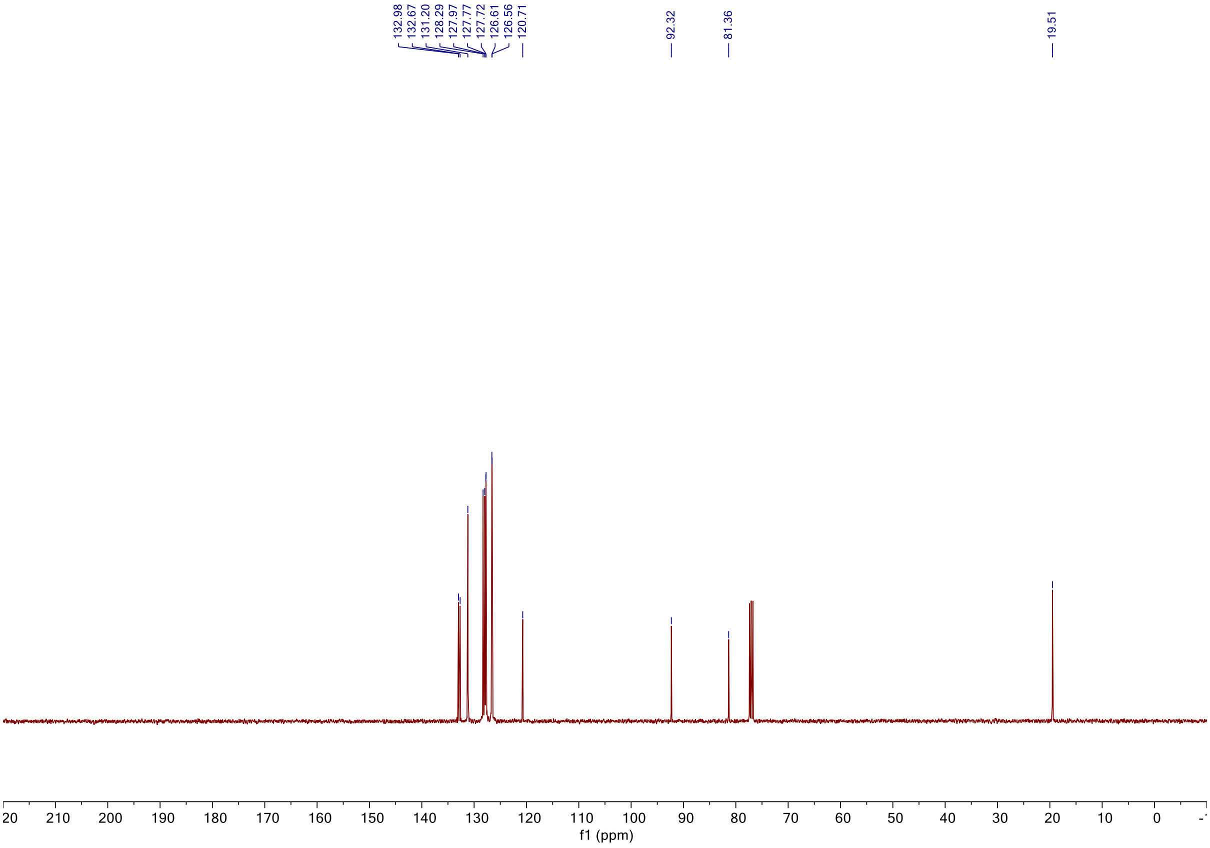

((4-Fluorophenyl)ethynyl)(methyl)sulfane (1m) in $CDCl_3$ ^{1}H-NMR and ^{13}C-NMR

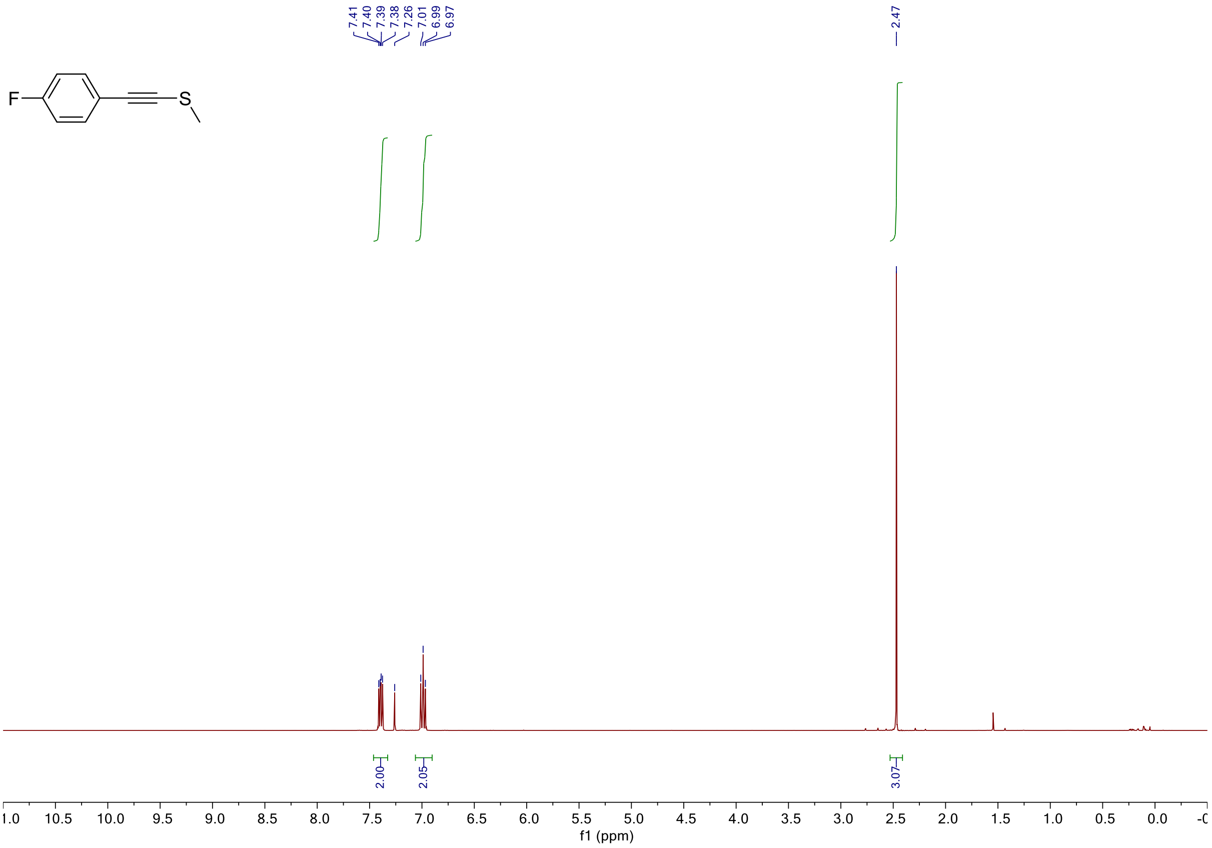

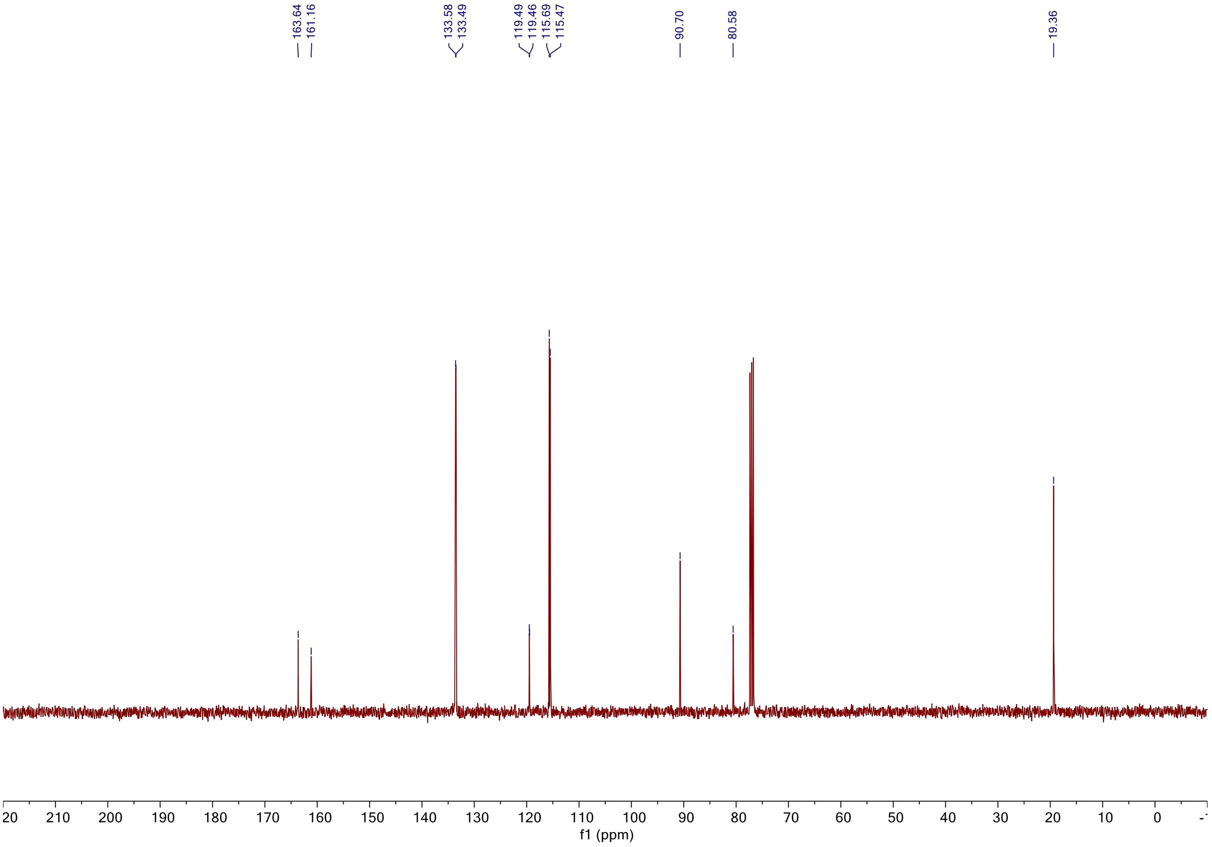

_N,N_-Dimethyl-4-((methylthio)ethynyl)aniline (1n) in $CDCl_3$ ^{1}H-NMR and ^{13}C-NMR

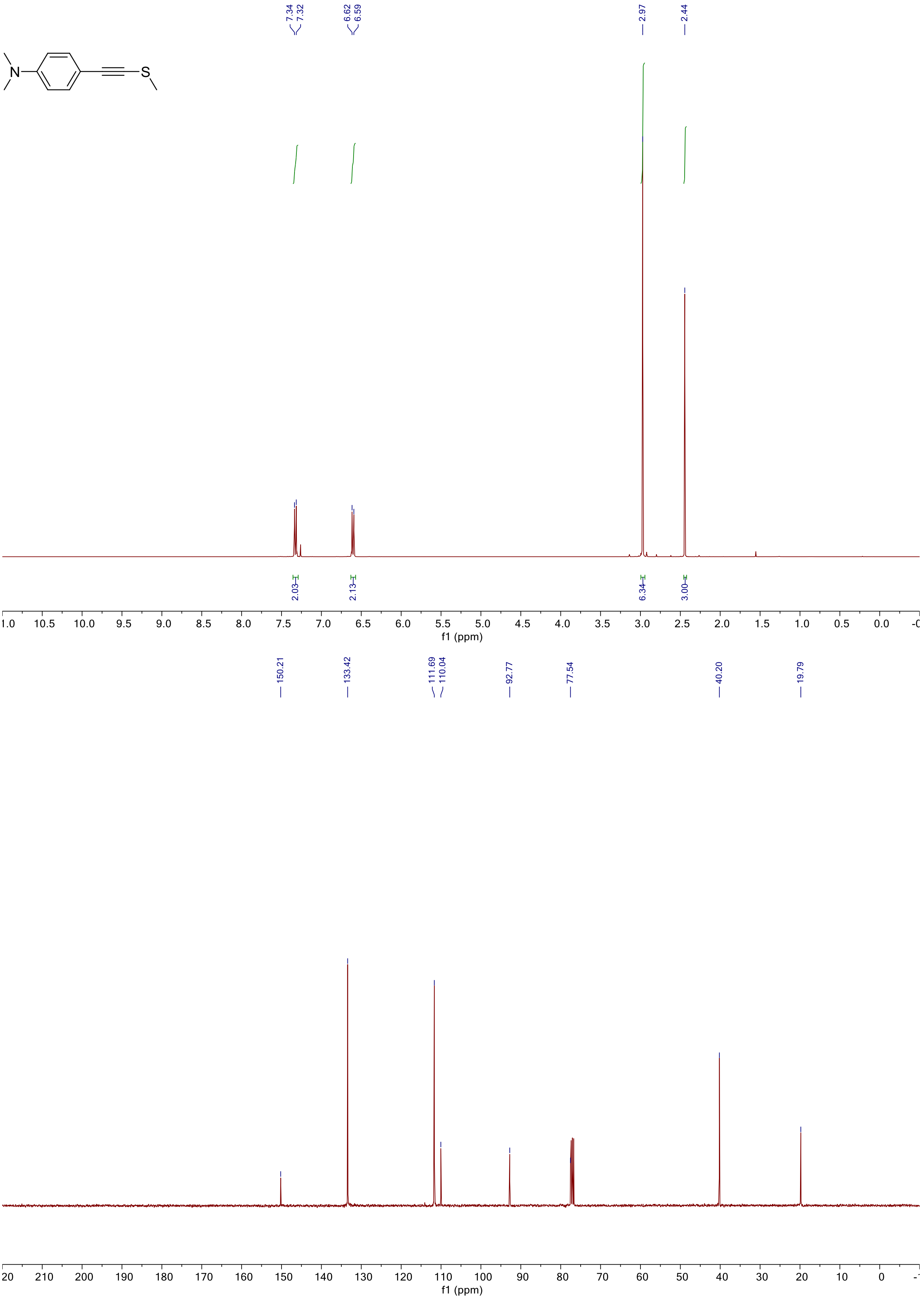

2-((Methylthio)ethynyl)thiophene (1p) in $CDCl_3$ ^1H-NMR and ^{13}C-NMR

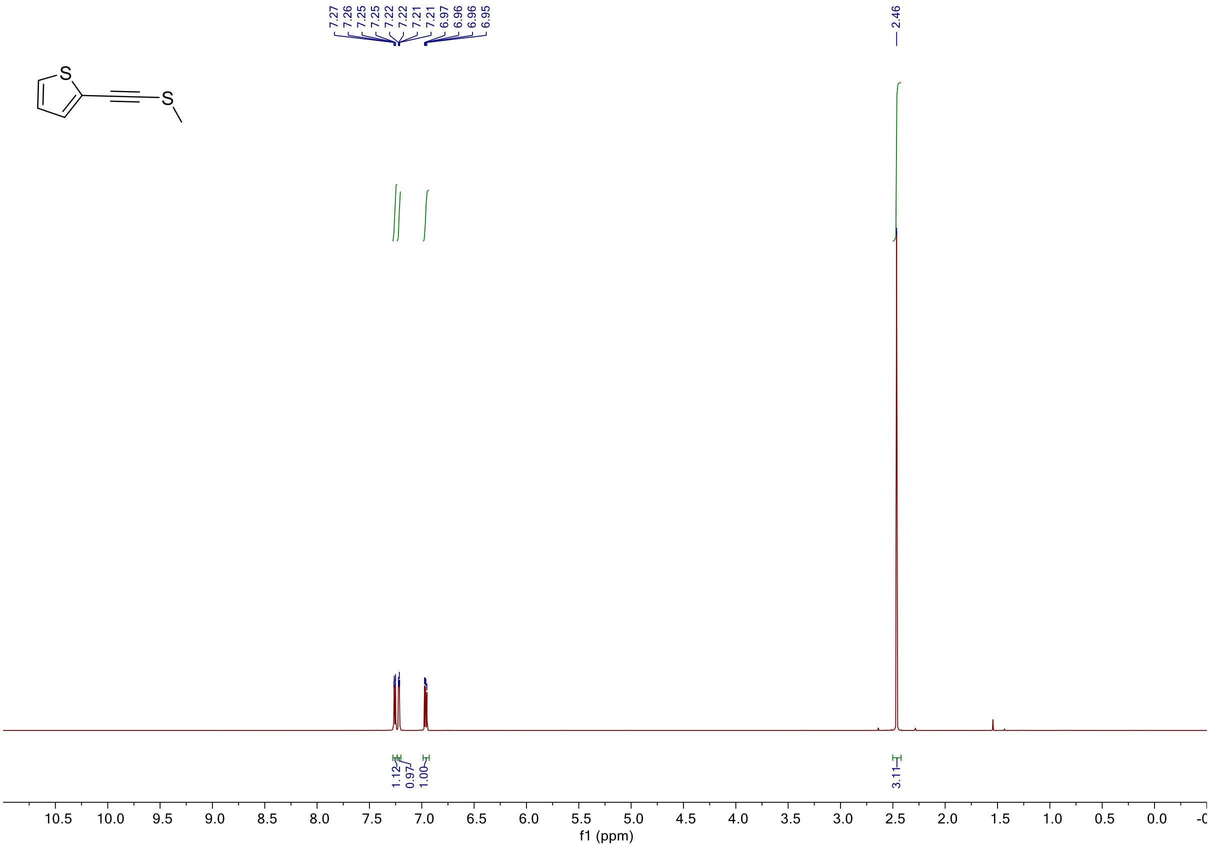

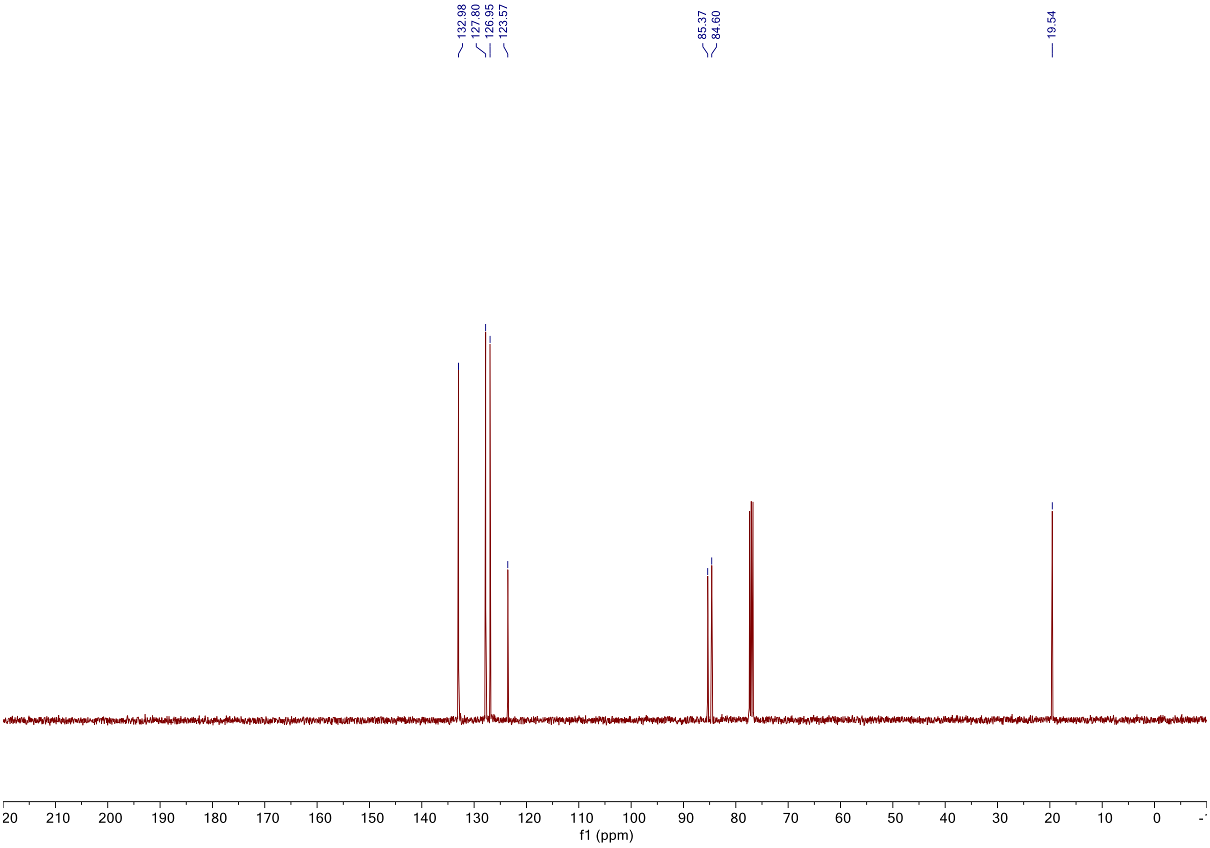

((3,4-Dimethoxyphenyl)ethynyl)(4-methoxybenzyl)sulfane (1q) in CDCl$_3$ ^{1}H-NMR and ^{13}C-NMR

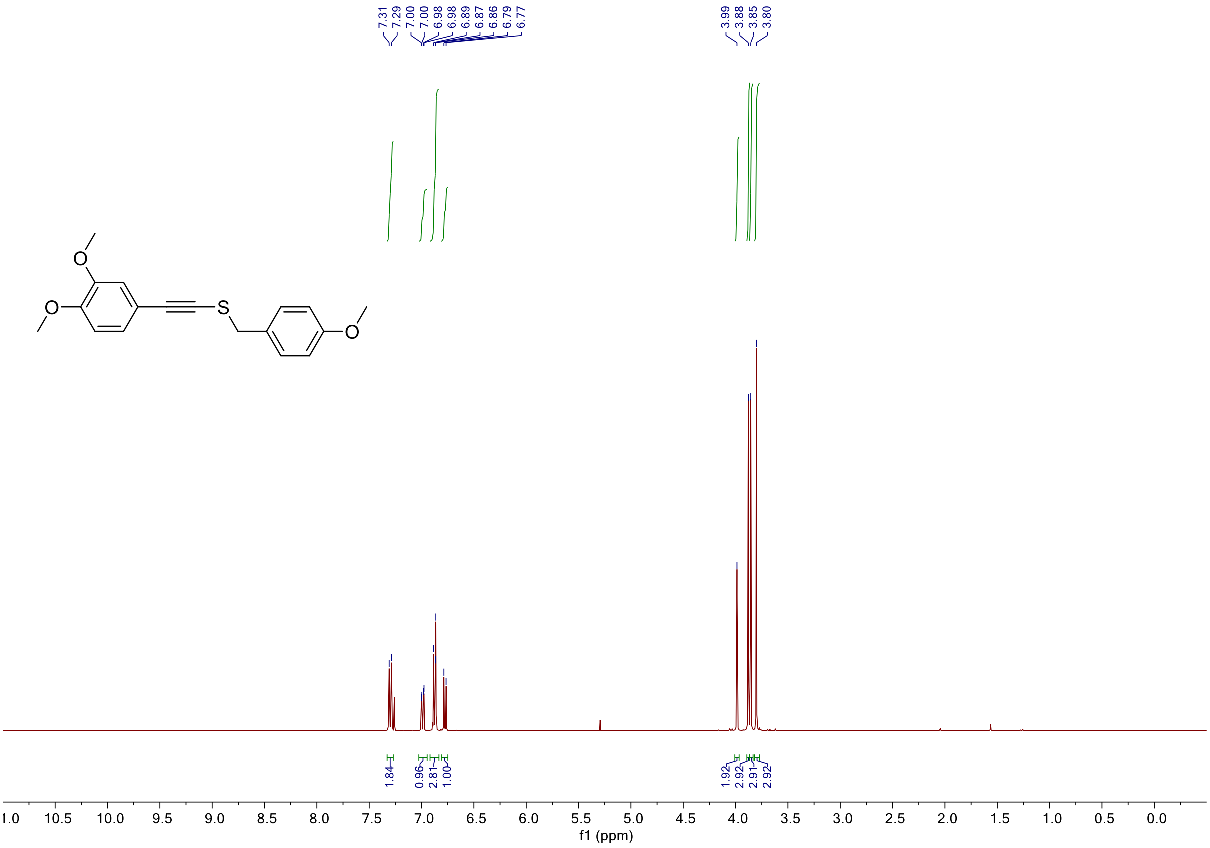

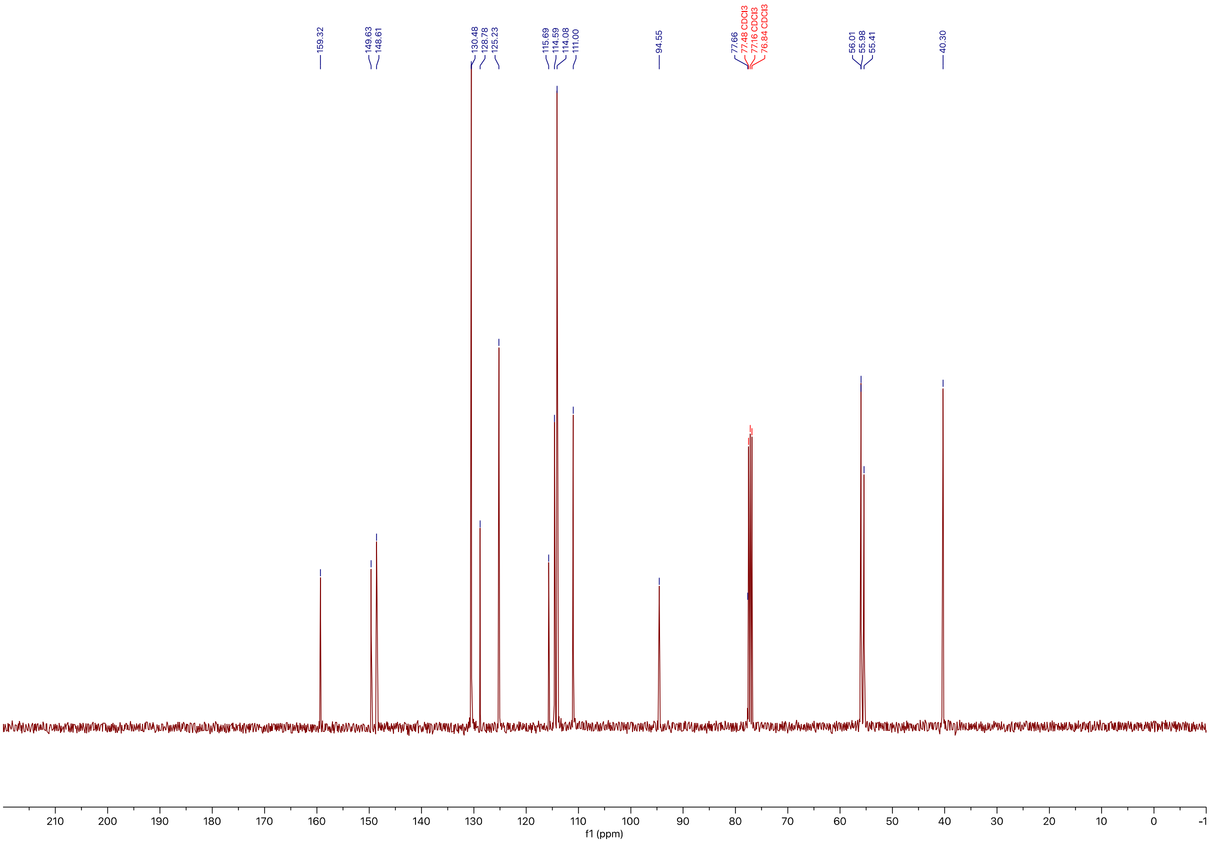

(Cyclopropylethynyl)(methyl)sulfane (1s) in $CDCl_3$ ^{1}H-NMR and ^{13}C-NMR

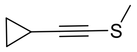

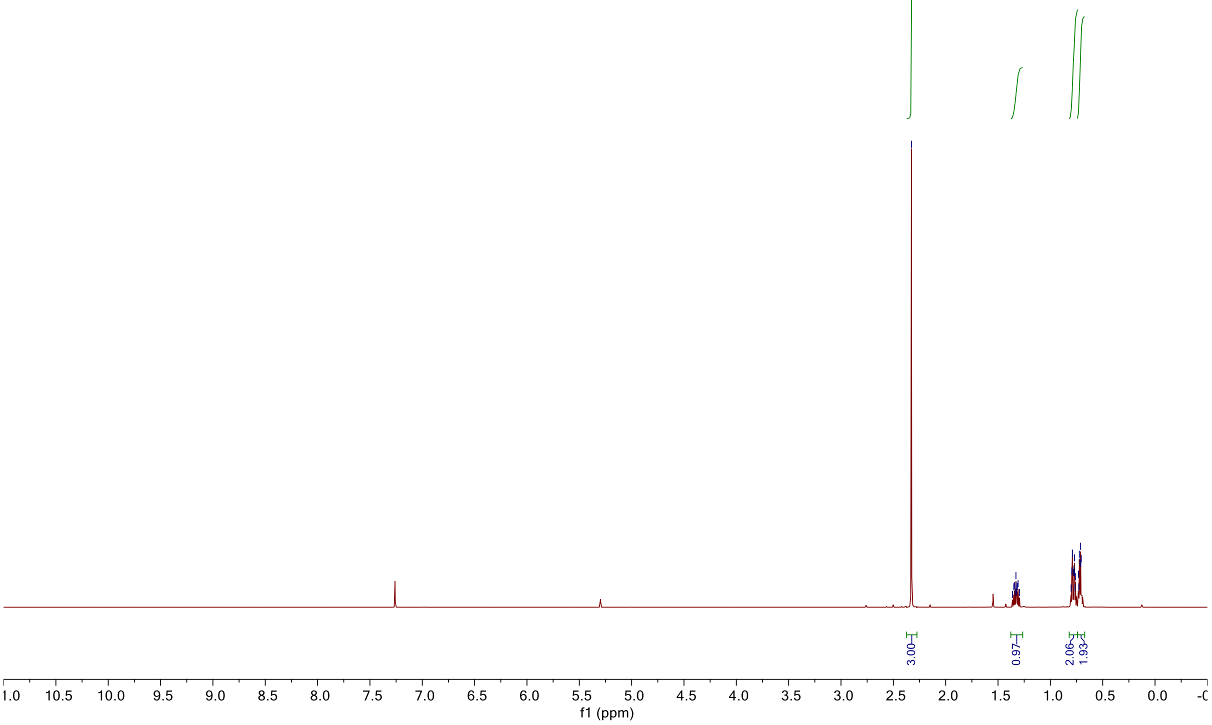

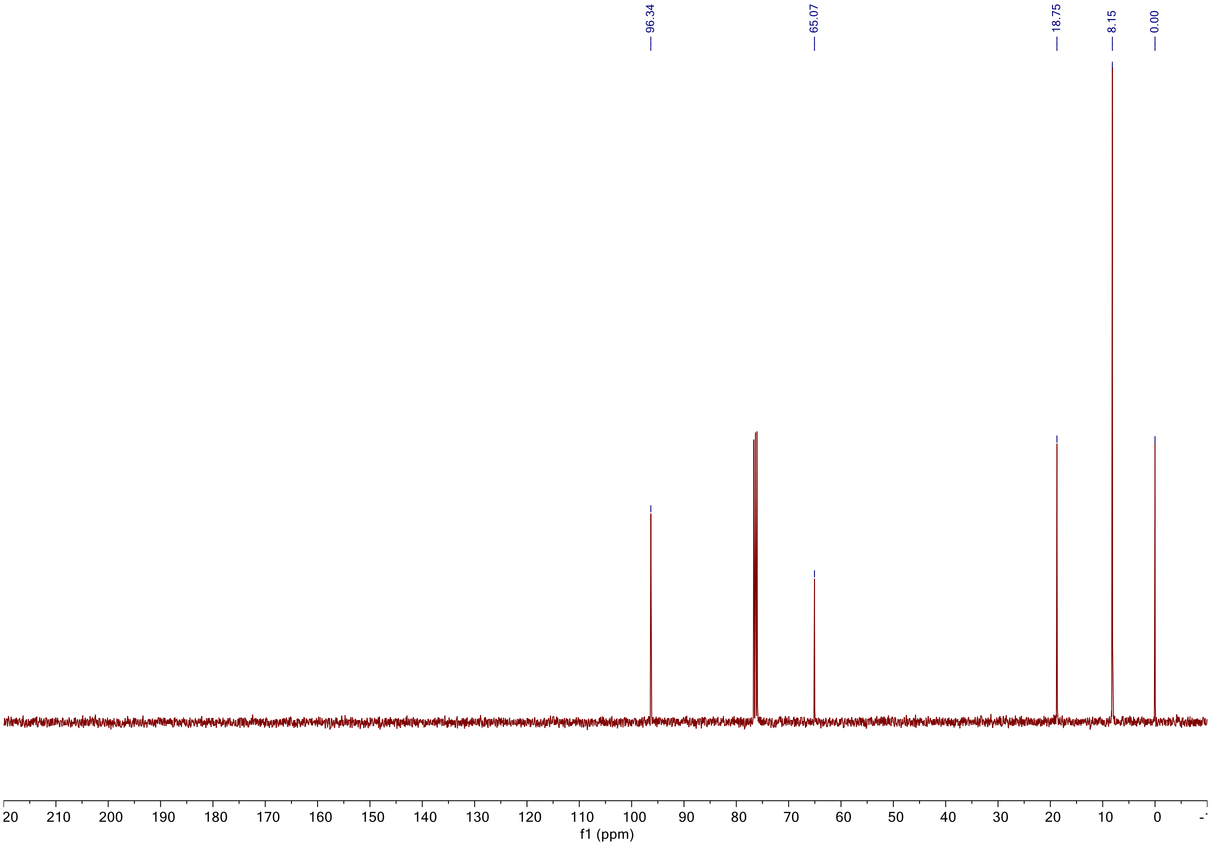

Methyl((phenylthio)ethynyl)sulfane (1t) in $CDCl_3$ ^1H-NMR and ^{13}C-NMR

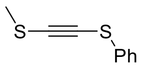

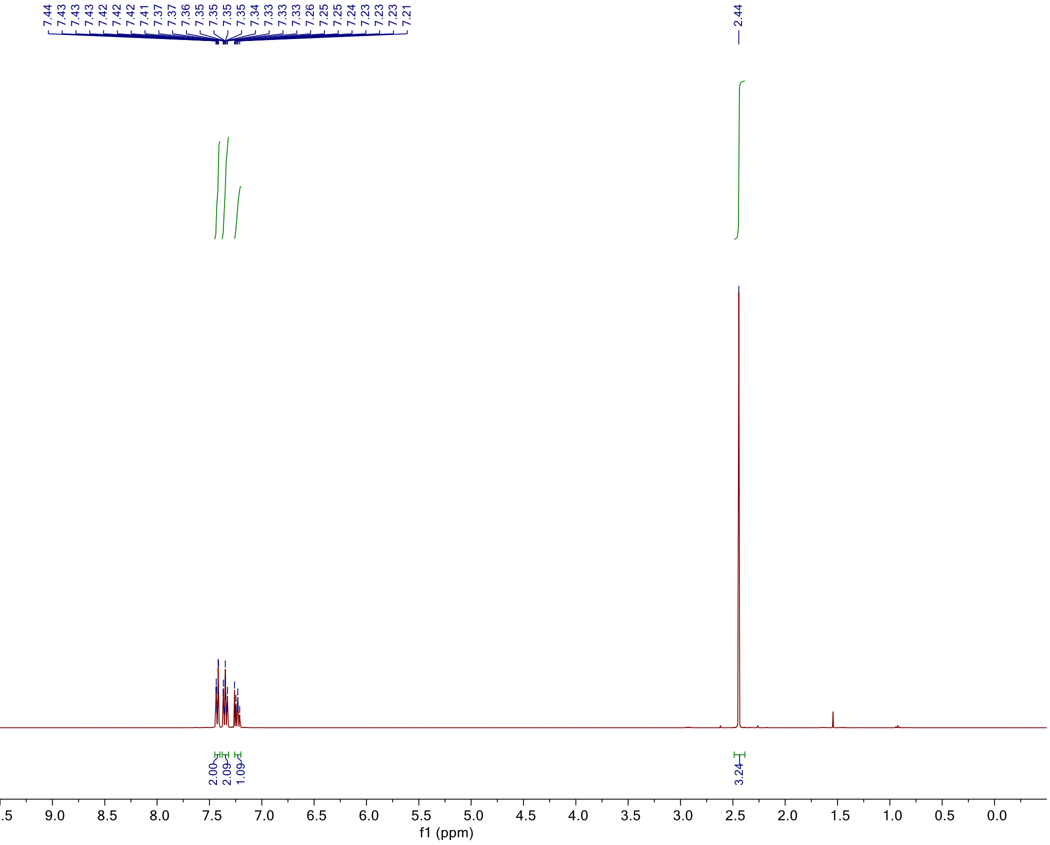

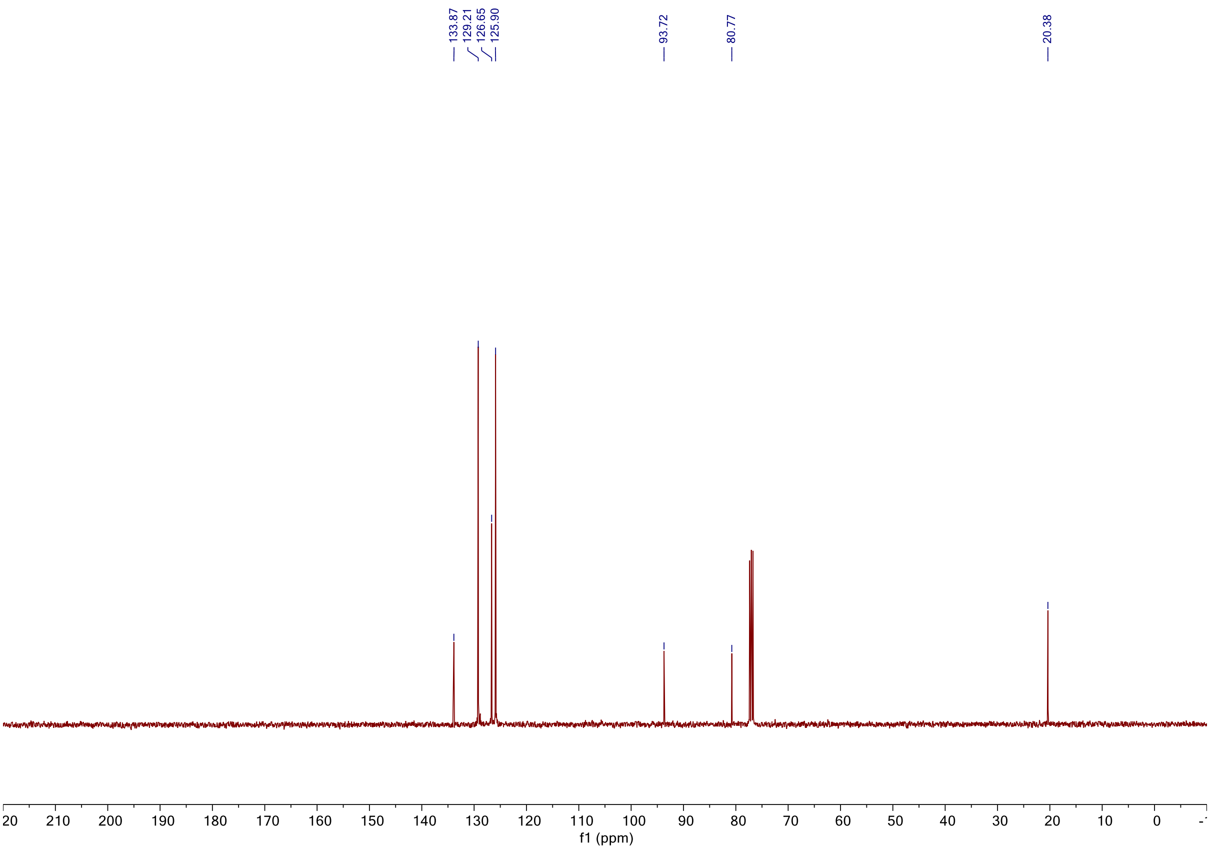

Triisopropyl((methylthio)ethynyl)silane (1u) in $CDCl_3$ ^1H NMR and ^{13}C NMR

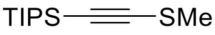

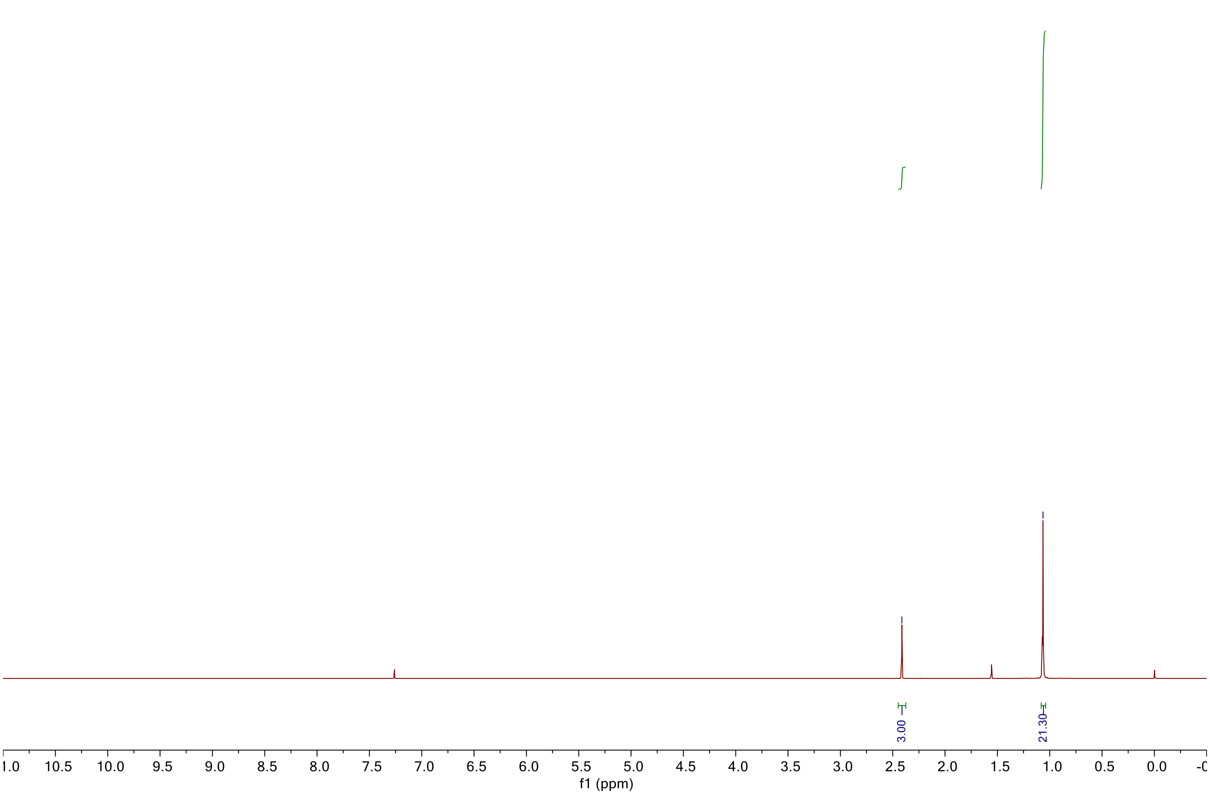

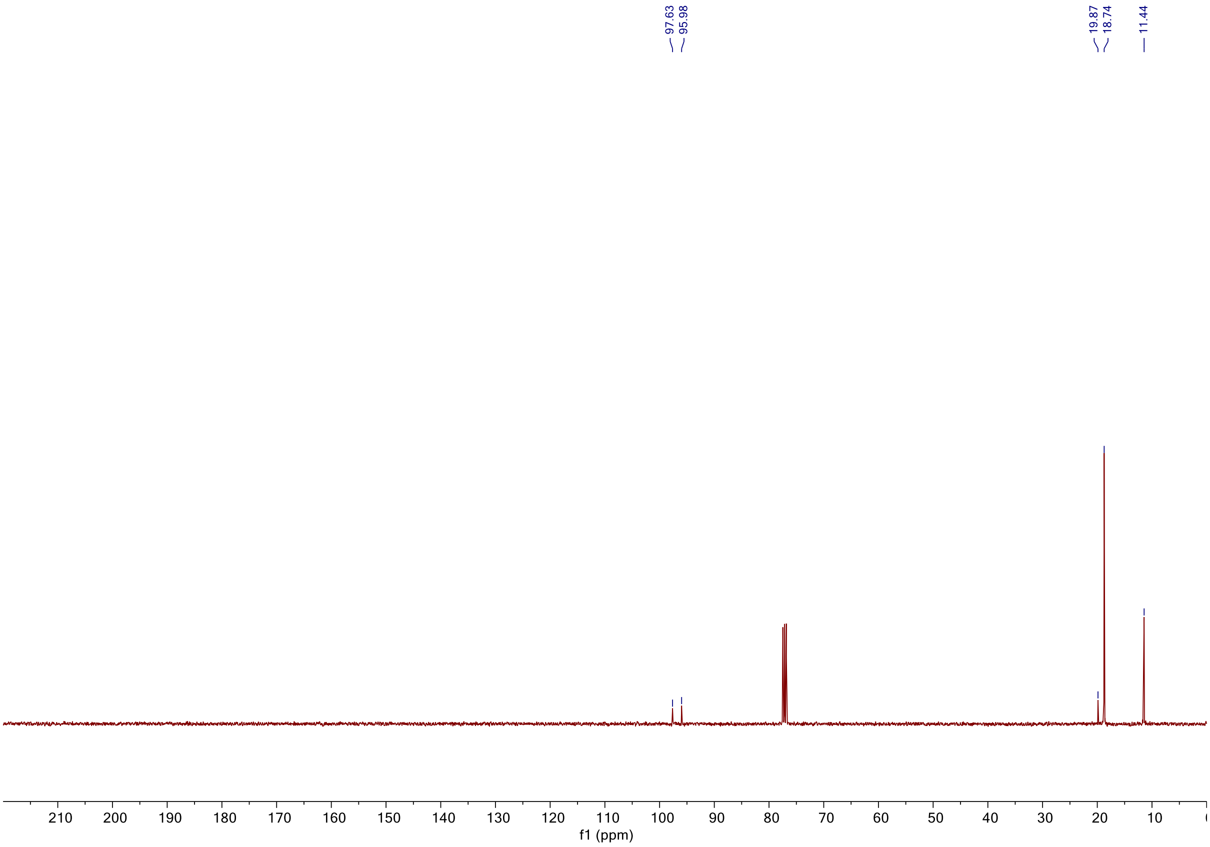

But-3-en-1-yl(ethynyl)sulfane (1v) in $CDCl_3$ ^1H NMR and ^{13}C NMR

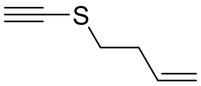

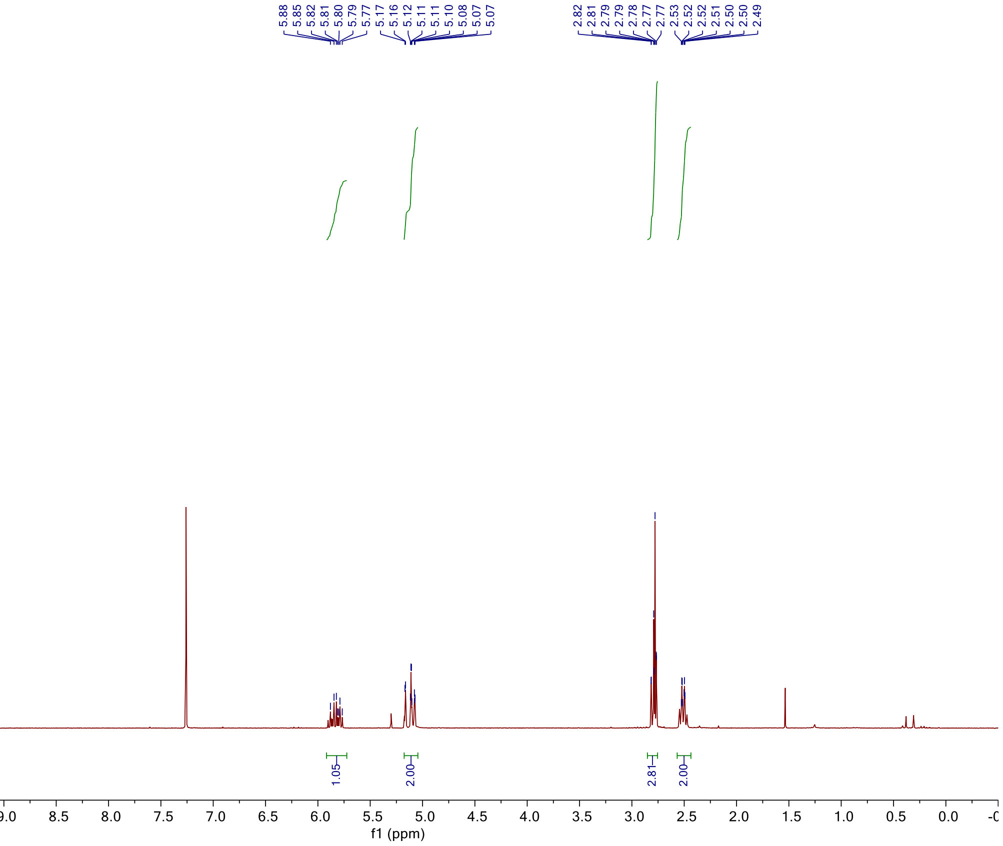

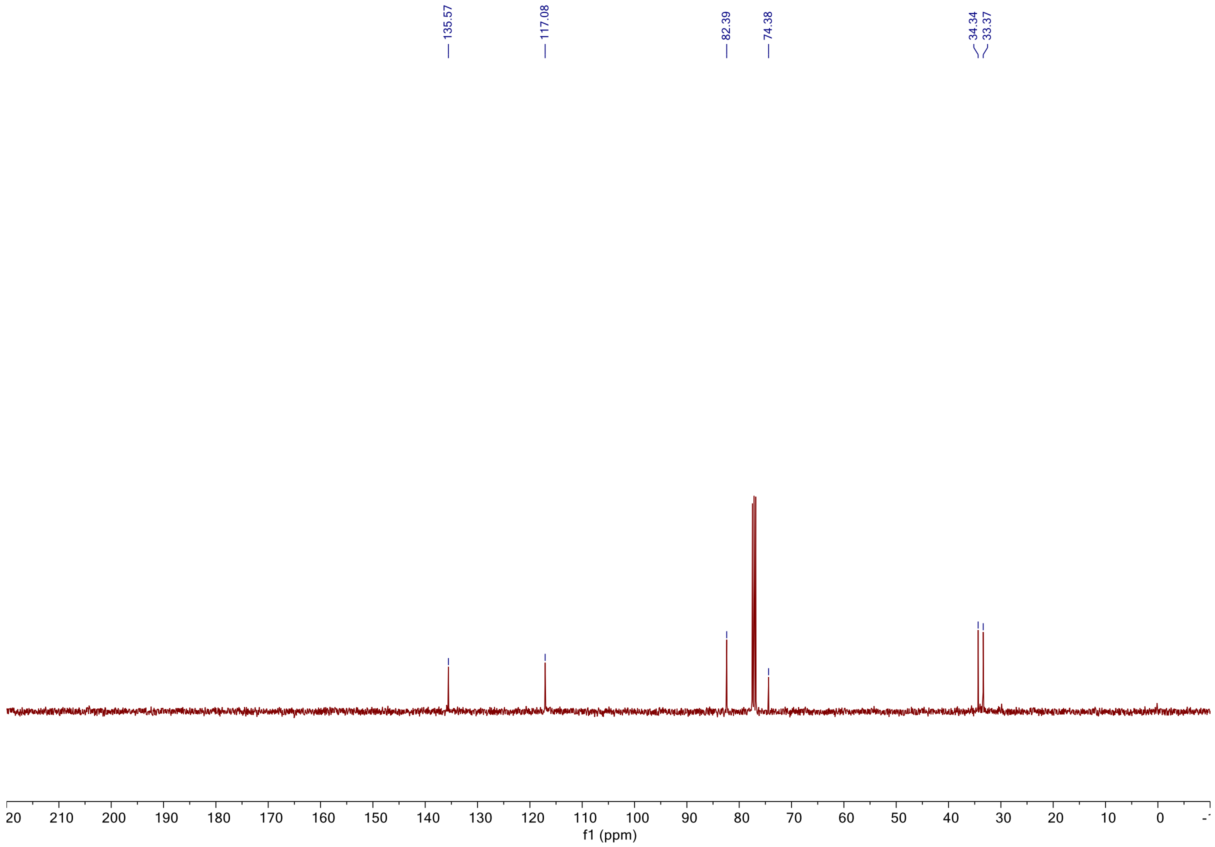

^{13}C Labelled methyl(phenylethynyl)sulfane (^{13}C-1l) in $CDCl_3$ ^{1}H-NMR and ^{13}C-NMR

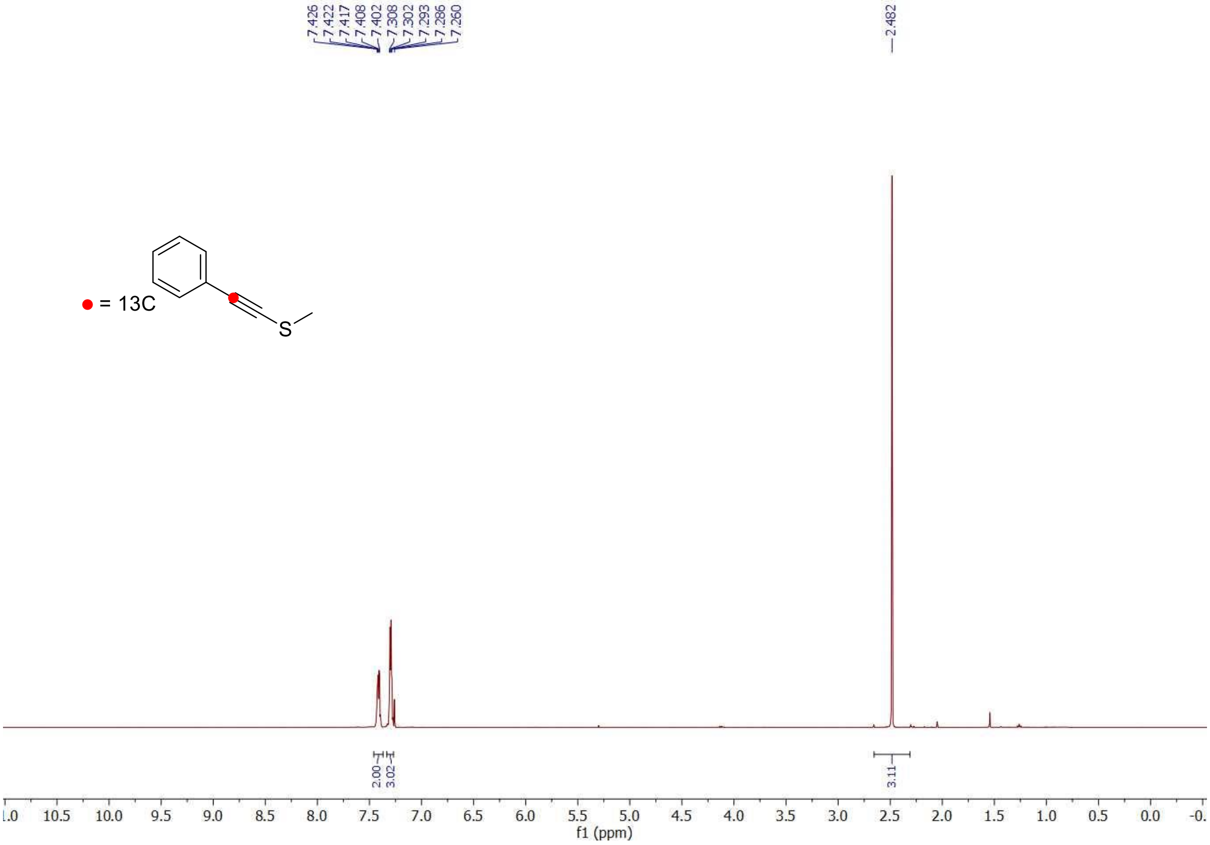

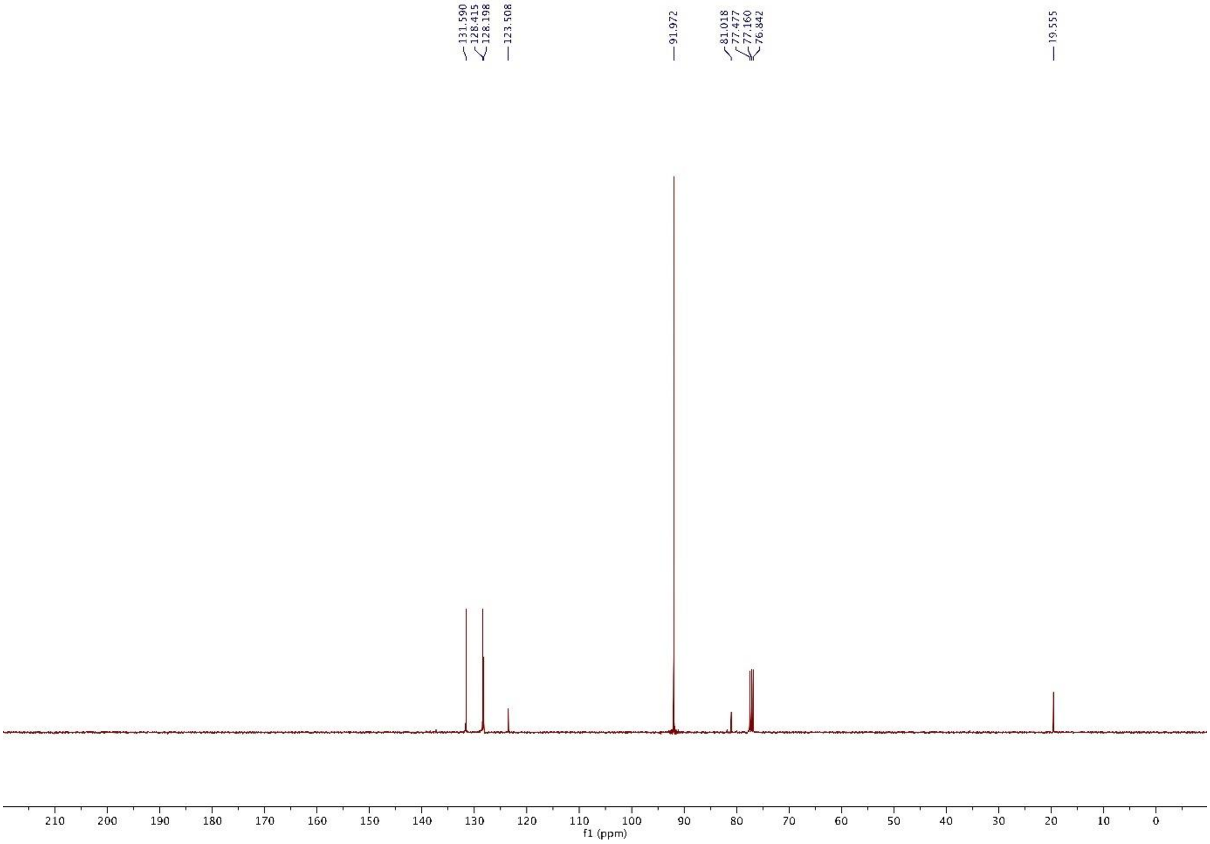

3-(4-Methoxyphenyl)-5-phenylisoxazole (2c) in $CDCl_3$ ^1H-NMR and ^{13}C-NMR

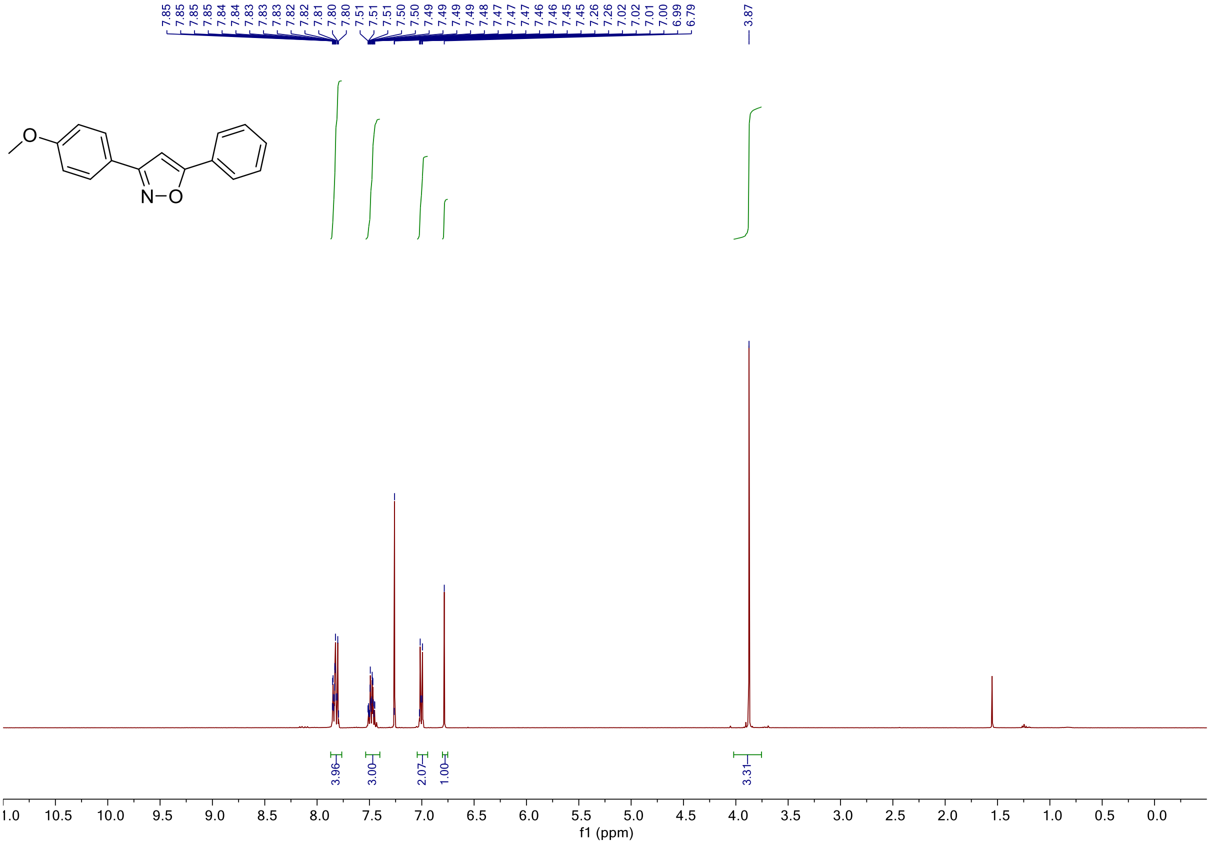

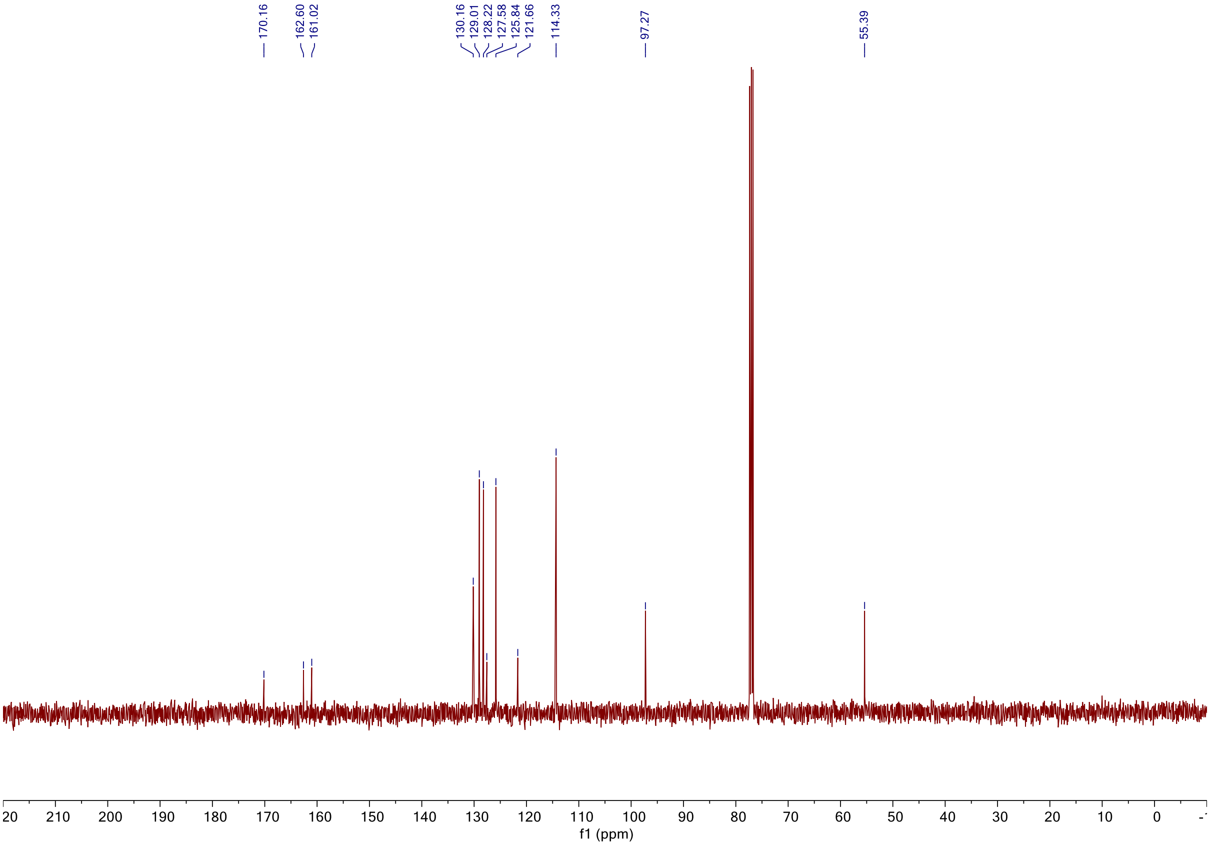

5-Butyl-3-(4-methoxyphenyl)isoxazole (2d) in $CDCl_3$ ^{1}H-NMR and ^{13}C-NMR

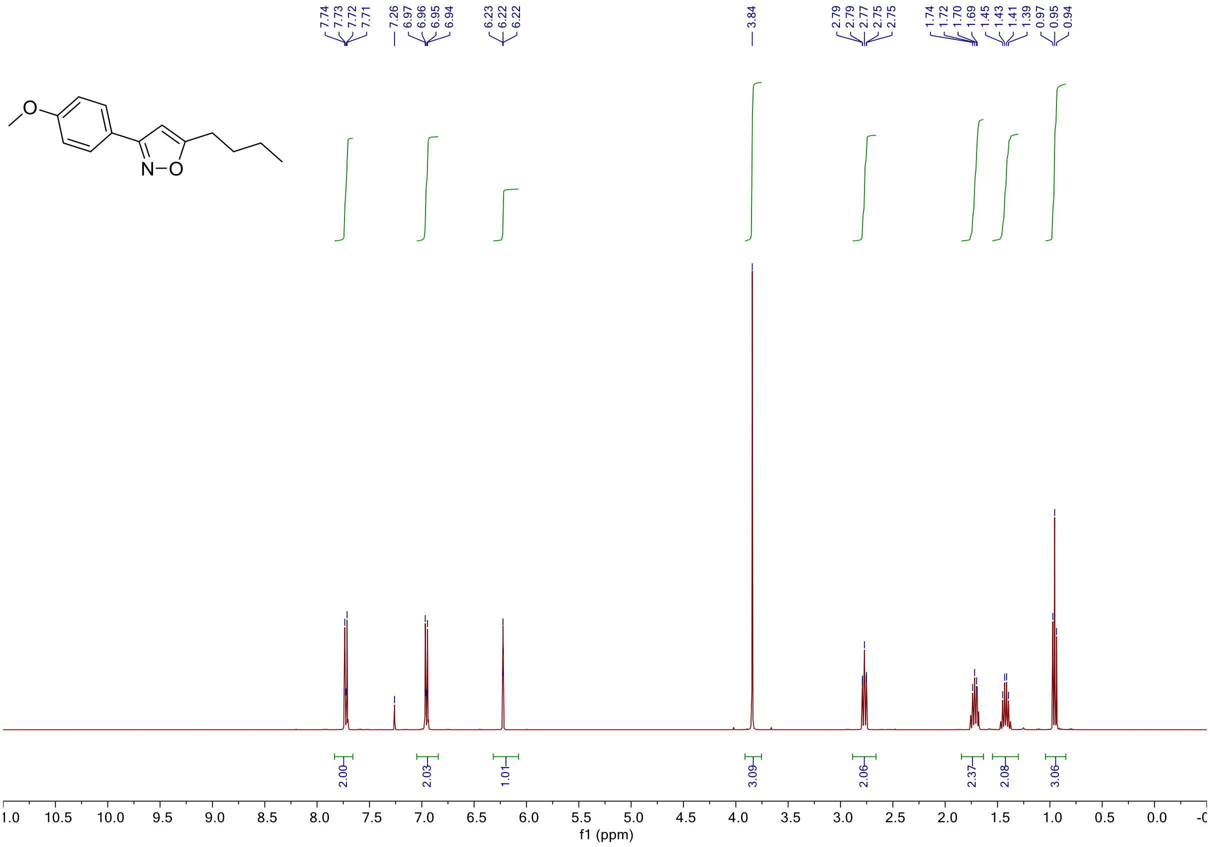

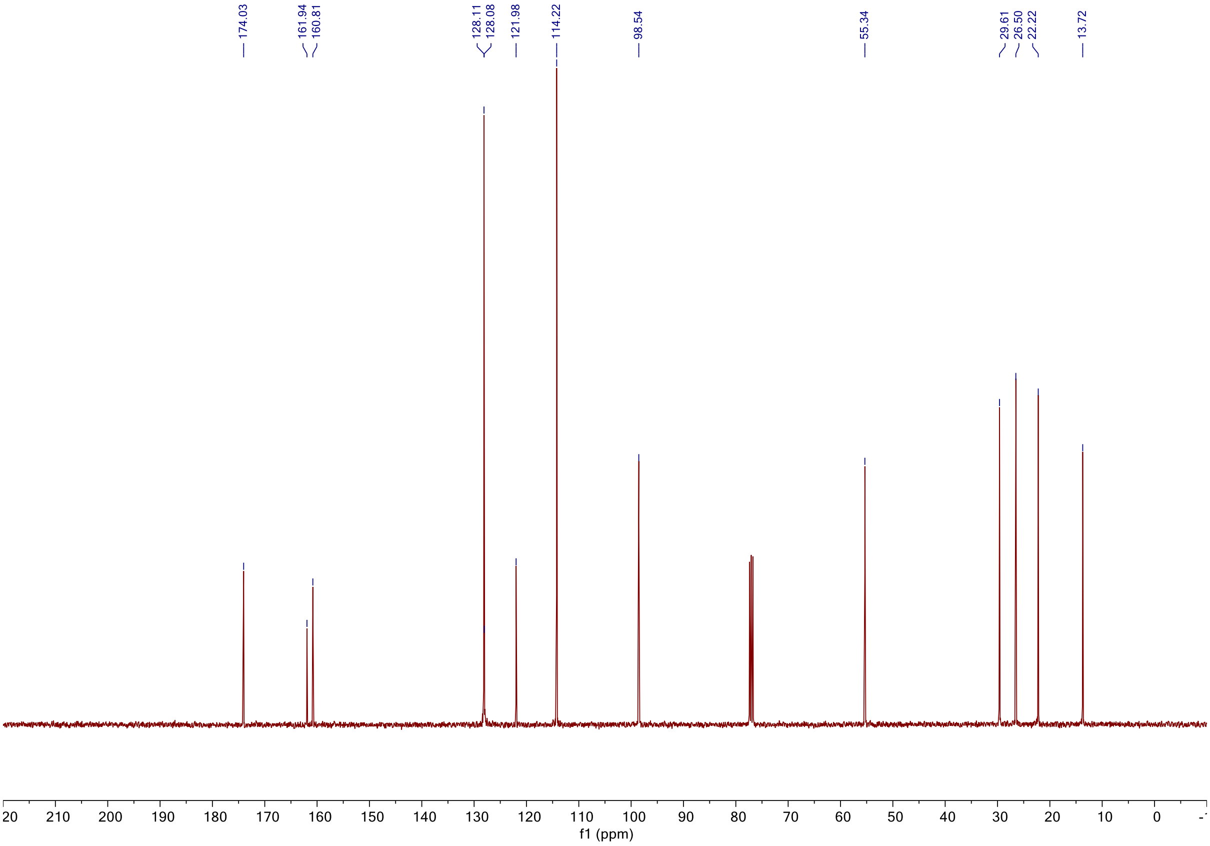

(*E*)-5-Phenyl-3-styrylisoxazole (2h) in $CDCl_3$ ^{1}H-NMR and ^{13}C-NMR

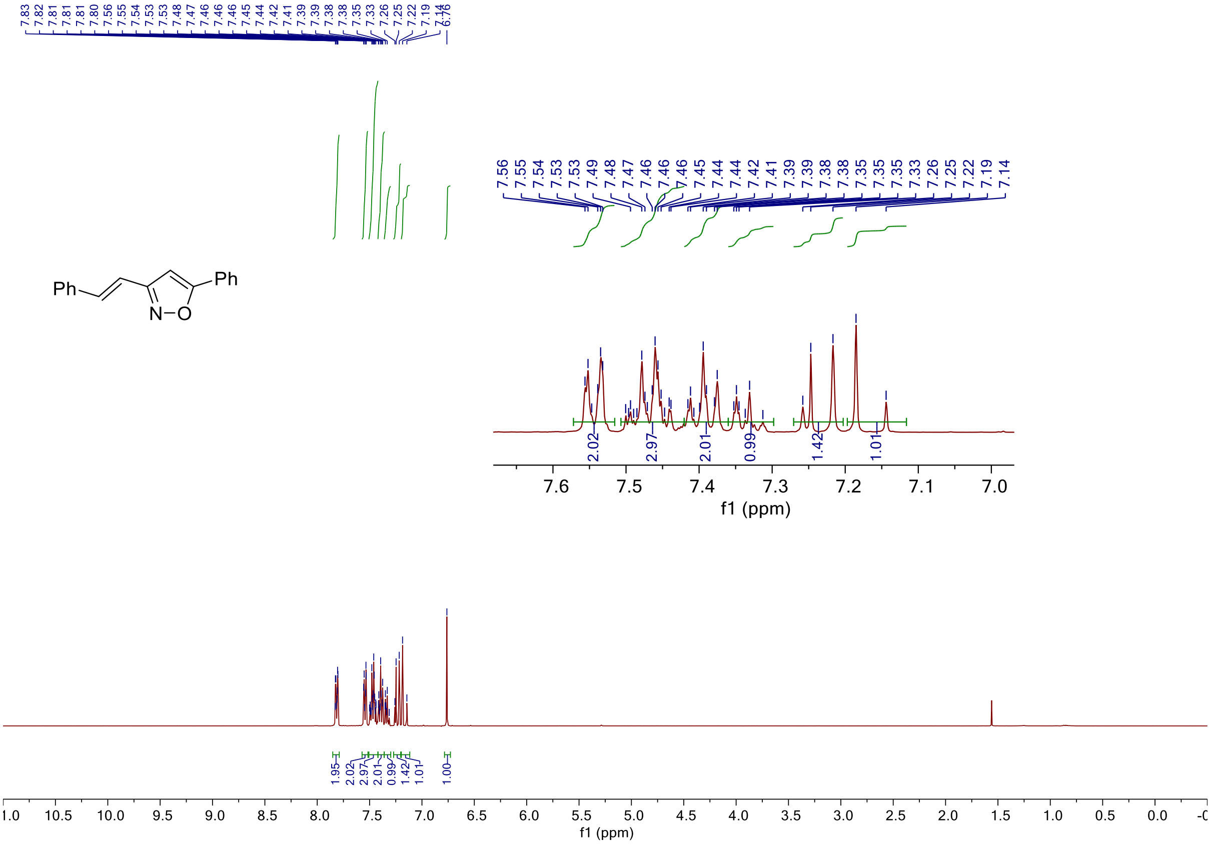

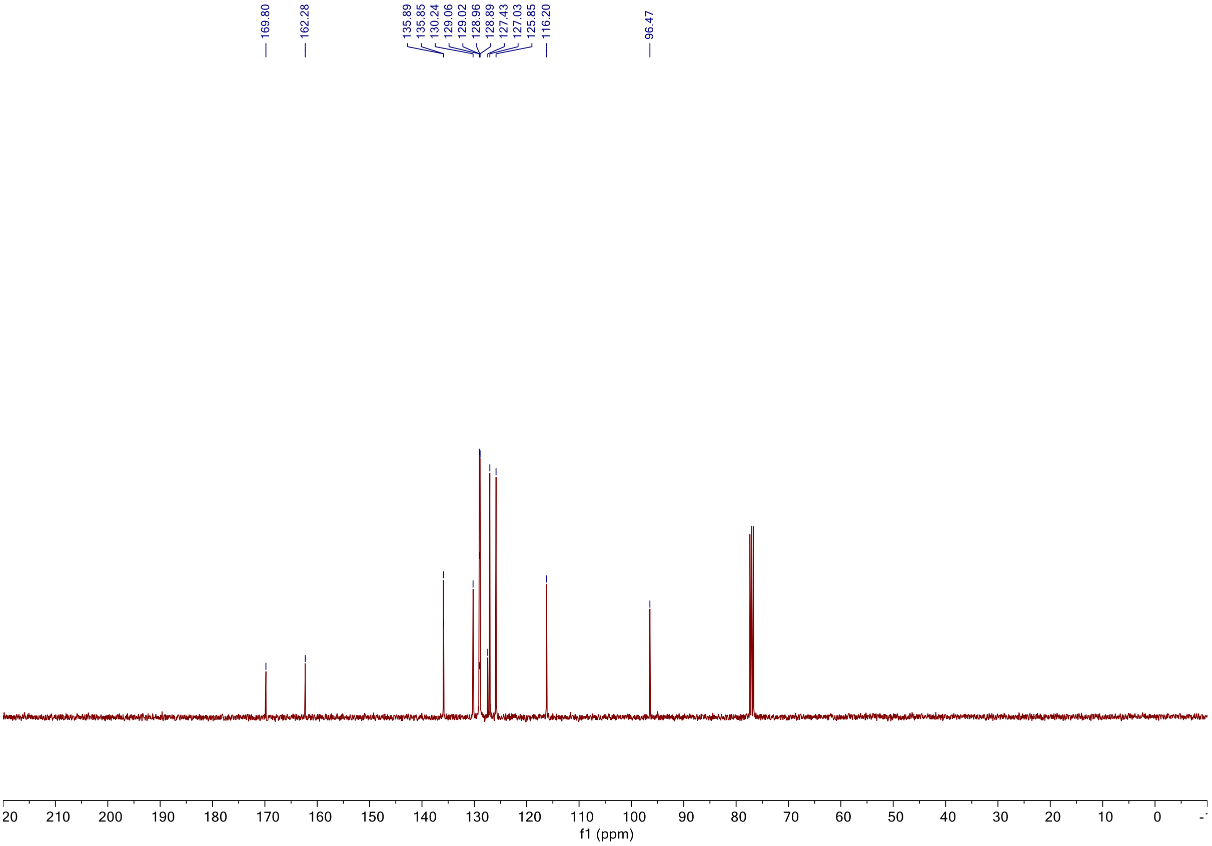

3-cyclohexyl-5-phenylisoxazole (2k) in $CDCl_3$ ^1H-NMR and ^{13}C-NMR

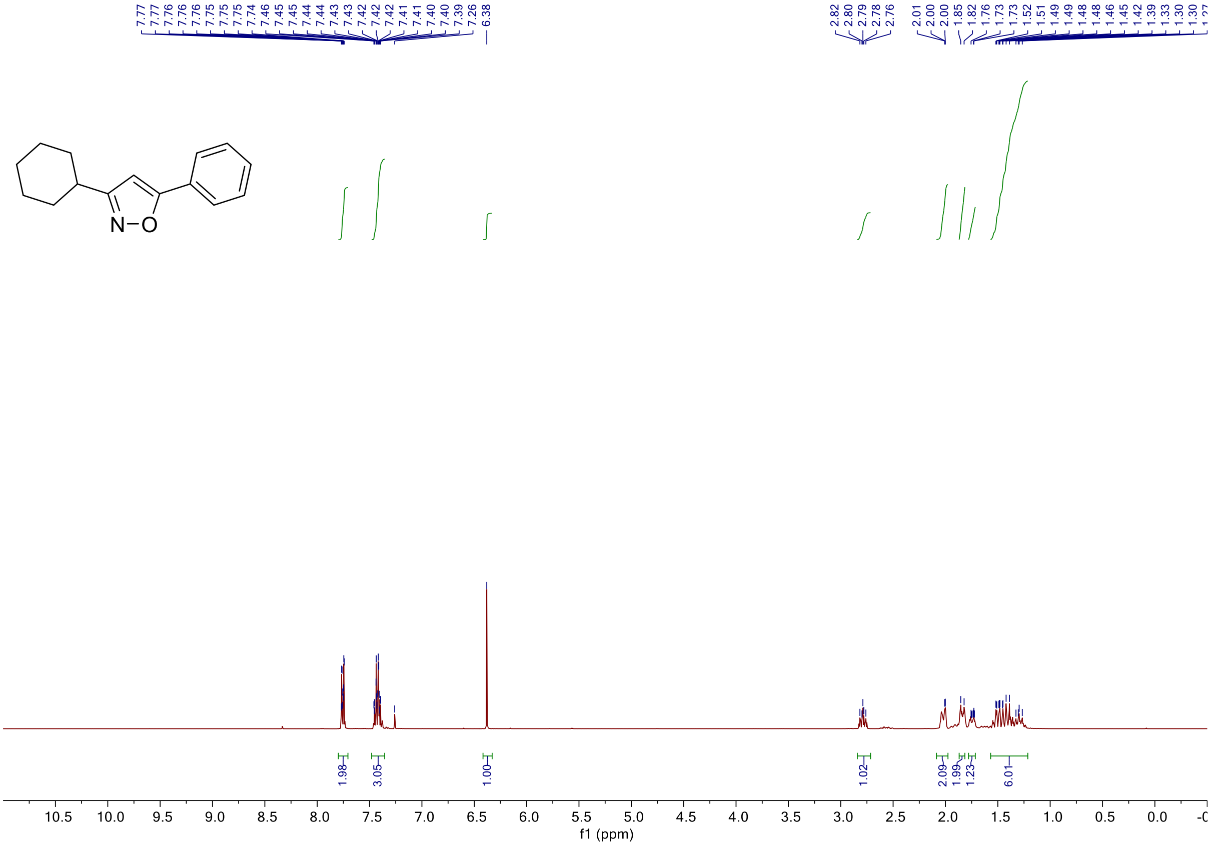

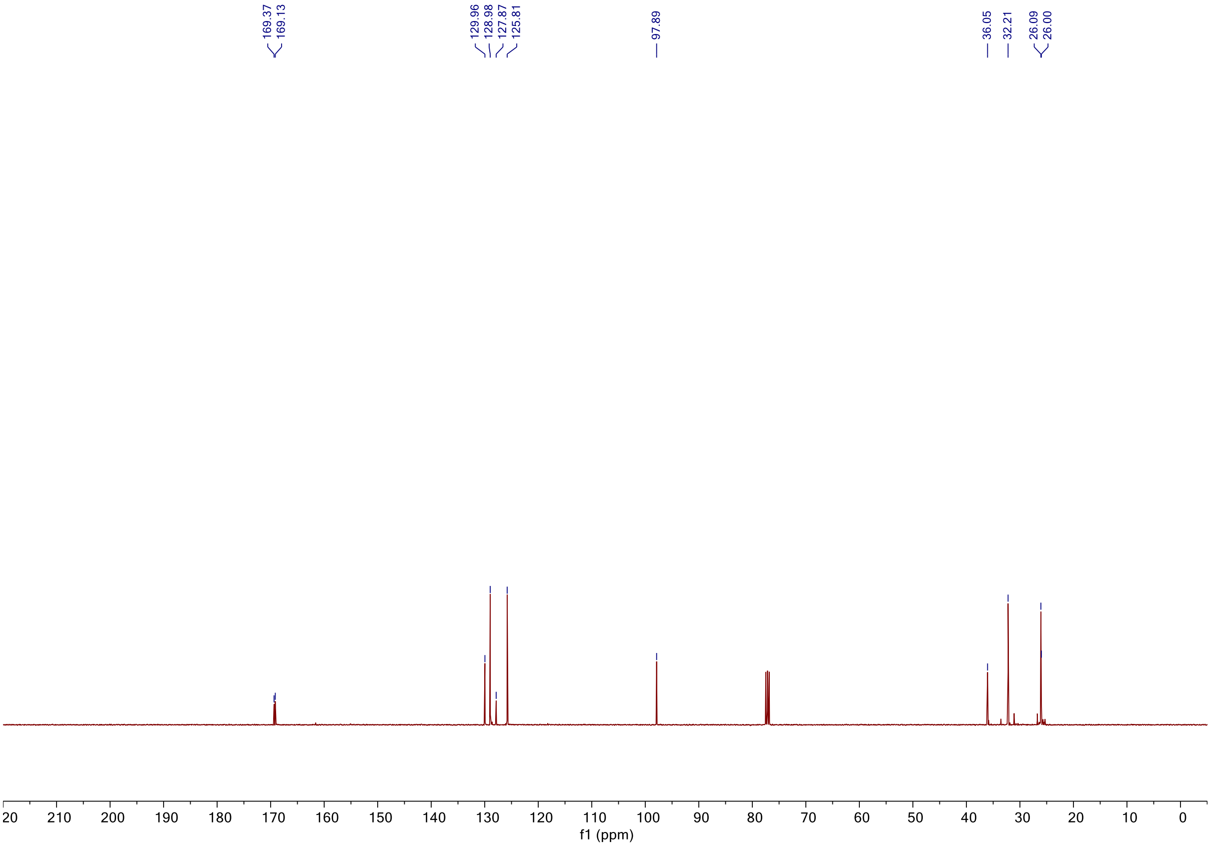

3-(2-Bromophenyl)-5-phenylisoxazole (2l) in $CDCl_3$ ^1H-NMR and ^{13}C-NMR

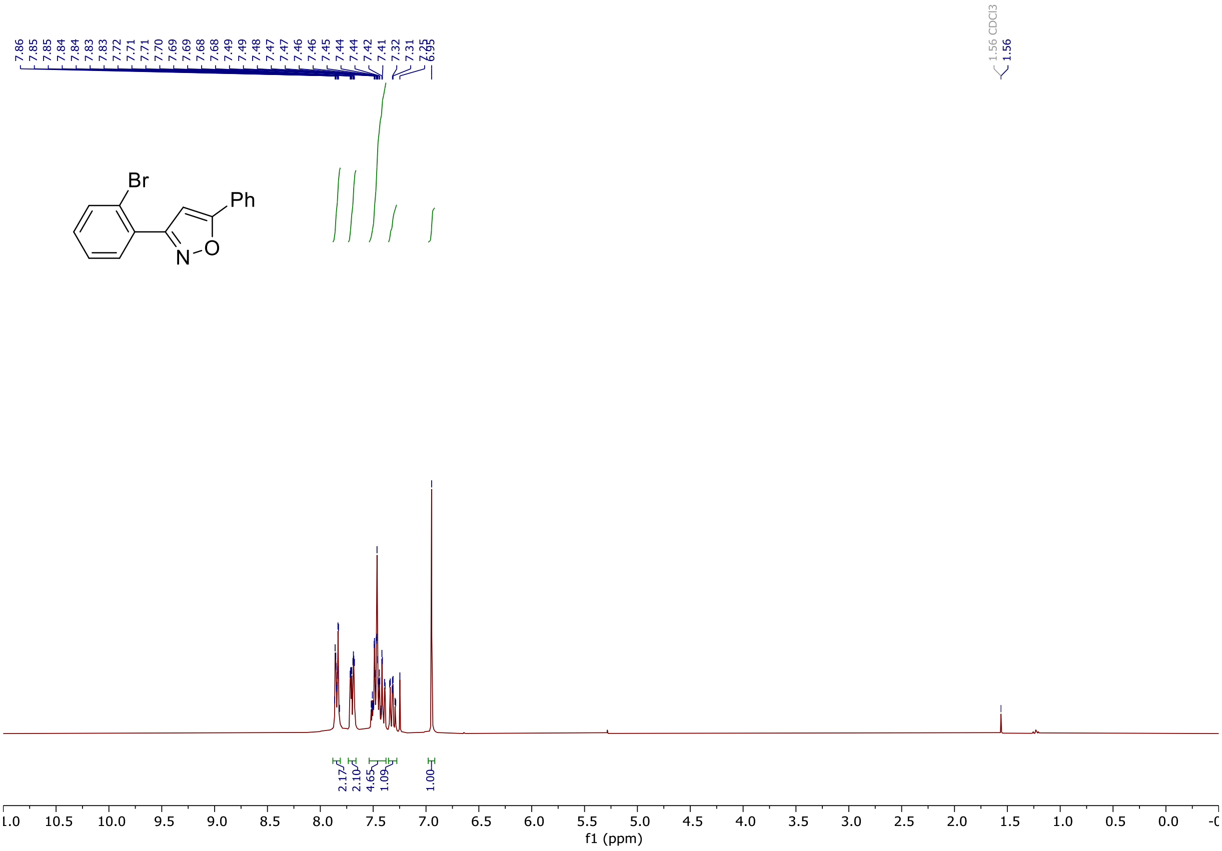

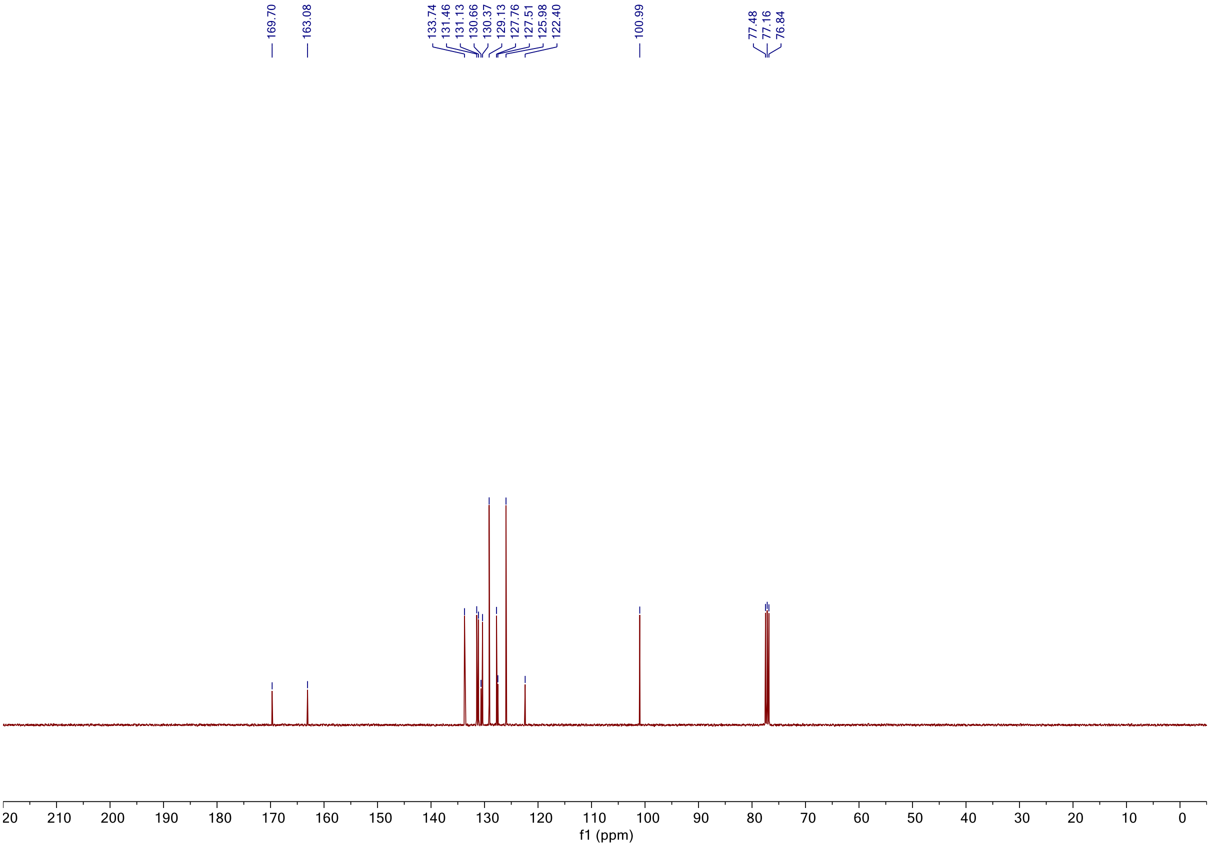

1-(5-(4-Methoxyphenyl)-2-methyl-4-(methylthio)-1*H*-pyrrol-3-yl)ethan-1-one (3a) in $CDCl_3$ ^{1}H-NMR and ^{13}C-NMR

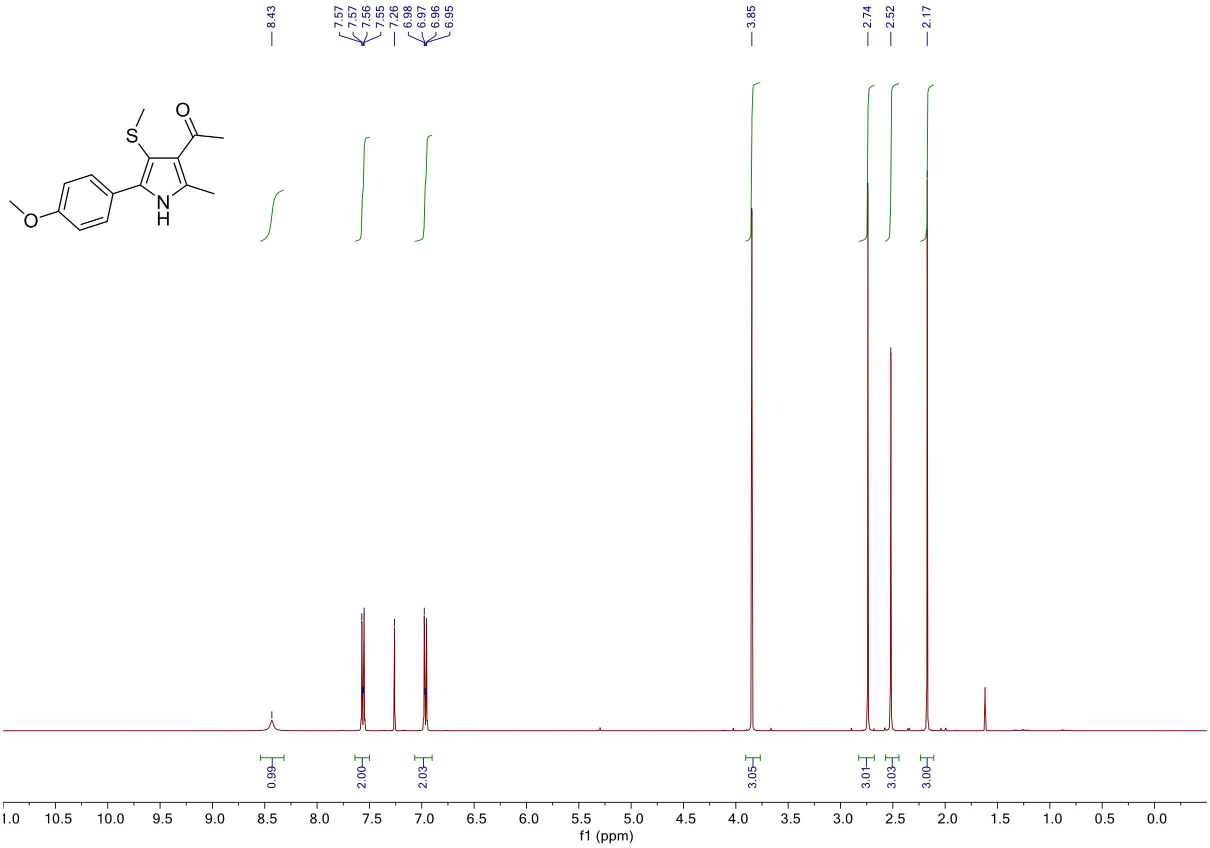

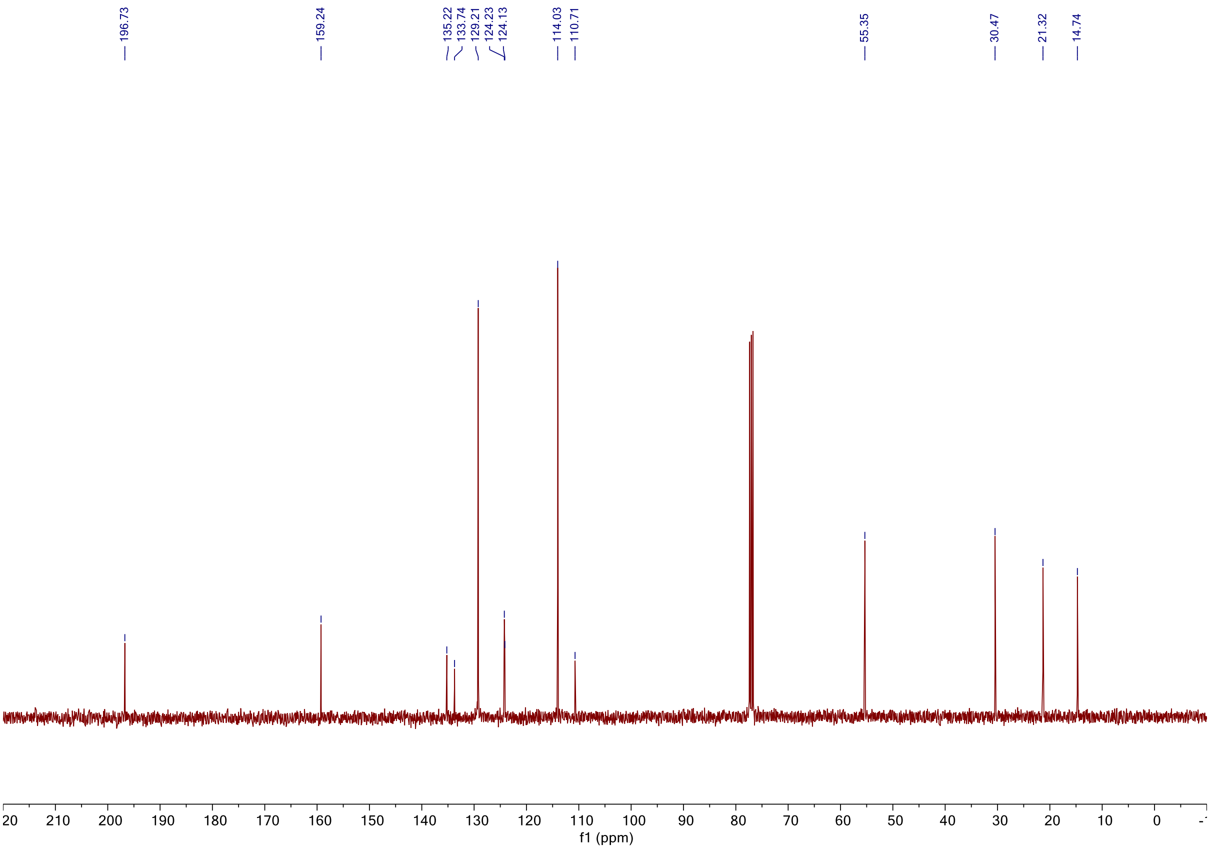

(4-(Benzylthio)-5-(4-methoxyphenyl)-2-methyl-1*H*-pyrrol-3-yl)ethan-1-one (3c) in $CDCl_3$ ^1H-NMR and ^{13}C-NMR

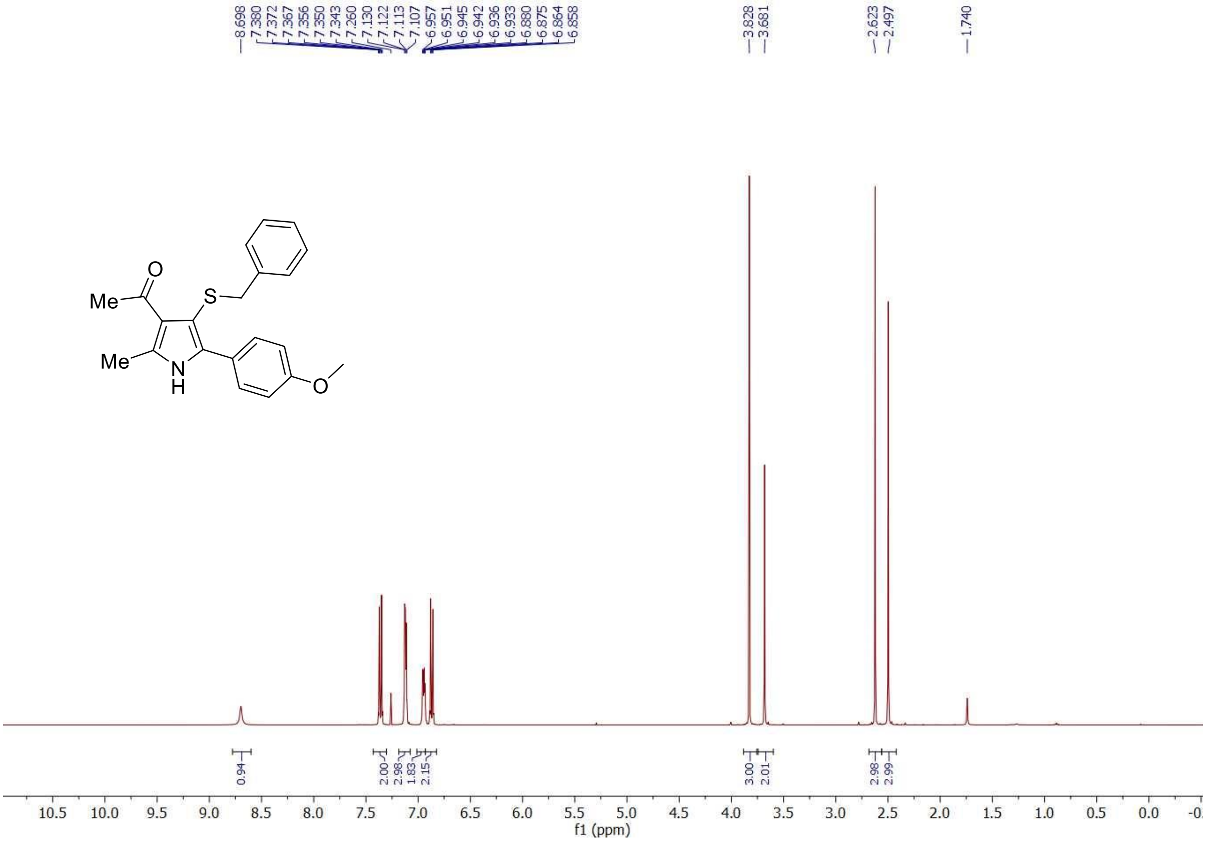

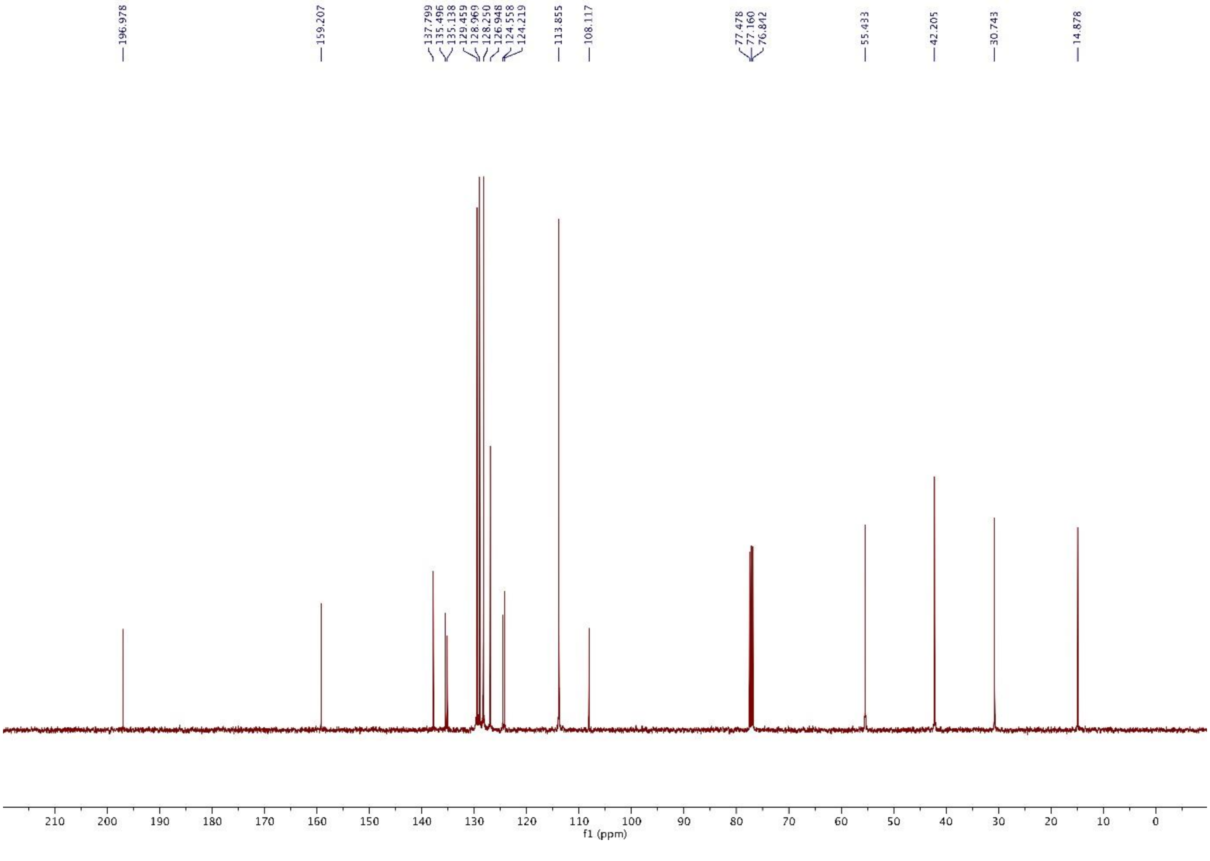

1-(4-((4-Methoxybenzyl)thio)-5-(4-methoxyphenyl)-2-methyl-1*H*-pyrrol-3-yl)ethan-1-one (3d) in $CDCl_3$ ^{1}H-NMR and ^{13}C-NMR

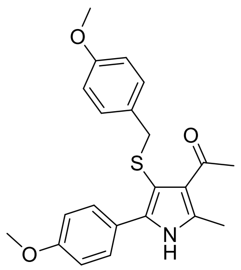

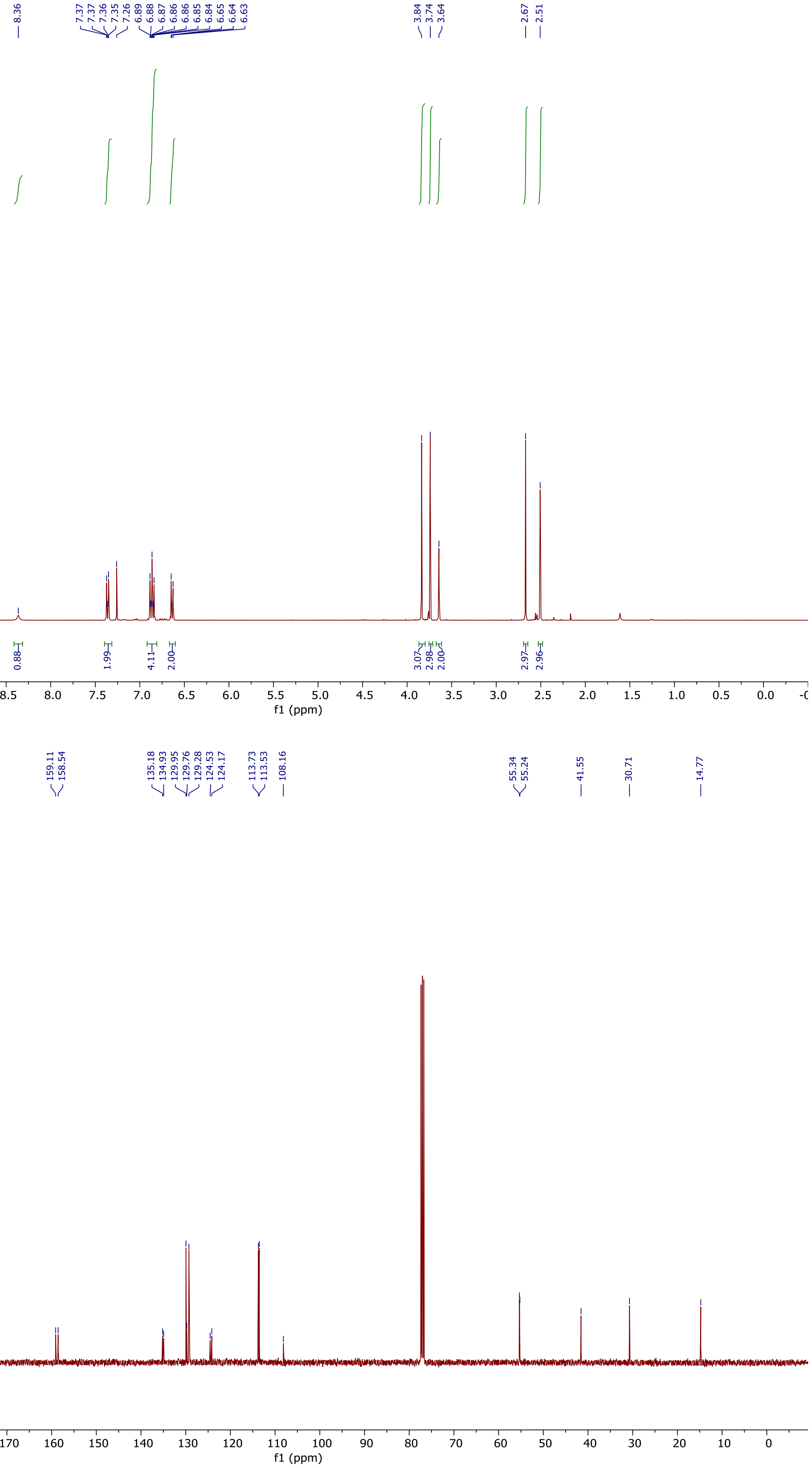

1-(4-(But-3-en-1-ylthio)-5-(4-methoxyphenyl)-2-methyl-1*H*-pyrrol-3-yl)ethan-1-one (3e) in $CDCl_3$ ^{1}H-NMR and ^{13}C-NMR

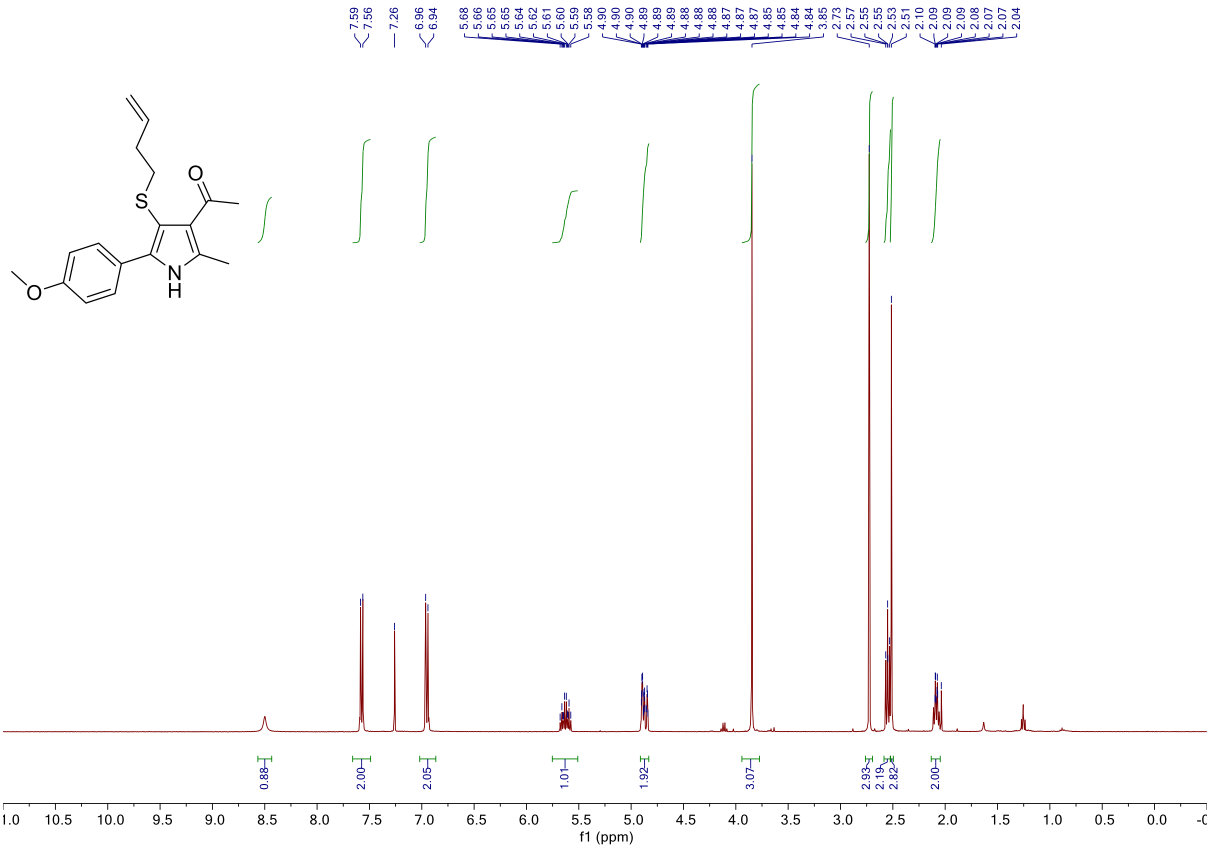

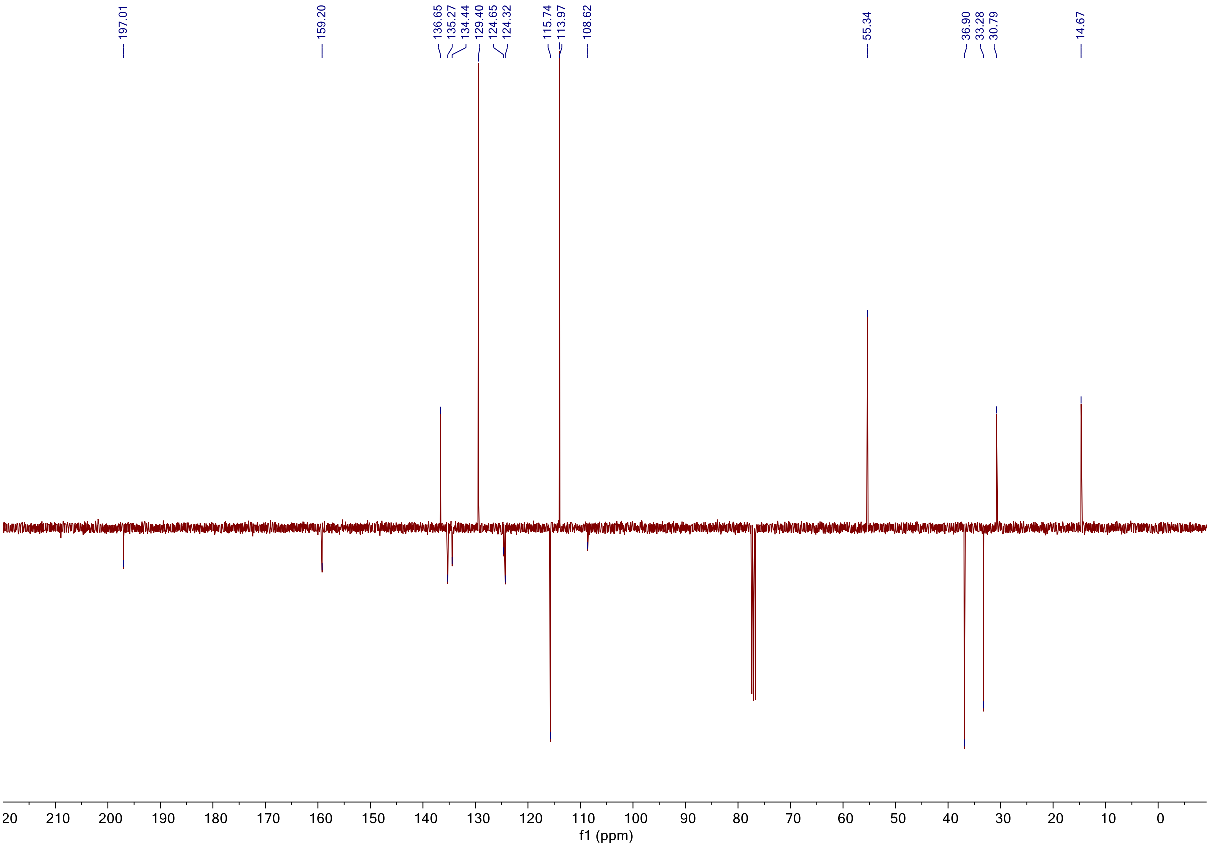

Ethyl 2-((4-acetyl-2-(4-methoxyphenyl)-5-methyl-1*H*-pyrrol-3-yl)thio)acetate (3g) in $CDCl_3$ ^{1}H-NMR and ^{13}C-NMR

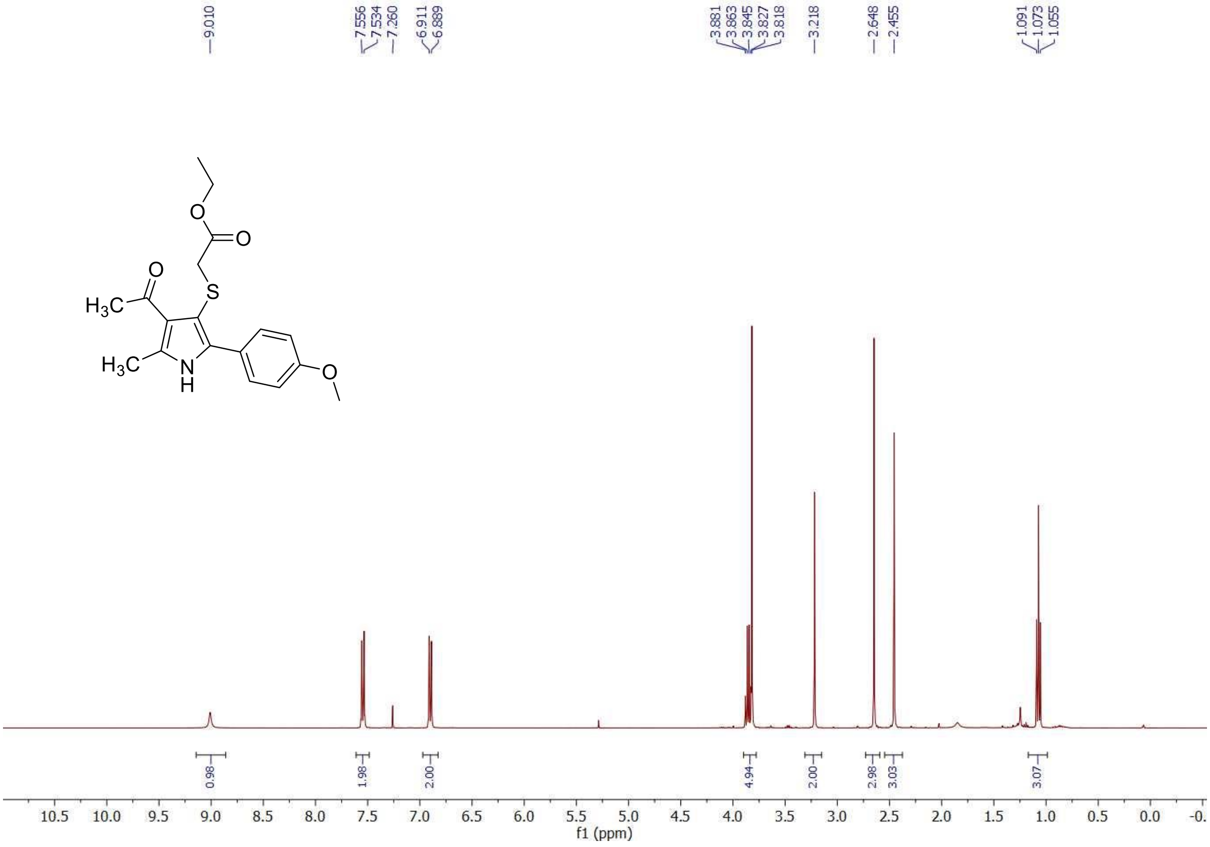

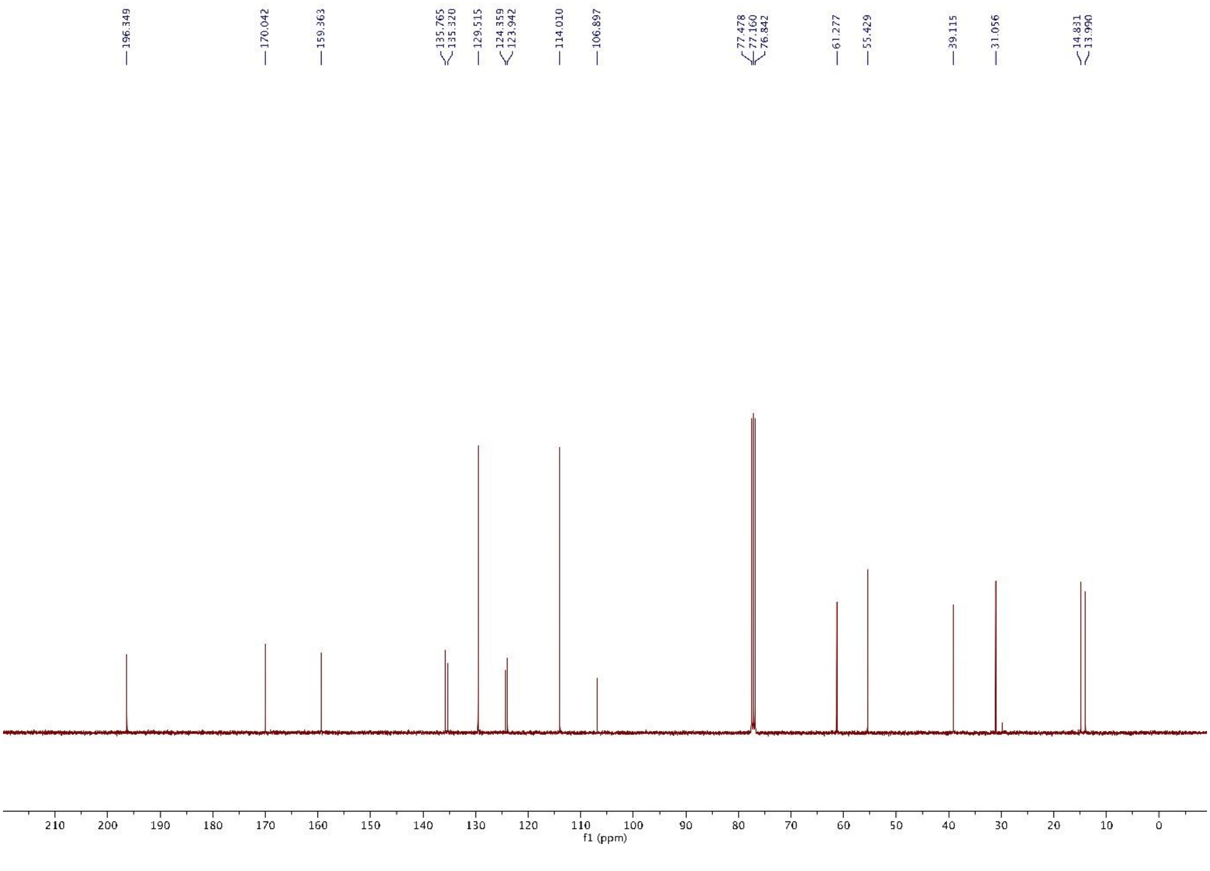

1-(5-(2-Methoxyphenyl)-2-methyl-4-(methylthio)-1*H*-pyrrol-3-yl)ethan-1-one (3h) in $CDCl_3$ ^{1}H-NMR and ^{13}C-NMR

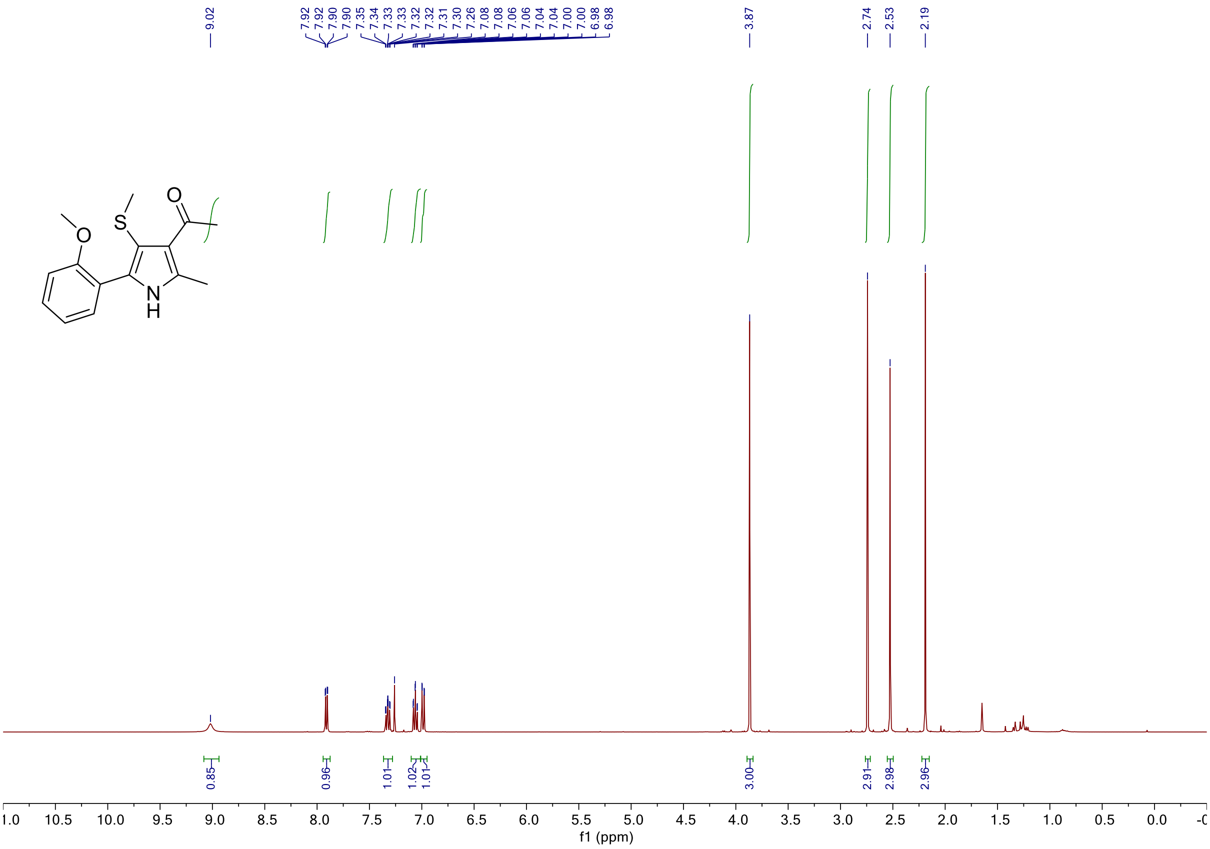

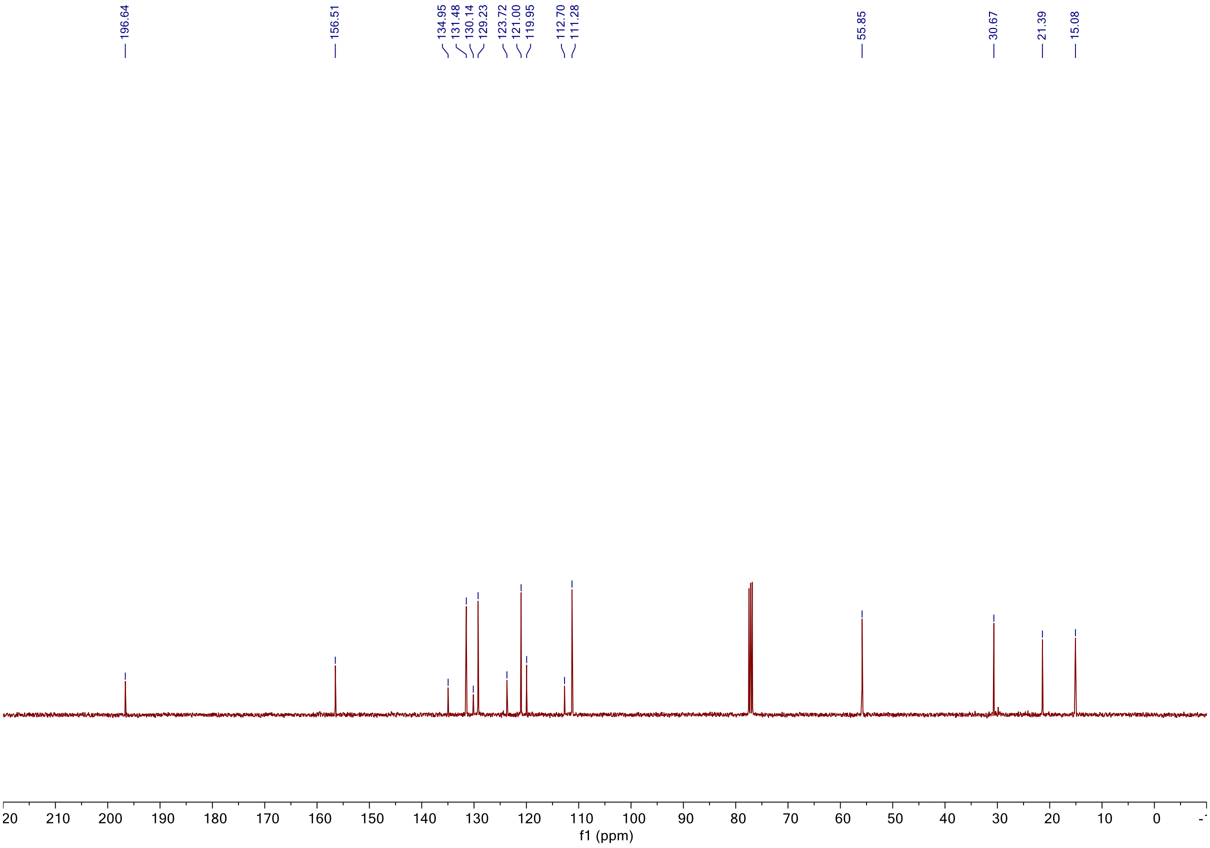

Ethyl 2-((4-acetyl-2-(2-methoxyphenyl)-5-methyl-1*H*-pyrrol-3-yl)thio)acetate (3i) in $CDCl_3$ ^{1}H-NMR and ^{13}C-NMR

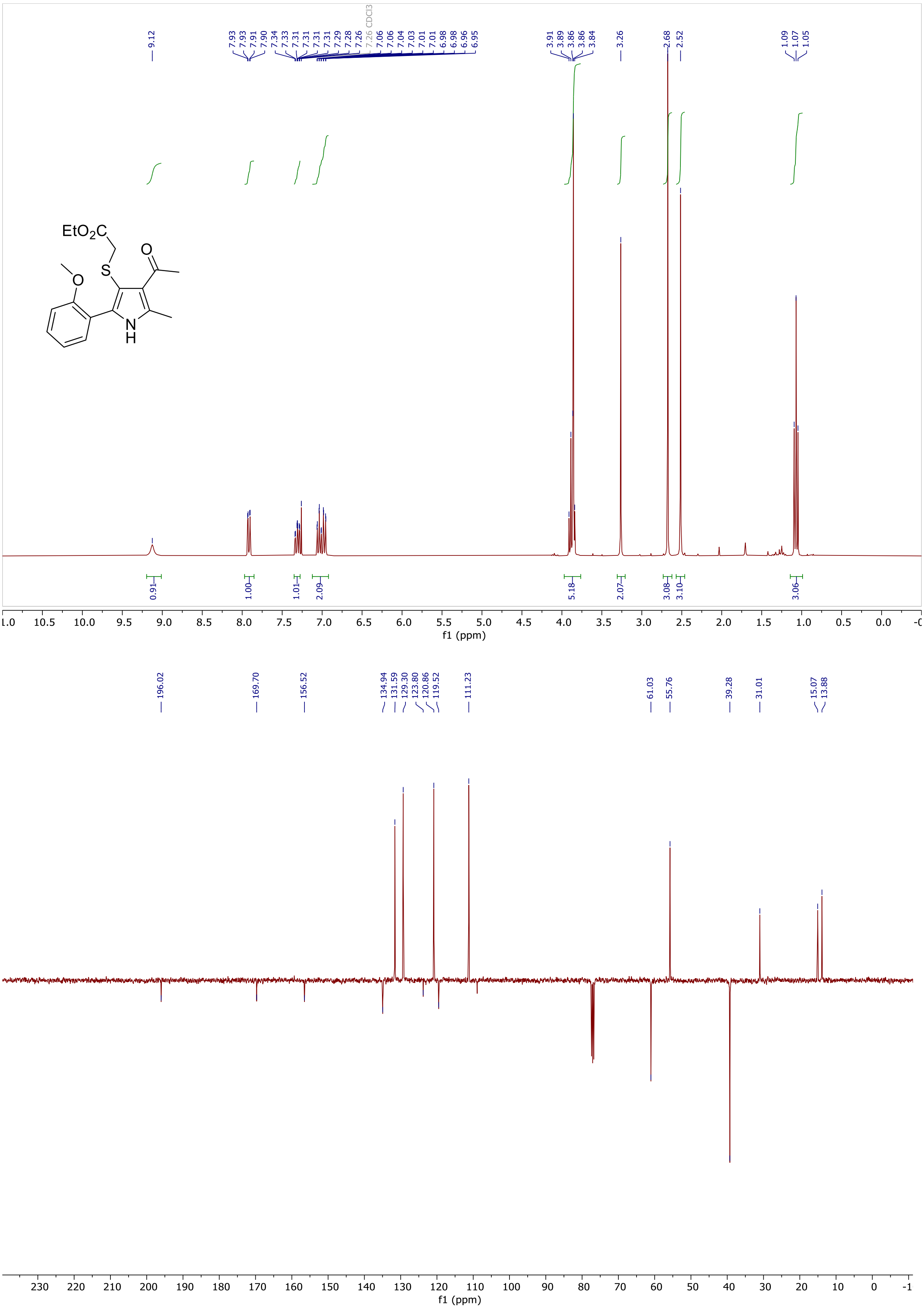

1-(5-Mesityl-2-methyl-4-(methylthio)-1*H*-pyrrol-3-yl)ethan-1-one (3j) in $CDCl_3$ ^1H-NMR and ^{13}C-NMR

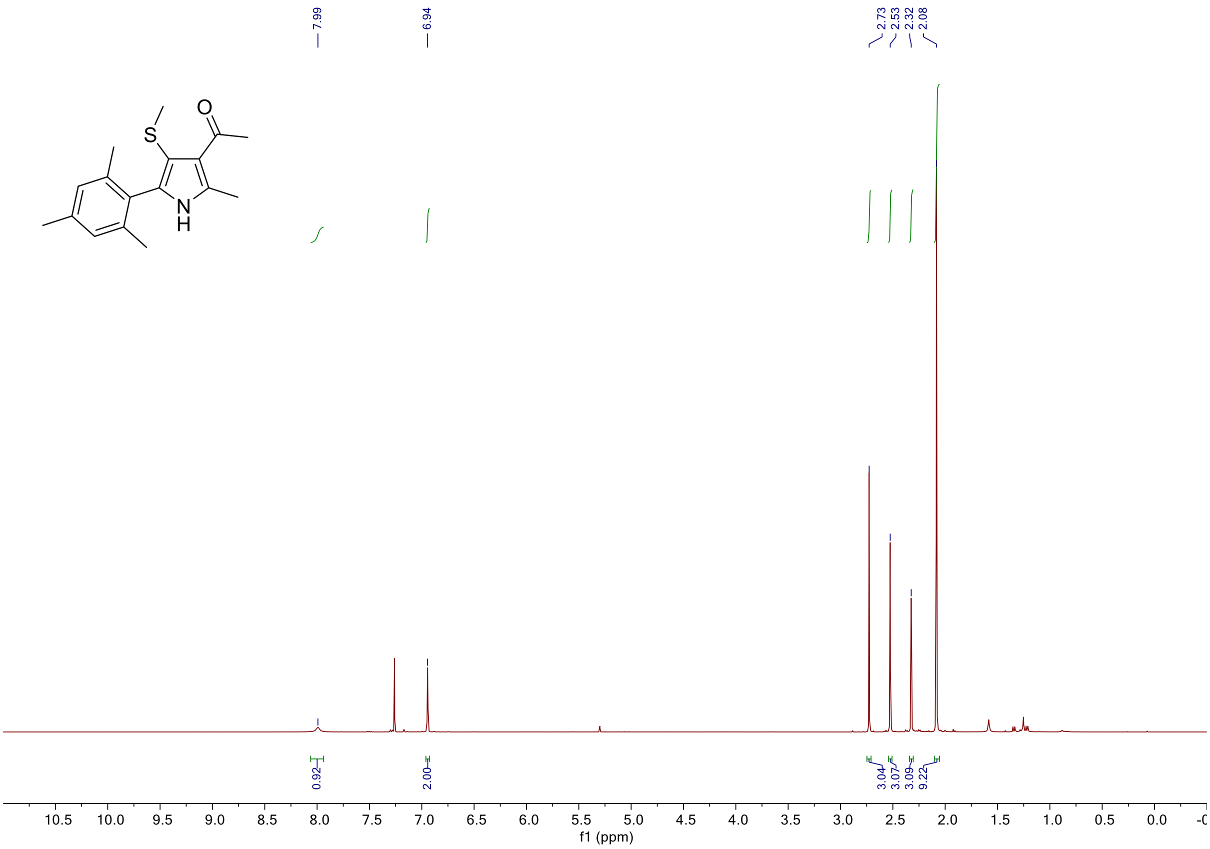

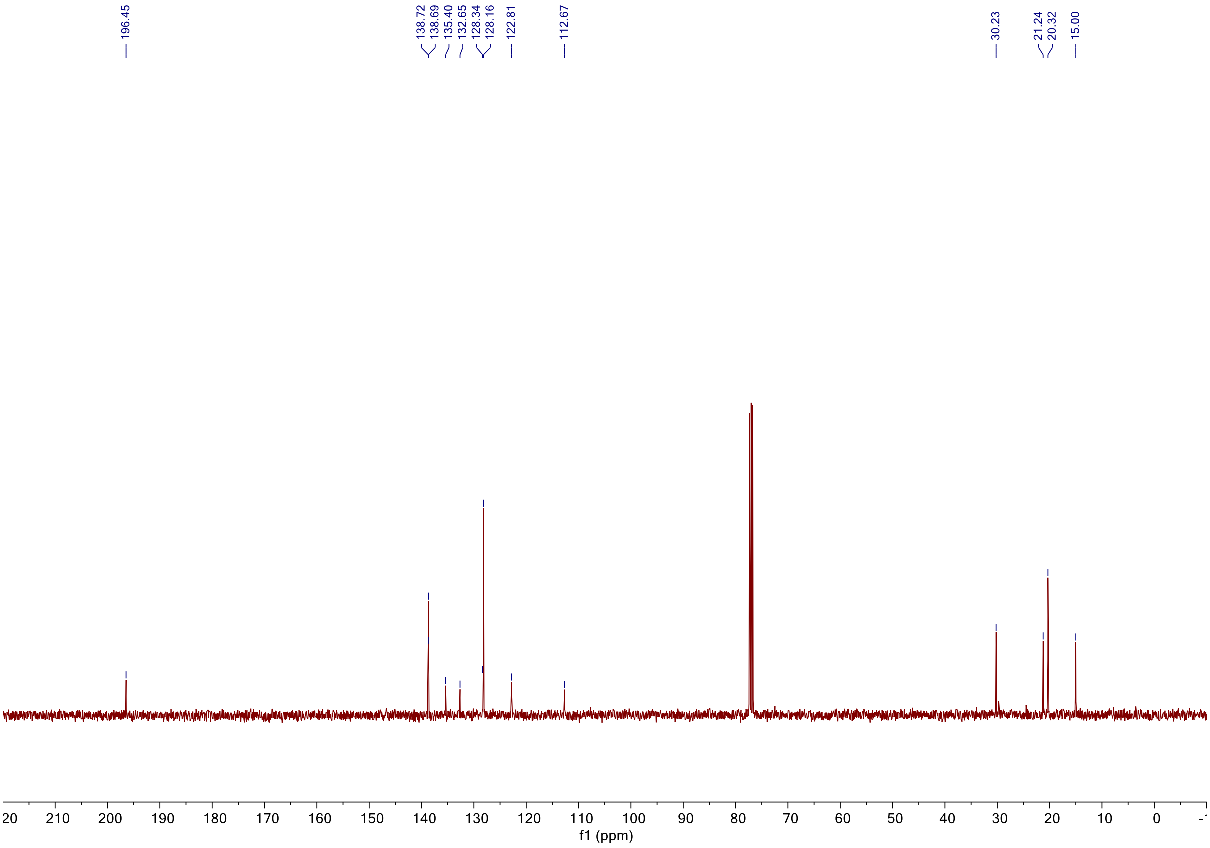

1-(2-Methyl-4-(methylthio)-5-(naphthalen-2-yl)-1*H*-pyrrol-3-yl)ethan-1-one (3k) in $CDCl_3$ ^1H-NMR and ^{13}C-NMR

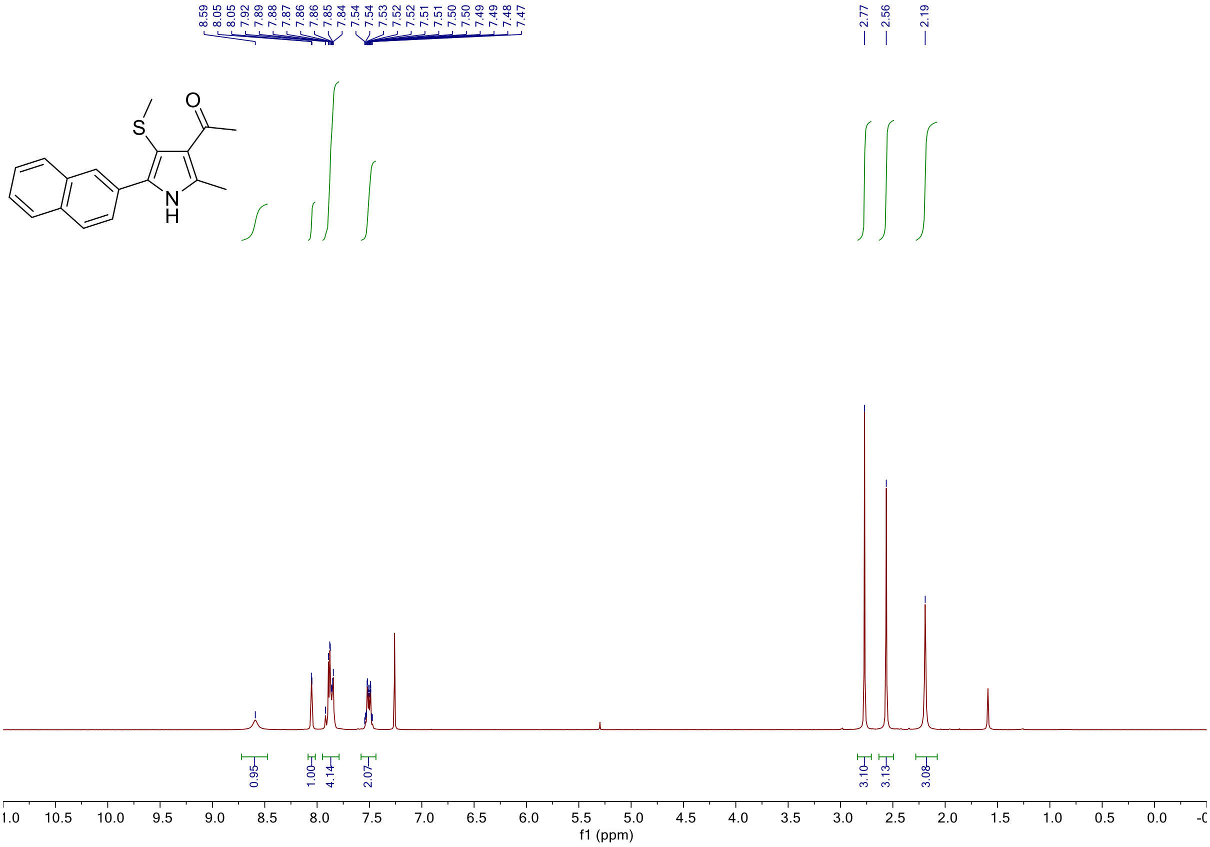

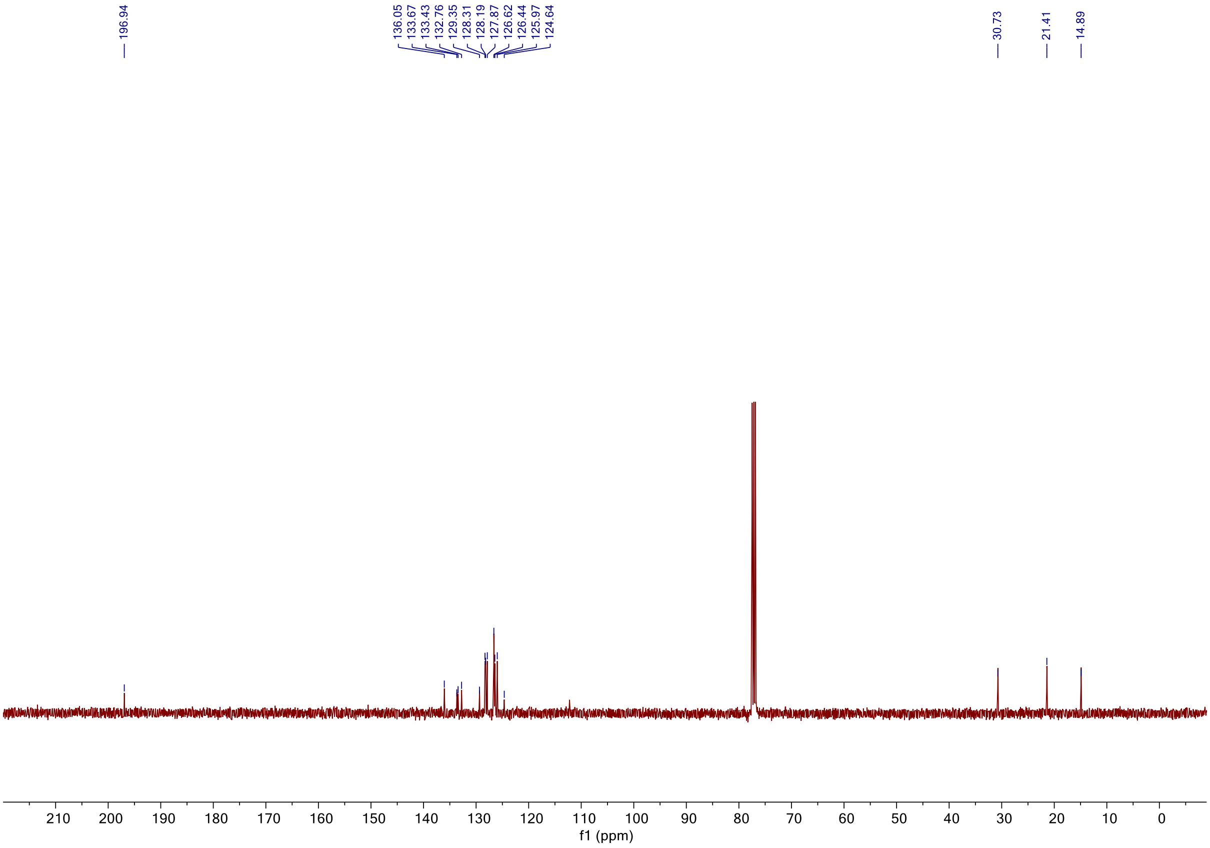

1-(2-Methyl-4-(methylthio)-5-phenyl-1*H*-pyrrol-3-yl)ethan-1-one (3l) in $CDCl_3$ ^{1}H-NMR and ^{13}C-NMR

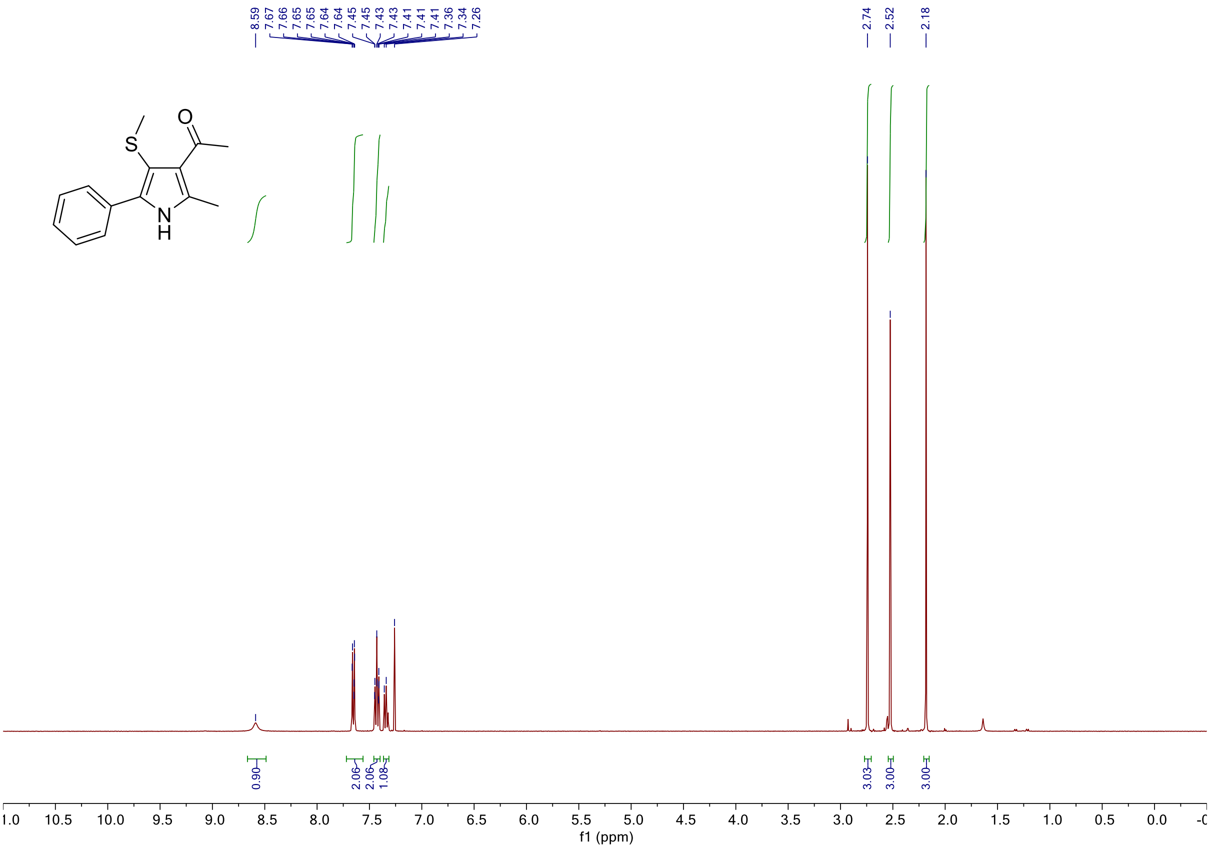

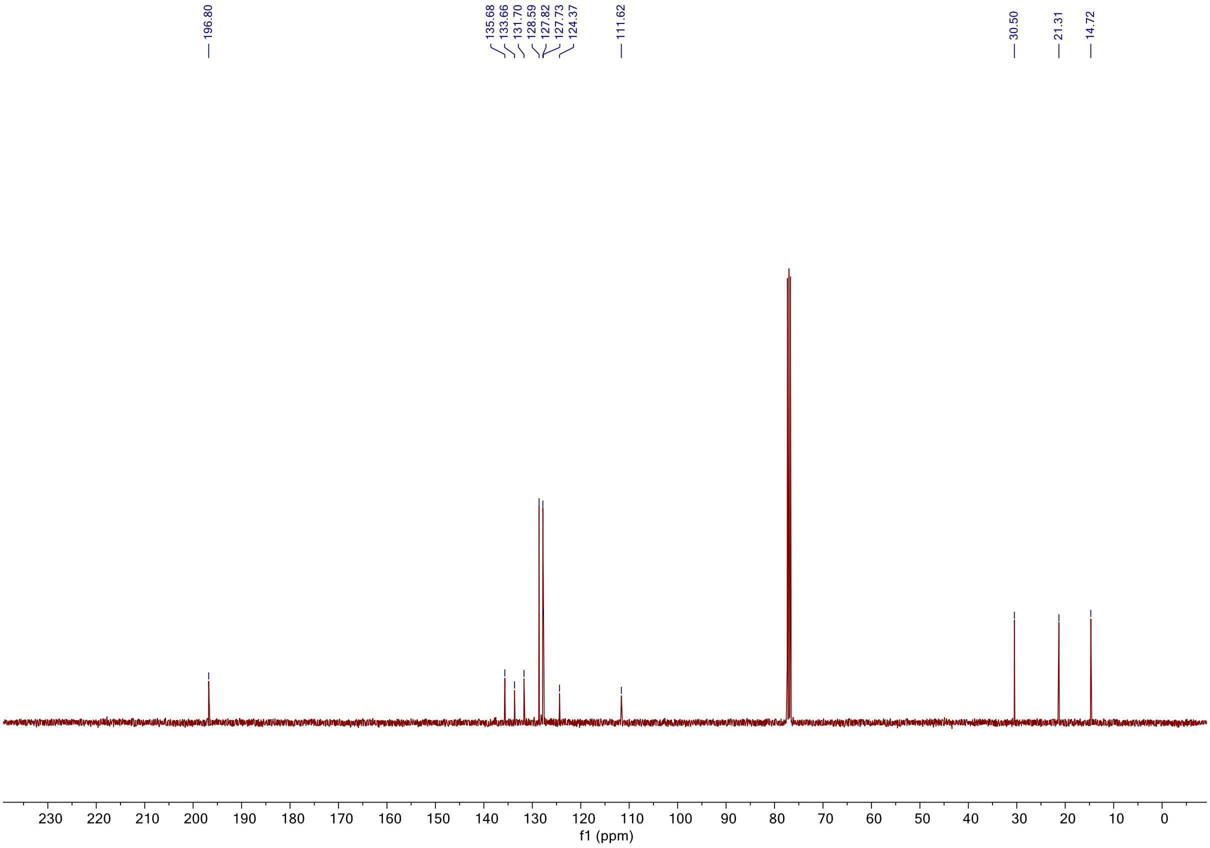

1-(5-(4-Fluorophenyl)-2-methyl-4-(methylthio)-1*H*-pyrrol-3-yl)ethan-1-one (3m) in $CDCl_3$ ^{1}H-NMR and ^{13}C-NMR

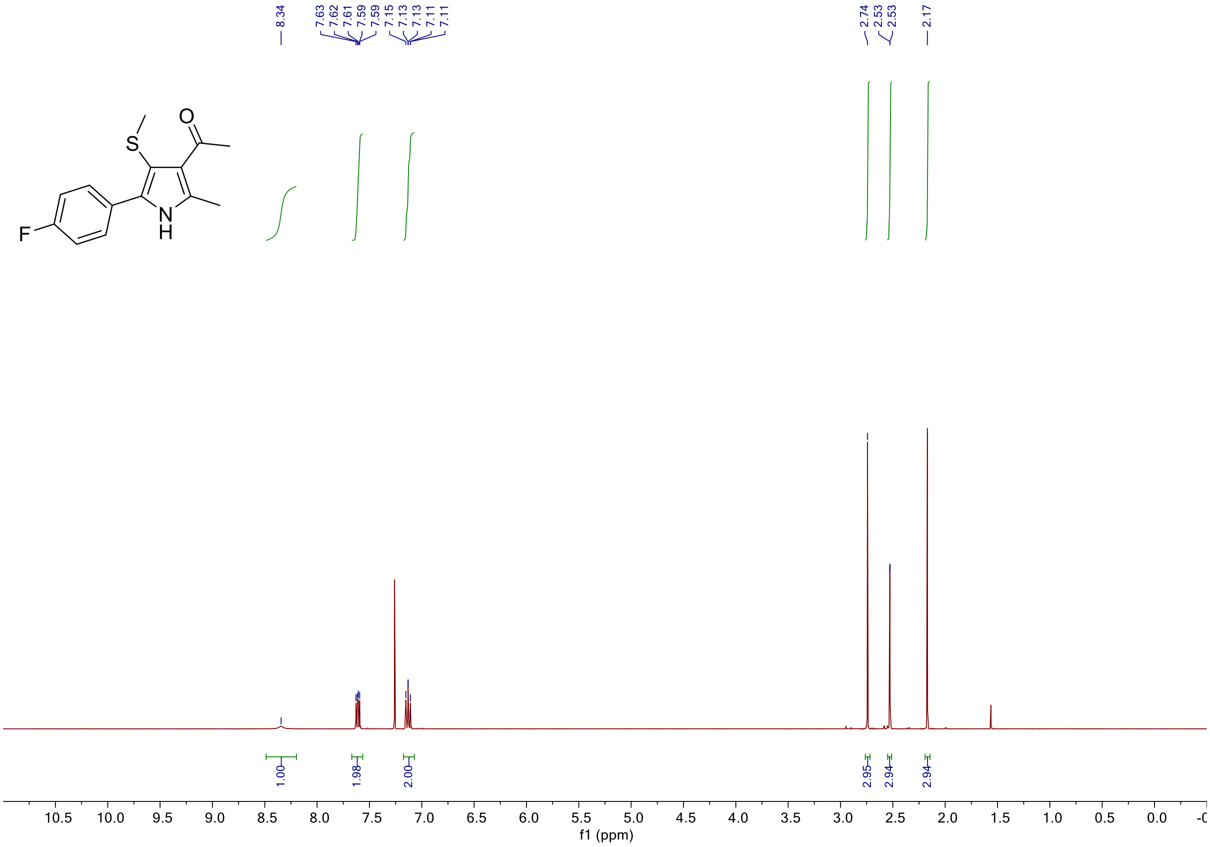

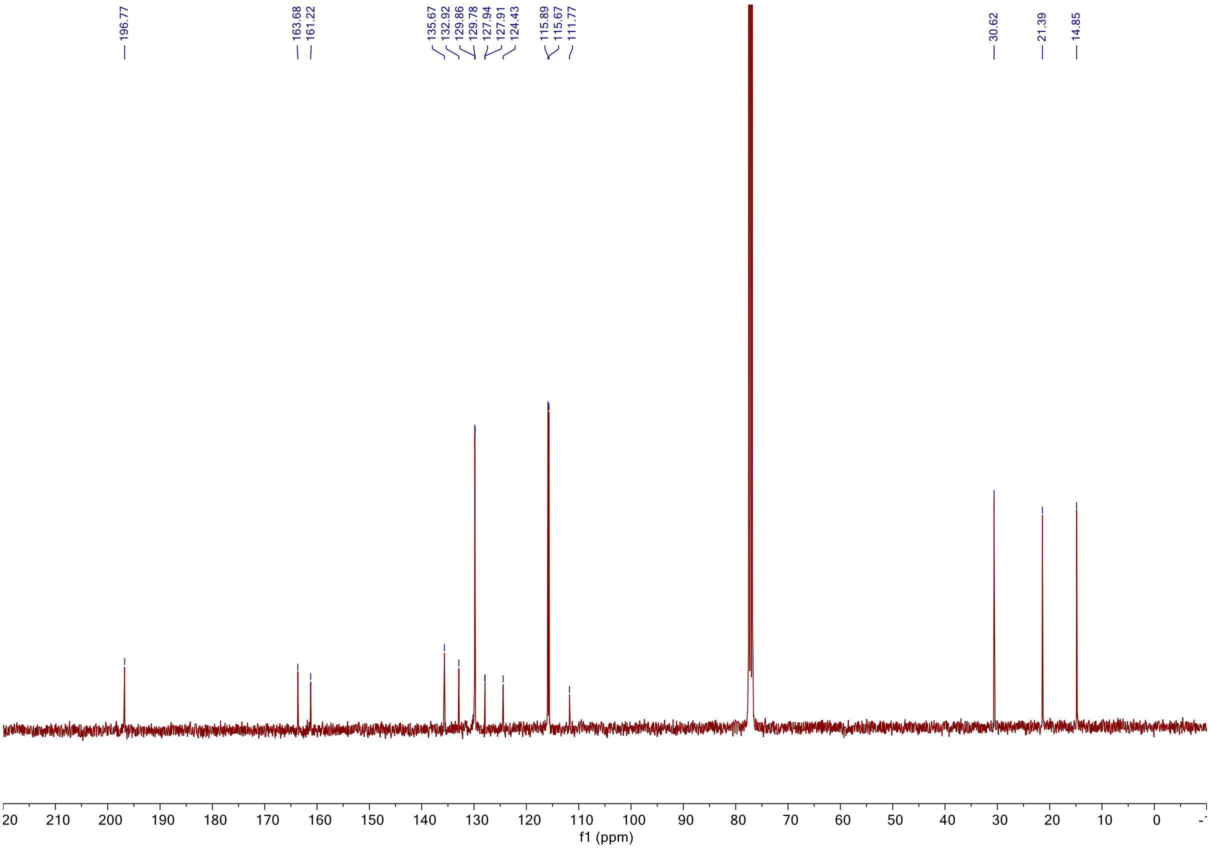

1-(5-(4-(Dimethylamino)phenyl)-2-methyl-4-(methylthio)-1*H*-pyrrol-3-yl)ethan-1-one (3n) in $CDCl_3$ ^{1}H-NMR and ^{13}C-NMR

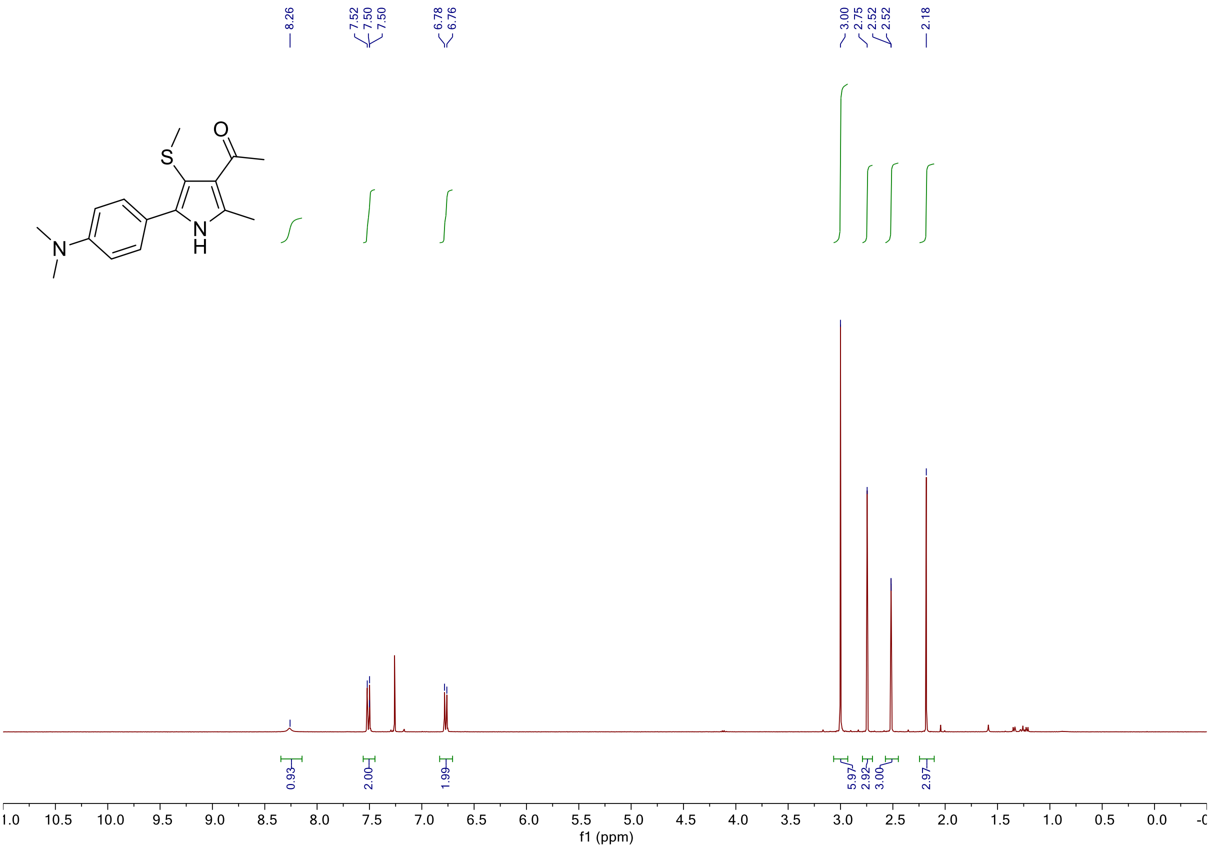

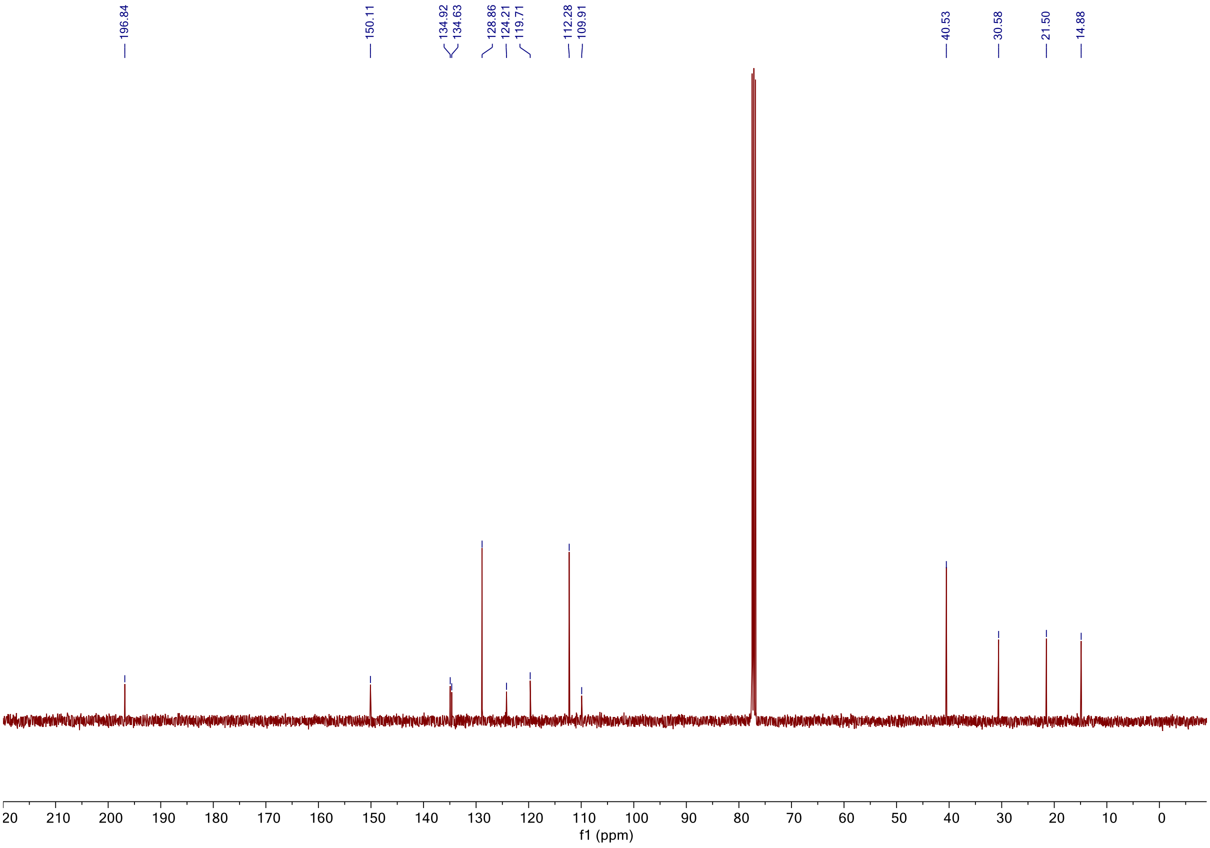

1-(2-Methyl-4-(methylthio)-5-(1-tosyl-1*H*-indol-3-yl)-1*H*-pyrrol-3-yl)ethan-1-one (3o) in $CDCl_3$

^1H-NMR and ^{13}C-NMR

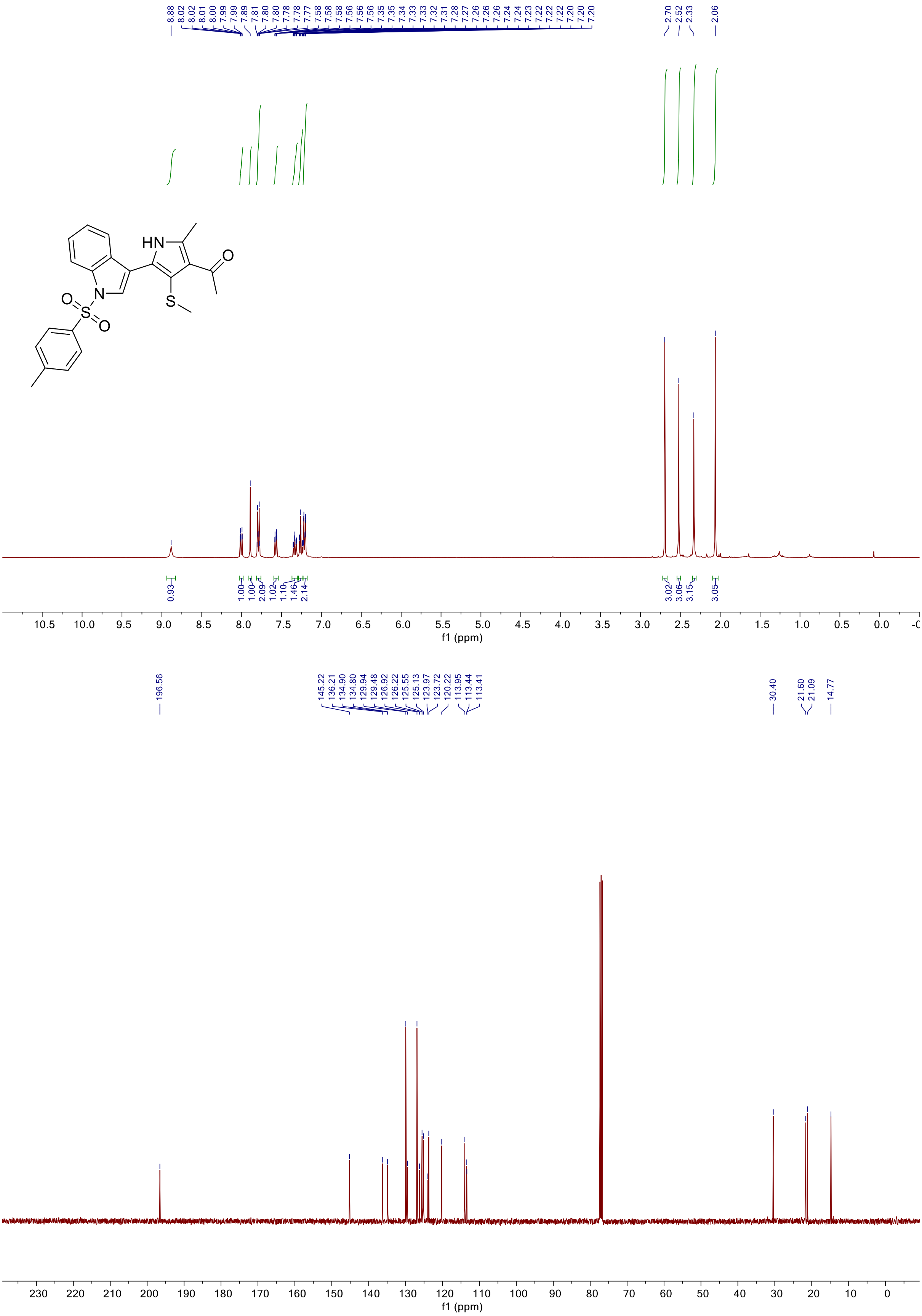

1-(2-Methyl-4-(methylthio)-5-(thiophen-2-yl)-1*H*-pyrrol-3-yl)ethan-1-one (3p) in $CDCl_3$ ^{1}H-NMR and ^{13}C-NMR

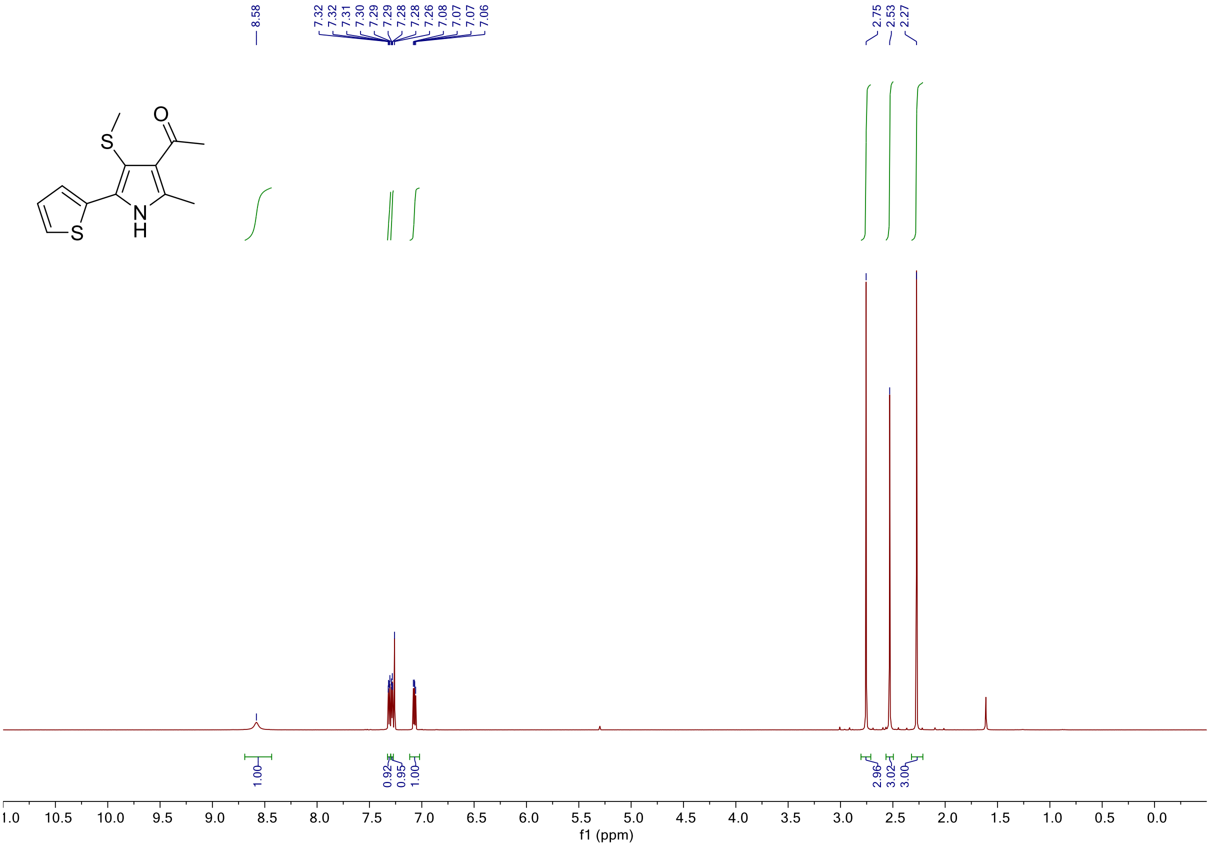

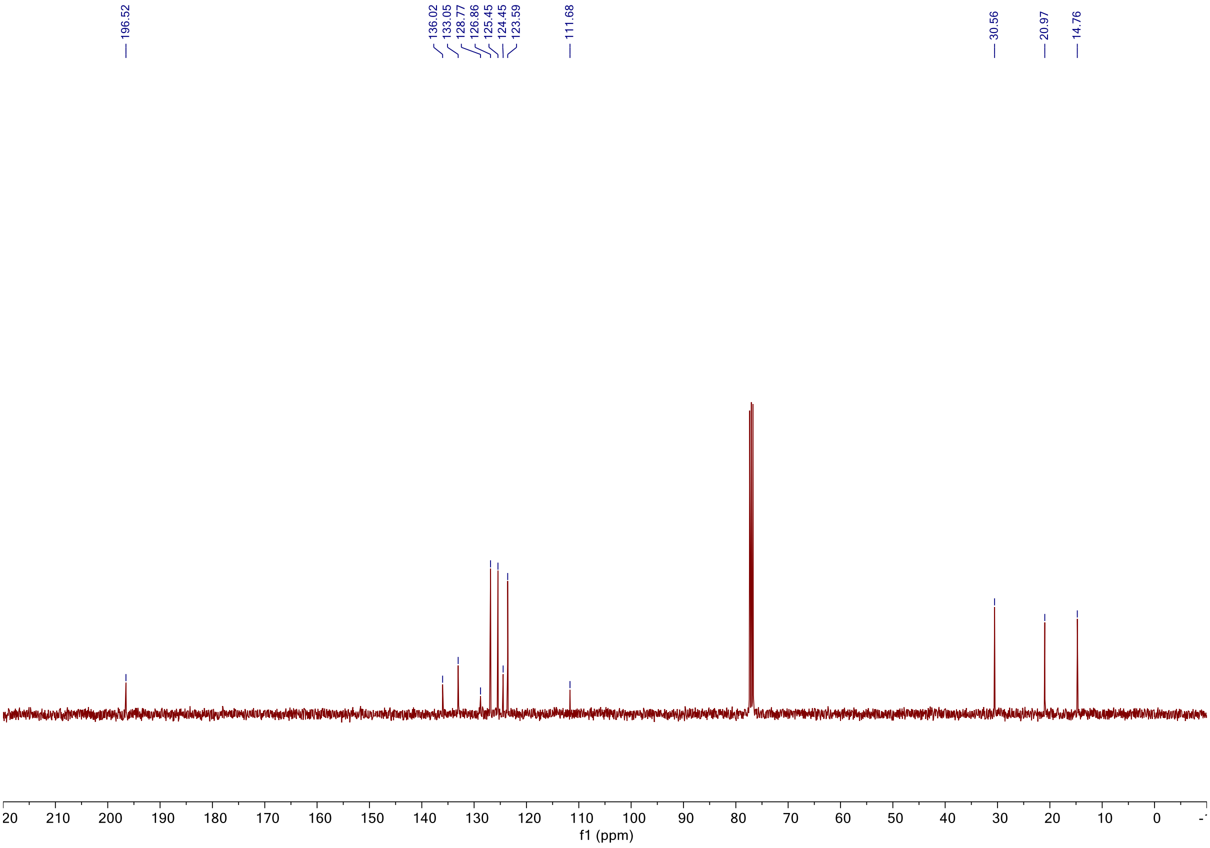

1-(5-(3,4-Dimethoxyphenyl)-4-((4-methoxybenzyl)thio)-2-methyl-1*H*-pyrrol-3-yl)ethan-1-one (3q) in CDCl$_3$ ^{1}H-NMR and ^{13}C-NMR

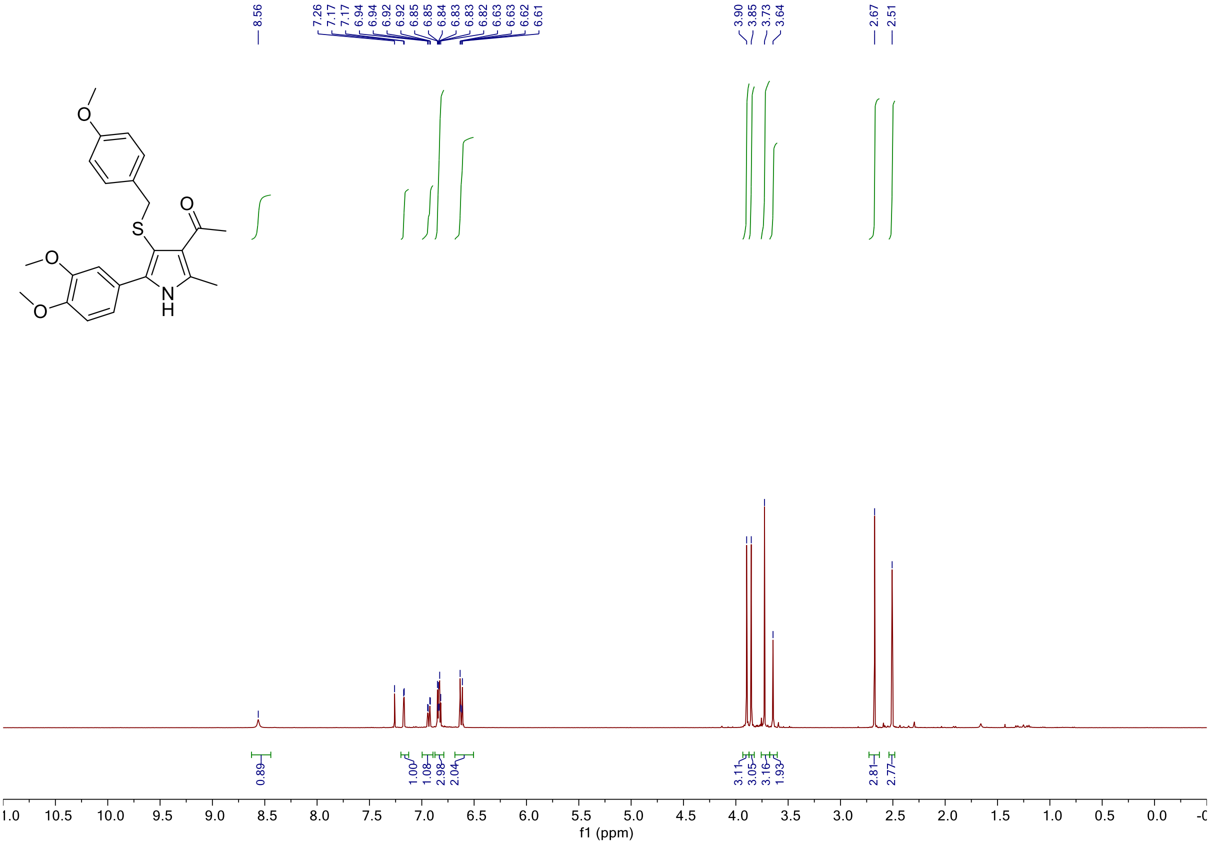

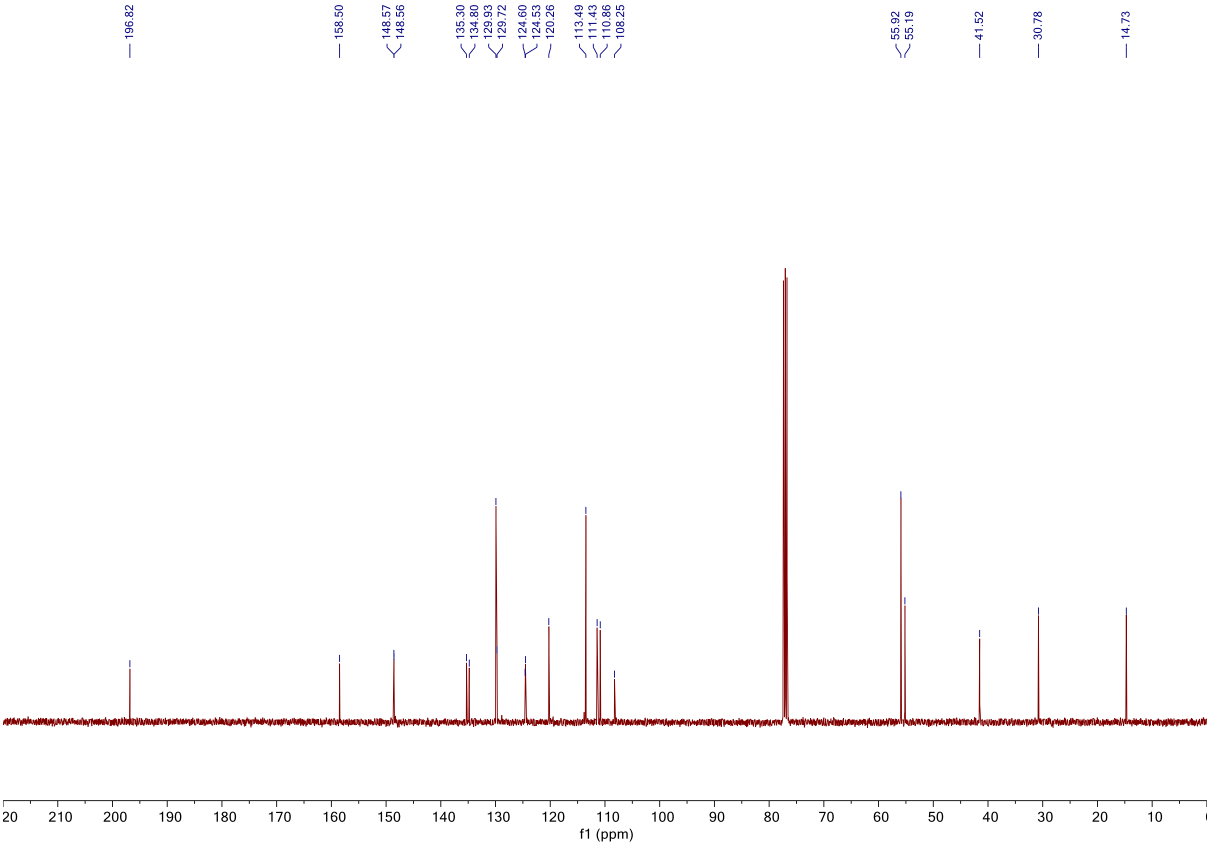

1-(5-Butyl-2-methyl-4-(methylthio)-1*H*-pyrrol-3-yl)ethan-1-one (3r) in $CDCl_3$ ^{1}H-NMR and ^{13}C-NMR

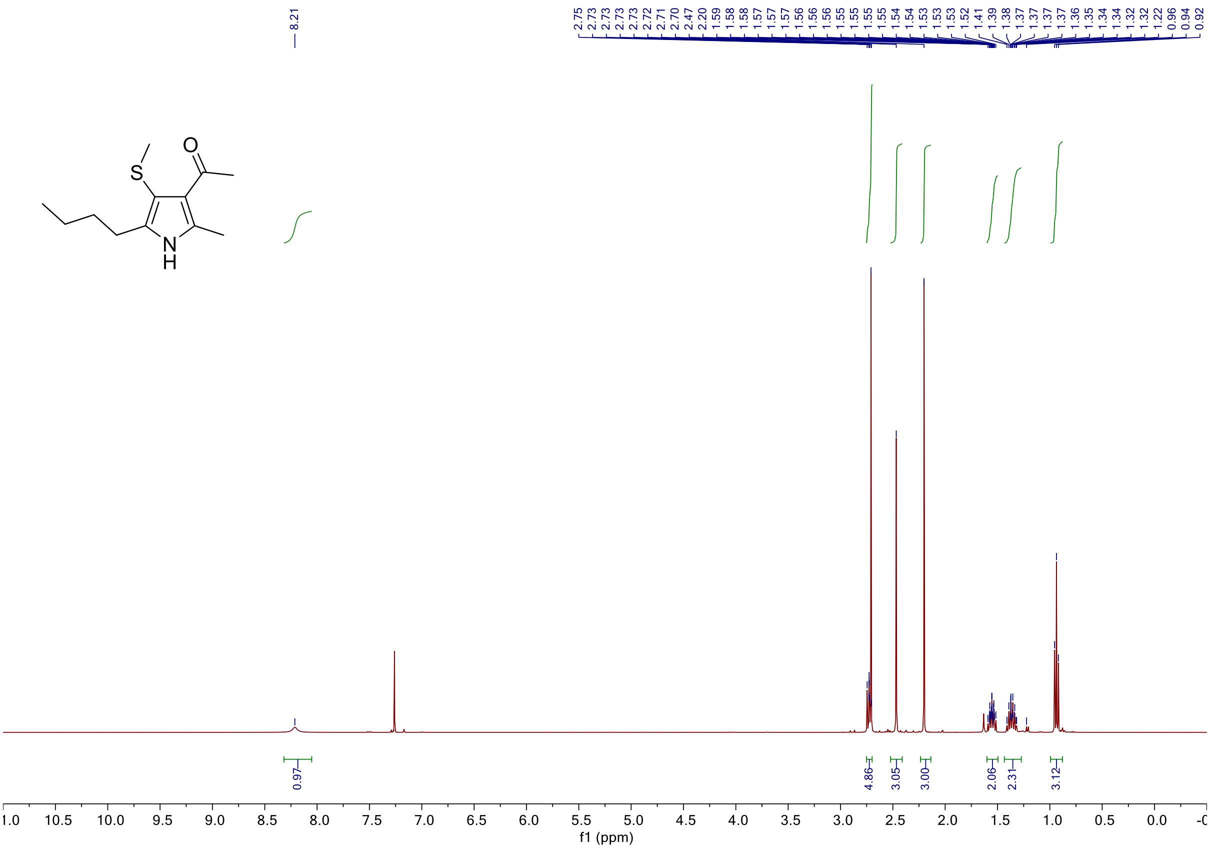

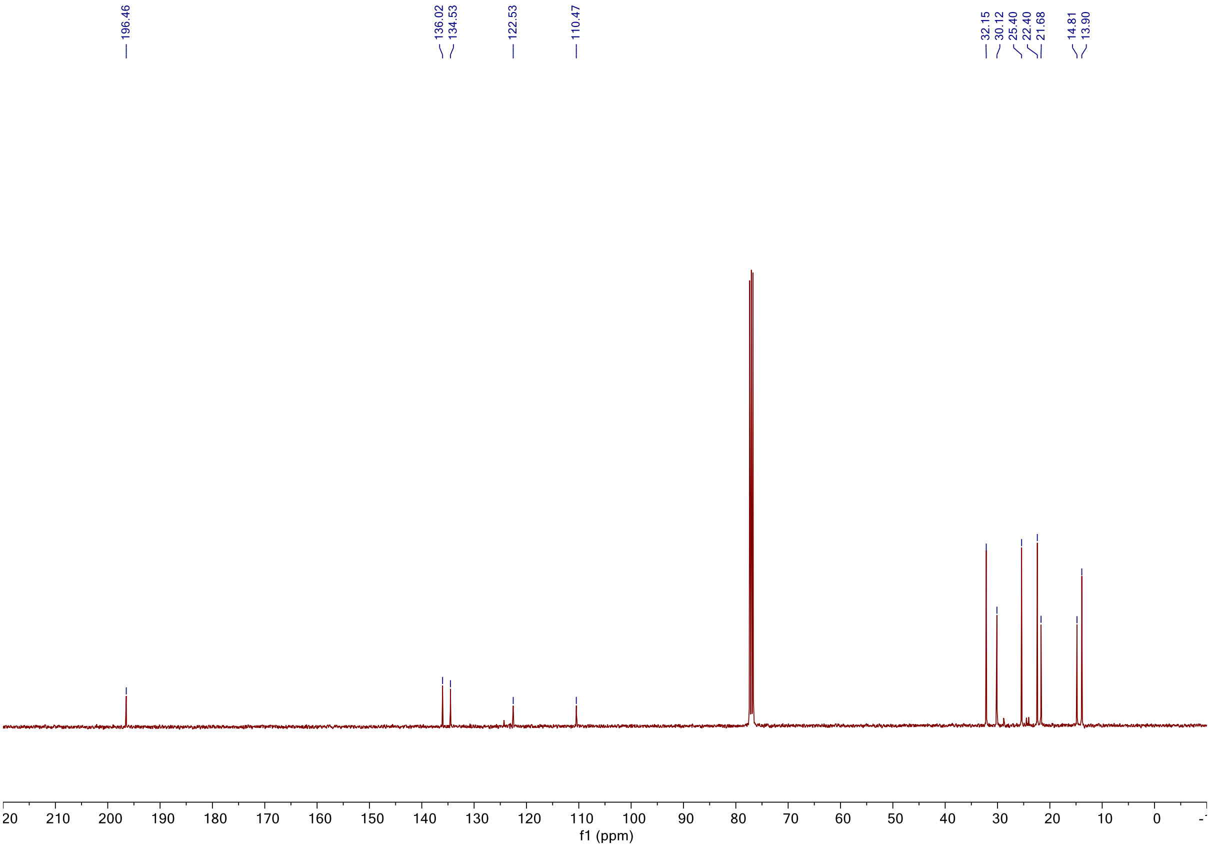

1-(5-Cyclopropyl-2-methyl-4-(methylthio)-1*H*-pyrrol-3-yl)ethan-1-one (3s) in $CDCl_3$ ^{1}H-NMR and ^{13}C-NMR

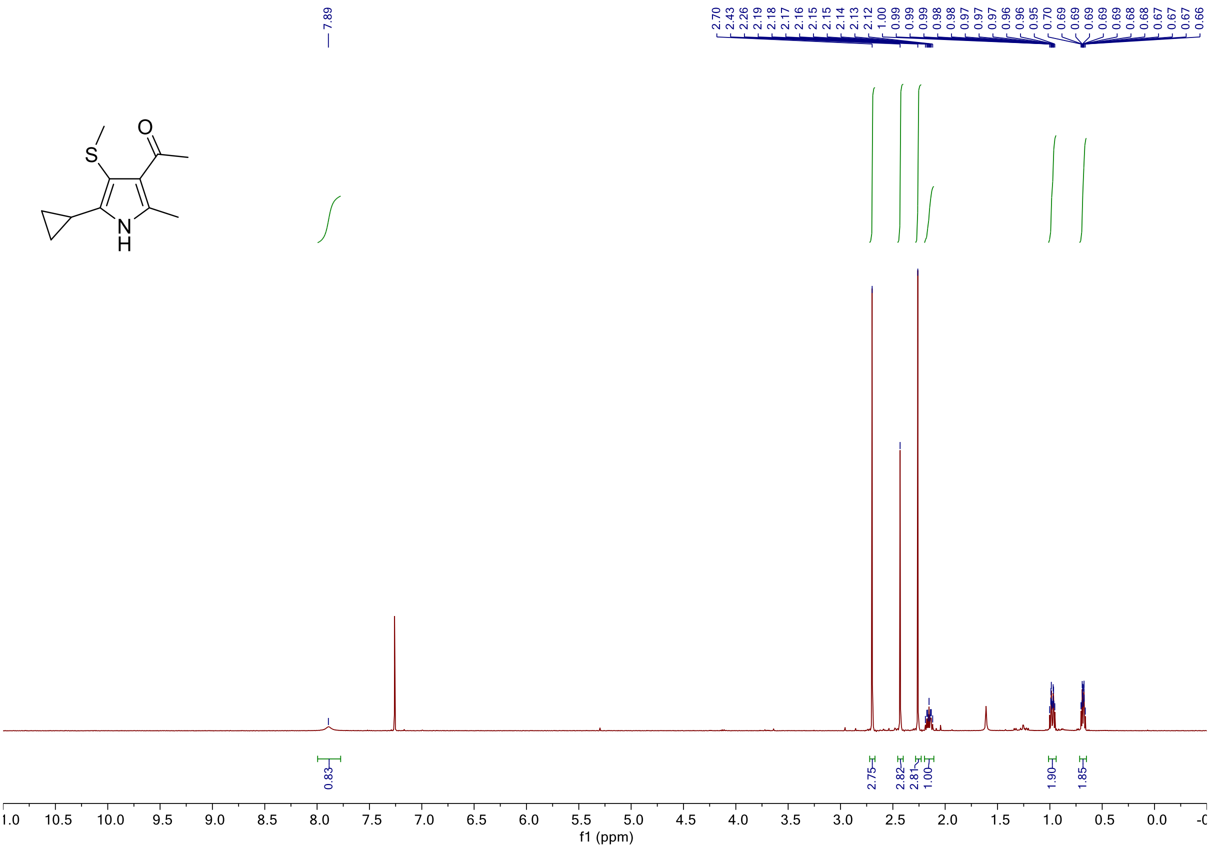

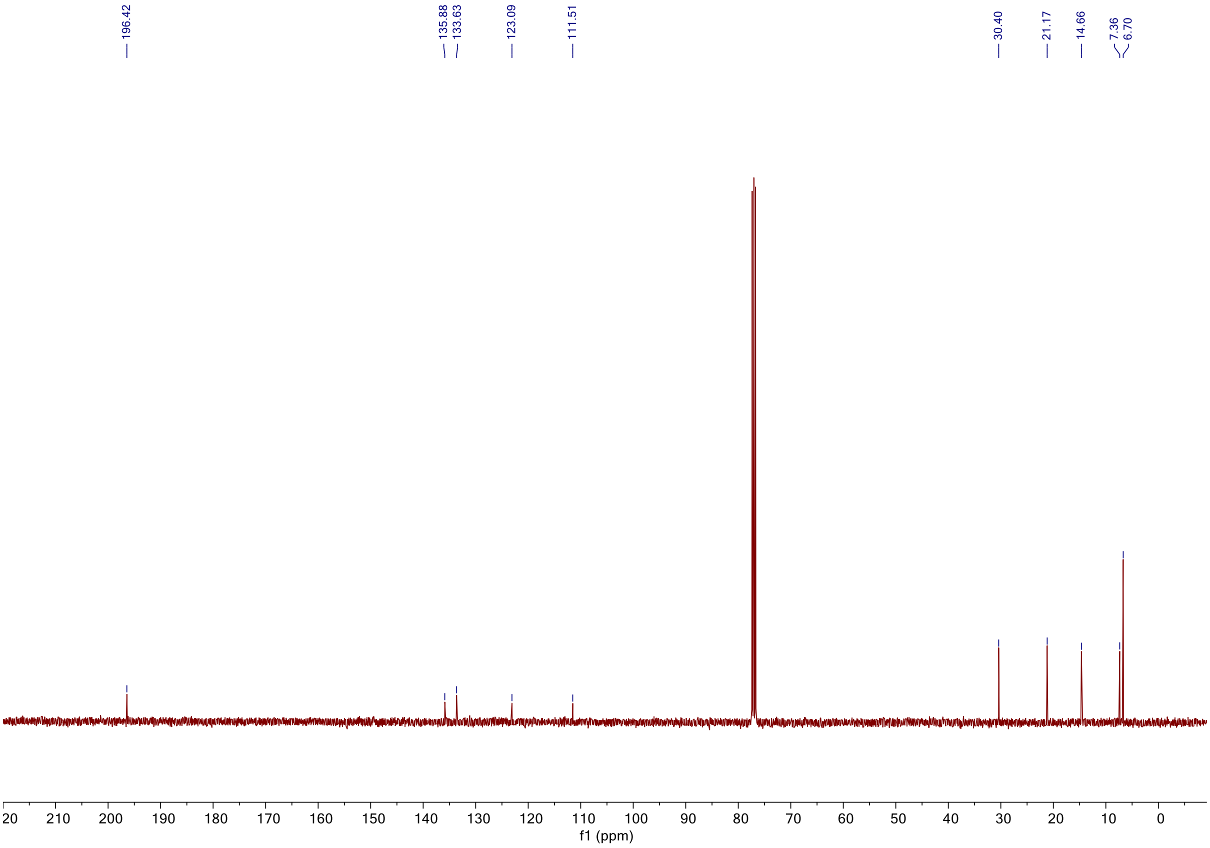

1-(2-Methyl-4-(methylthio)-5-(phenylthio)-1*H*-pyrrol-3-yl)ethan-1-one (3t) in $CDCl_3$ ^{1}H-NMR and ^{13}C-NMR

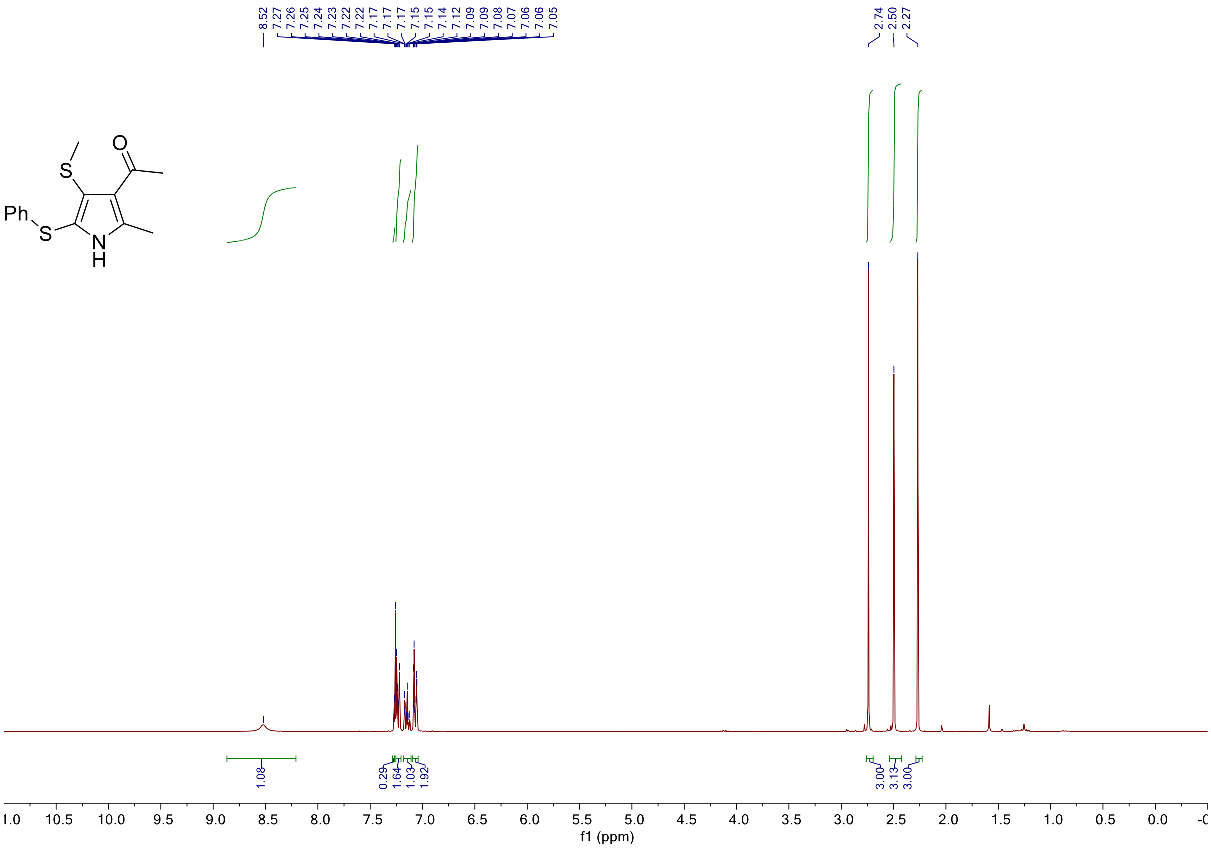

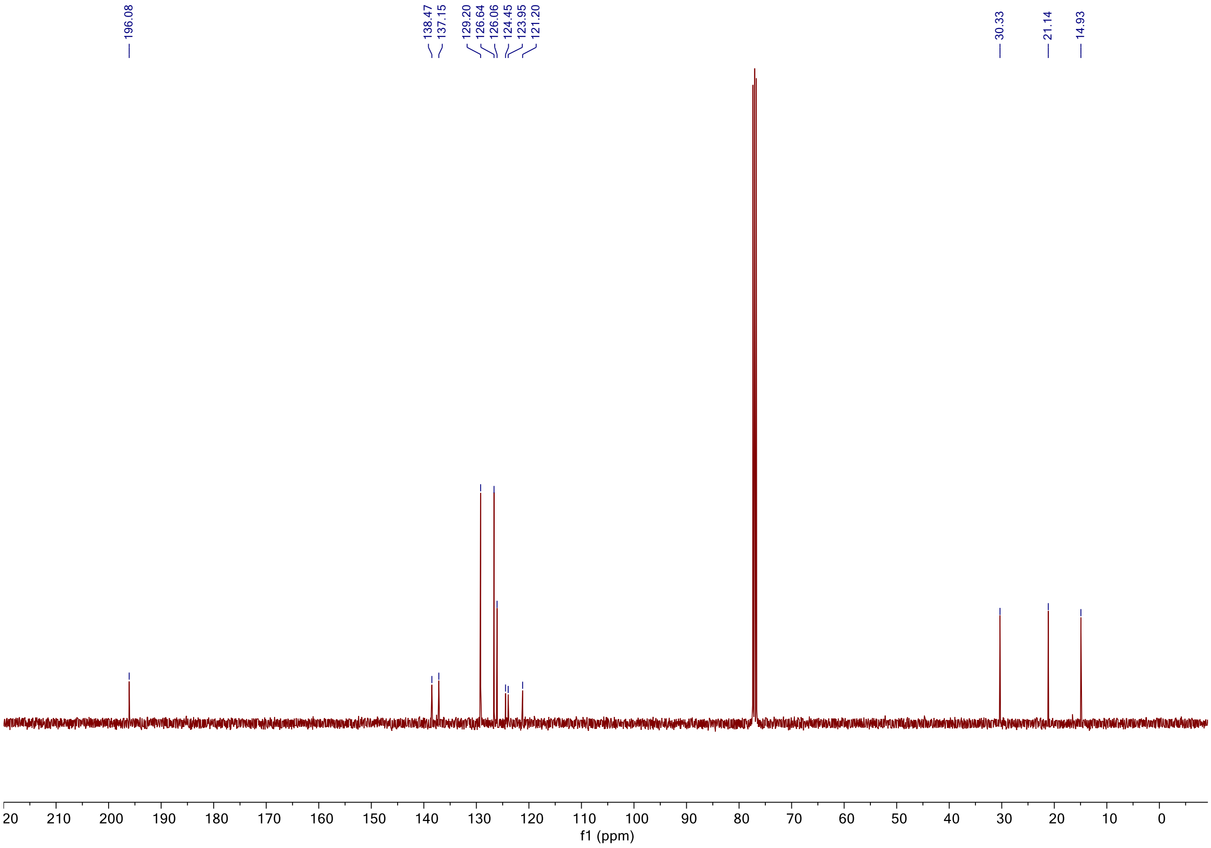

^{1}H-^{1}H NOESY, and ^{1}H-^{1}H NOESY (full and selected region) annotated with diagnostic assignments of 3t.

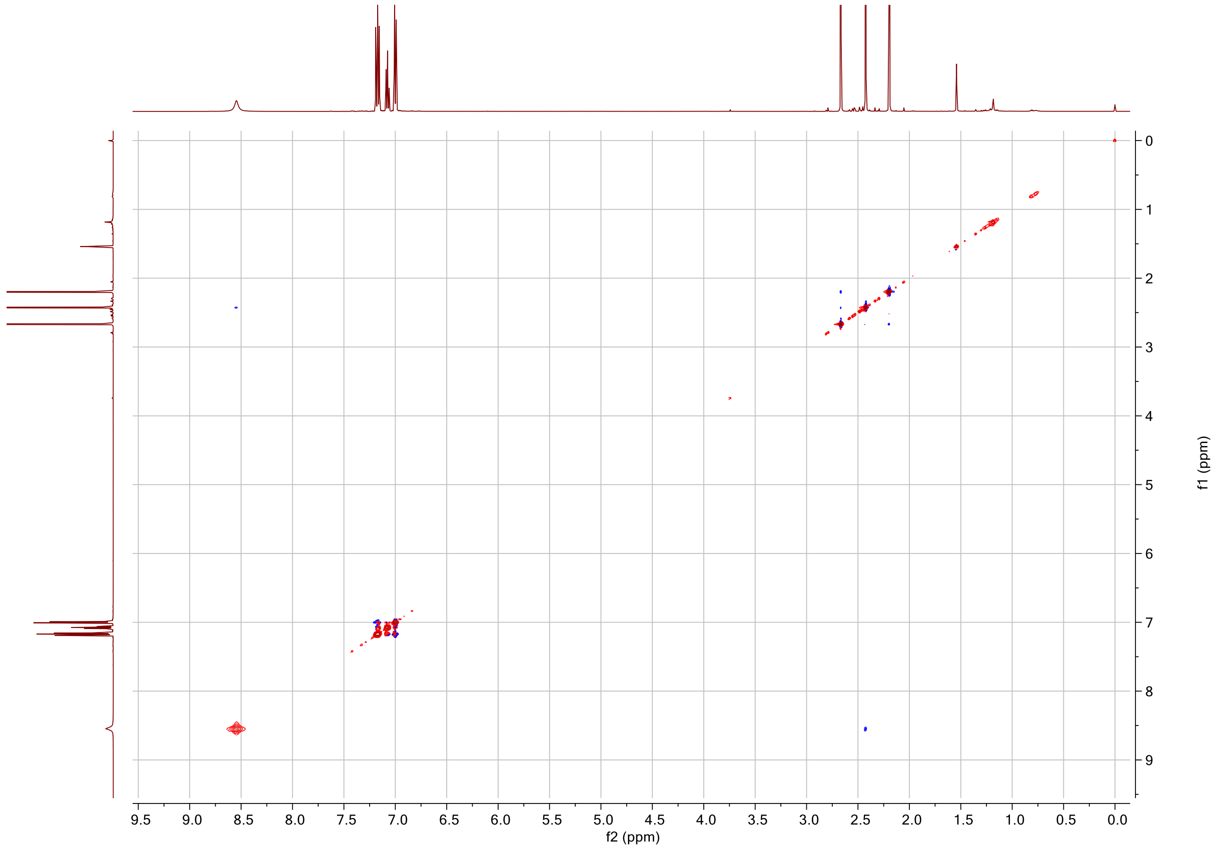

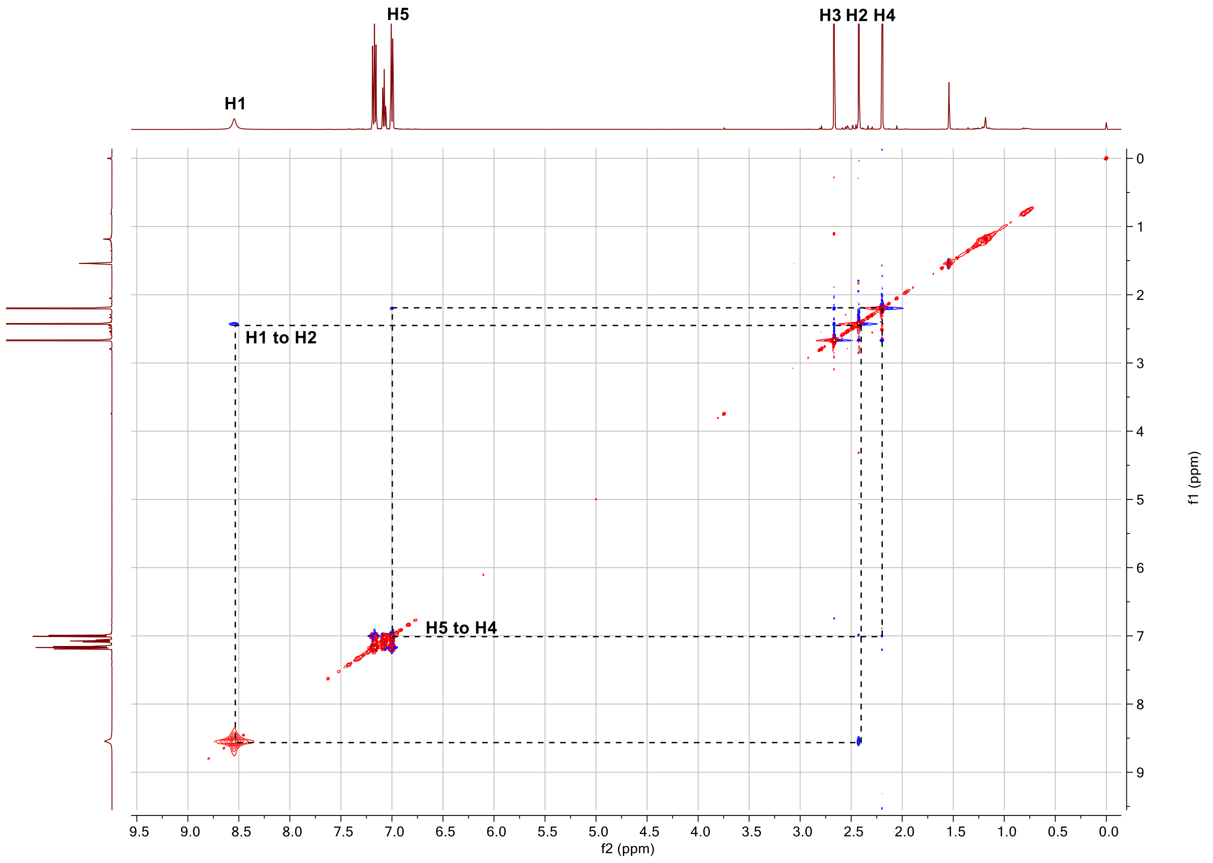

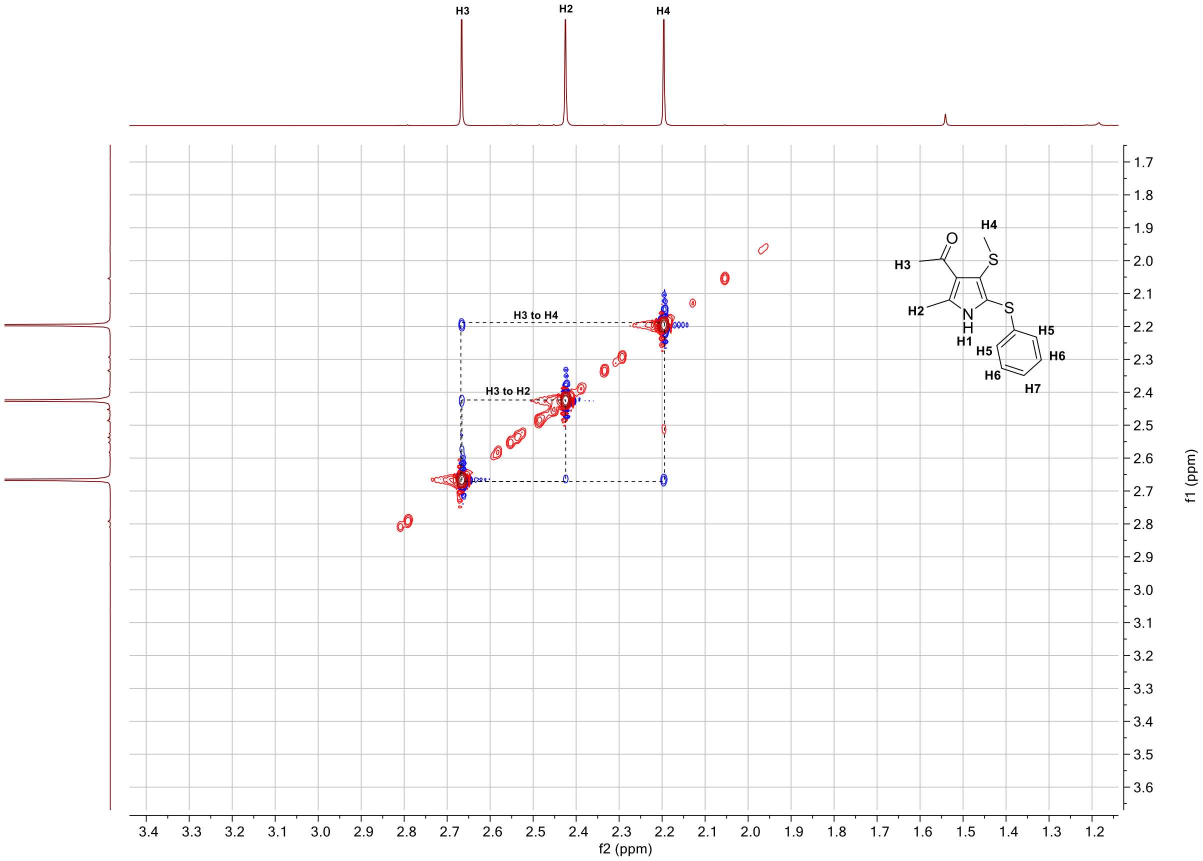
H3
H2
H4
H3 to H4
H3 to H2
O
S
S
N
H
H1
H5
H6
H7
f1 (ppm)
f2 (ppm)

1-(2-Methyl-5-(methylthio)-4-(phenylthio)-1*H*-pyrrol-3-yl)ethan-1-one (3t') in $CDCl_3$ ^{1}H-NMR and ^{13}C-NMR

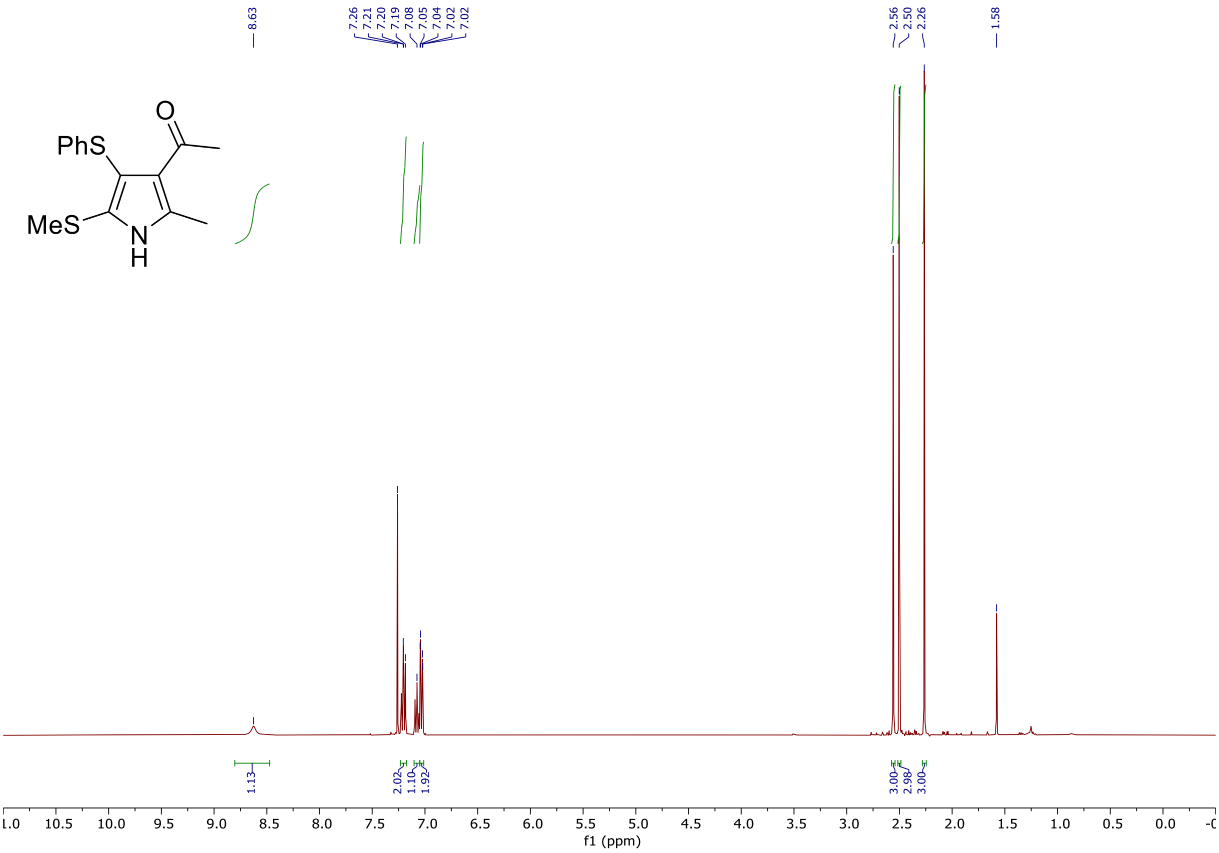

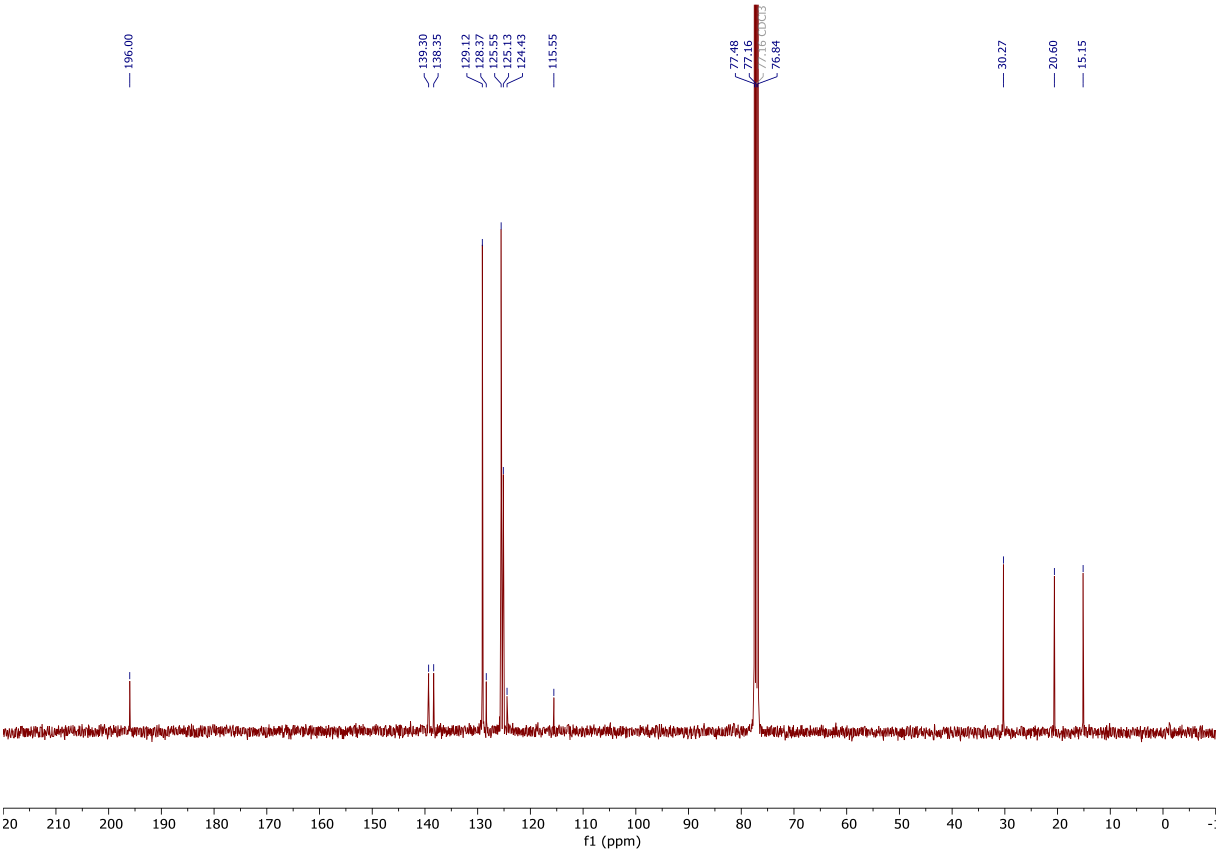

(5-(4-Methoxyphenyl)-4-(methylthio)-2-phenyl-1*H*-pyrrol-3-yl)(phenyl)methanone (3u) in $CDCl_3$

^{1}H-NMR and ^{13}C-NMR

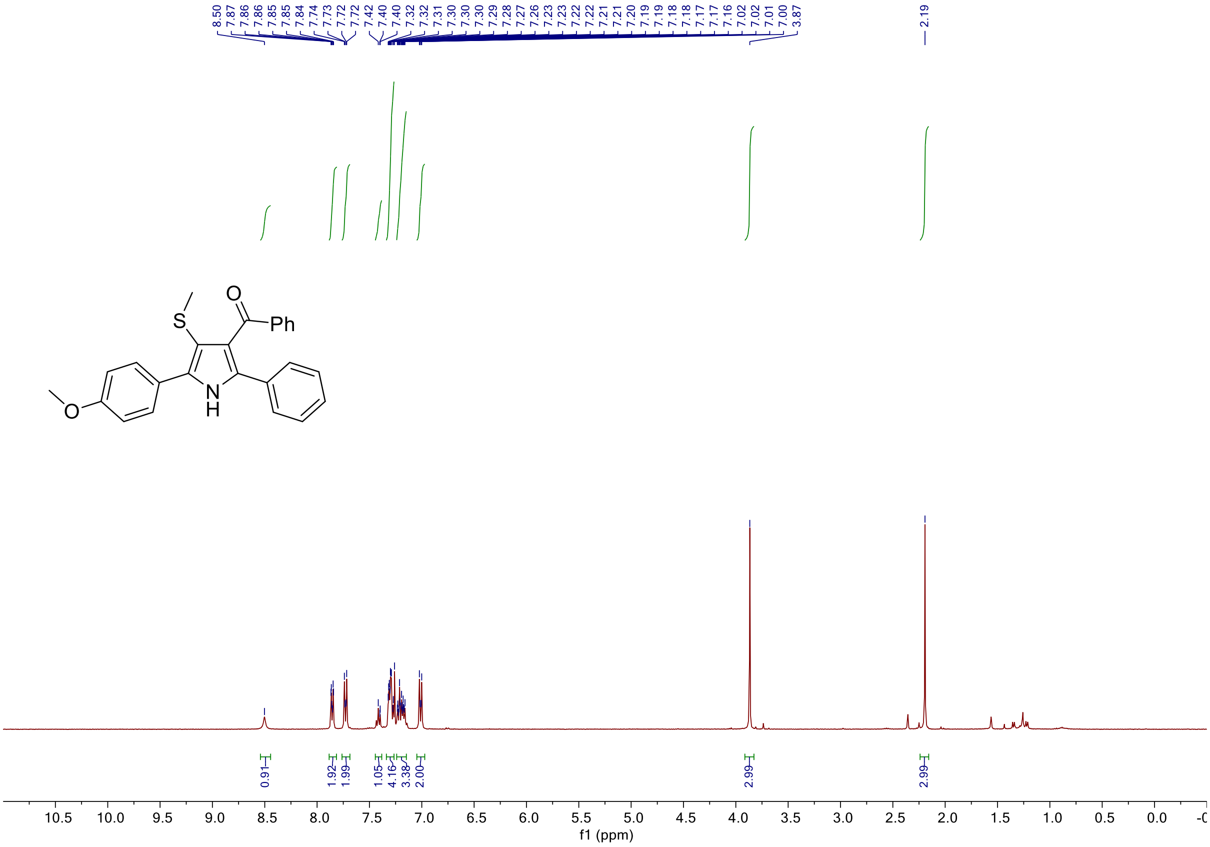

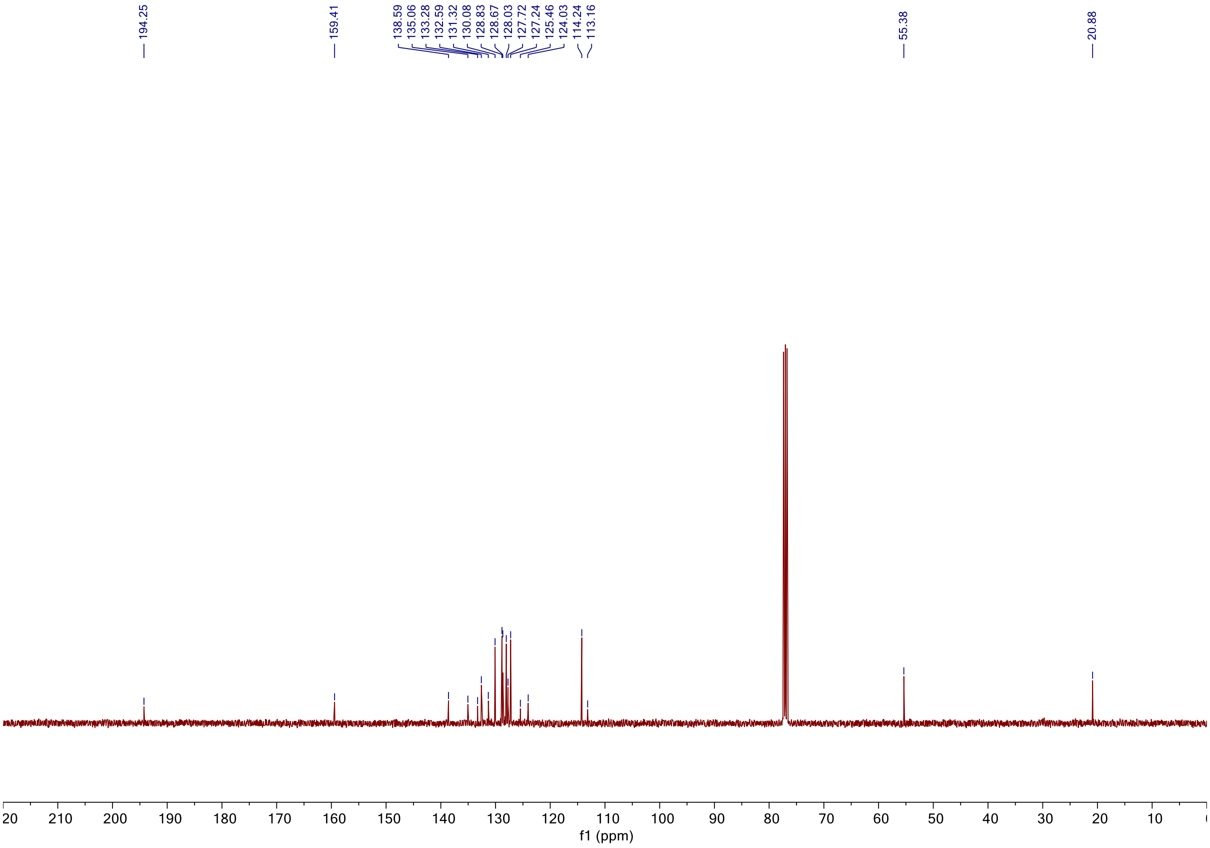

(5-(4-Methoxyphenyl)-4-(methylthio)-2-phenyl-1*H*-pyrrol-3-yl)(phenyl)methanone (3v) in $CDCl_3$

^{1}H-NMR and ^{13}C-NMR

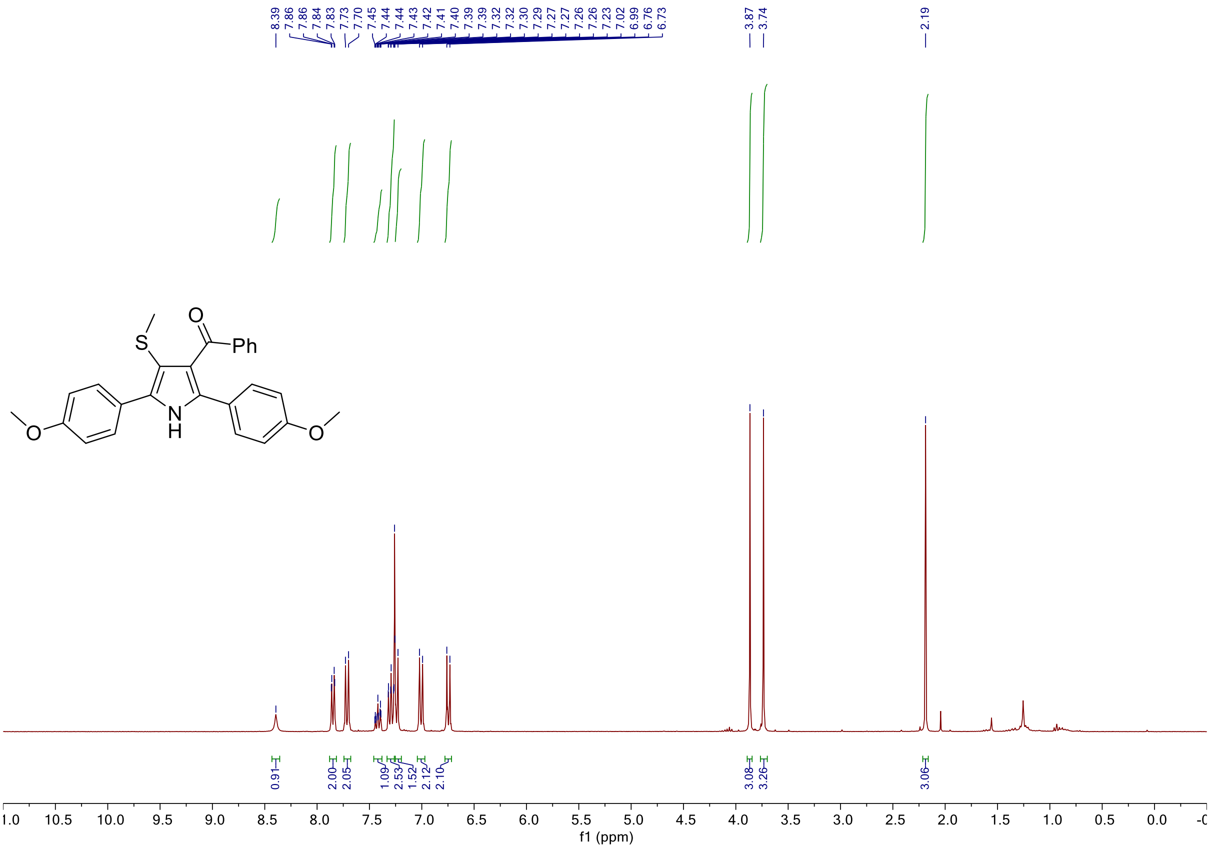

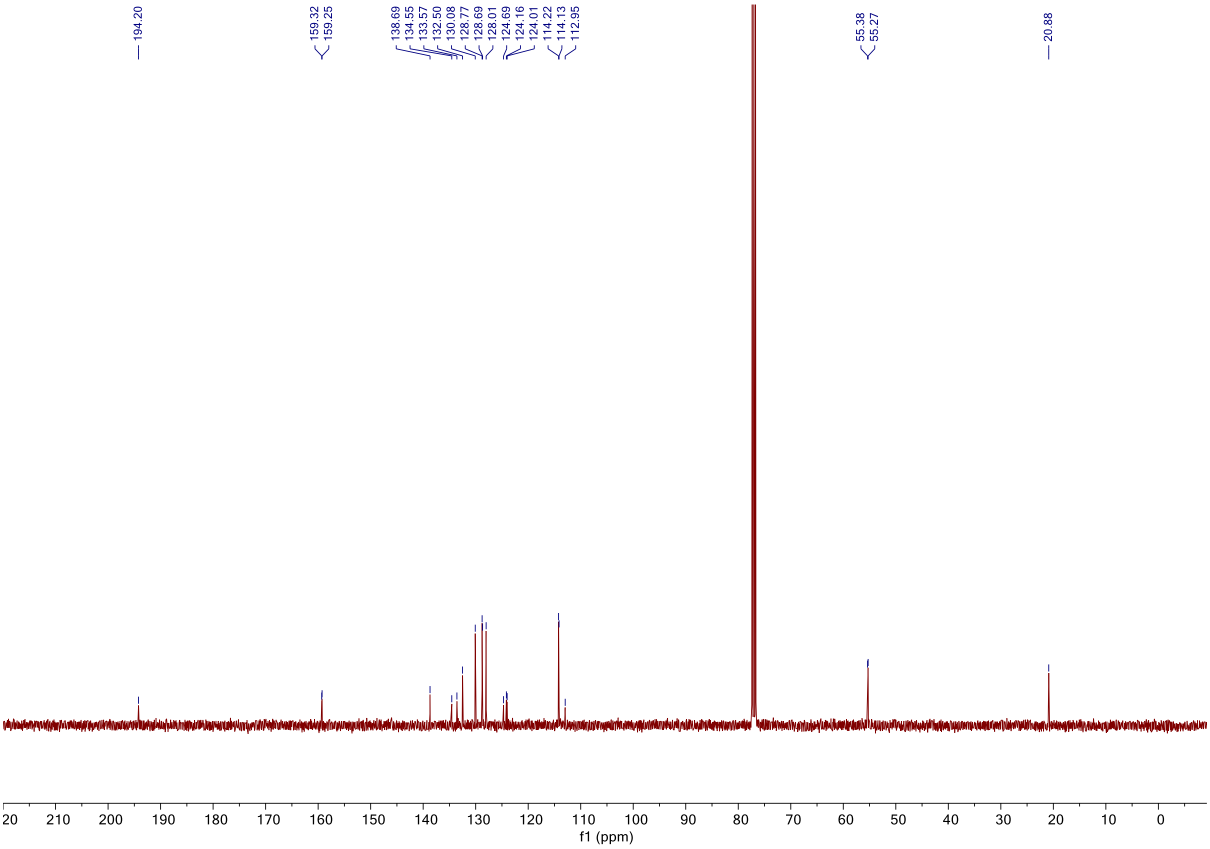

1-(2,5-Bis(4-methoxyphenyl)-4-(methylthio)-1*H*-pyrrol-3-yl)pentan-1-one (3w) in $CDCl_3$ ^{1}H-NMR and ^{13}C-NMR

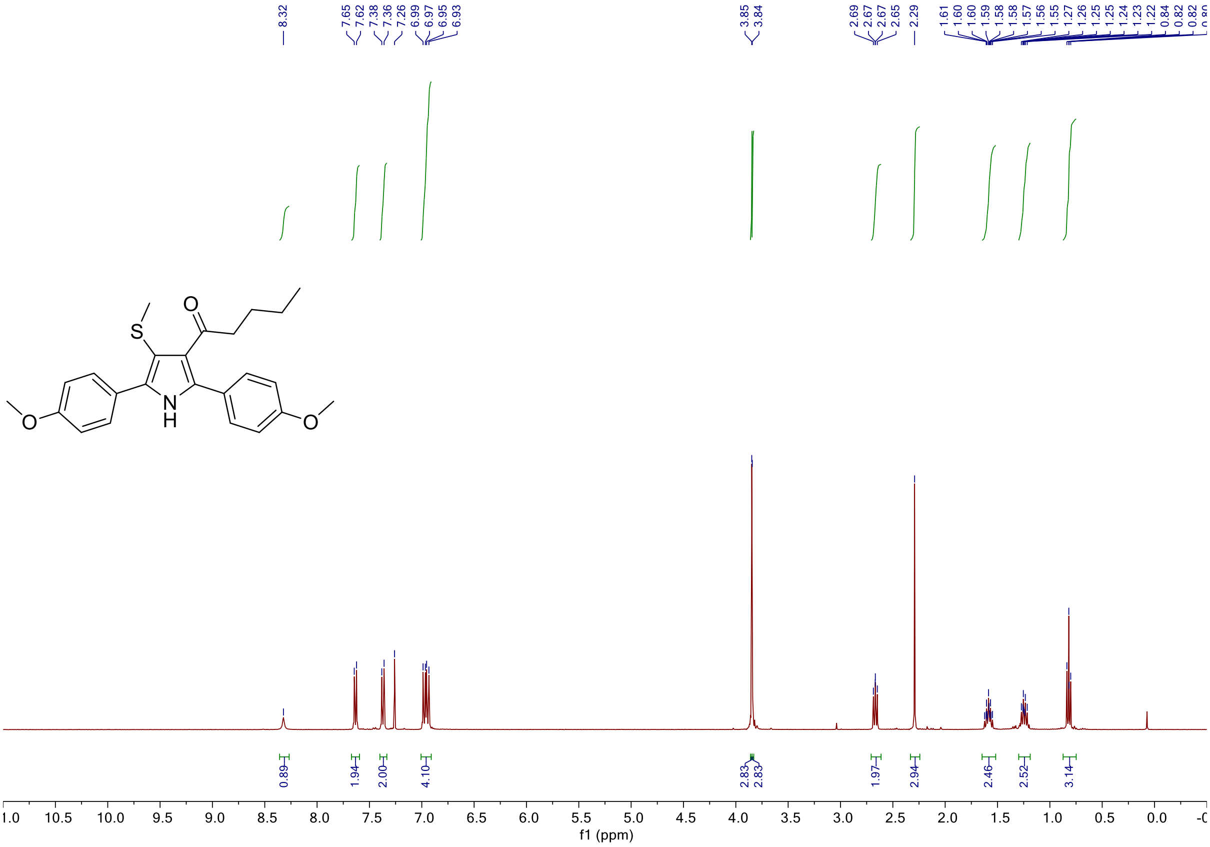

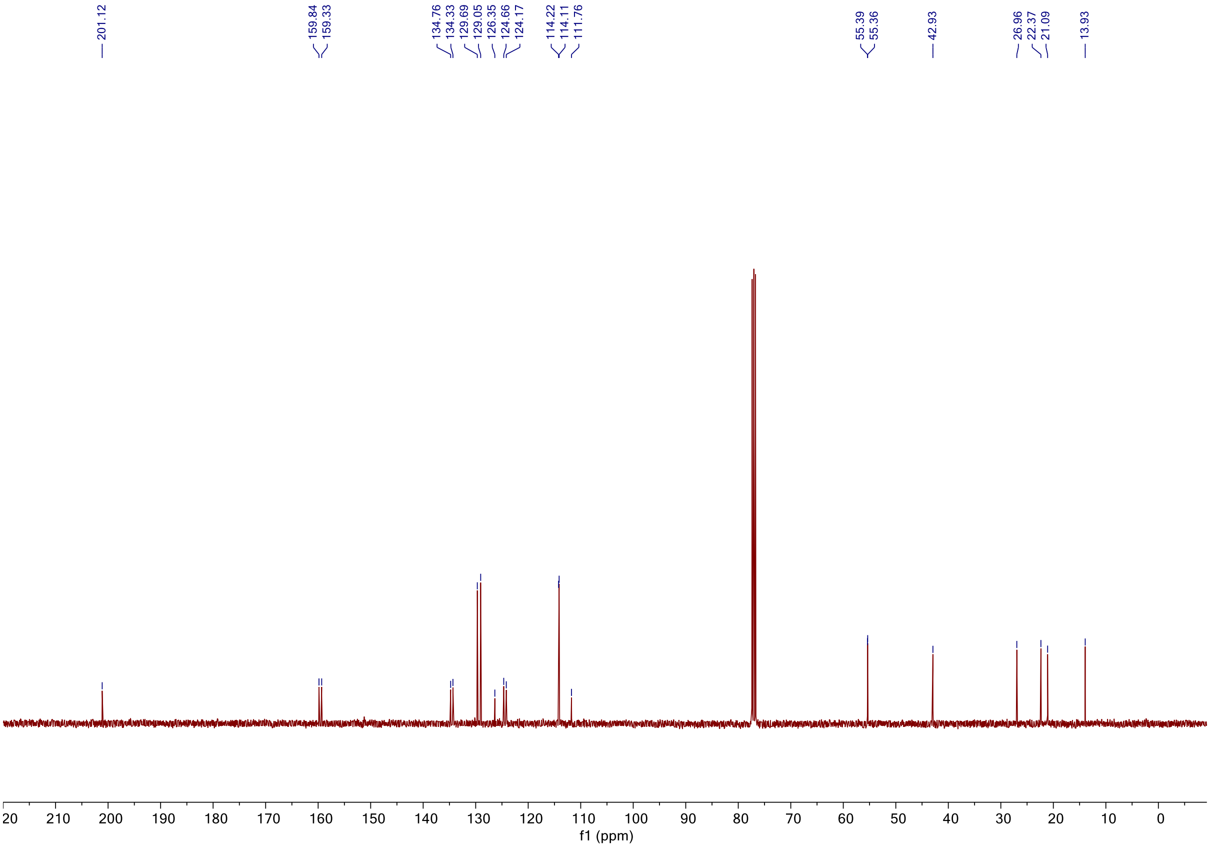

5-(4-Methoxyphenyl)-4-(methylthio)-1*H*-pyrrole-3-carbaldehyde (3x) in $CDCl_3$ ^{1}H-NMR and ^{13}C-NMR

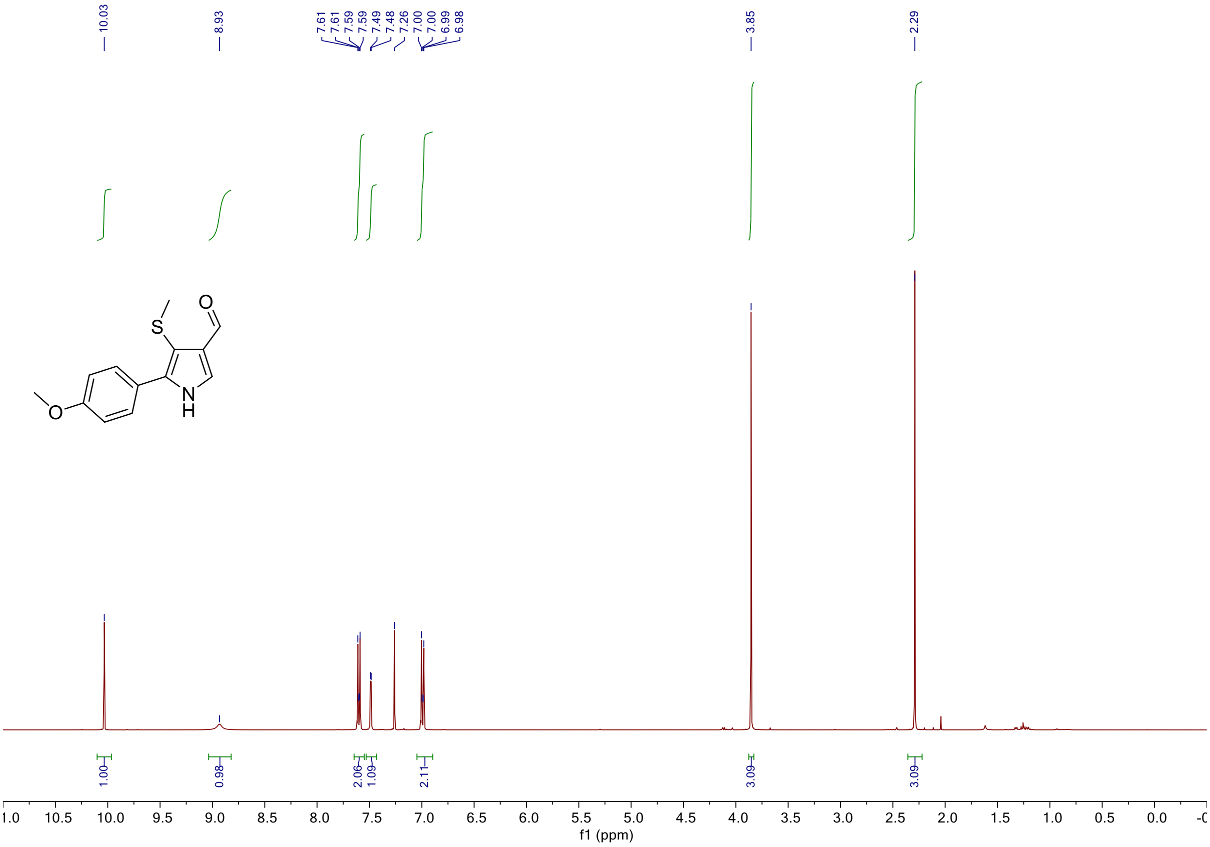

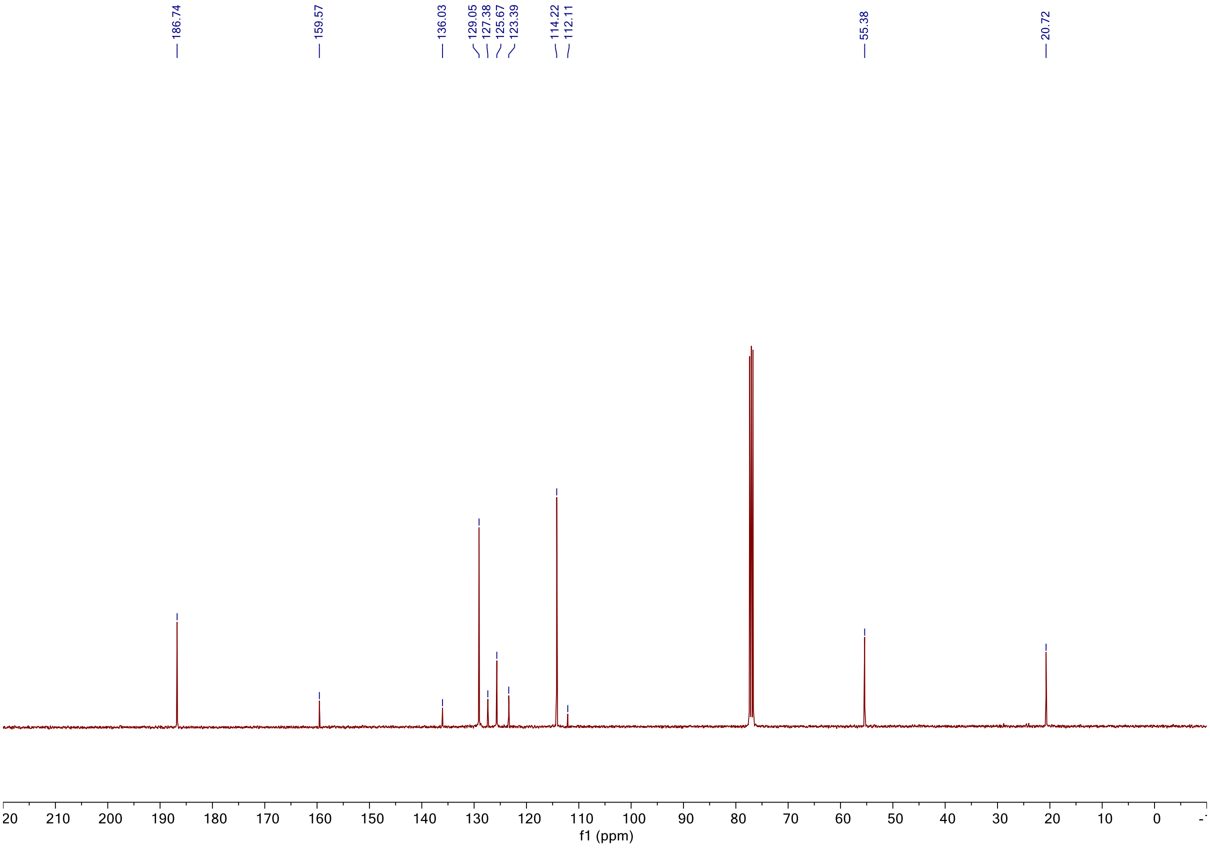

5-(4-Methoxyphenyl)-4-(methylthio)-2-phenyl-1*H*-pyrrole-3-carboxamide (3y) in $CDCl_3$ (^{1}H-NMR) and DMSO-d_6 (^{13}C-NMR)

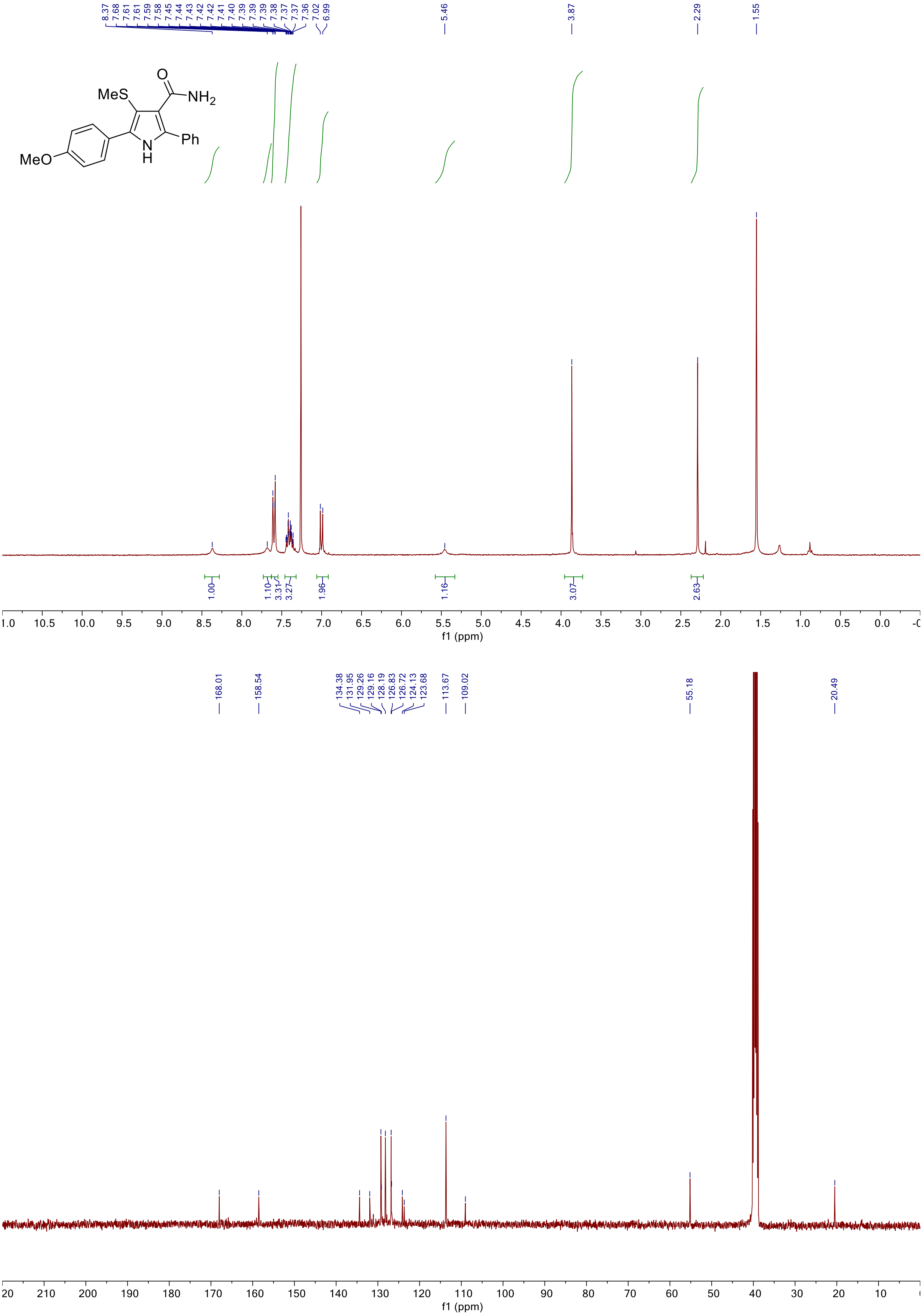

5-(4-Methoxyphenyl)-2-methyl-4-(methylthio)-1*H*-pyrrole-3-carboxamide (3z) in CD_3OD ^1H-NMR and ^{13}C-NMR

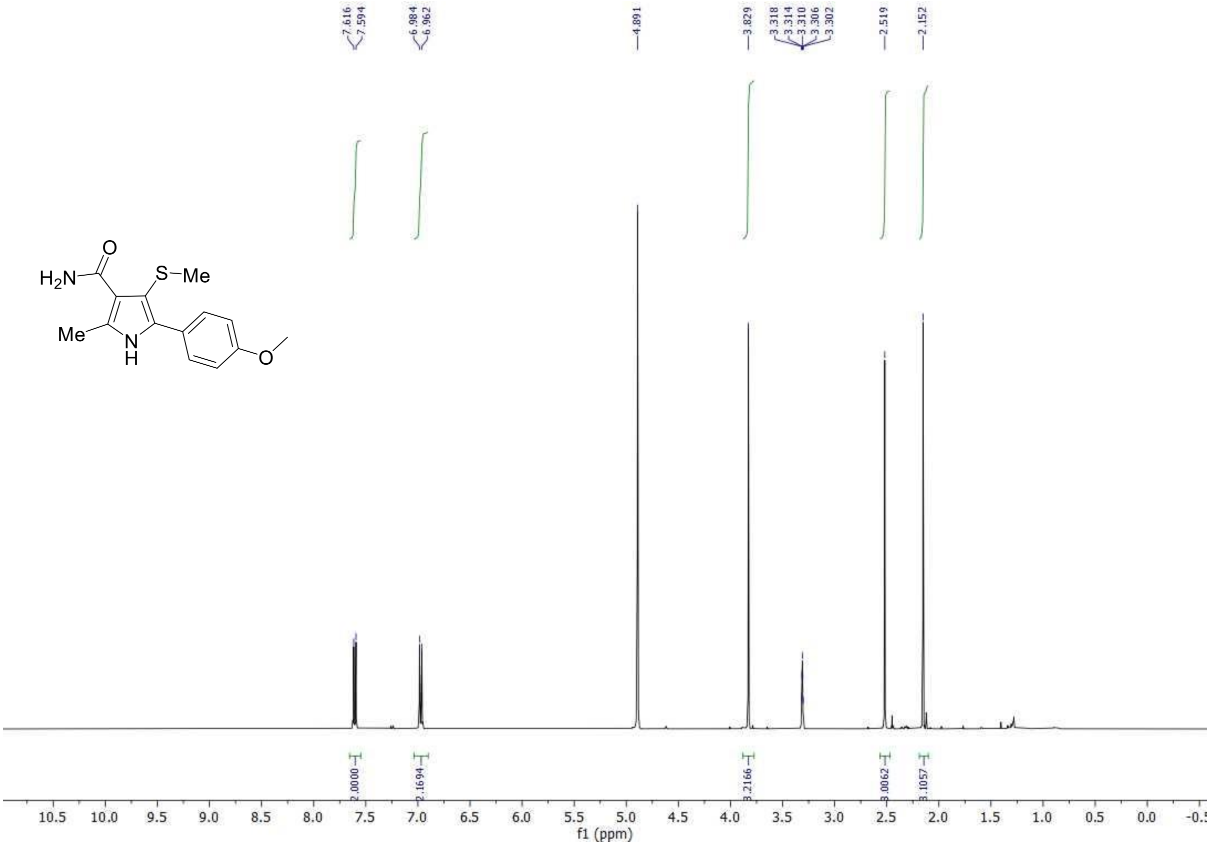

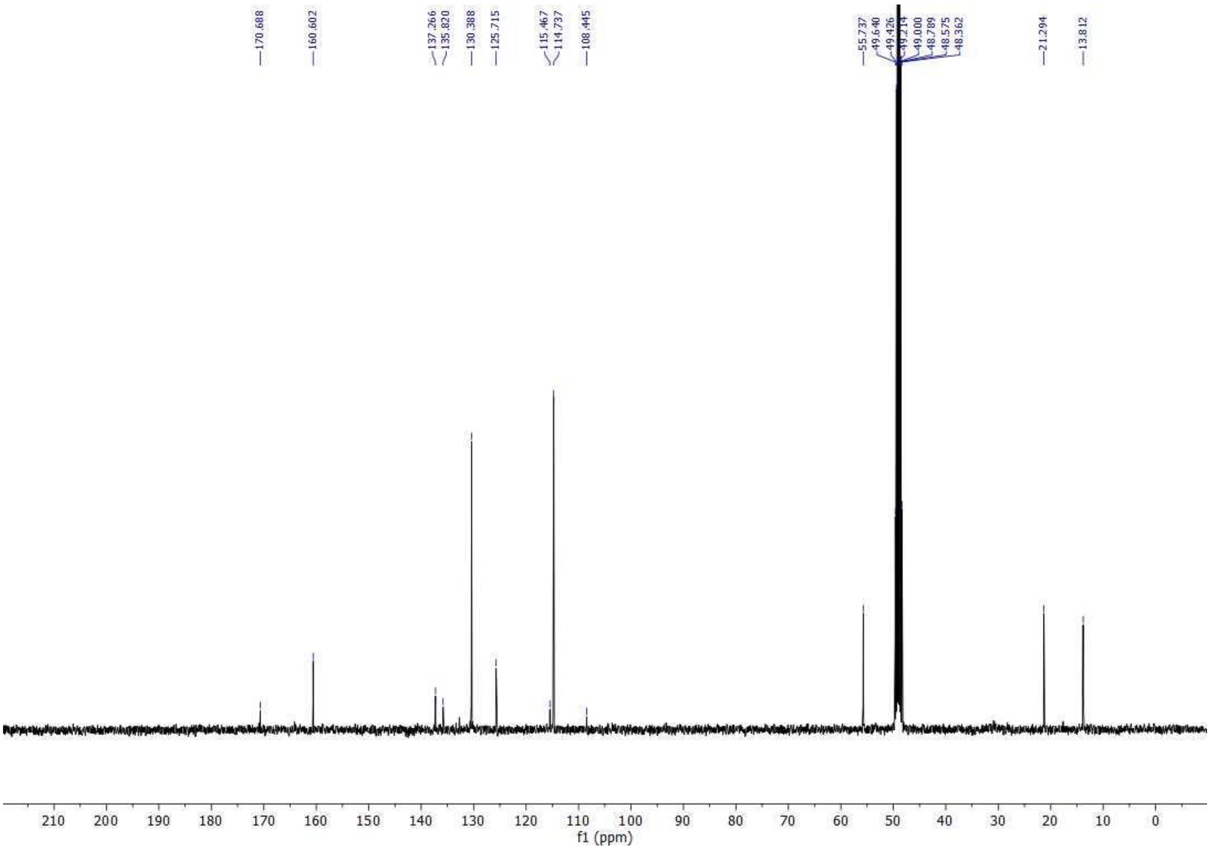

(*E*)-(5-(4-Methoxyphenyl)-4-(methylthio)-2-styryl-1*H*-pyrrol-3-yl)(phenyl)methanone (3aa) in $CDCl_3$ ^1H-NMR and ^{13}C-NMR

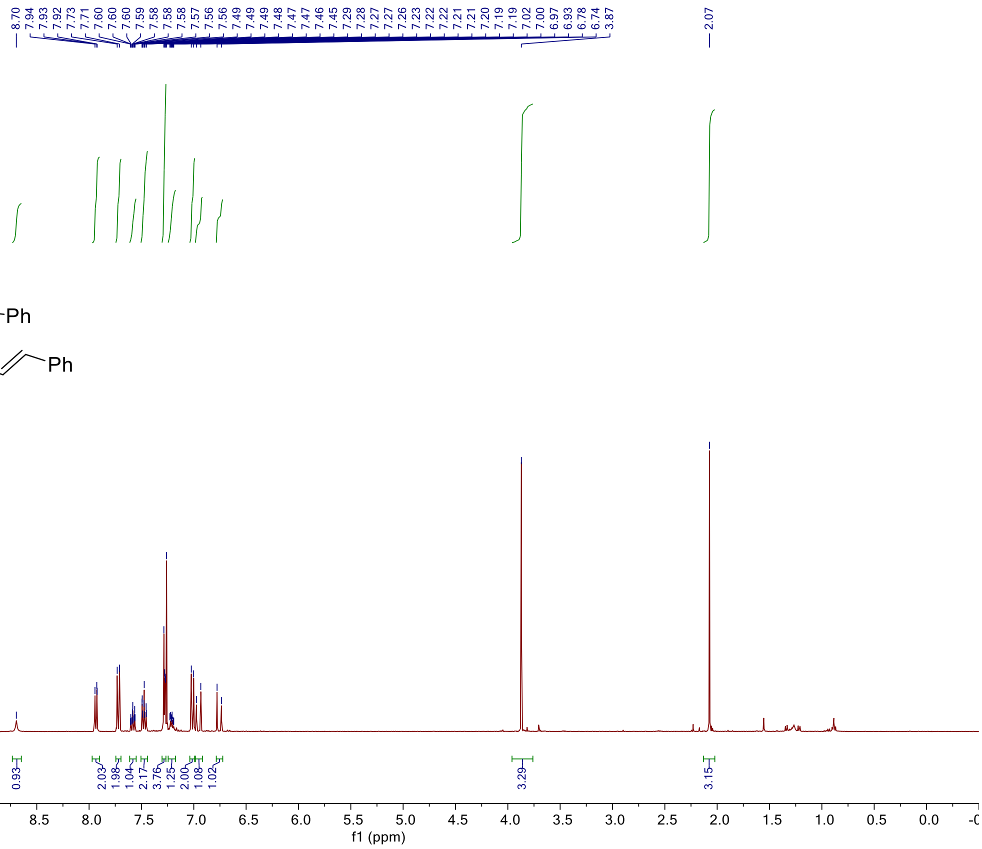

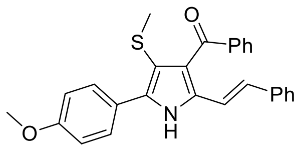

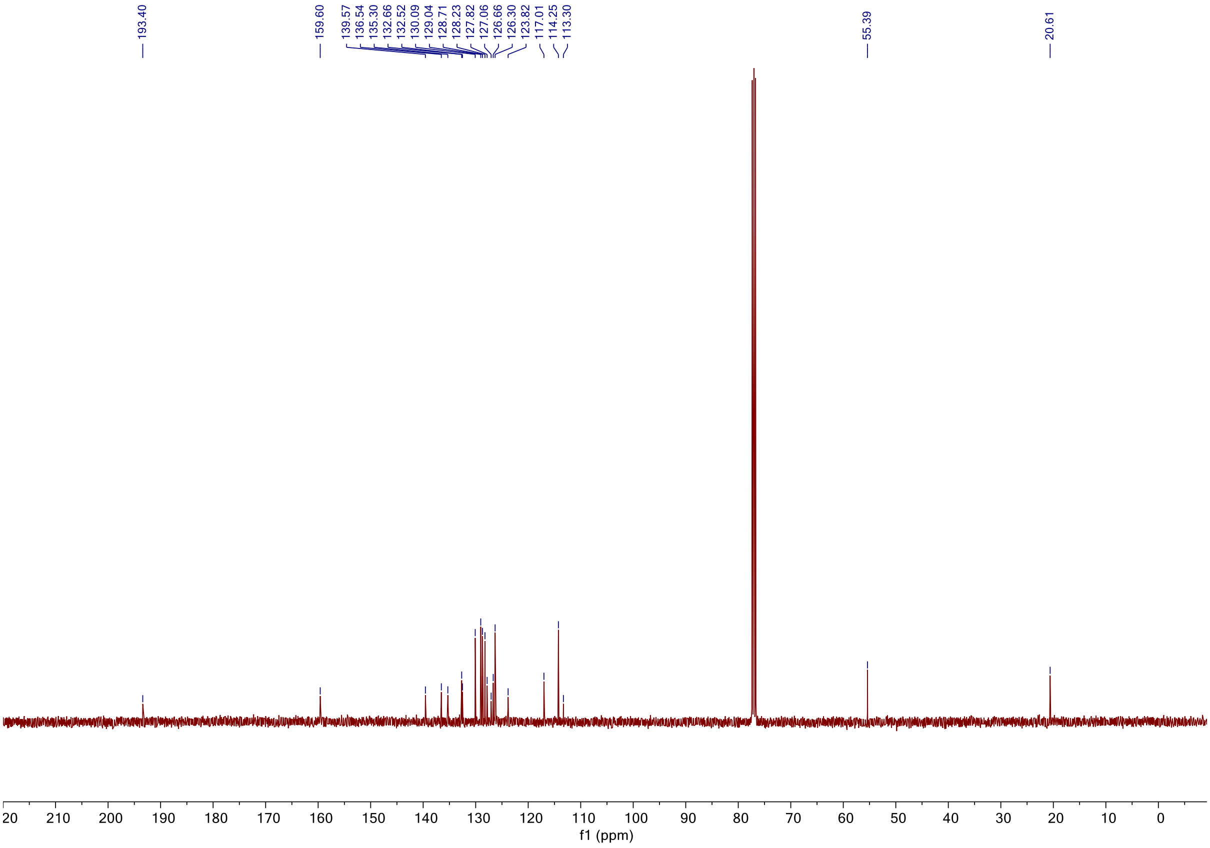

(*E*)-(5-(4-Fluorophenyl)-4-(methylthio)-2-styryl-1*H*-pyrrol-3-yl)(phenyl)methanone (3ab) in $CDCl_3$ ^1H-NMR and ^{13}C-NMR

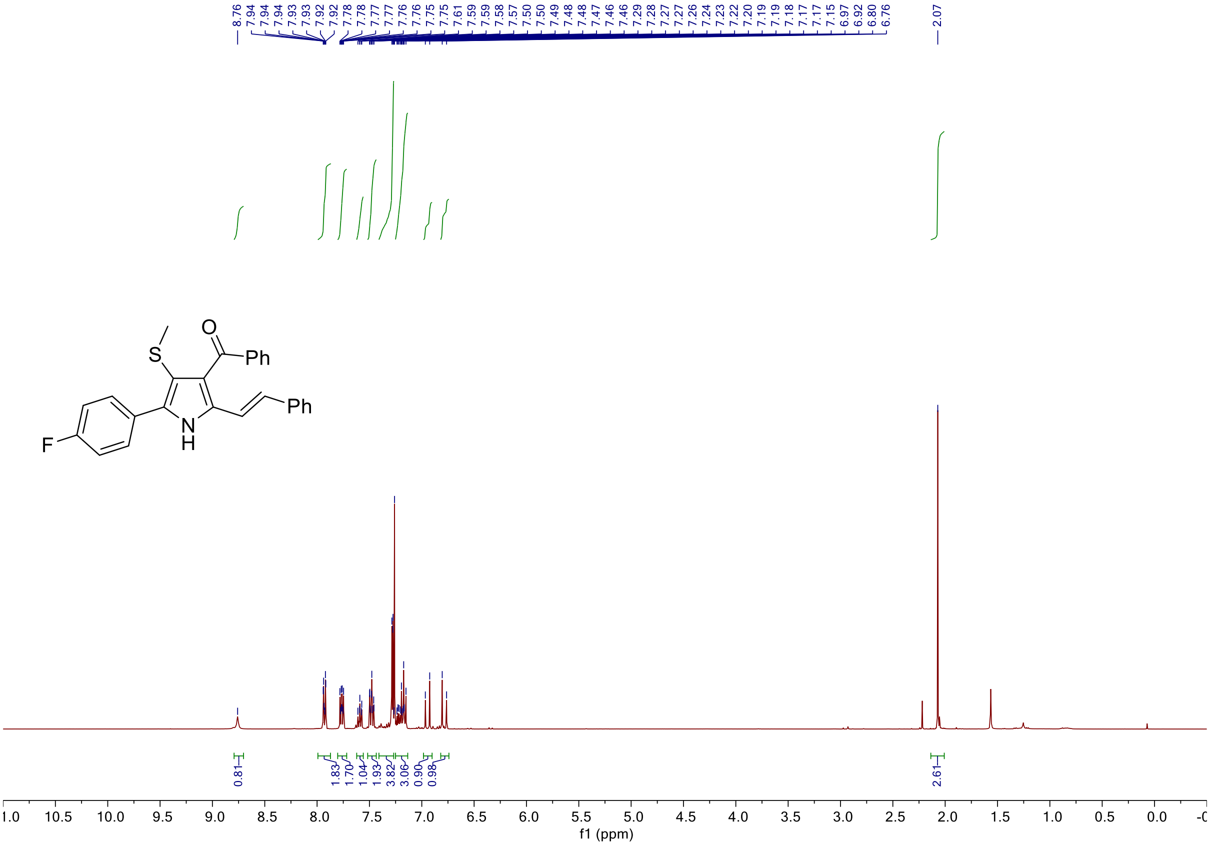

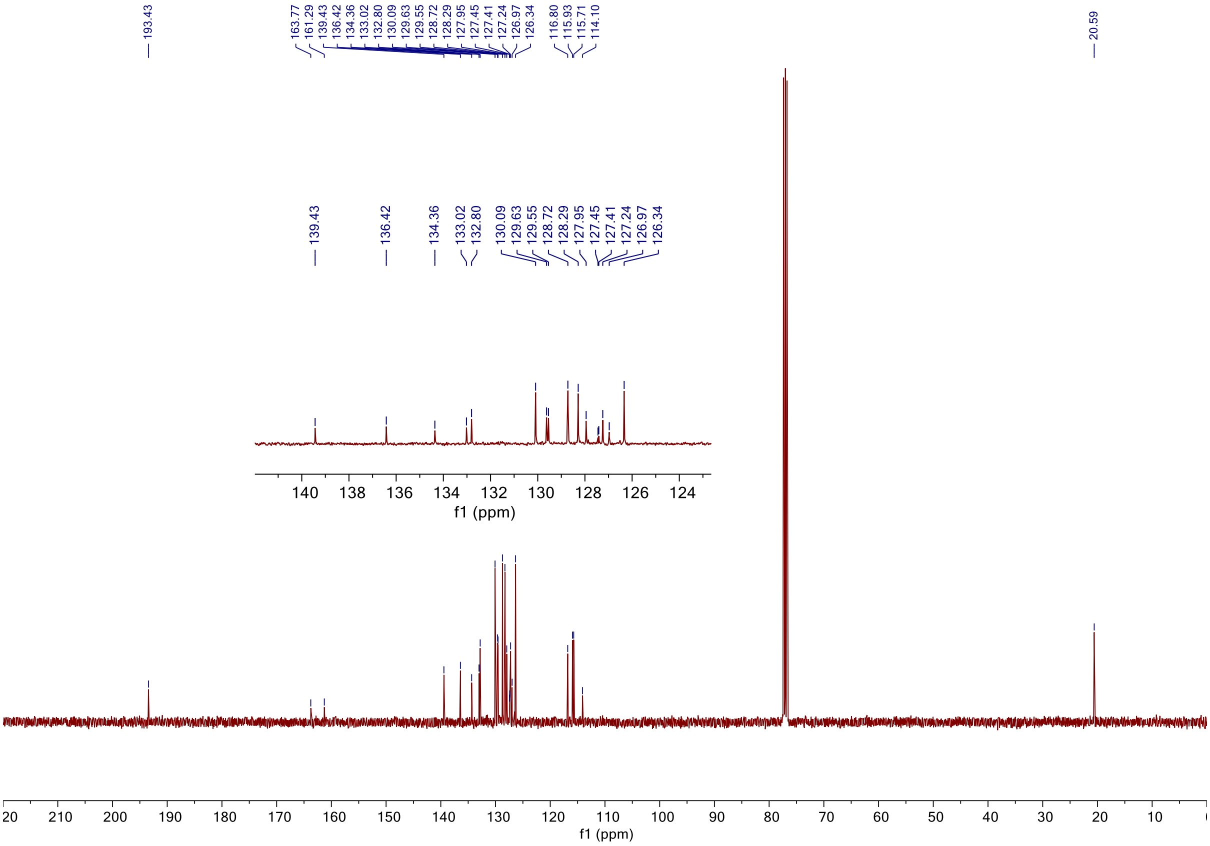

1-(2,5-Bis(4-methoxyphenyl)-4-(methylthio)-1*H*-pyrrol-3-yl)-2-hydroxyethan-1-one (3ac) in $CDCl_3$ (^1H-NMR) and acetone-d_6 (^{13}C-NMR)

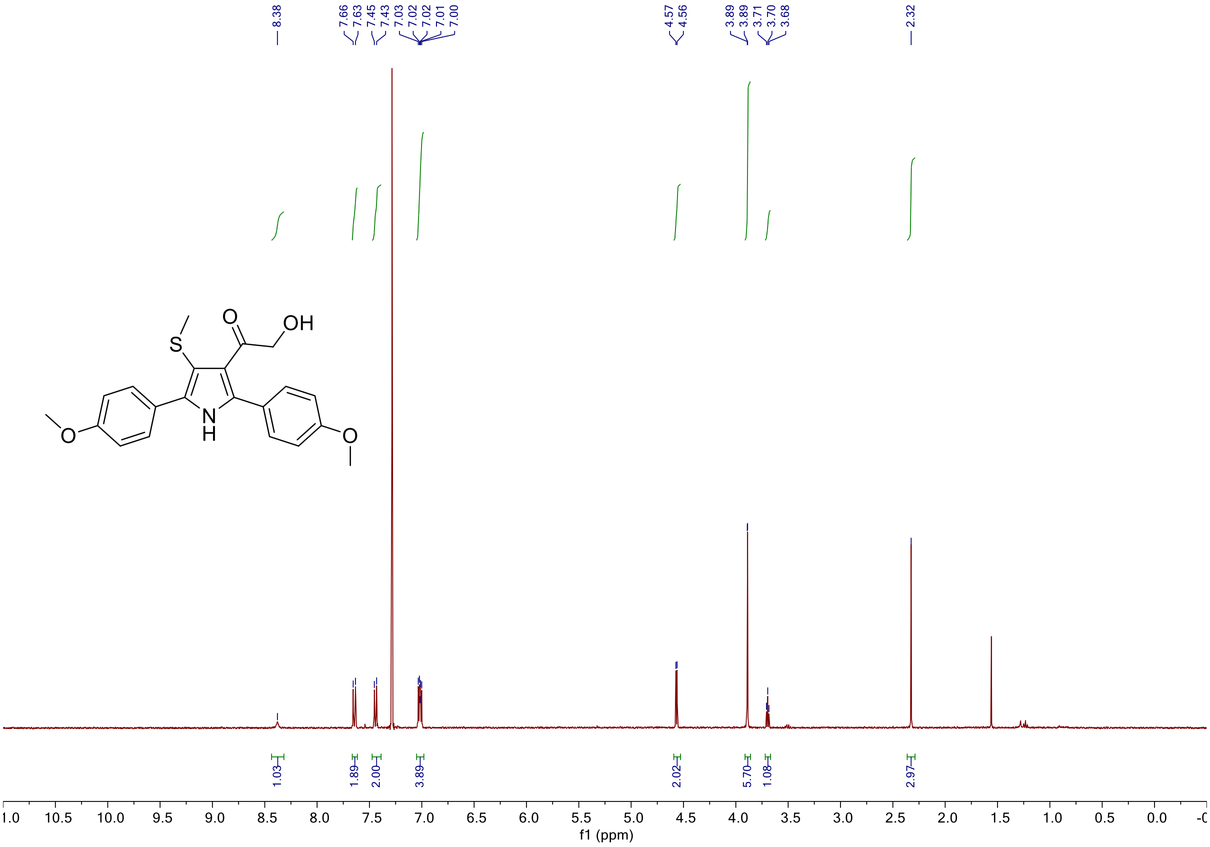

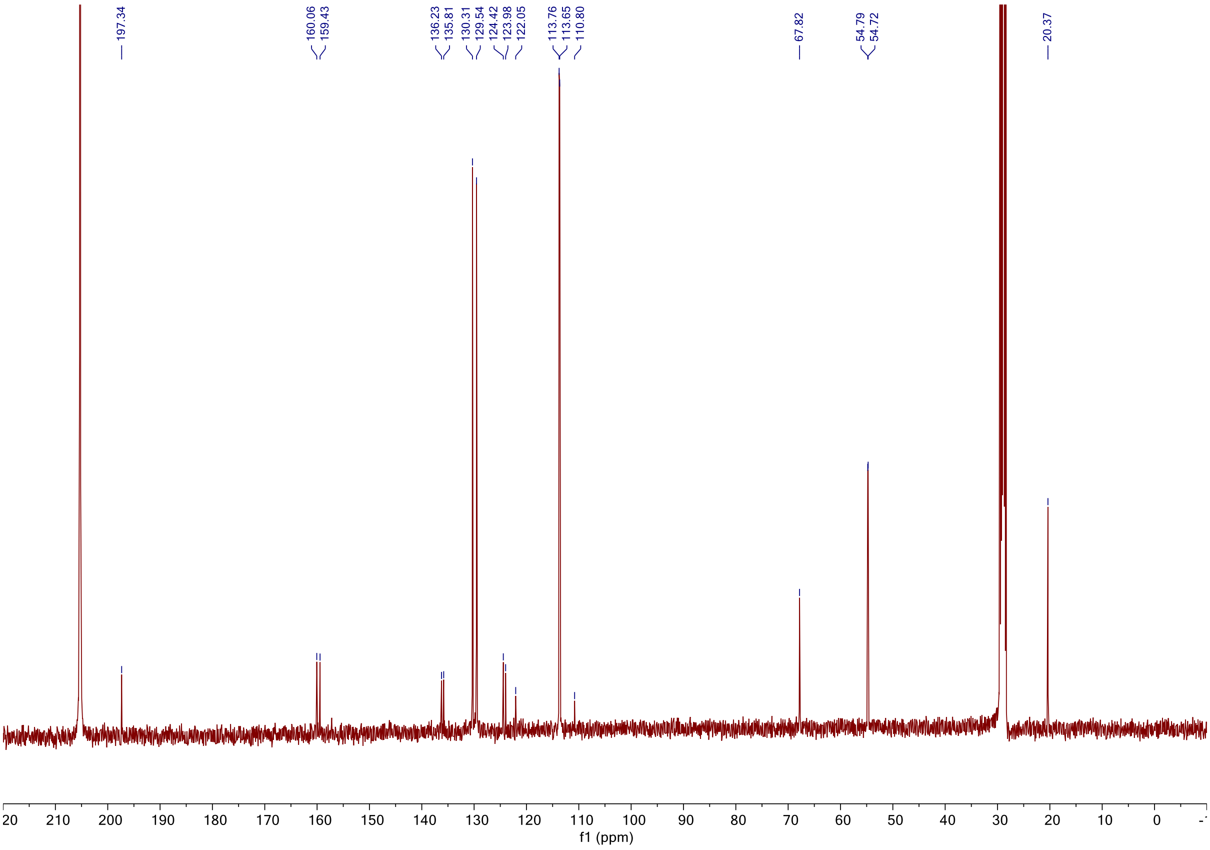

2-(2,5-Bis(4-methoxyphenyl)-4-(methylthio)-1*H*-pyrrol-3-yl)-2-oxoethyl acetate (3ad) in $CDCl_3$

^{1}H-NMR and ^{13}C-NMR

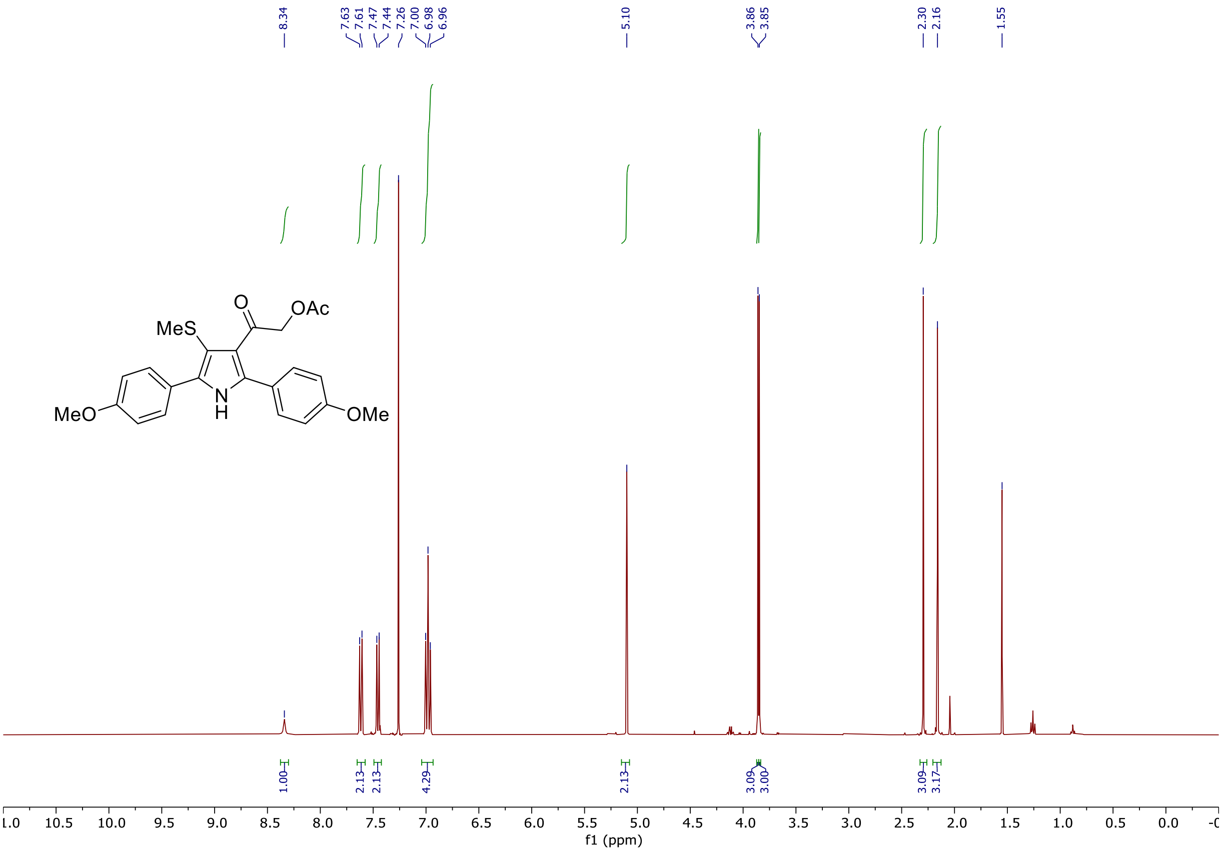

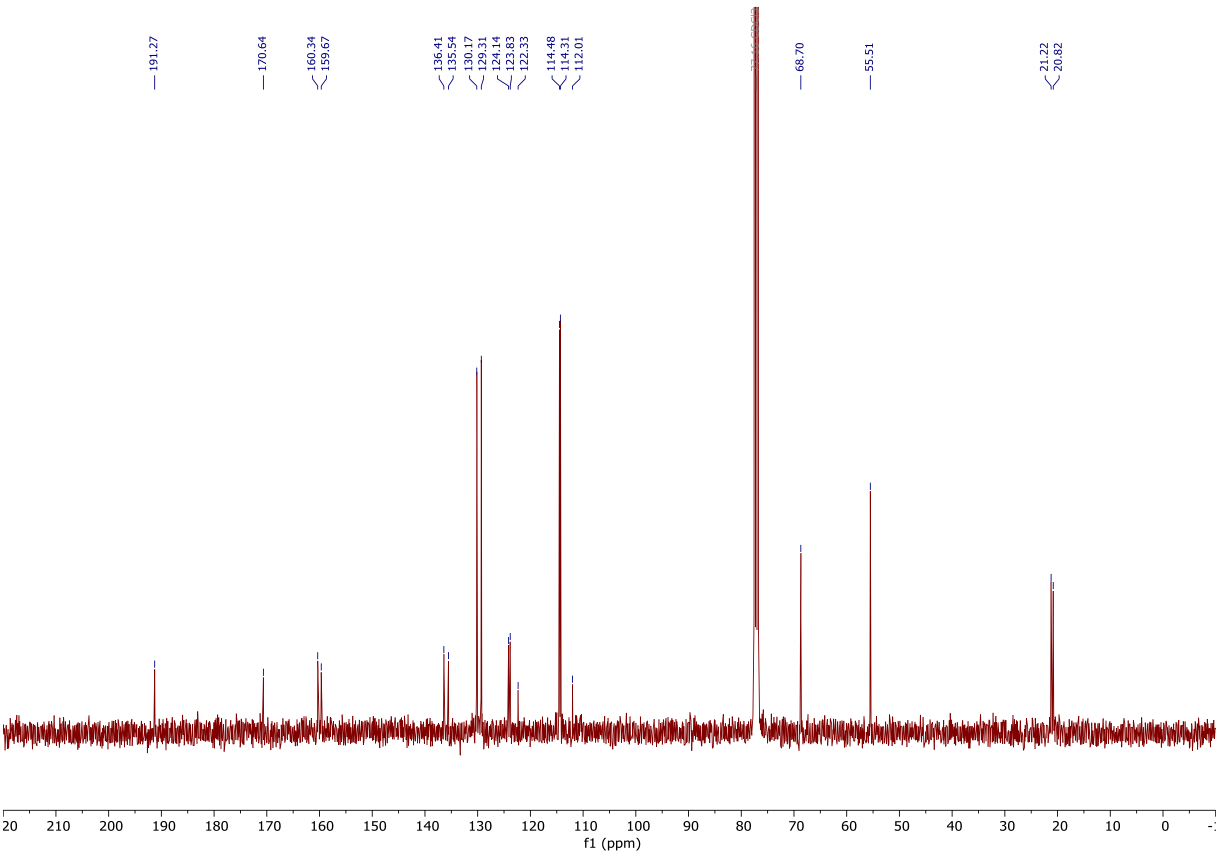

(2-Cyclohexyl-5-(4-methoxyphenyl)-4-(methylthio)-1*H*-pyrrol-3-yl)(phenyl)methanone (3ae) in $CDCl_3$ ^{1}H-NMR and ^{13}C-NMR

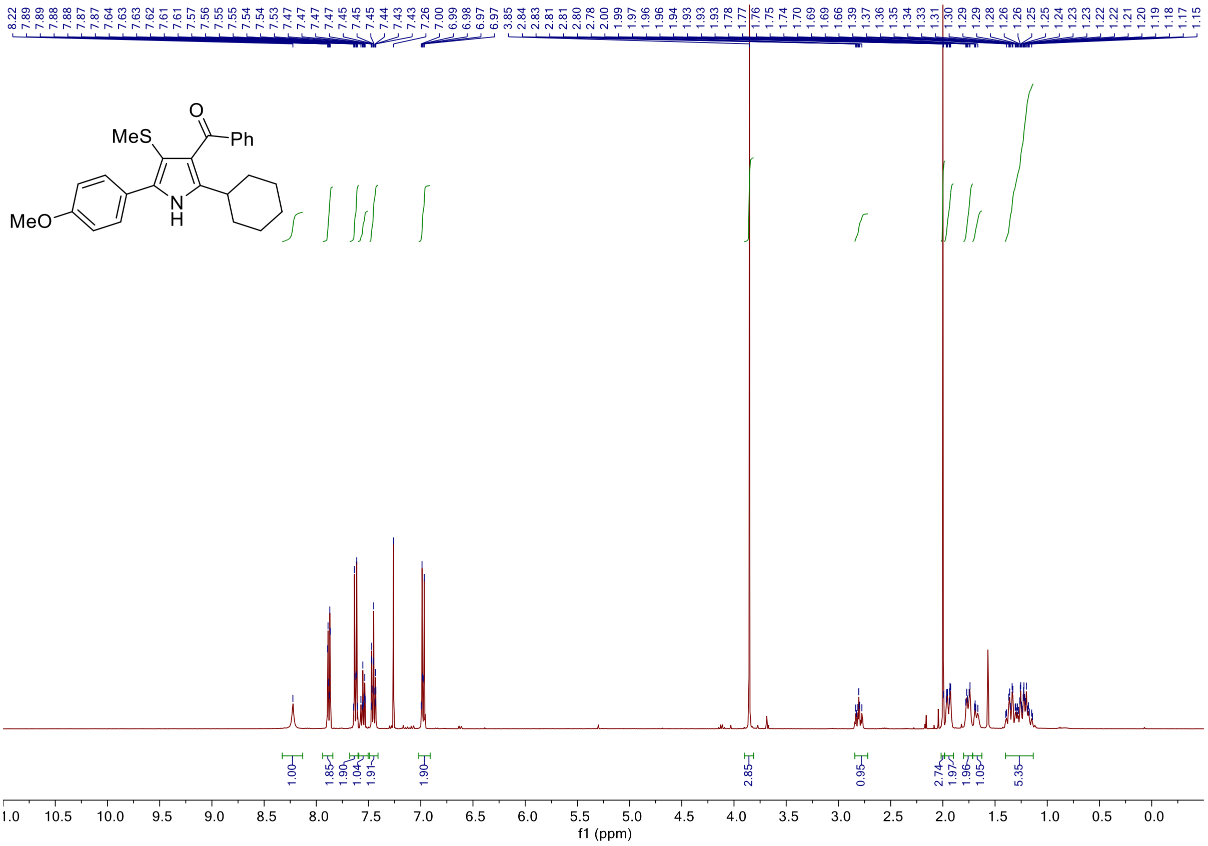

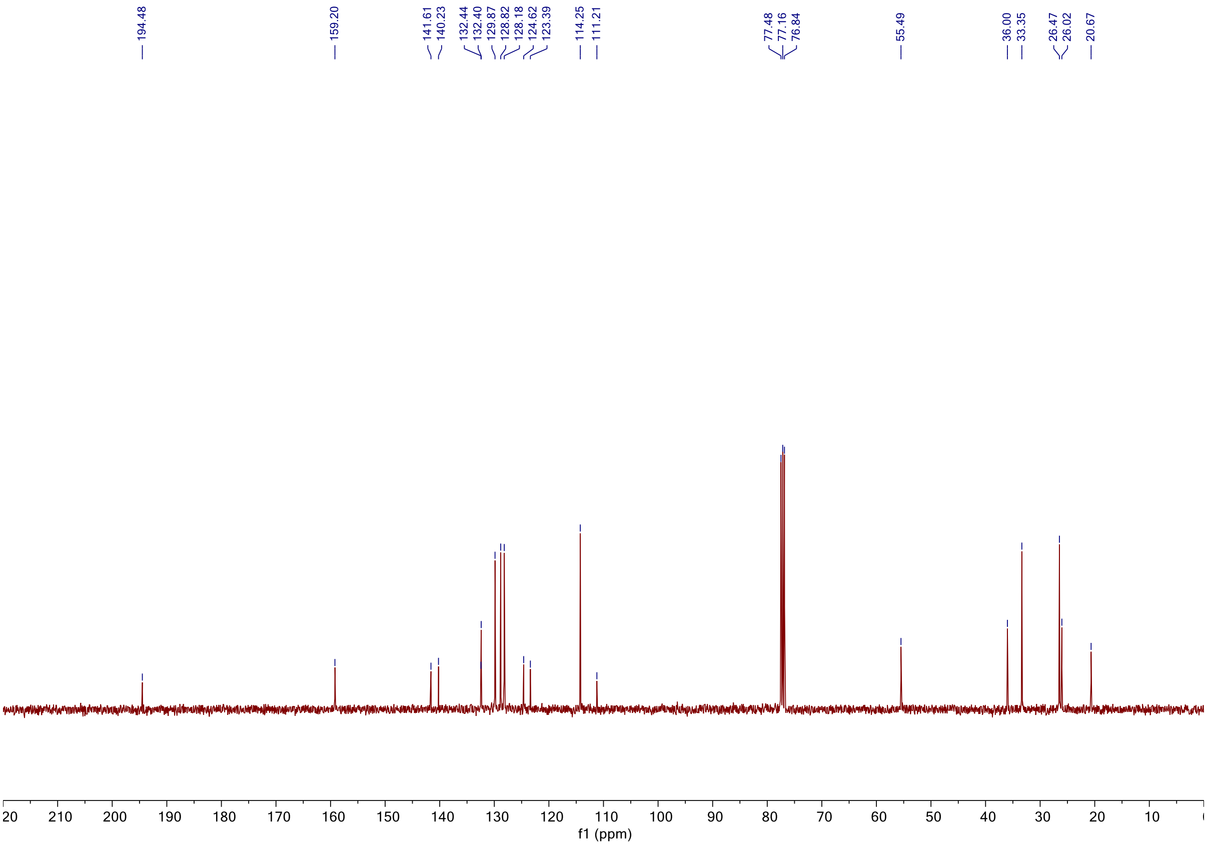

(2-(2-Bromophenyl)-5-(4-methoxyphenyl)-4-(methylthio)-1*H*-pyrrol-3-yl)(phenyl)methanone (3af) in $CDCl_3$ ^{1}H-NMR and ^{13}C-NMR

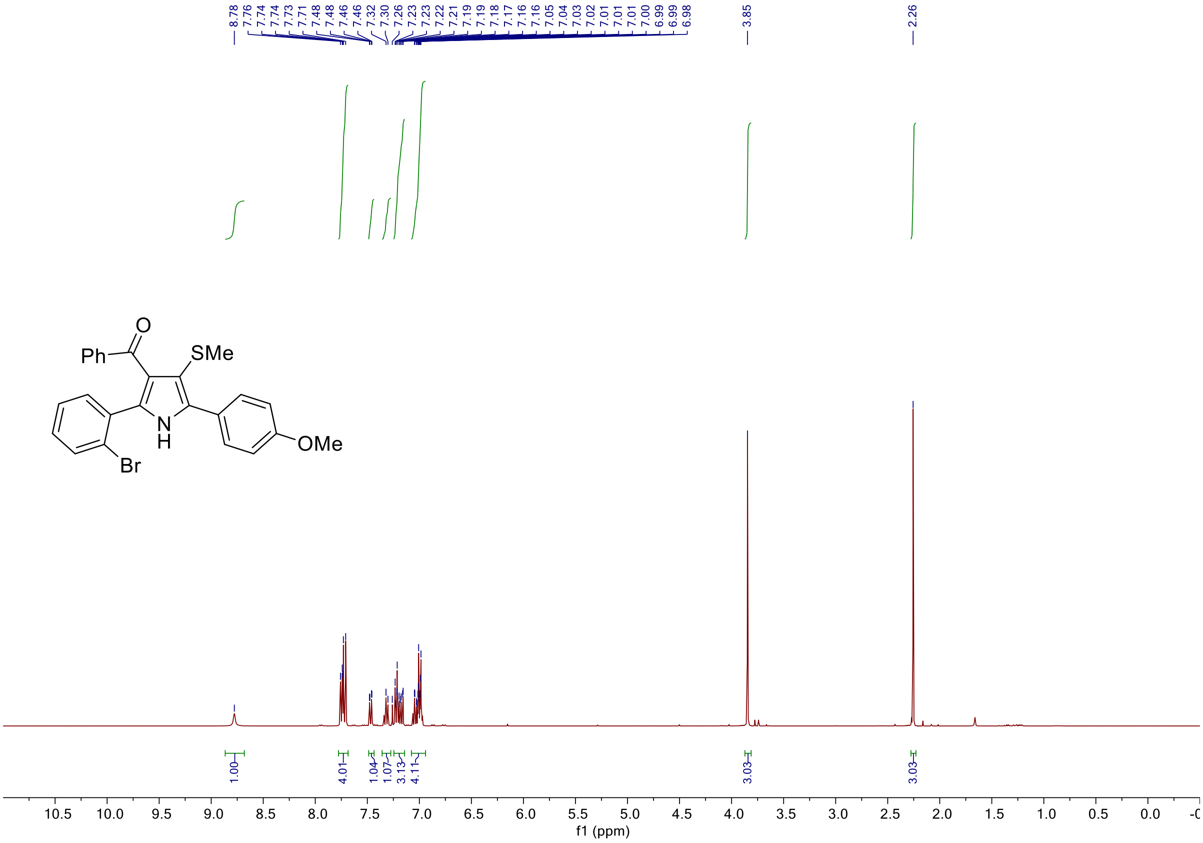

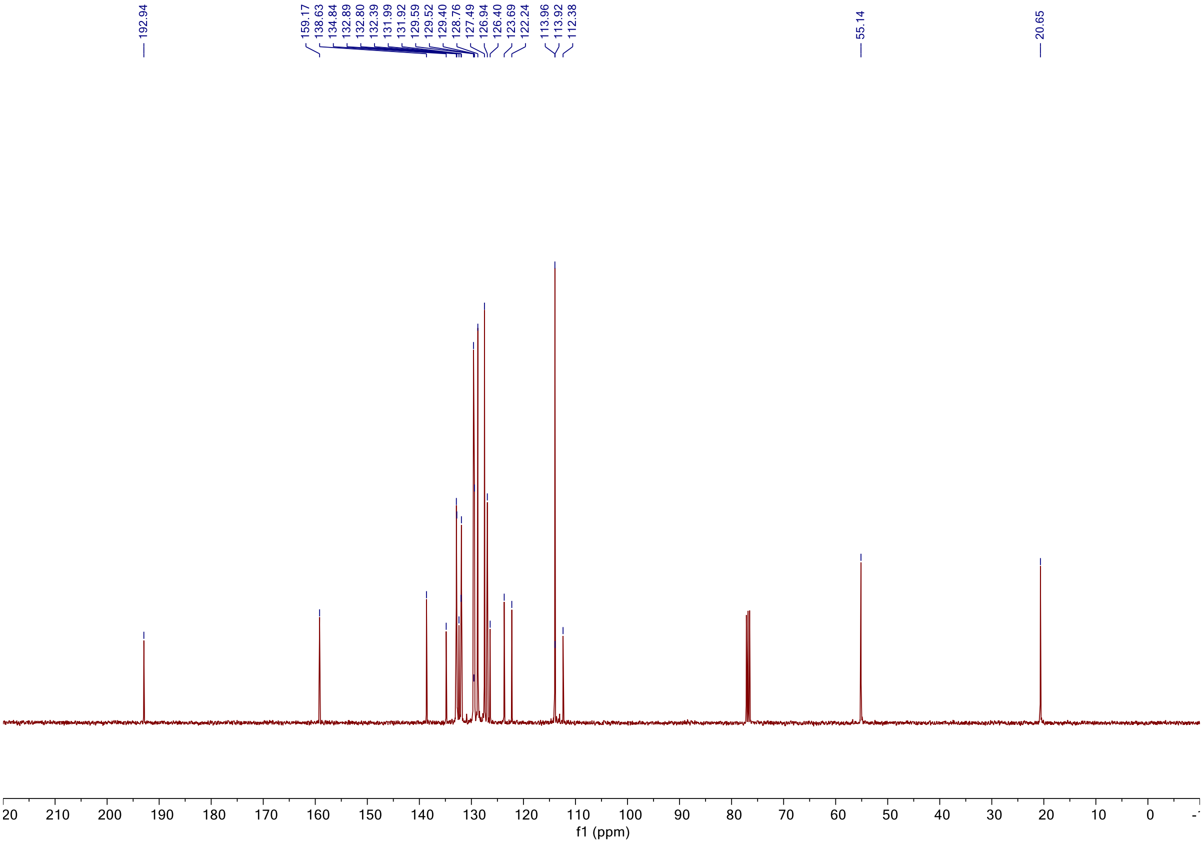

2-(4-Methoxyphenyl)-3-(methylthio)-1*H*-indole-7-carbaldehyde (5a) in $CDCl_3$ ^{1}H-NMR and ^{13}C-NMR

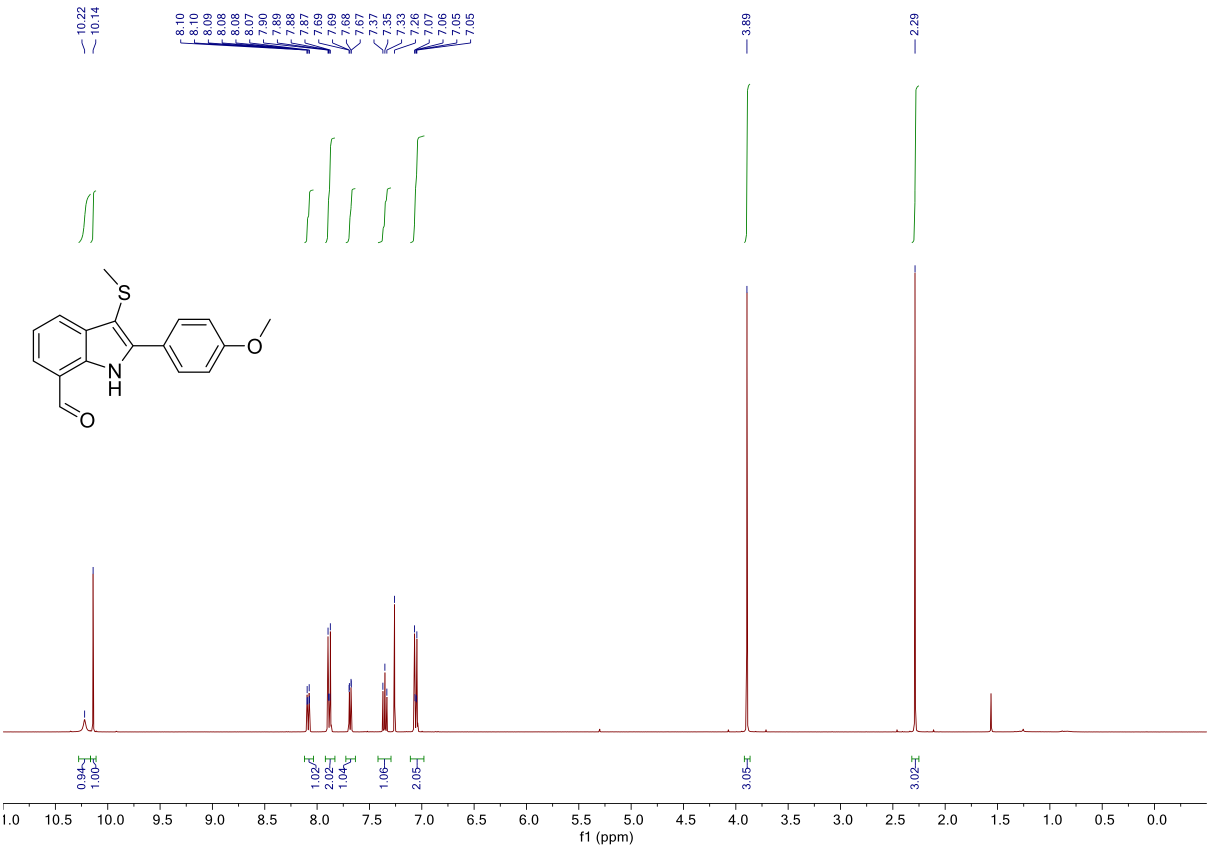

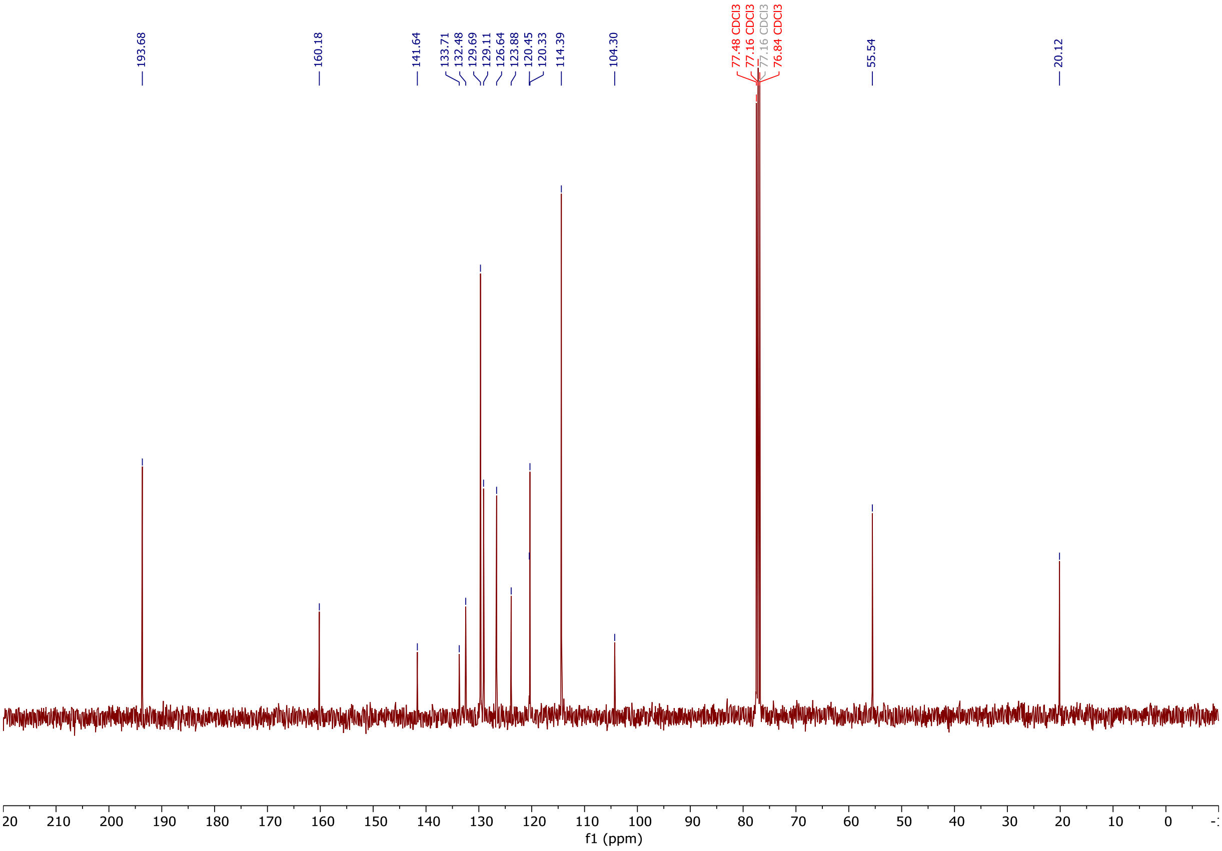

3-(But-3-en-1-ylthio)-2-(4-methoxyphenyl)-1*H*-indole-7-carbaldehyde (5b) in $CDCl_3$ ^{1}H-NMR and ^{13}C-NMR

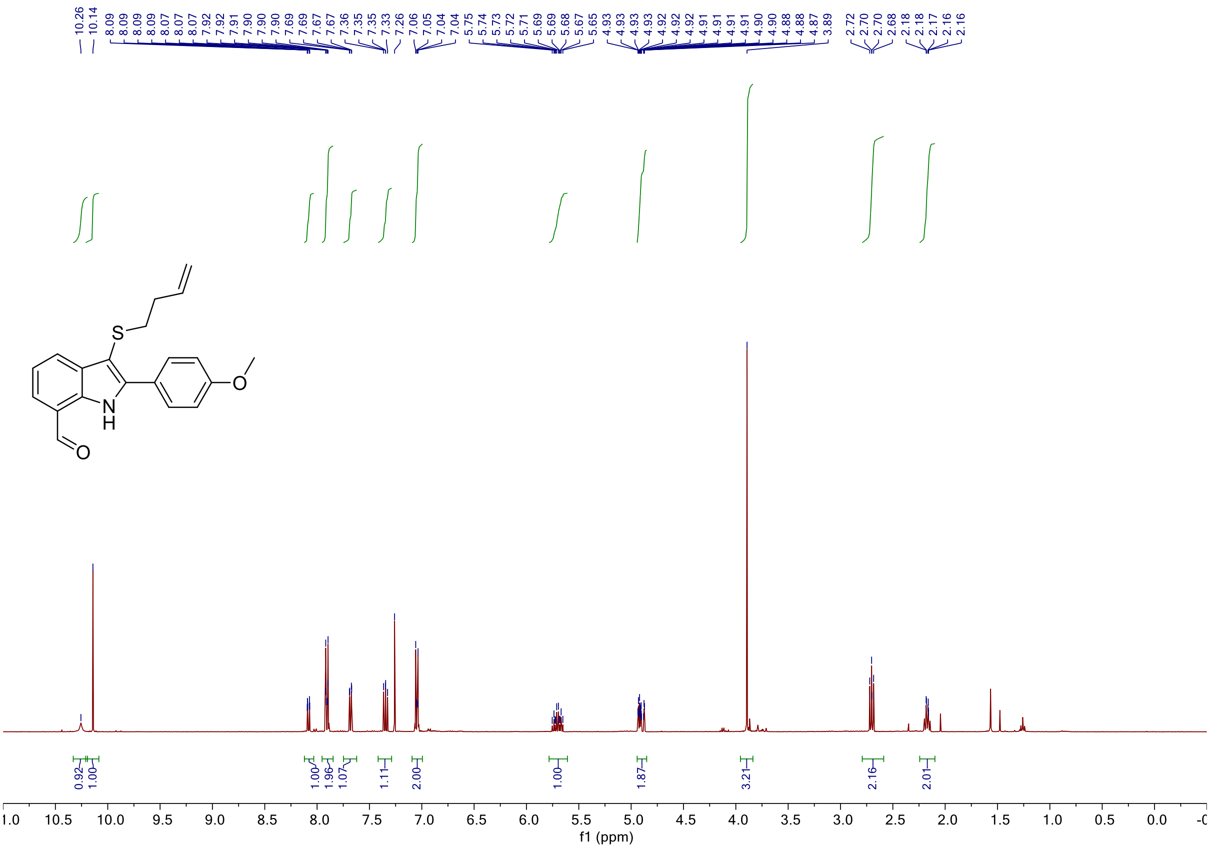

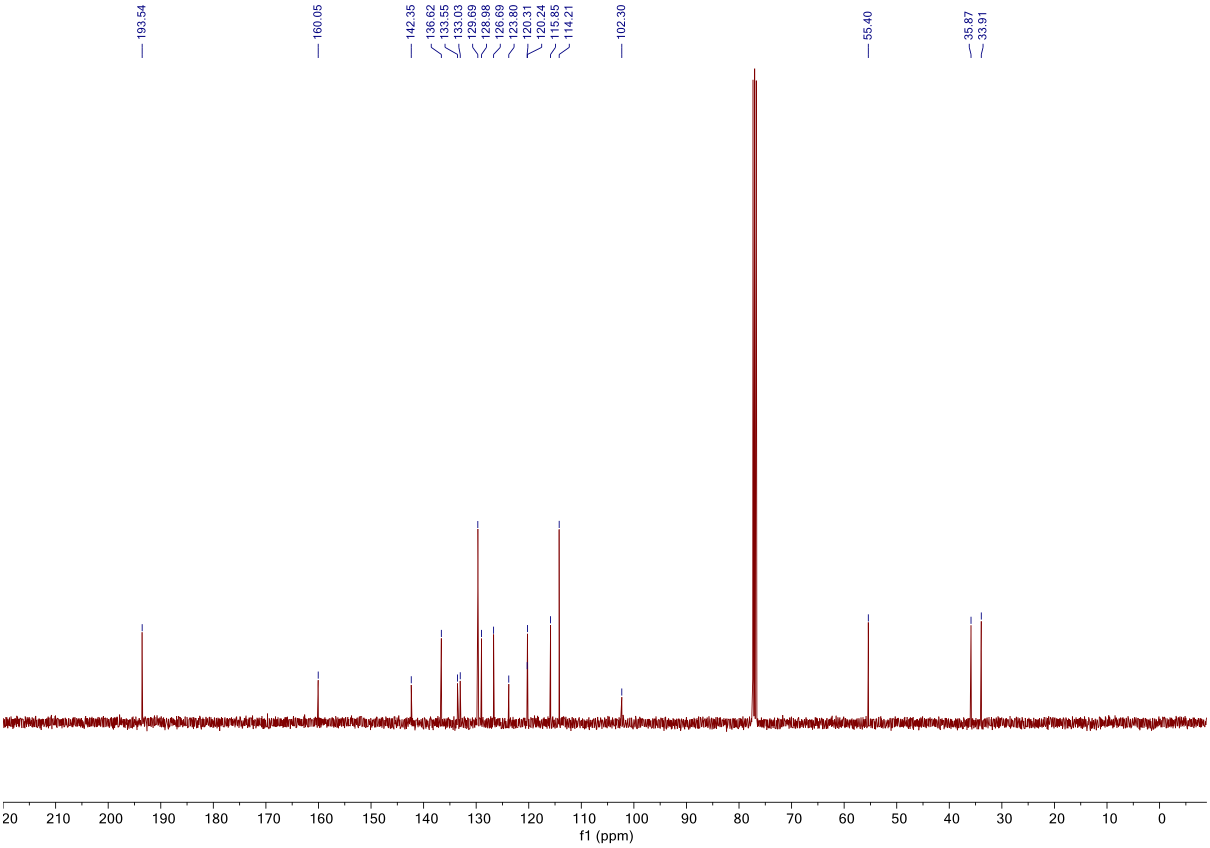

3-(Methylthio)-2-(naphthalen-2-yl)-1*H*-indole-7-carbaldehyde (5c) in $CDCl_3$ ^{1}H-NMR and ^{13}C-NMR

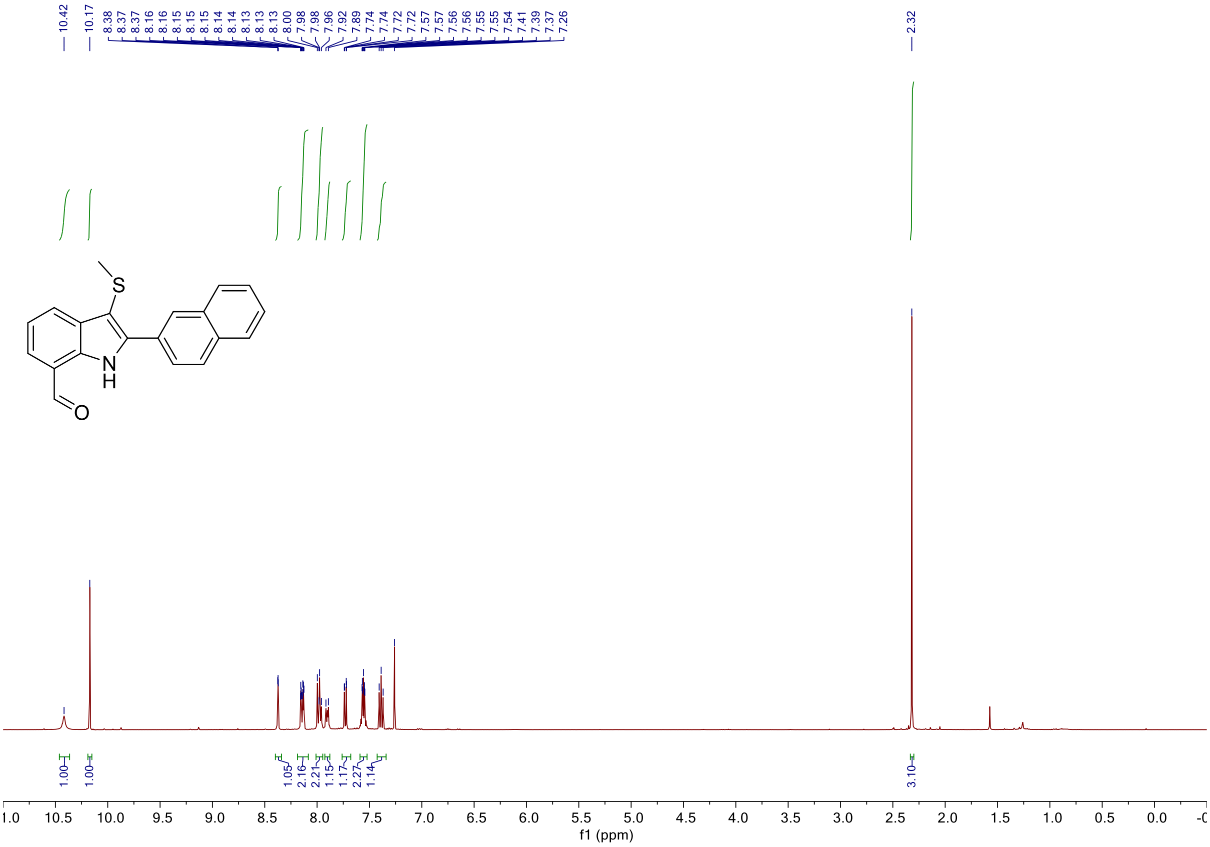

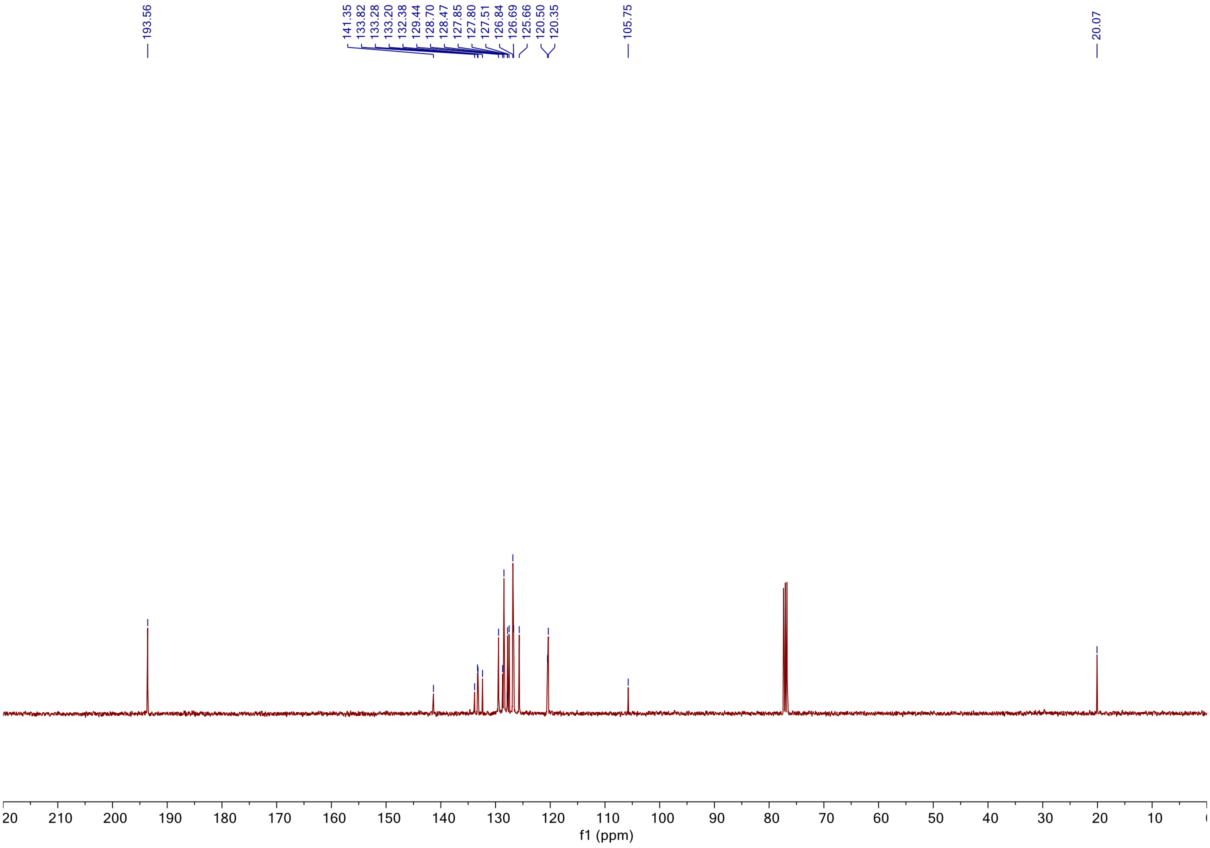

2-(4-Fluorophenyl)-3-(methylthio)-1*H*-indole-7-carbaldehyde (5d) in $CDCl_3$ ^1H-NMR and ^{13}C-NMR

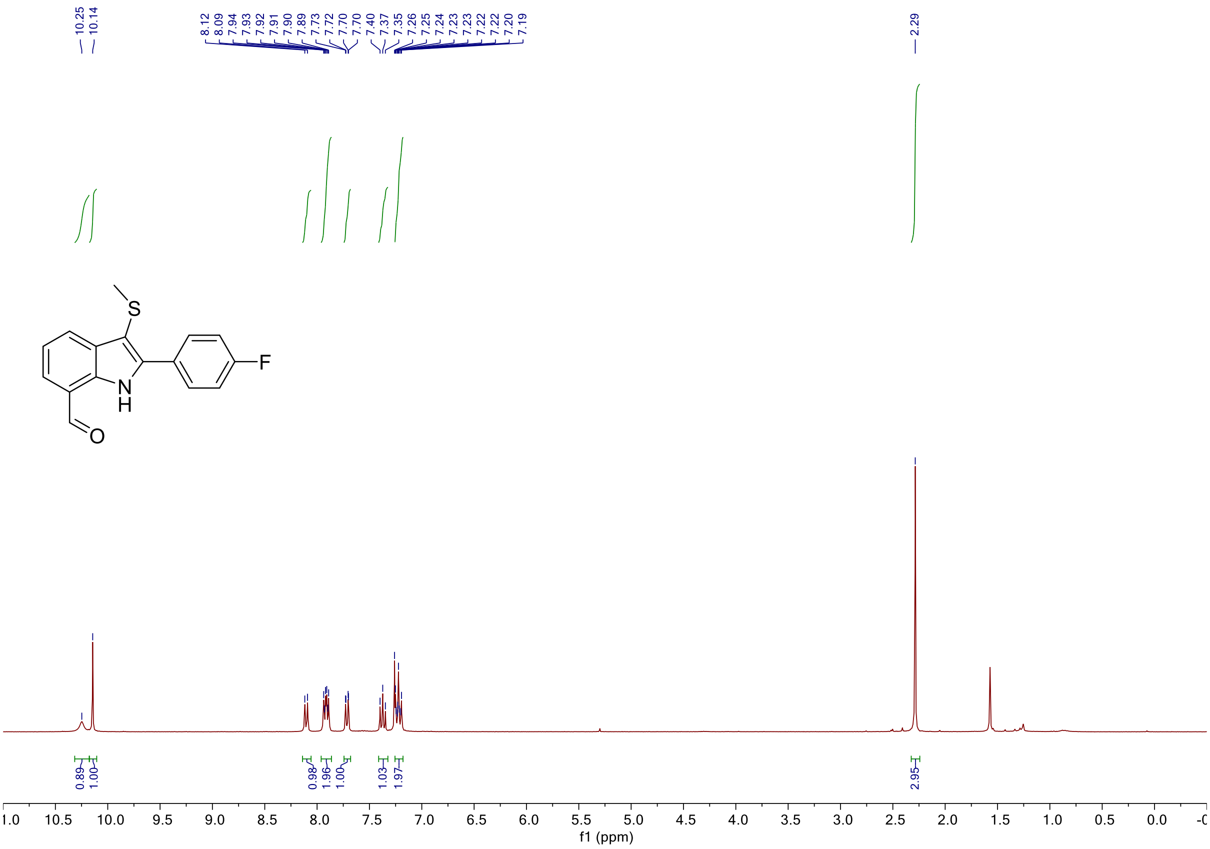

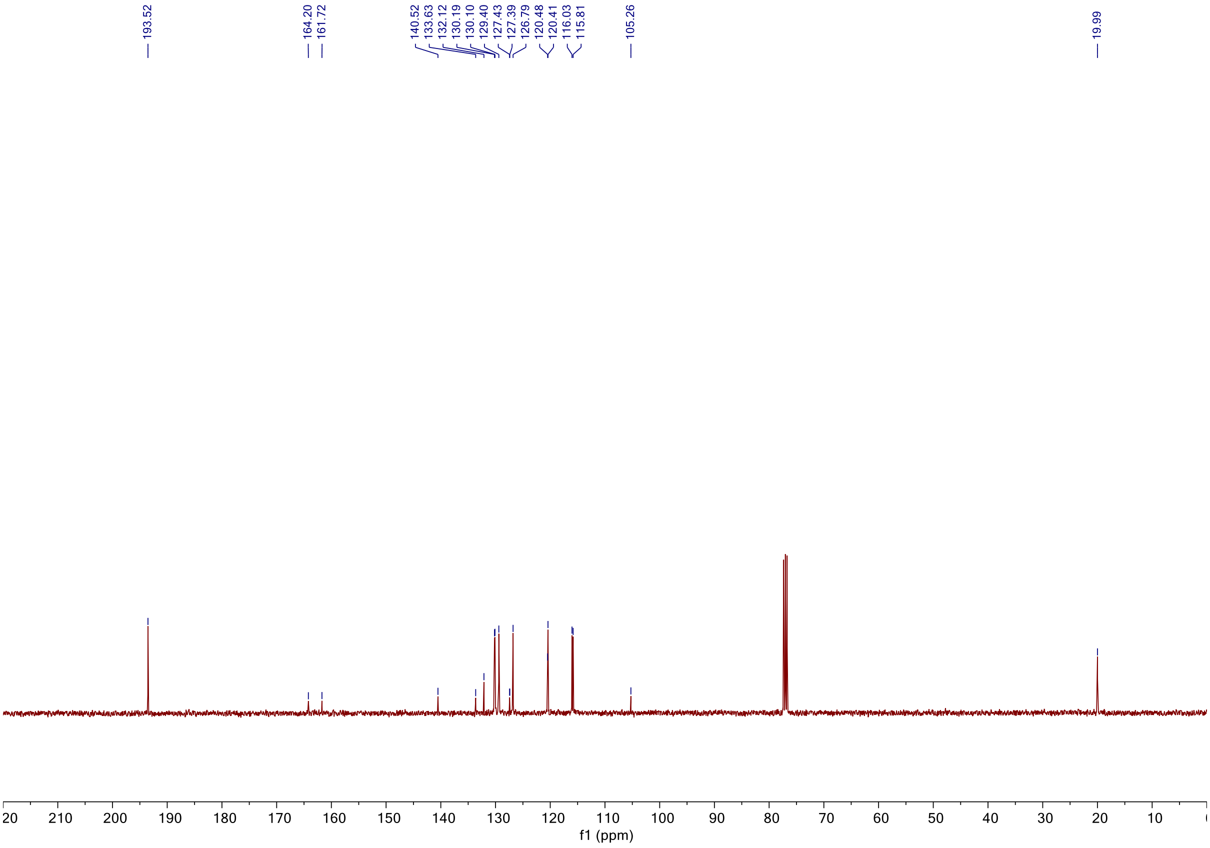

3-(Methylthio)-2-(thiophen-2-yl)-1*H*-indole-7-carbaldehyde (5e) in $CDCl_3$ ^{1}H-NMR and ^{13}C-NMR

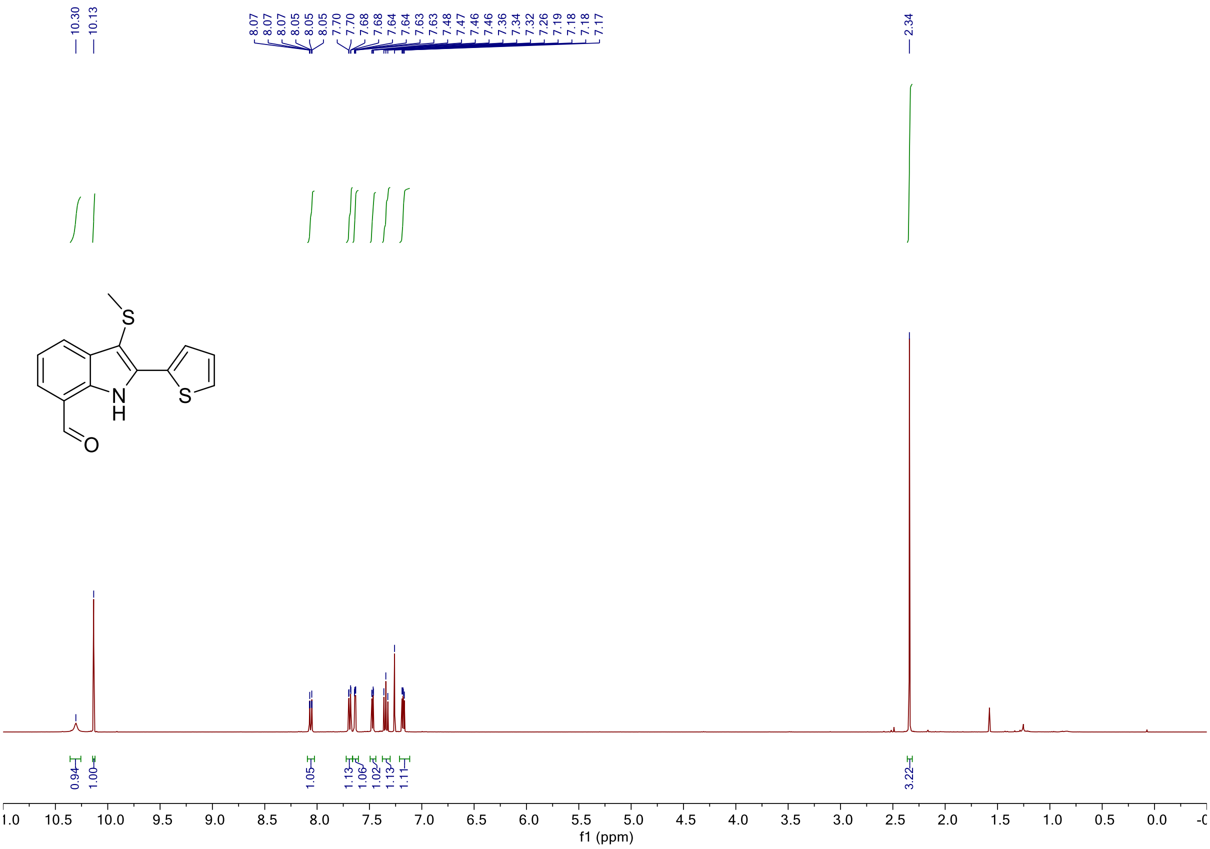

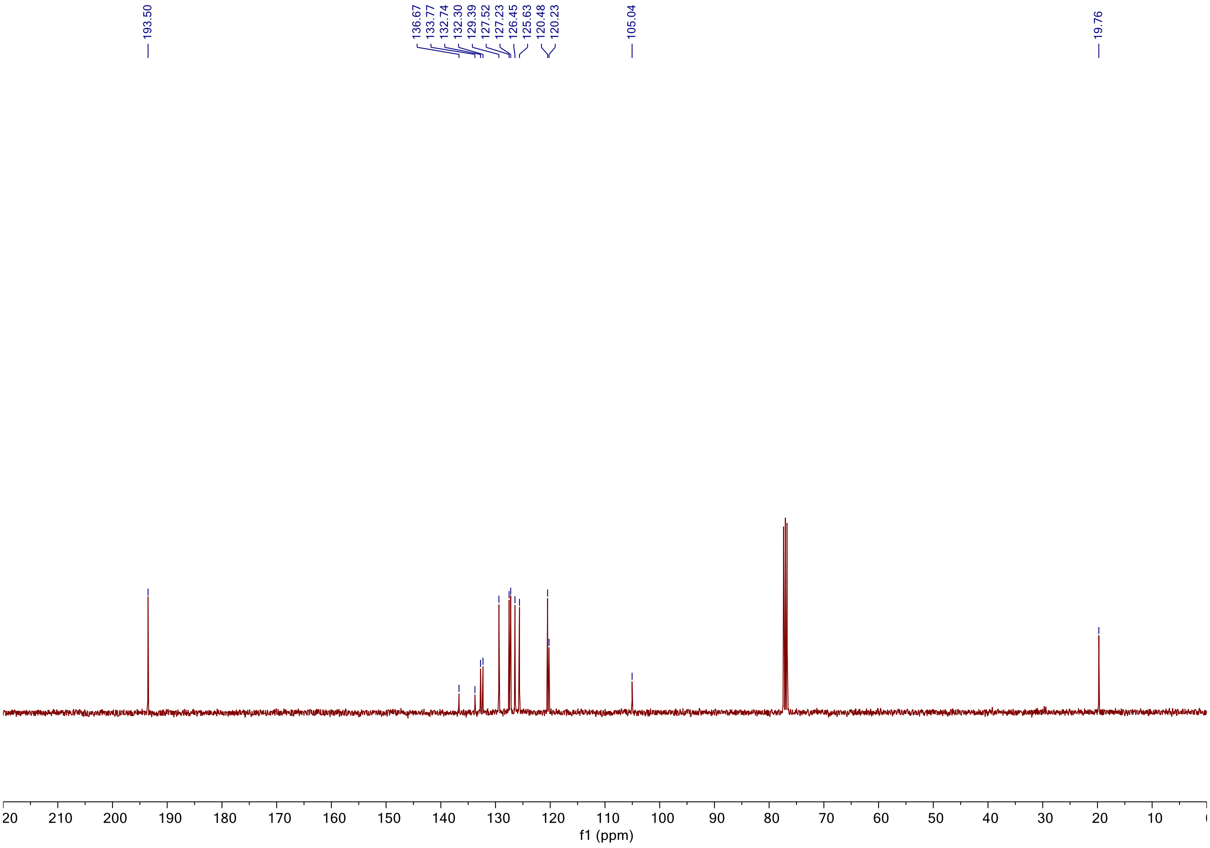

3-(Methylthio)-1'-tosyl-1*H*,1'*H*-[2,3'-biindole]-7-carbaldehyde (5f) in $CDCl_3$ ^1H-NMR and ^{13}C-NMR

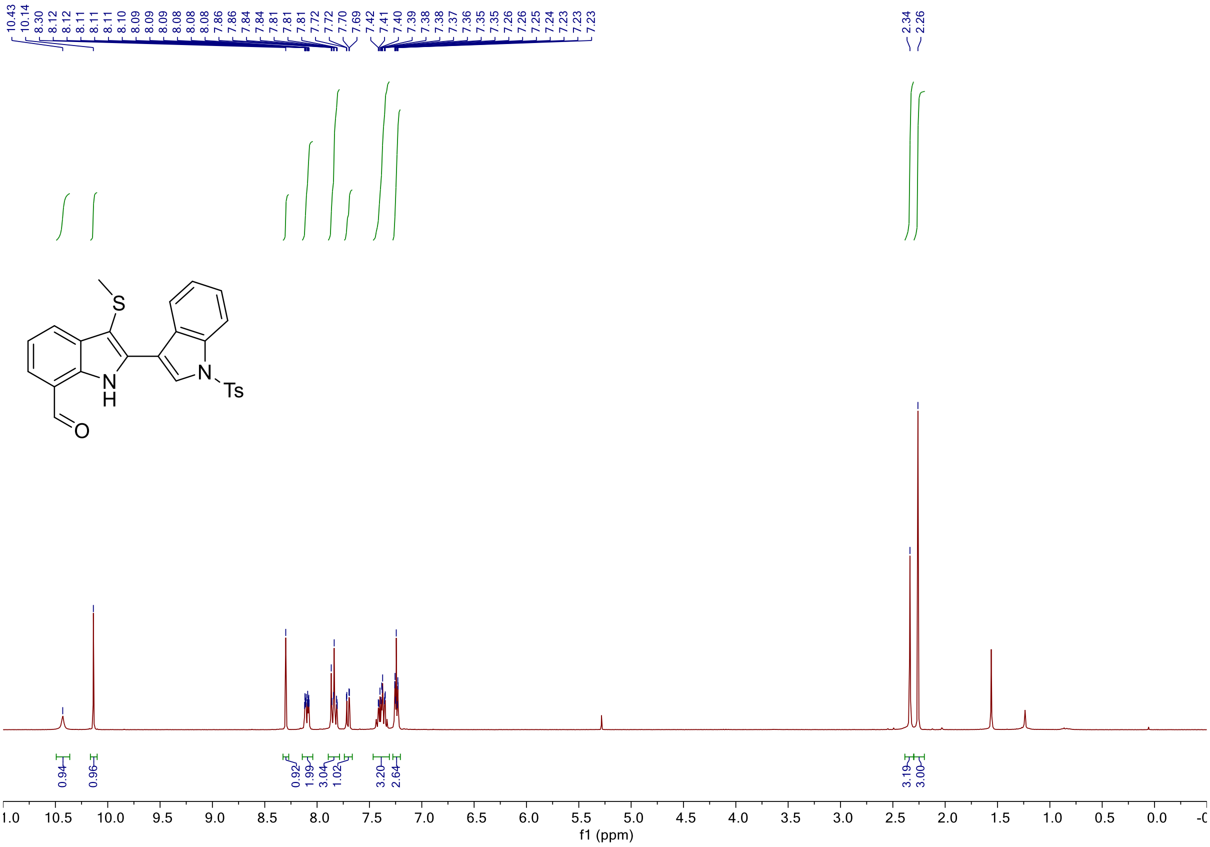

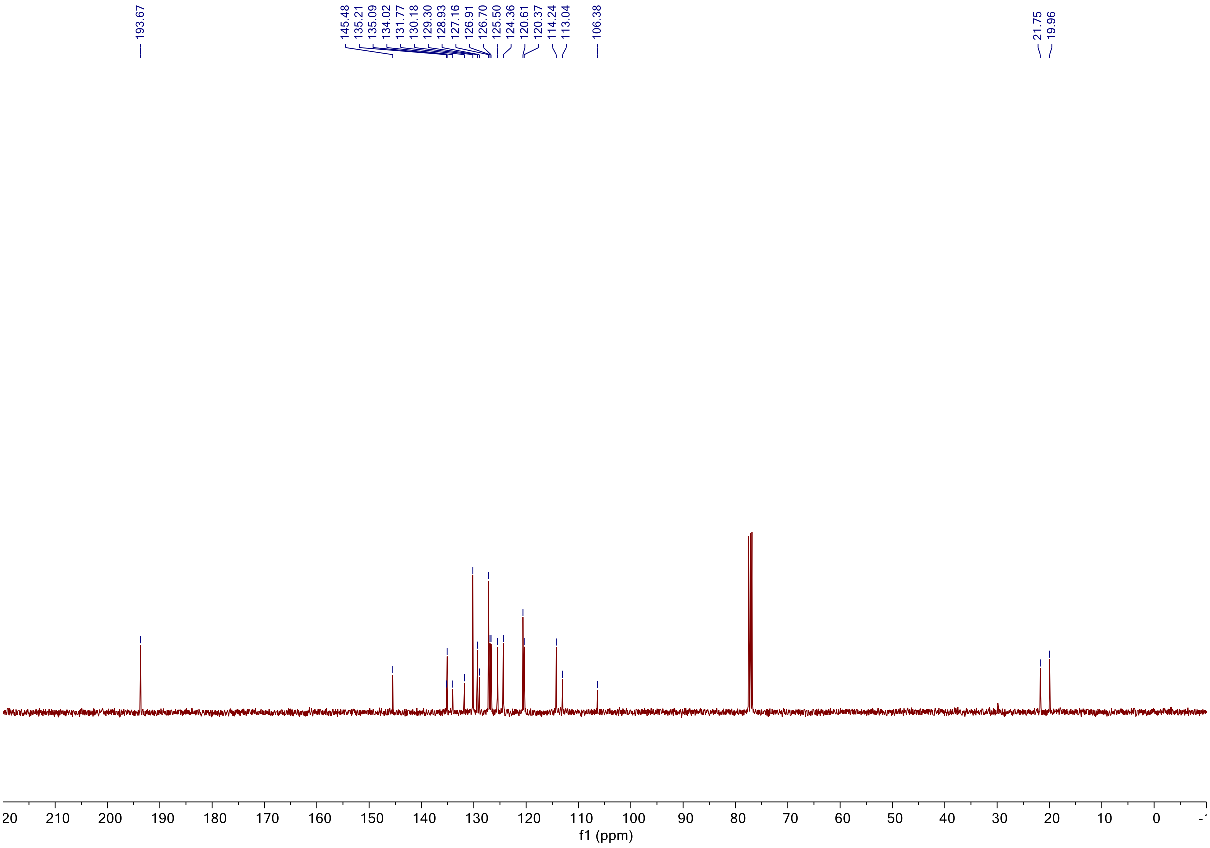

2-(4-Methoxyphenyl)-5-methyl-3-(methylthio)-4-phenyl-1*H*-pyrrole (9) in $CDCl_3$ ^{1}H-NMR and ^{13}C-NMR

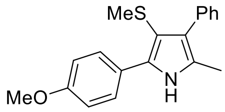

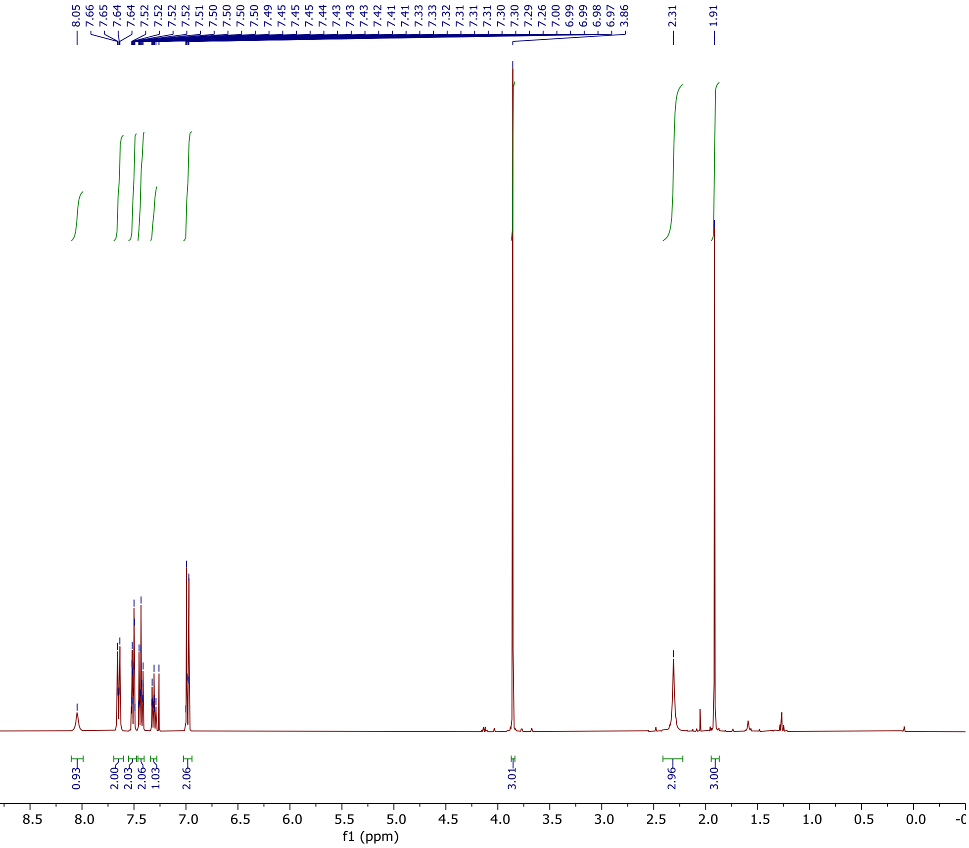

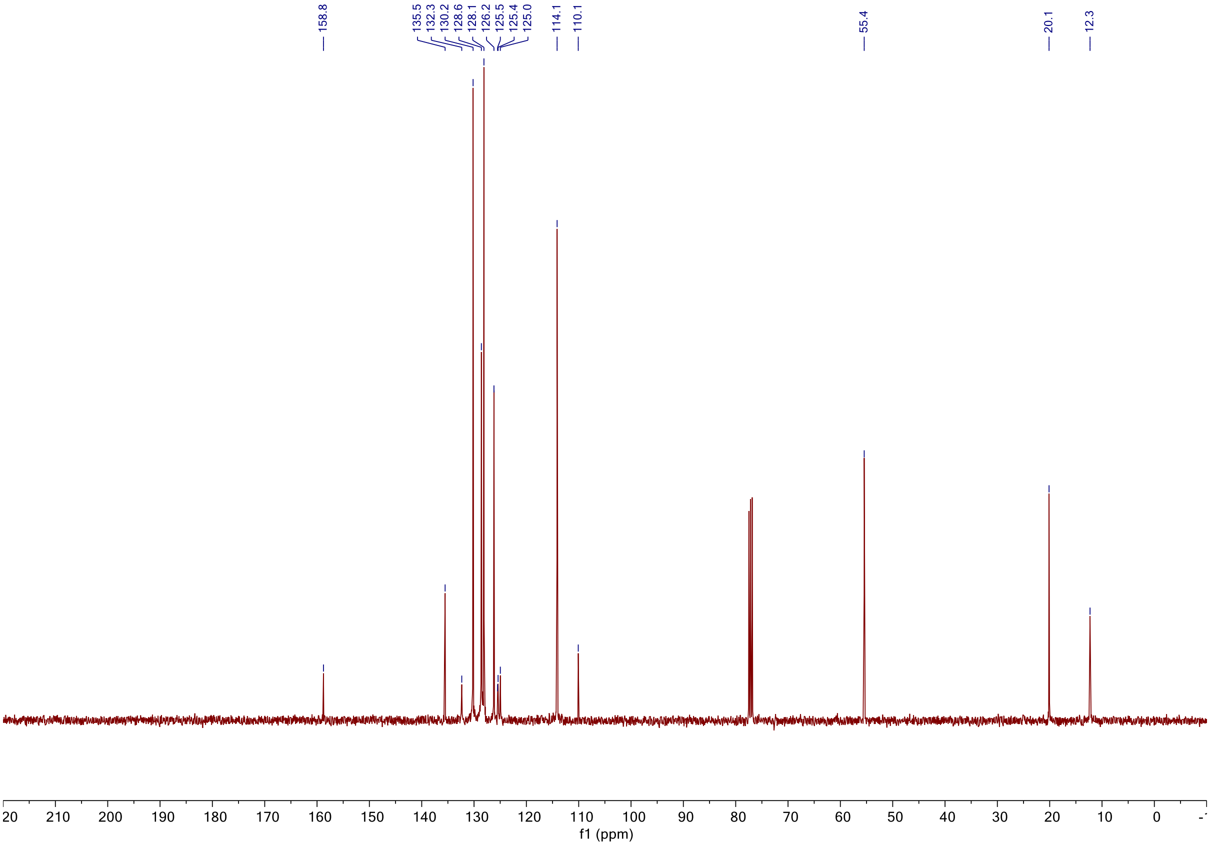

1-(2-(4-Methoxyphenyl)-5-methyl-4-phenyl-1*H*-pyrrol-3-yl)ethan-1-one (8b) in $CDCl_3$ ^{1}H-NMR and ^{13}C-NMR

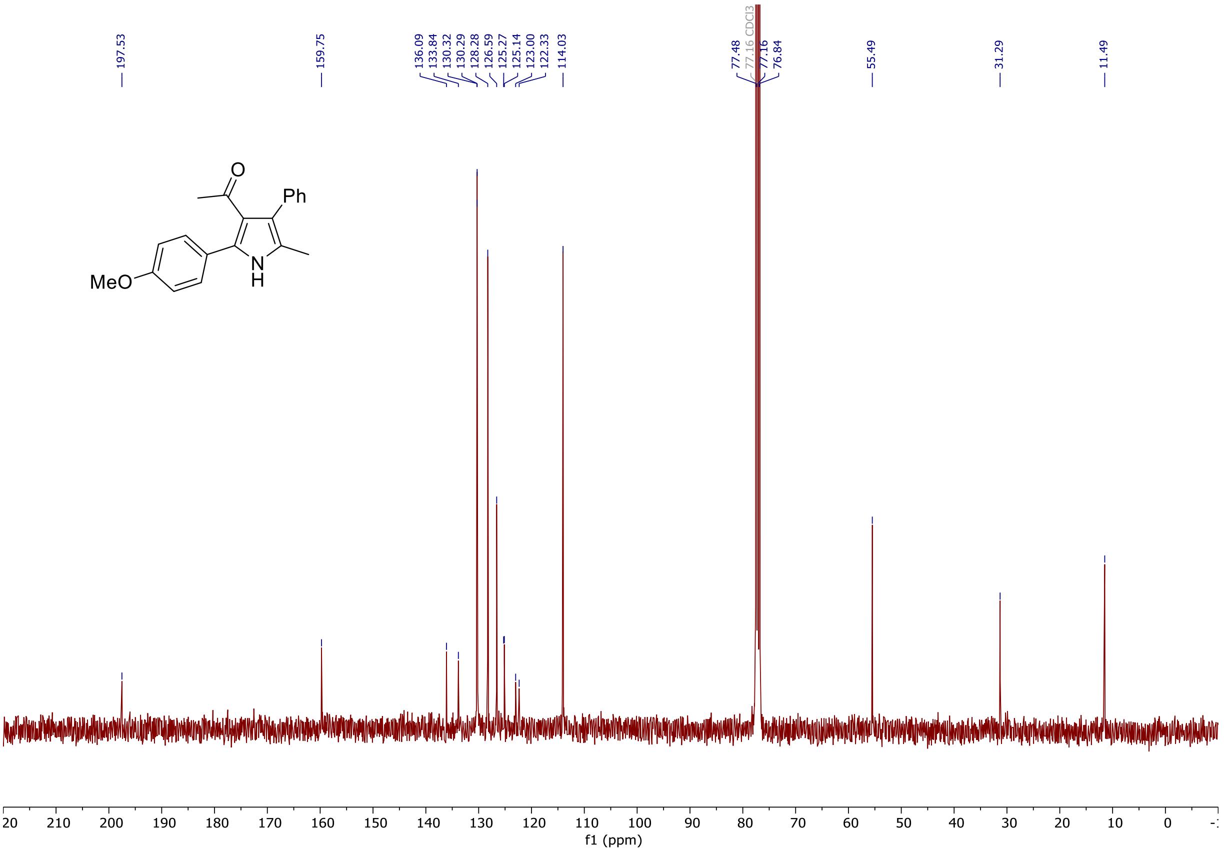

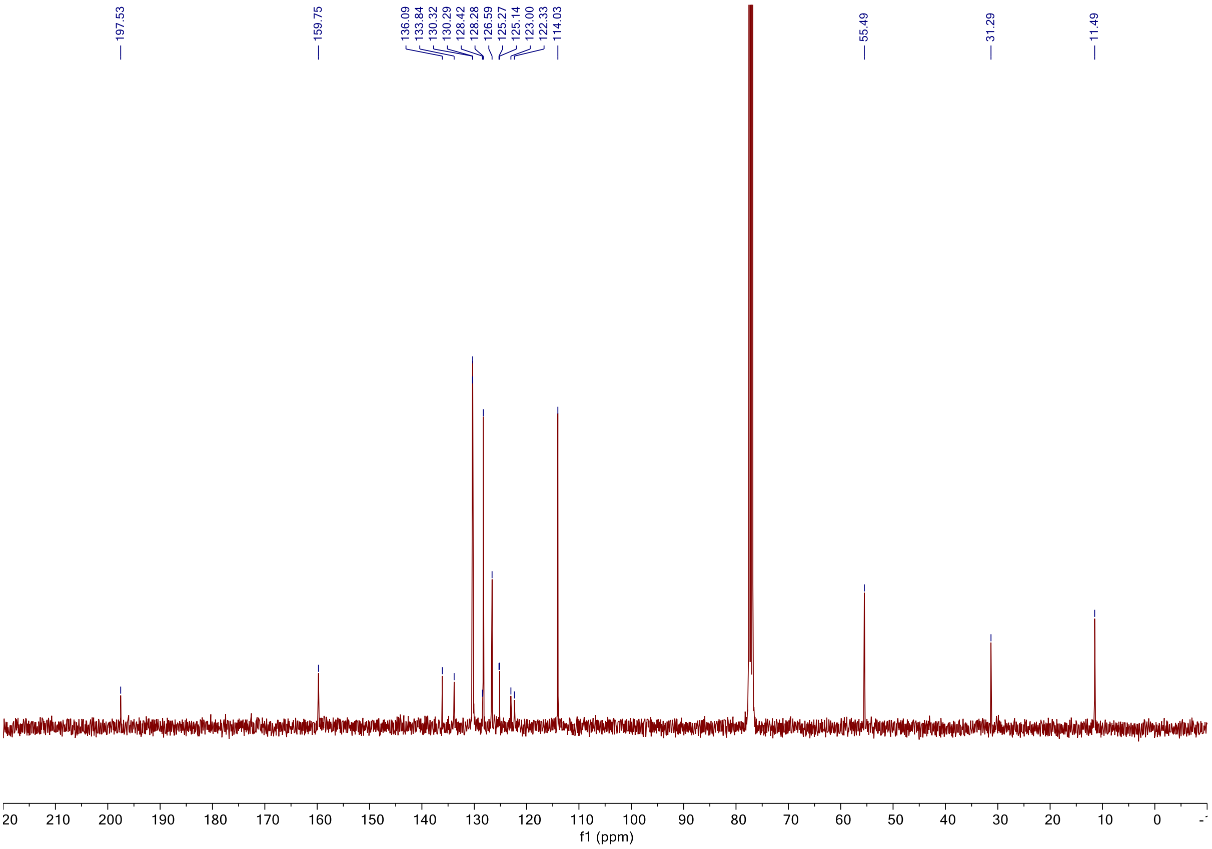

2-(4-Methoxyphenyl)-4-methyl-3-(methylthio)-5-phenyl-1*H*-pyrrol-1-yl)(phenyl)methanone (7) in $CDCl_3$ ^{1}H-NMR and ^{13}C-NMR

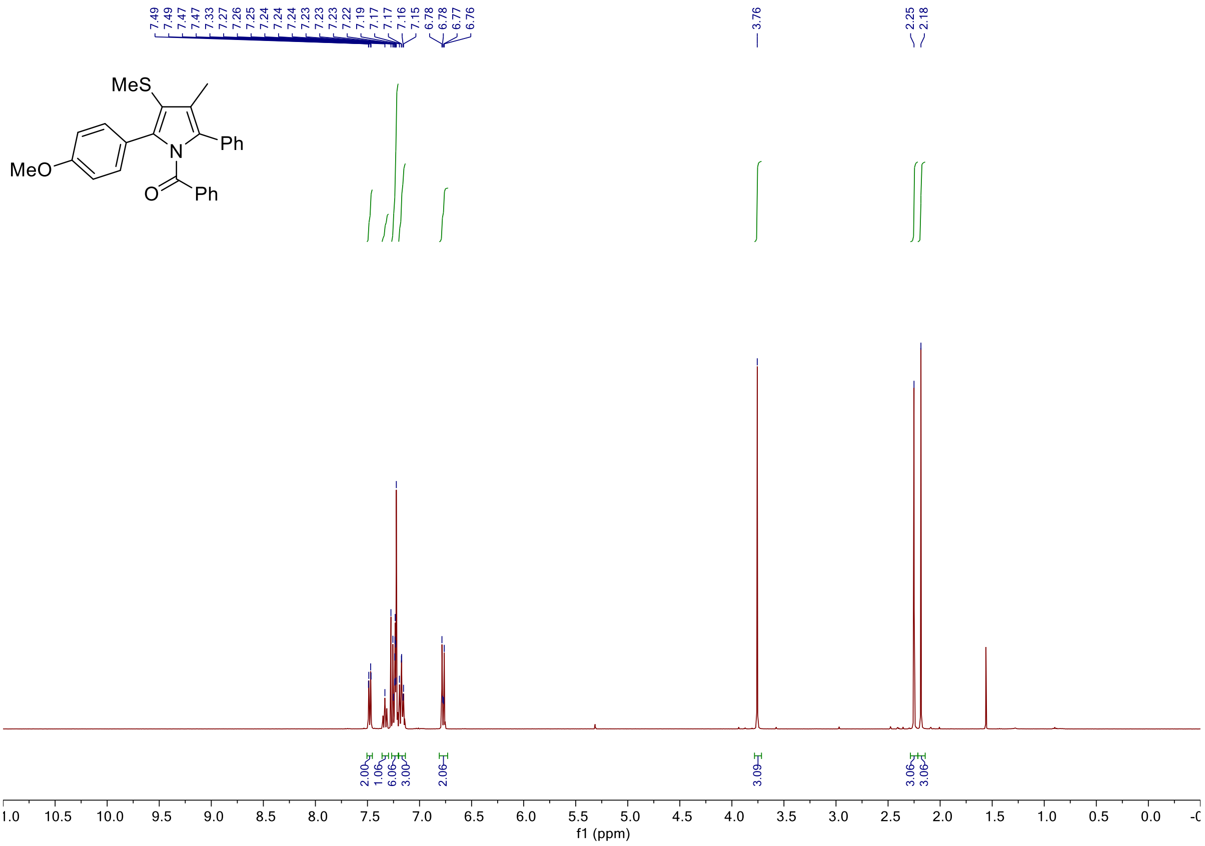

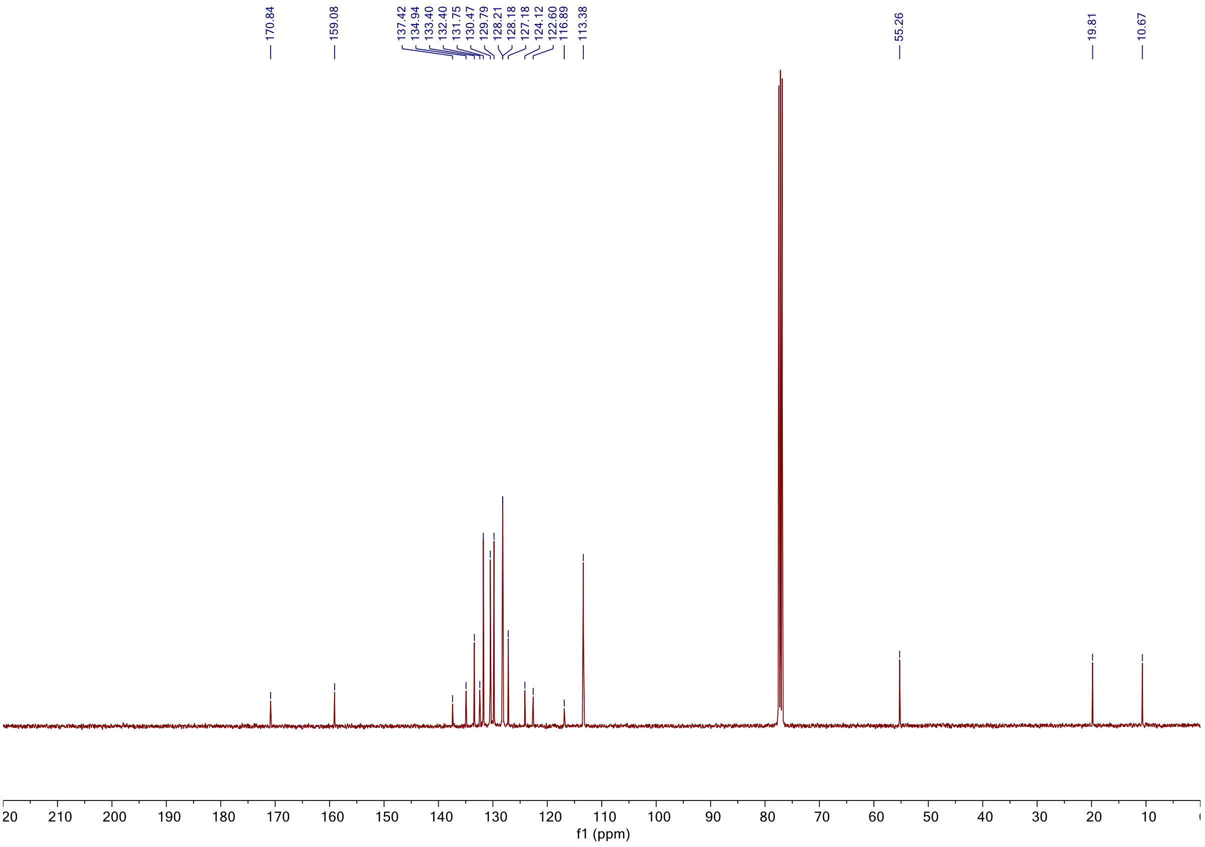

(2-(4-Methoxyphenyl)-4-methyl-5-phenyl-1*H*-pyrrol-3-yl)(phenyl)methanone (8a) in $CDCl_3$ ^{1}H-NMR and ^{13}C-NMR

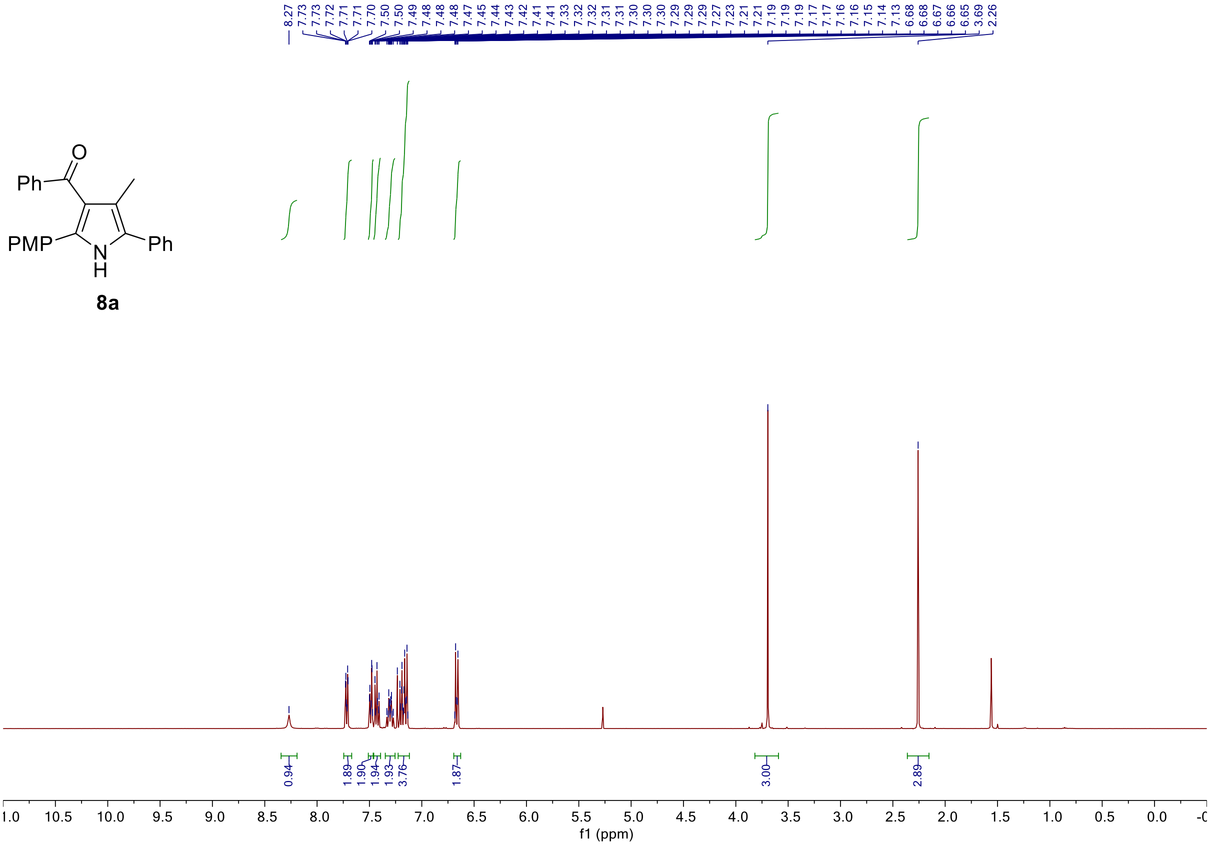

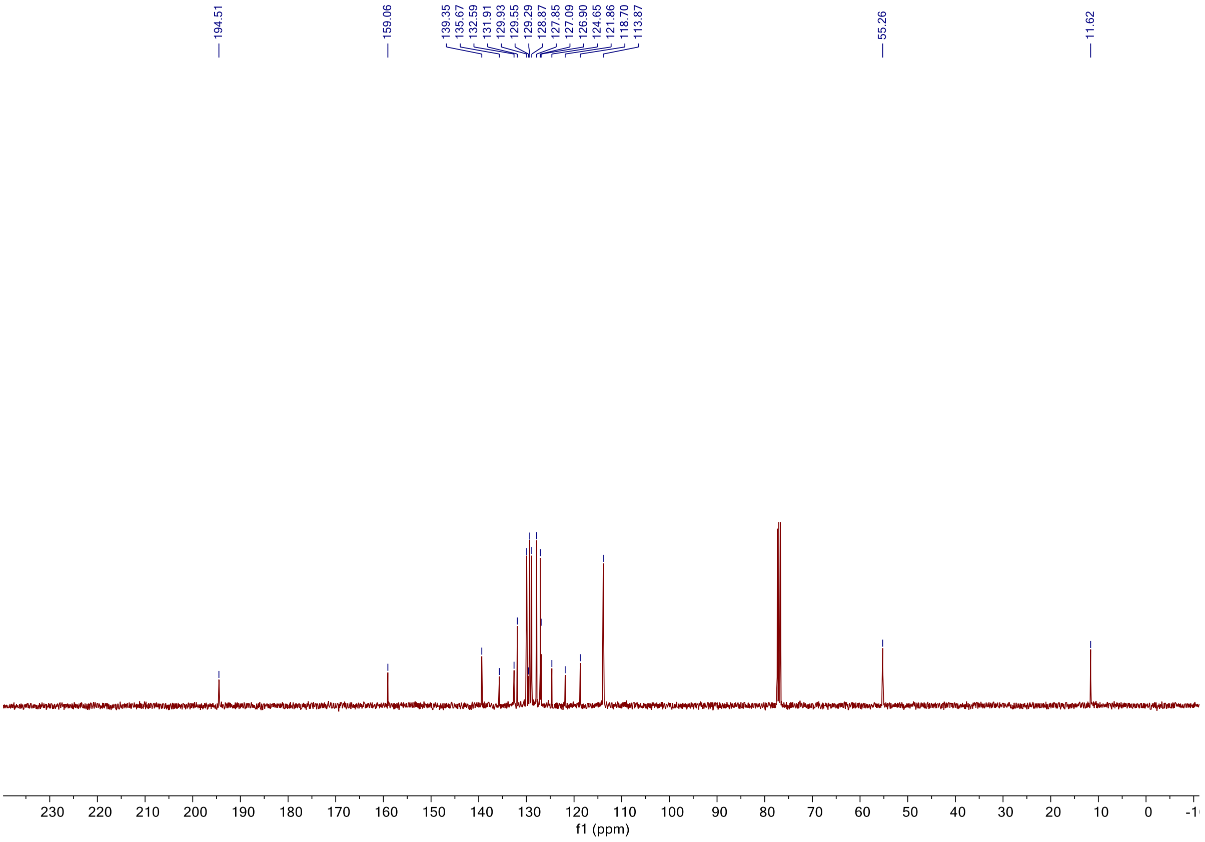

1-(2-Methyl-4-(methylthio)-5-phenyl-1*H*-pyrrol-3-yl-5-^{13}*C*)ethan-1-one (^{13}C-3l) in $CDCl_3$ ^{1}H-NMR and ^{13}C-NMR

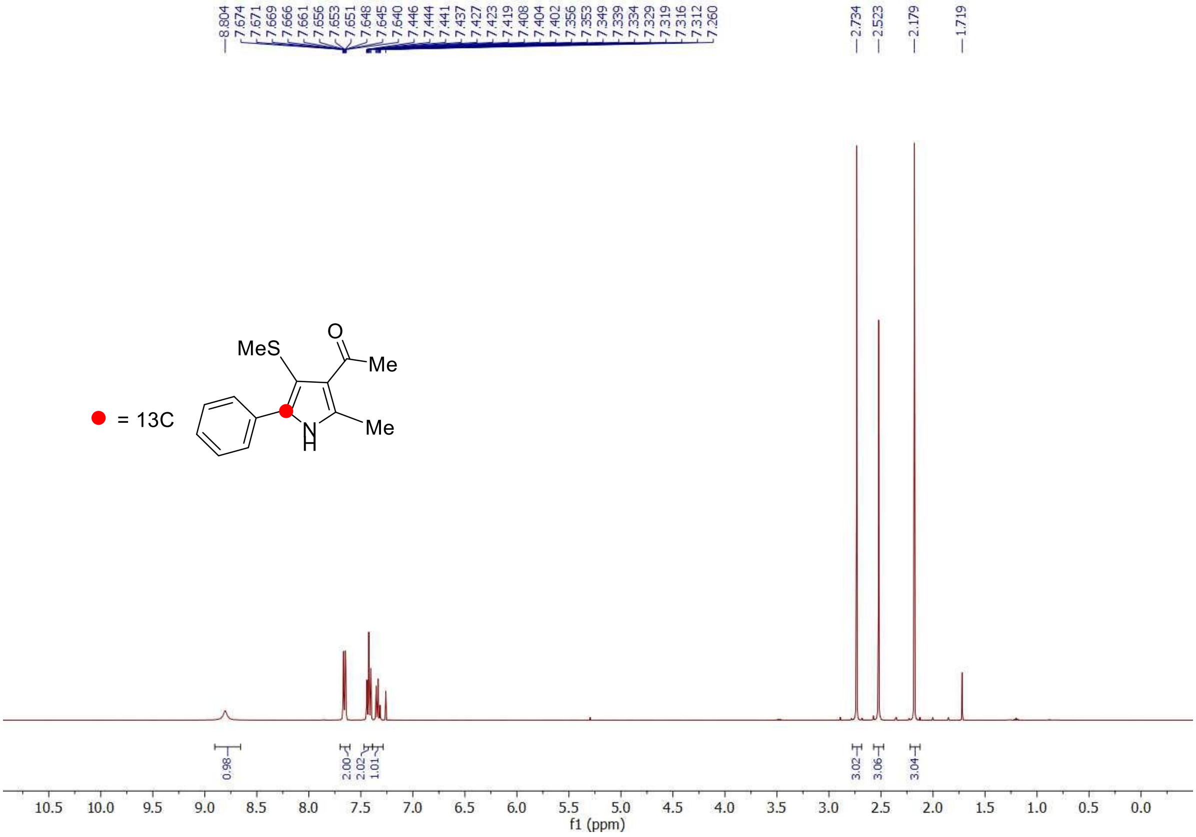

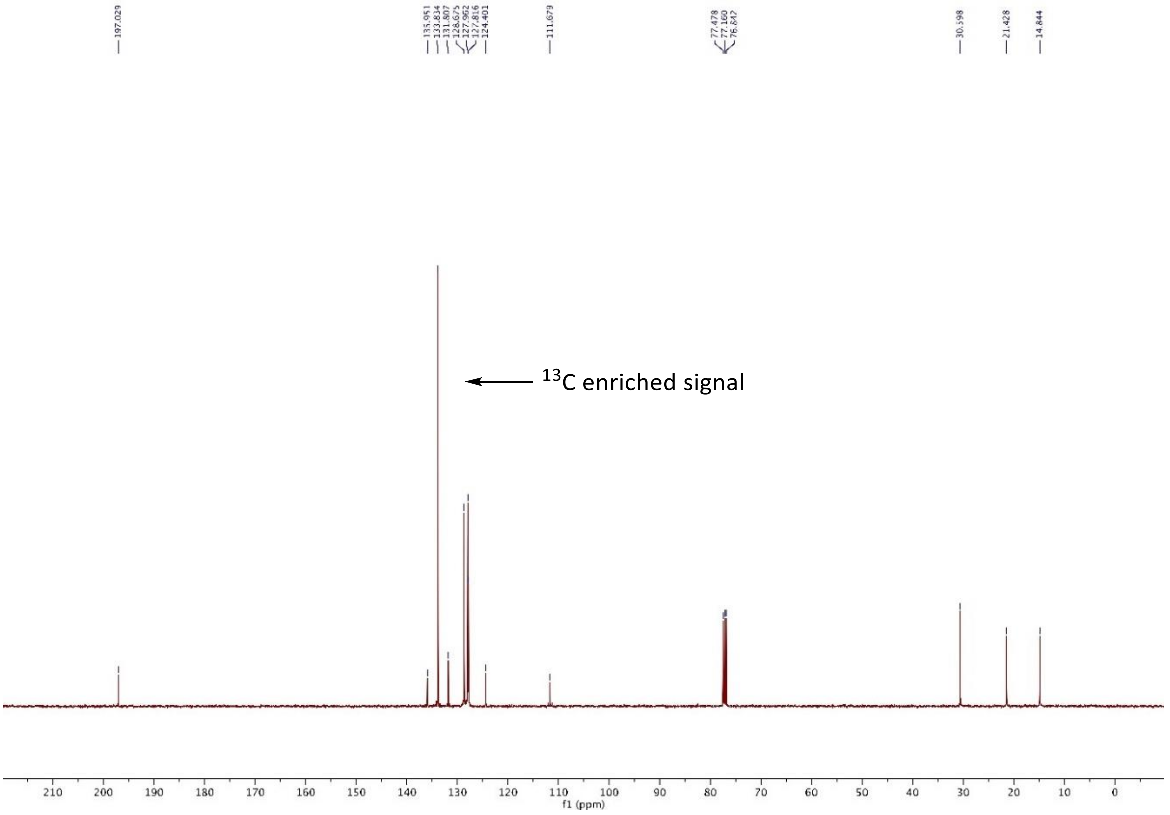

^{13}C-3l ^{1}H-^{13}C HMBC

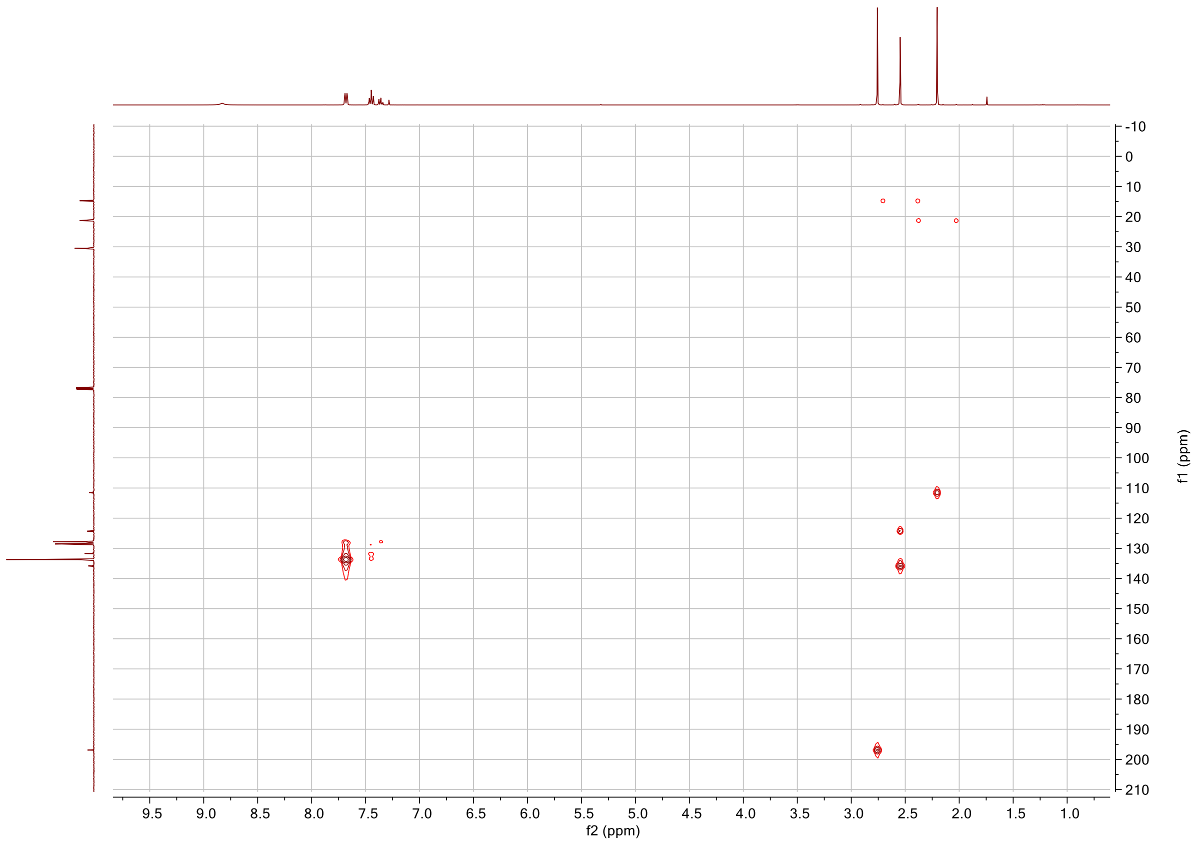

^{13}C-3l ^{1}H-^{13}C HMBC (Reduced intensity)

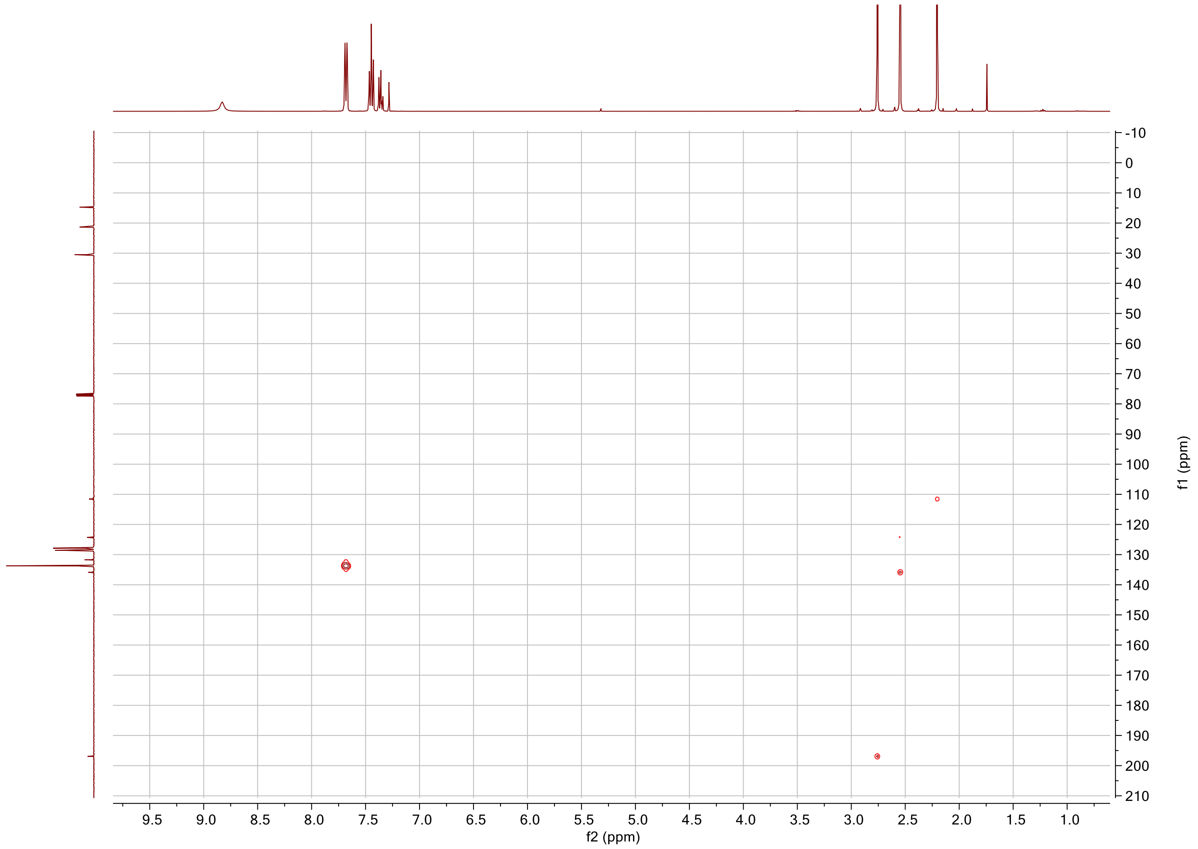

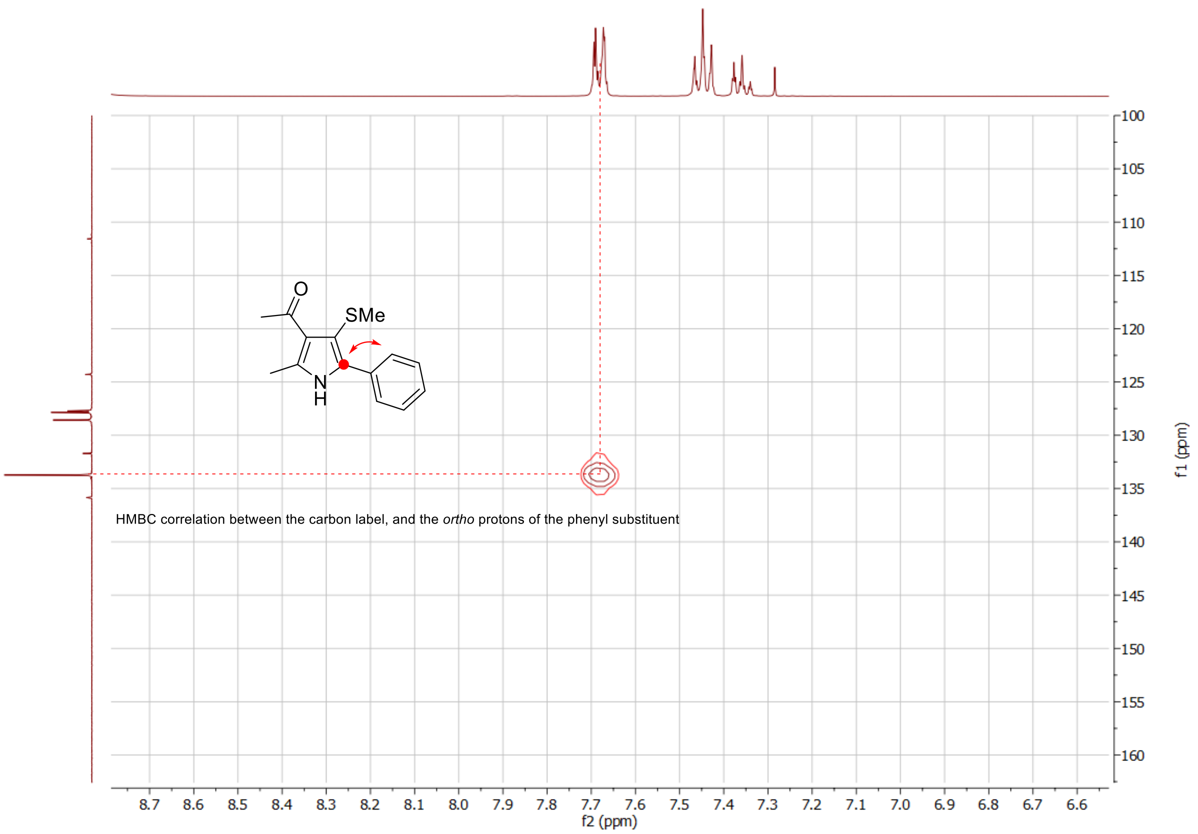
O
SMe
N
H
HMBC correlation between the carbon label, and the *ortho* protons of the phenyl substituent
f1 (ppm)
f2 (ppm)
100
105
110
115
120
125
130
135
140
145
150
155
160
8.7
8.6
8.5
8.4
8.3
8.2
8.1
8.0
7.9
7.8
7.7
7.6
7.5
7.4
7.3
7.2
7.1
7.0
6.9
6.8
6.7
6.6

(*Z*)-2-(Methylthio)-*N*-(4-oxopent-2-en-2-yl)-2-phenylacetamide-2-^{13}C (^{13}C-13) in $CDCl_3$ ^{1}H-NMR and ^{13}C-NMR

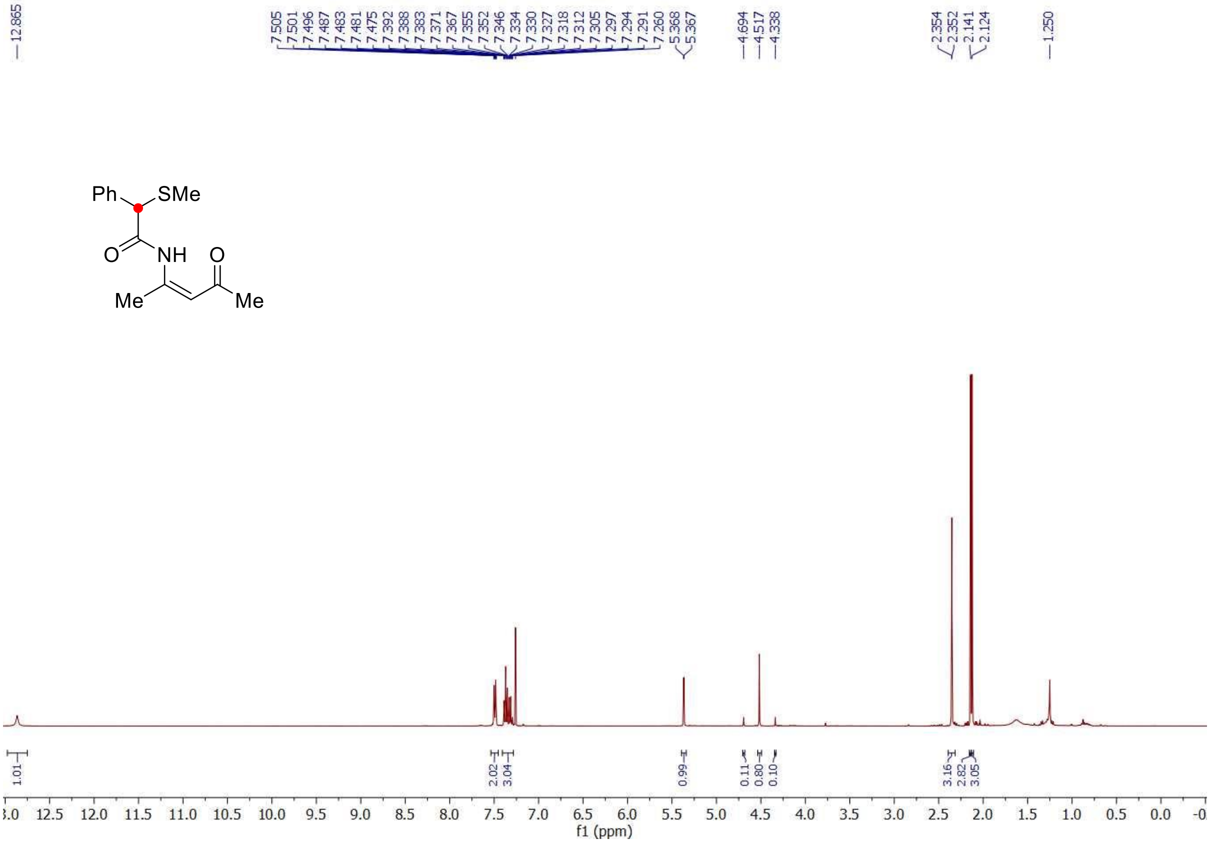

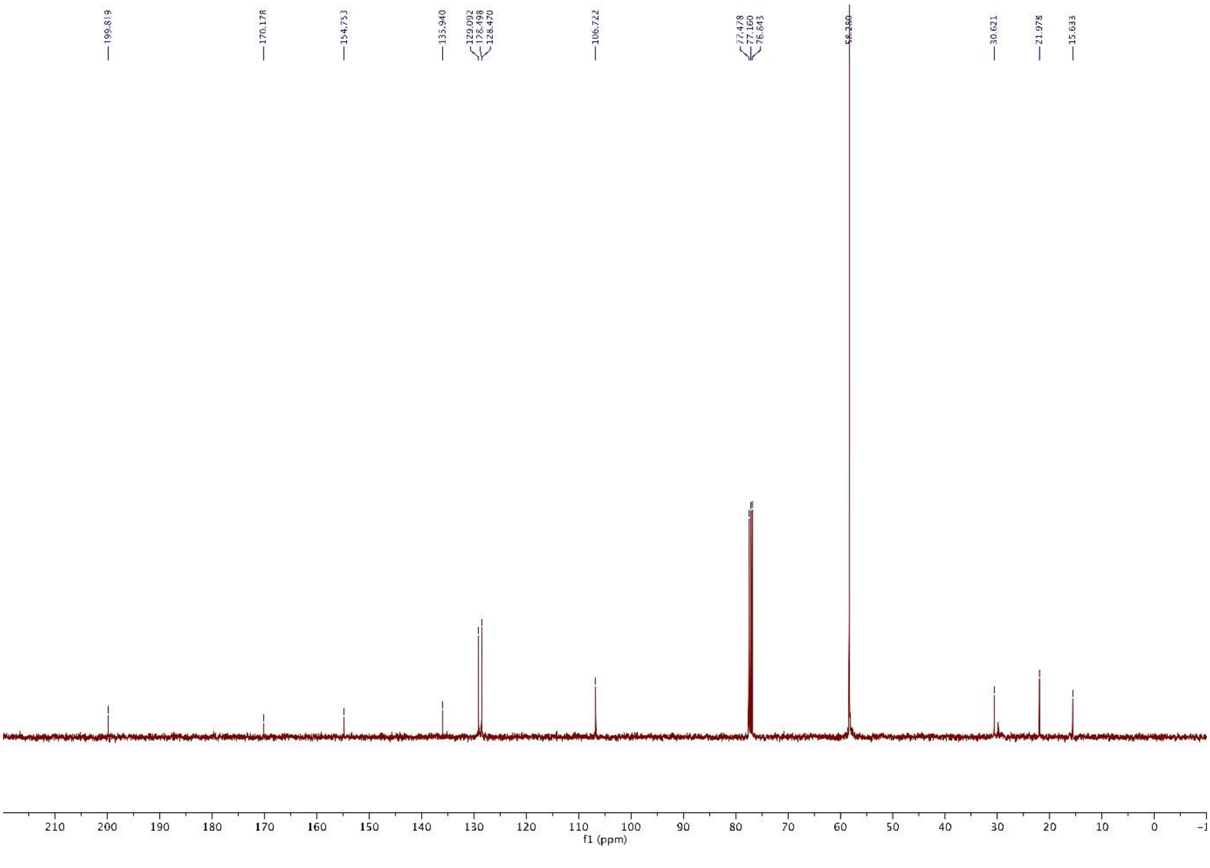

1-(5-(4-Methoxyphenyl)-2-methyl-4-(sulfonyl)-1*H*-pyrrol-3-yl)ethan-1-one (10) in DMSO-d_6 ^{1}H-NMR and ^{13}C-NMR

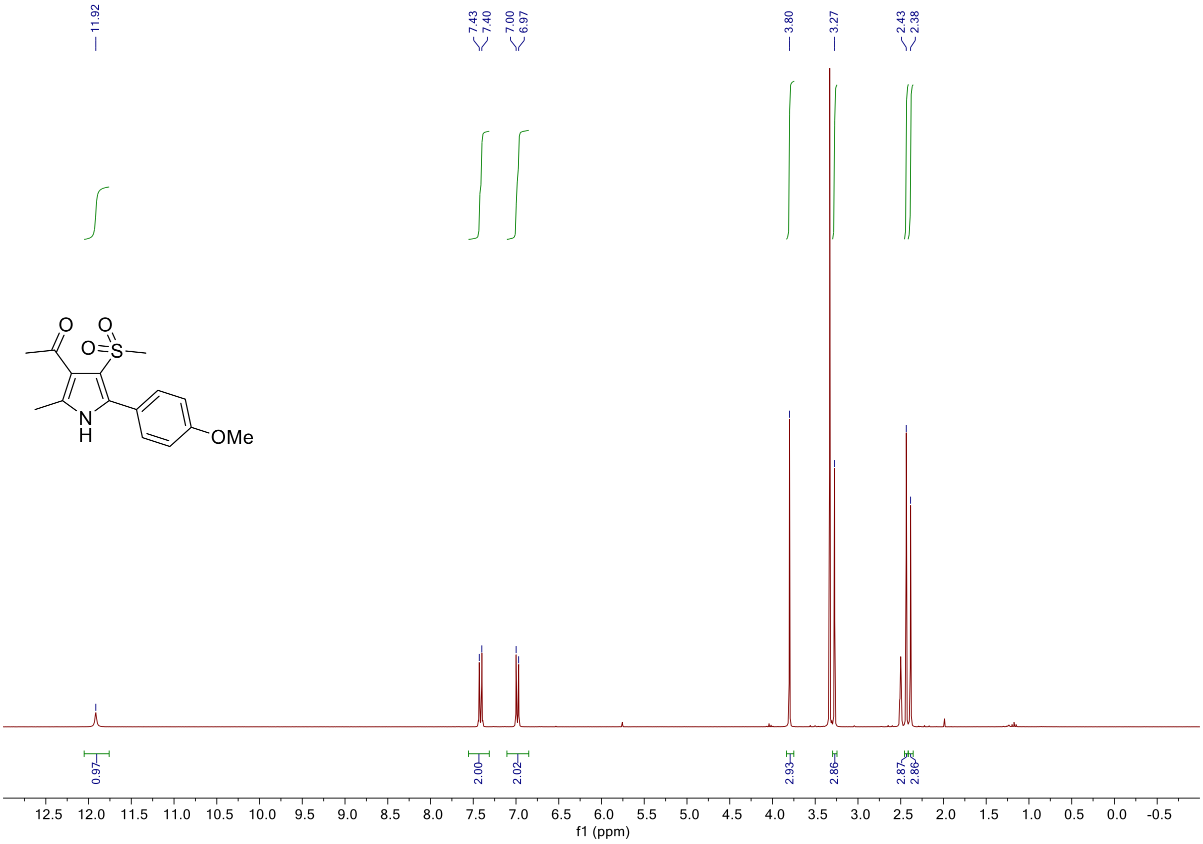

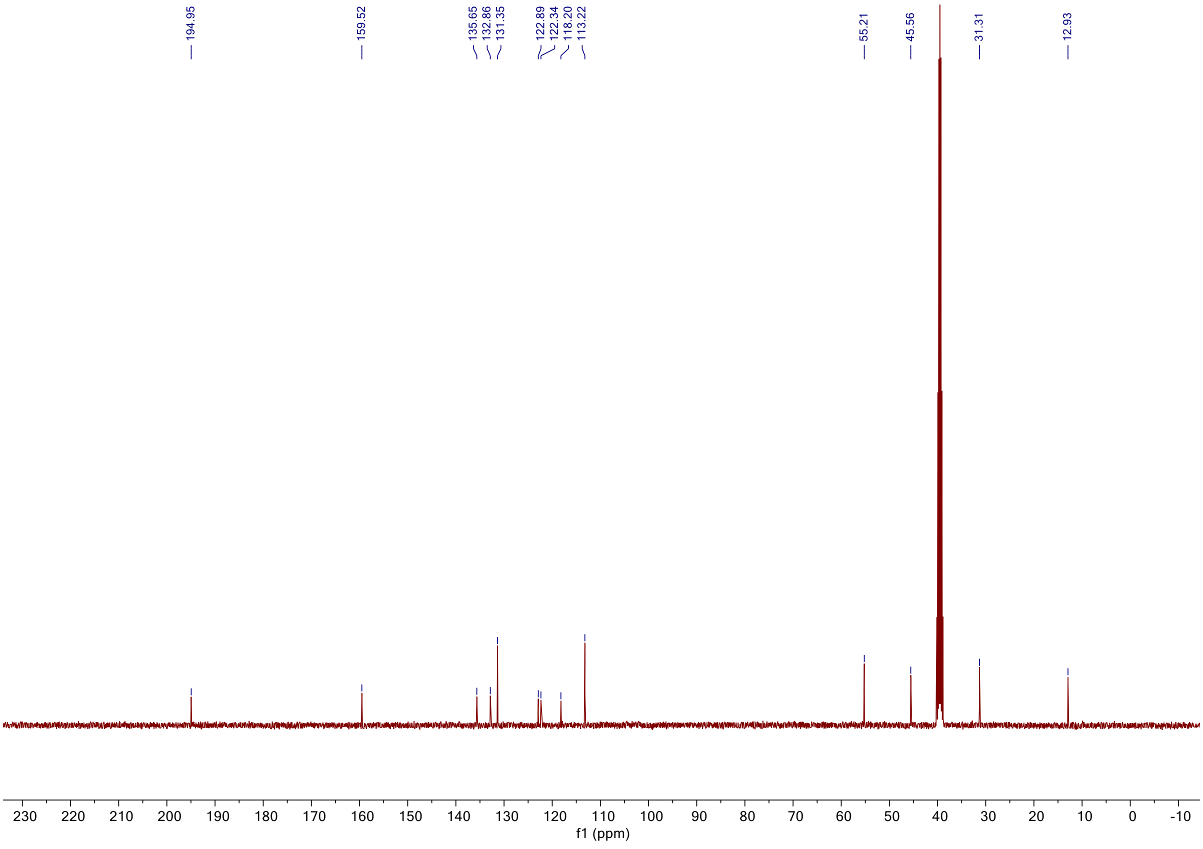

1-(5-(4-Methoxyphenyl)-2-methyl-4-(methylsulfinyl)-1*H*-pyrrol-3-yl)ethan-1-one (11) in DMSO-*d*$_6$ ^{1}H-NMR and ^{13}C-NMR

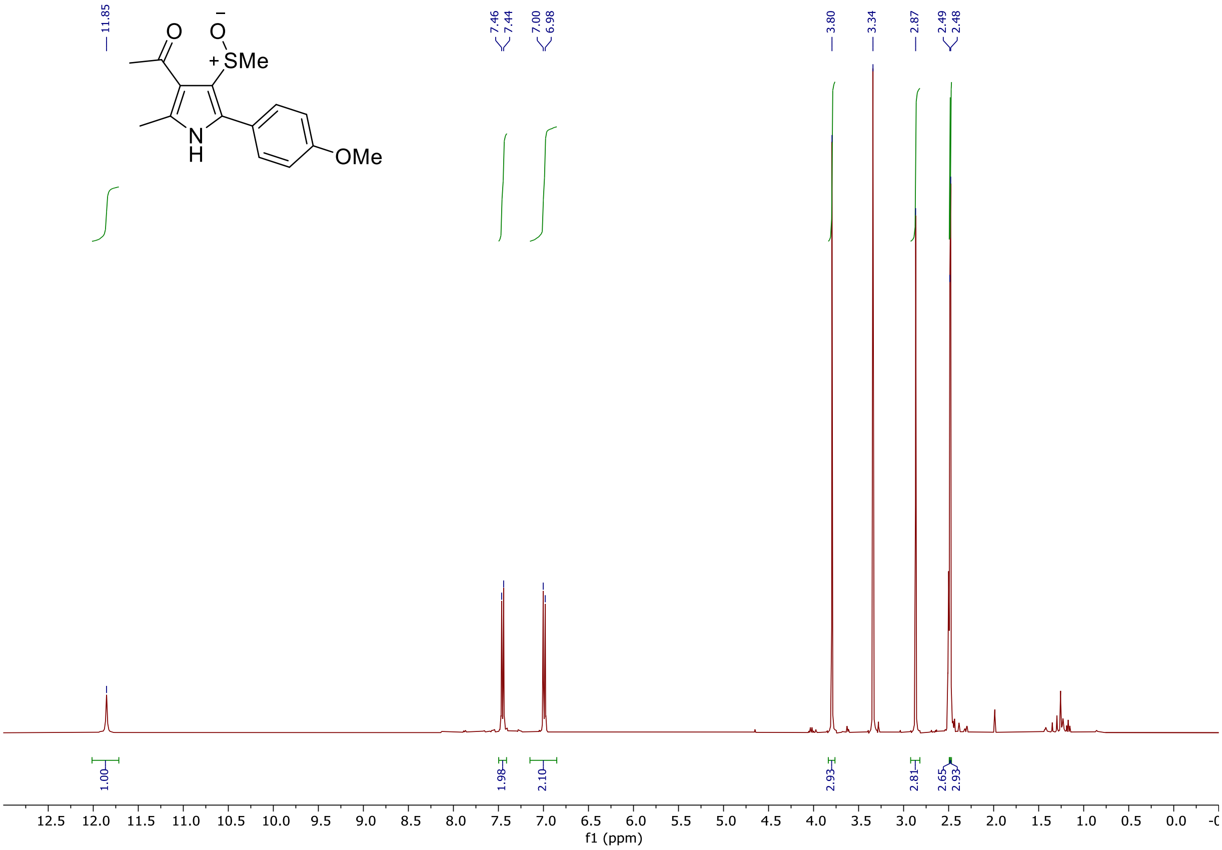

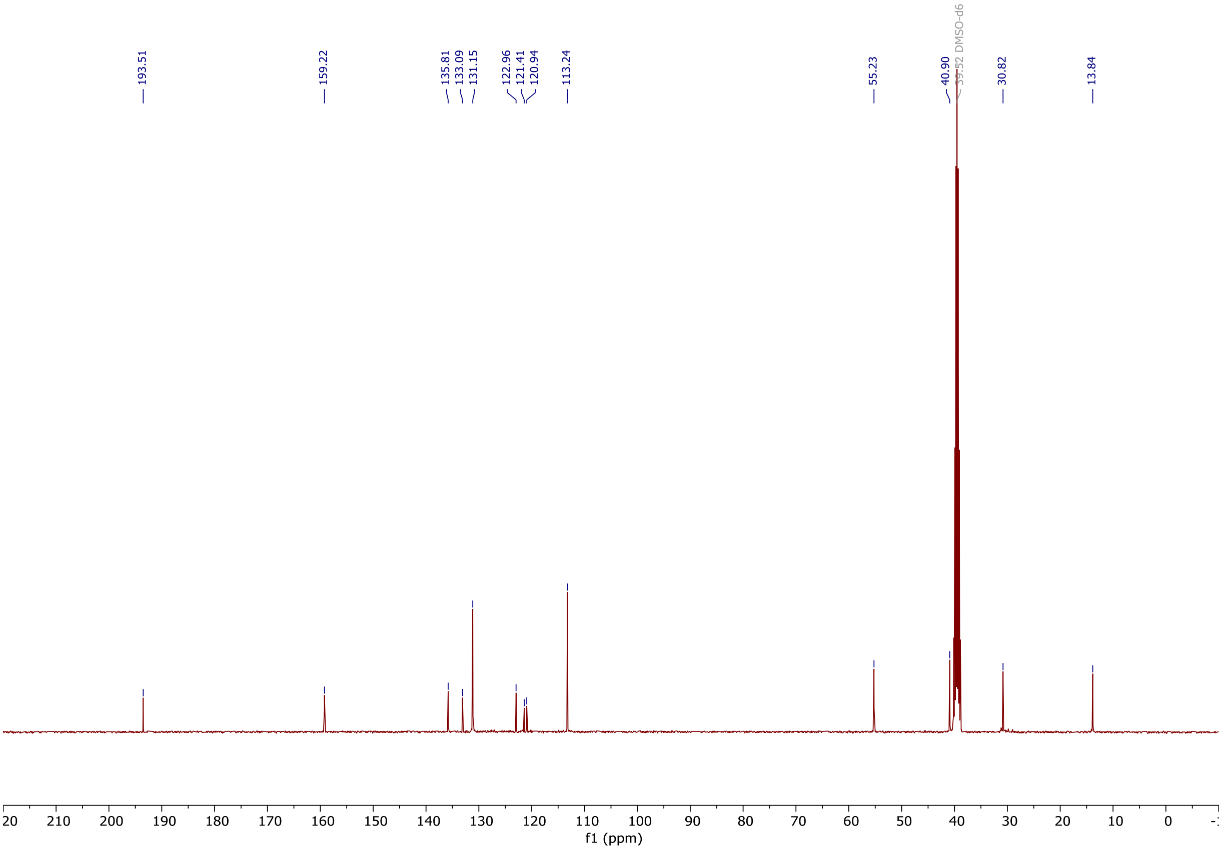

1-(5-(4-Methoxyphenyl)-2-methyl-1*H*-pyrrol-3-yl)ethan-1-one (12) in $CDCl_3$ ^1H-NMR, ^{13}C-NMR, ^1H-^1H NOESY and ^1H-^1H NOESY (selected region)

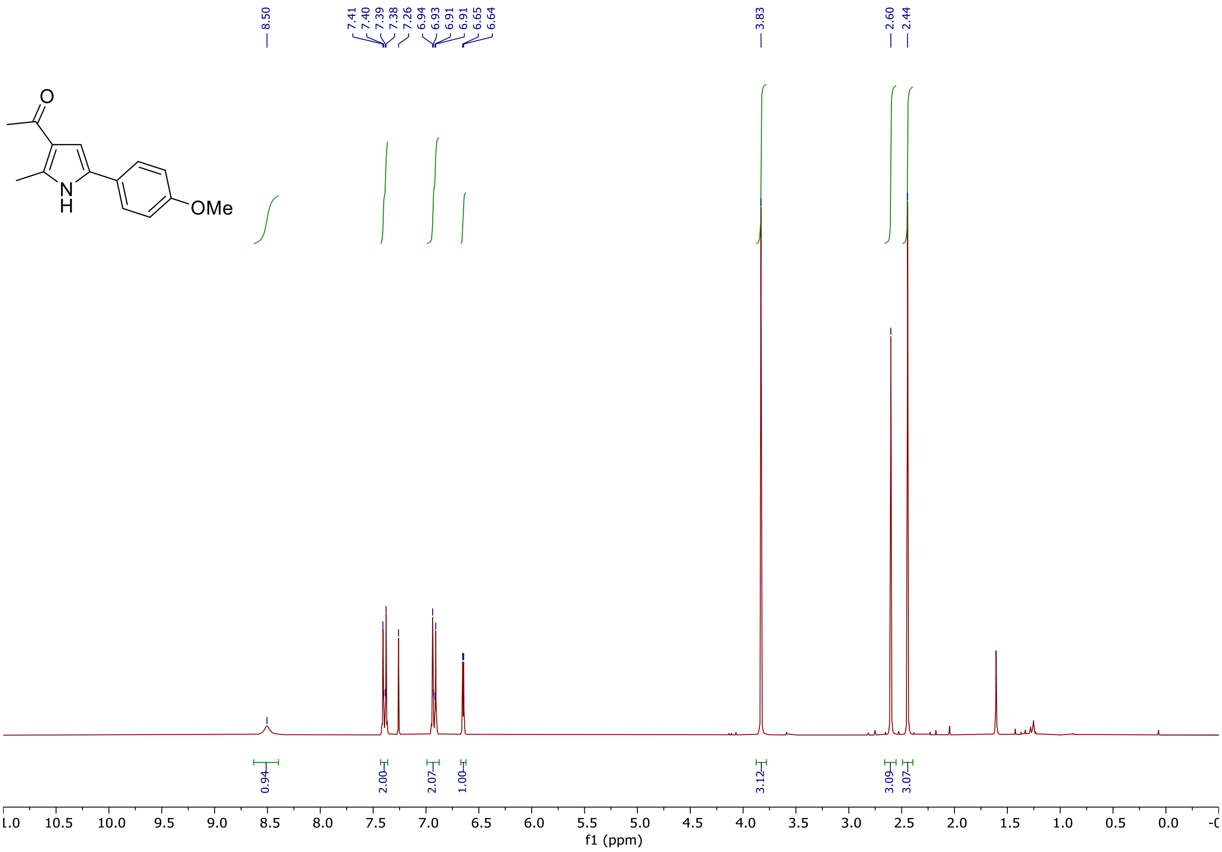

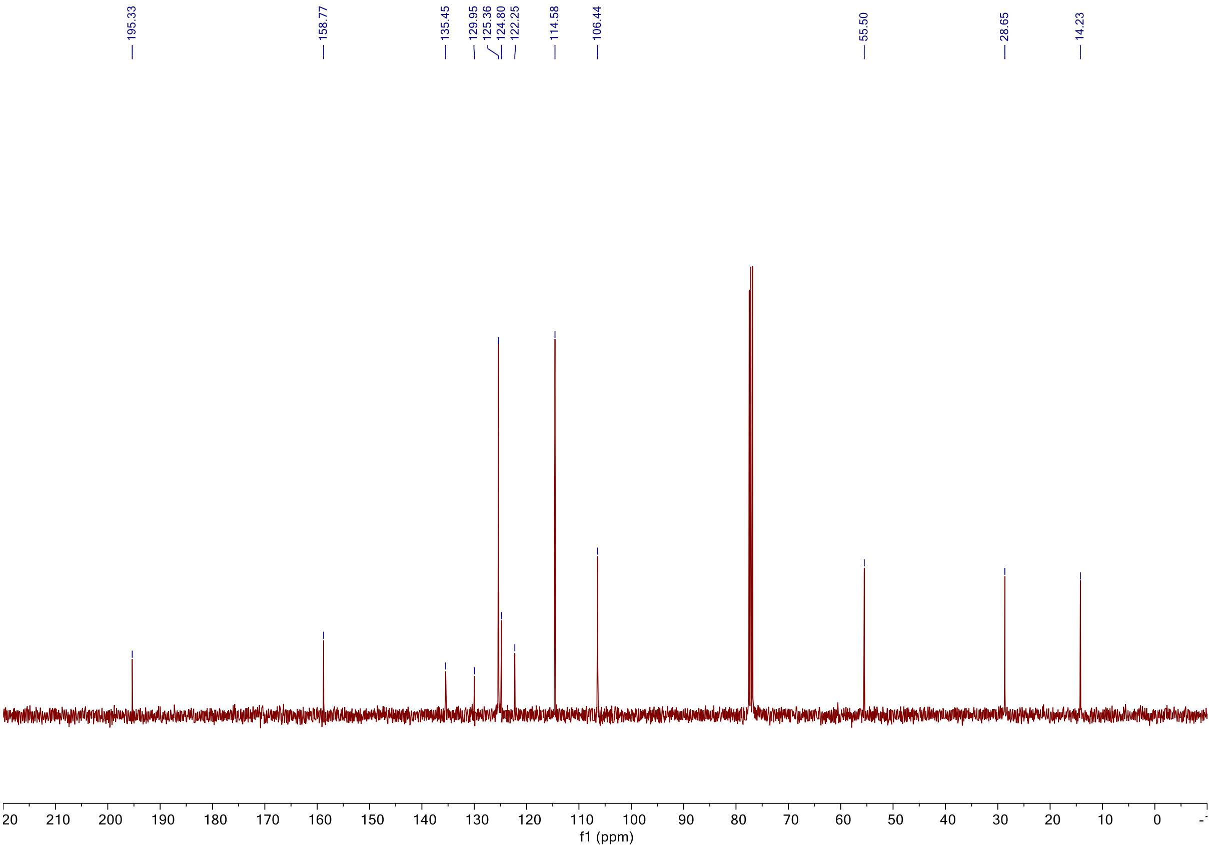

HMBC and HMBC annotated with methyl assignments of 12.

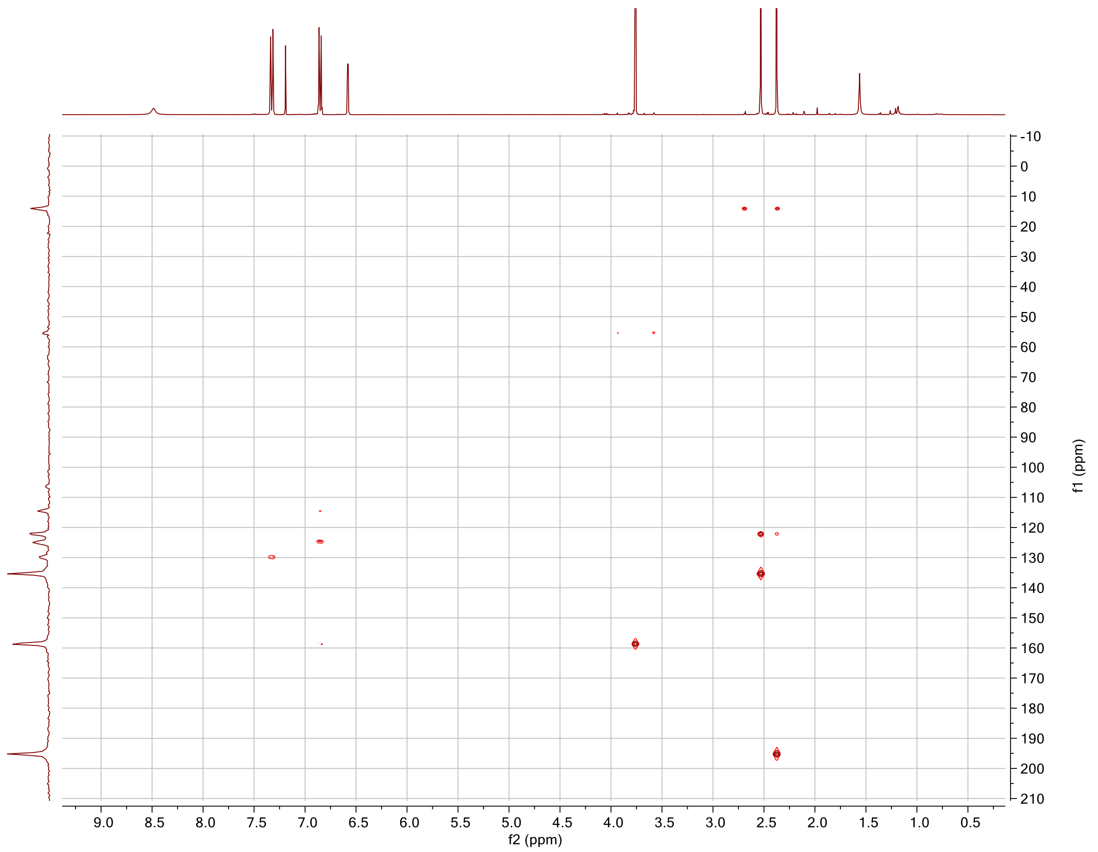

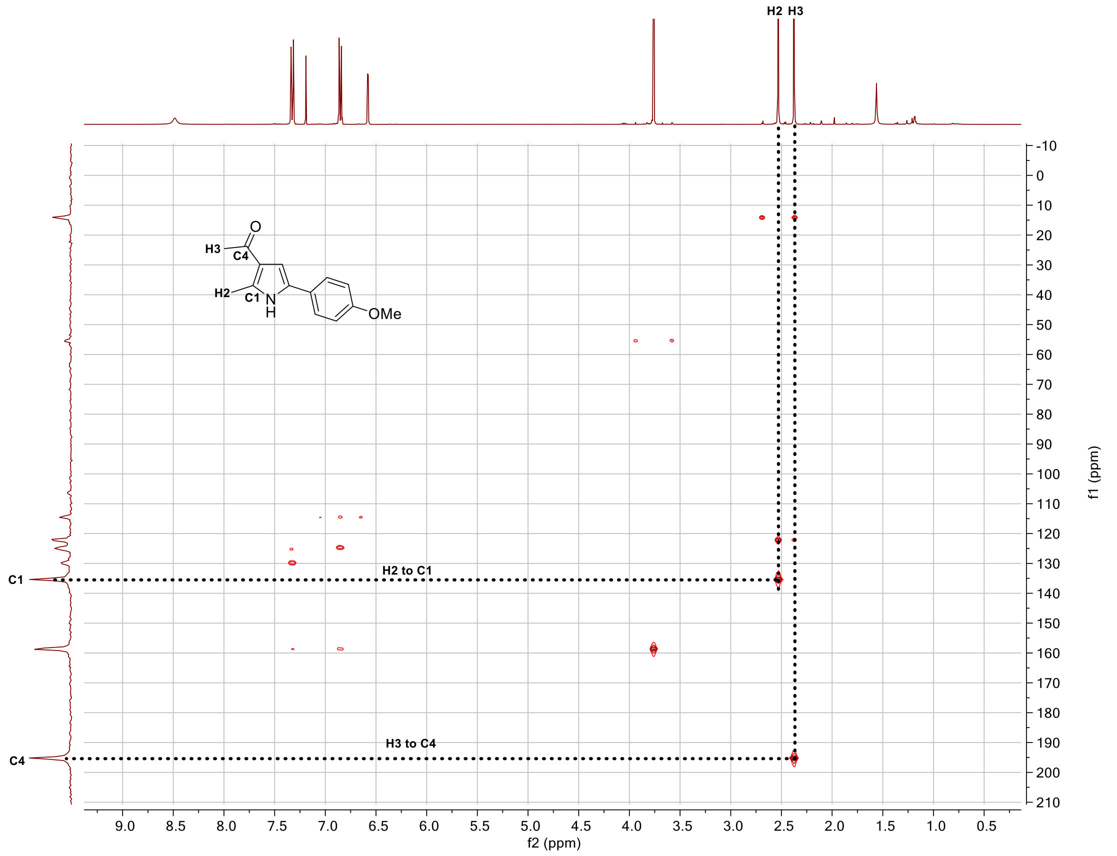

^1H-^1H NOESY and ^1H-^1H NOESY (annotated with diagnostic assignments) of 12.

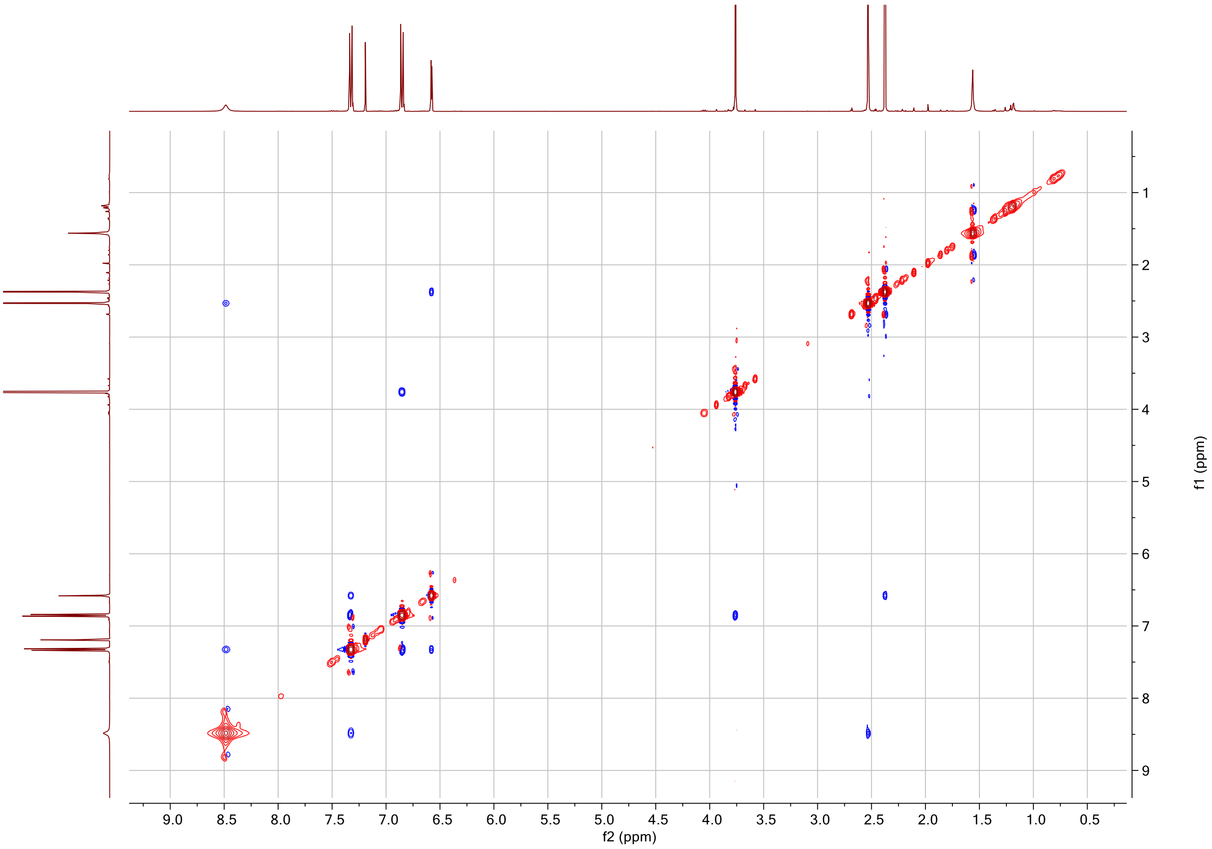

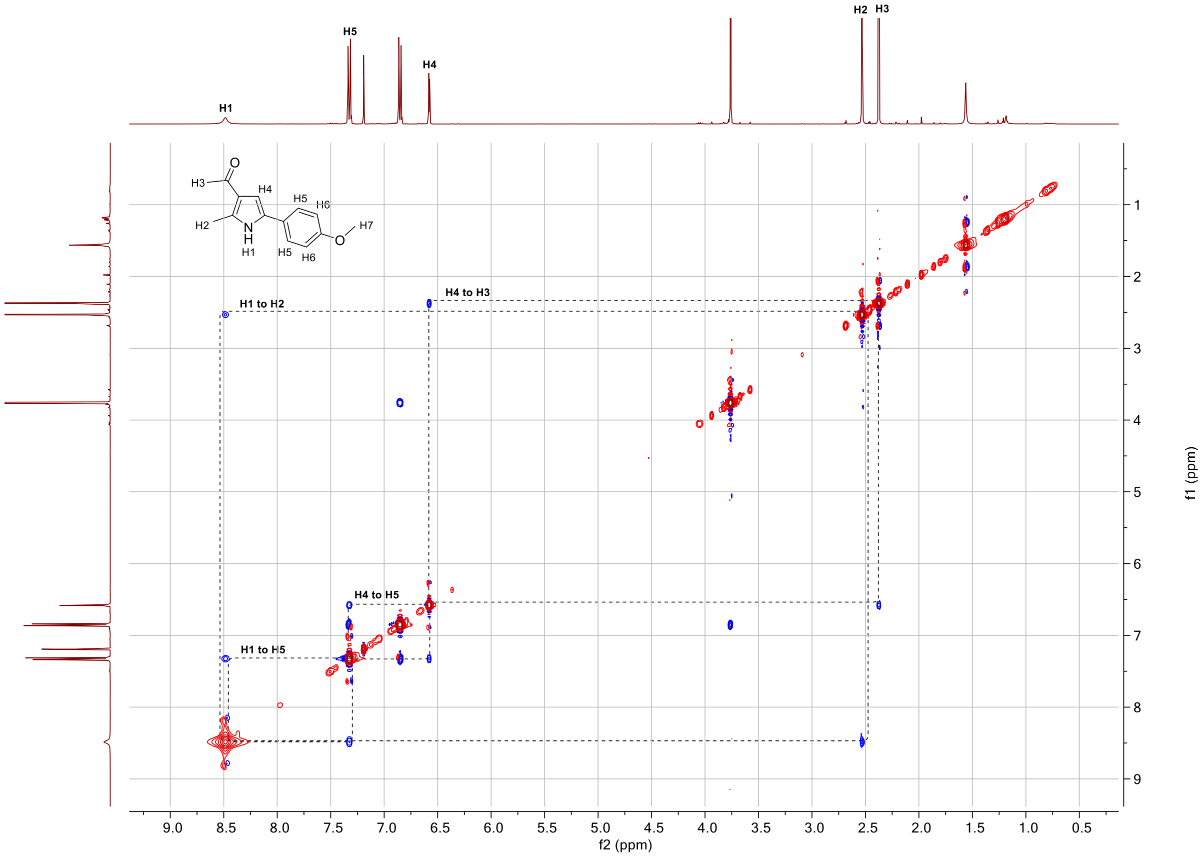

Supplement: Supplementary file 1 — cs1c01457_si_001.pdf [file cs1c01457_si_001.pdf]
